# Human induced pluripotent stem cell derived hepatocytes provide insights on parenteral nutrition associated cholestasis in the immature liver

**DOI:** 10.1038/s41598-021-90510-1

**Published:** 2021-06-11

**Authors:** T. Hang Nghiem-Rao, Courtney Pfeifer, Michelle Asuncion, Joshua Nord, Daniel Schill, Kirthi Pulakanti, Shailendra B. Patel, Lisa A. Cirillo, Sridhar Rao

**Affiliations:** 1 Department of Pediatrics, Medical College of Wisconsin, 999 N. 92 Street, PO Box 1997, Milwaukee, WI 53226 USA; 2 Department of Cell Biology, Neurobiology, and Anatomy, Medical College of Wisconsin, Milwaukee, WI USA; 3 Blood Research Institute, Versiti, Milwaukee, WI USA; 4 Department of Medicine, Medical College of Wisconsin, Milwaukee, WI USA

**Keywords:** Developmental biology, Gastroenterology

## Abstract

Parenteral nutrition-associated cholestasis (PNAC) significantly limits the safety of intravenous parenteral nutrition (PN). Critically ill infants are highly vulnerable to PNAC-related morbidity and mortality, however the impact of hepatic immaturity on PNAC is poorly understood. We examined developmental differences between fetal/infant and adult livers, and used human induced pluripotent stem cell-derived hepatocyte-like cells (iHLC) to gain insights into the contribution of development to altered sterol metabolism and PNAC. We used RNA-sequencing and computational techniques to compare gene expression patterns in human fetal/infant livers, adult liver, and iHLC. We identified distinct gene expression profiles between the human feta/infant livers compared to adult liver, and close resemblance of iHLC to human developing livers. Compared to adult, both developing livers and iHLC had significant downregulation of xenobiotic, bile acid, and fatty acid metabolism; and lower expression of the sterol metabolizing gene *ABCG8.* When challenged with stigmasterol, a plant sterol found in intravenous soy lipids, lipid accumulation was significantly higher in iHLC compared to adult-derived HepG2 cells. Our findings provide insights into altered bile acid and lipid metabolizing processes in the immature human liver, and support the use of iHLC as a relevant model system of developing liver to study lipid metabolism and PNAC.

## Introduction

Parenteral nutrition-associated cholestasis (PNAC) is a potentially life-threatening complication of prolonged intravenous parenteral nutrition (PN) in patients who cannot tolerate enteral feeding. While the risk for PNAC is not equally evident in each patient, evidence suggests that infants, particularly preterm infants, have the highest susceptibility for PNAC and the worst prognosis for severe cholestasis and death^1,2^. The higher susceptibility of infants to PNAC suggests that hepatic dysfunction may be a consequence of their relative hepatic immaturity^3^.

Plant sterols (phytosterols) in parenteral soy-based lipid emulsions contribute to the pathogenesis of PNAC^4,5^. Plant sterols are natural steroid alcohols unique to plants and their cell membranes, similar to cholesterol in mammalian species. Infants and children with PNAC have markedly elevated serum and liver plant sterol levels^4,6–8^; and laboratory studies demonstrate that plant sterols contribute to cholestasis, and promote hepatocyte injury and liver inflammation by antagonizing nuclear receptors critical for hepato-protection^5,9,10^. Mechanisms underlying the accumulation of plant sterols in infants receiving PN are unknown.

Sterol-regulating pathways require key hepatic proteins for the elimination of plant sterols. Excess sterols are directly excreted into the biliary tree or broken down and converted into bile acids for excretion in the bile. Hepatic ABCG5 and ABCG8 proteins are important regulators of plant sterols and cholesterol. They are found on the apical membrane of hepatocytes and heterodimerize to form sterol export pumps that prevent sterol accumulation in the body^11,12^. Mutations in either *ABCG5* or *ABCG8* genes result in toxic accumulation of plant sterols and hypercholesterolemia^13,14^. In addition, genome-wide association studies suggest that *ABCG5* and *ABCG8* genetic variation in the normal population may play a biological role in altered plasma lipid concentrations^15,16^. Thus, proper expression of *ABCG5* and *ABCG8* is required for plant sterol clearance.

Normal mechanisms of eliminating sterols may be impaired in the immature liver and contribute to the development of PNAC. Higher plant sterol levels have been described in infants receiving soy lipids compared to older children with soy lipids^6^. During a short period of soy lipid therapy, we found higher accumulation of plant sterols in very preterm infants compared to more mature infants^17^. These findings suggest that the developmental expression of critical sterol-regulating genes may be of particular significance in the development of PNAC in infants dependent on TPN.

There are little data regarding the expression of sterol-regulating genes, including *ABCG5 and ABCG8,* during normal human development. A developmental inability to regulate xenosterol levels may predispose the immature liver to sterol accumulation and PNAC when challenged with intravenous lipids containing plant sterols. Furthermore, our understanding of hepatic lipid metabolism during development is limited because of difficulties obtaining liver samples in fetal and infant populations for experimental studies. Stem cell technology has enabled researchers to recapitulate hepatogenesis from human stem cells in vitro to allow studies that are difficult to perform in humans. Human induced pluripotent stem cells (iPSC) can be differentiated into a variety of tissue-specific cell types, and iPSC-derived hepatocyte-like cells (iHLC) are a promising system for modeling the developing human liver and hepatic metabolism^18,19^. Reports suggest that iHLC more closely resemble immature hepatocytes than adult hepatocytes^20^, supporting their potential as a model for PNAC in infants. The primary objective of this study was to identify transcriptome differences in sterol metabolizing pathways between fetal/infant and adult livers. The secondary objective was to explore the contribution of development to altered sterol metabolism and PNAC using iHLC.

## Results

### Gene expression differences: developing liver vs iHLC vs adult liver

We focused on human developing liver at gestational ages eligible for neonatal intensive care unit (NICU) care and examined available, non-diseased liver tissue at clinically-relevant gestational ages from the National Institutes of Health (NIH) Neurobiobank. One liver sample each at 20, 22, 25, and 39 weeks gestation were available for this study. Information regarding fetal/infant liver tissue used in this study are shown in Table 1.

**Table 1:** Human fetal/infant liver tissue information.

Sample number	Gestational age (weeks)	Age (days)	Sex	Race	PMI (hours)	History
1	20	0	Female	Black	12	Pregnancy termination
2	22	1	Female	White	6	Prematurity Tetralogy of Fallot
3	25	1	Male	Unknown	15	Prematurity Bilateral germinal matrix hemorrhages and Intraventricular hemorrhage (Grade III), respiratory insufficiency
4	39	0	Female	White	4	Intrauterine fetal demise

PMI, post-mortem interval.

To generate iHLC, we used a previously described protocol^18^ to differentiate iPSC into iHLC (Supplementary Fig. S1). Similar to published reports^18,21^, qRT-PCR validation demonstrated mRNA expression of POU domain class 5 transcription factor 1 *(OCT4)* only in the pluripotent stage (day 0), and increasing amounts of hepatocyte-specific markers in the later stages of the differentiation (Supplementary Fig. S2a). At the completion of the differentiation protocol, there is appropriate intranuclear localization of the nuclear protein HNF4a and cytoplasmic localization of albumin (Supplementary Fig. S2b). These data show that we are able to recreate procedures from published literature to produce highly differentiated hepatocytes (Supplementary Fig. S2a–d).

**Figure 1:**
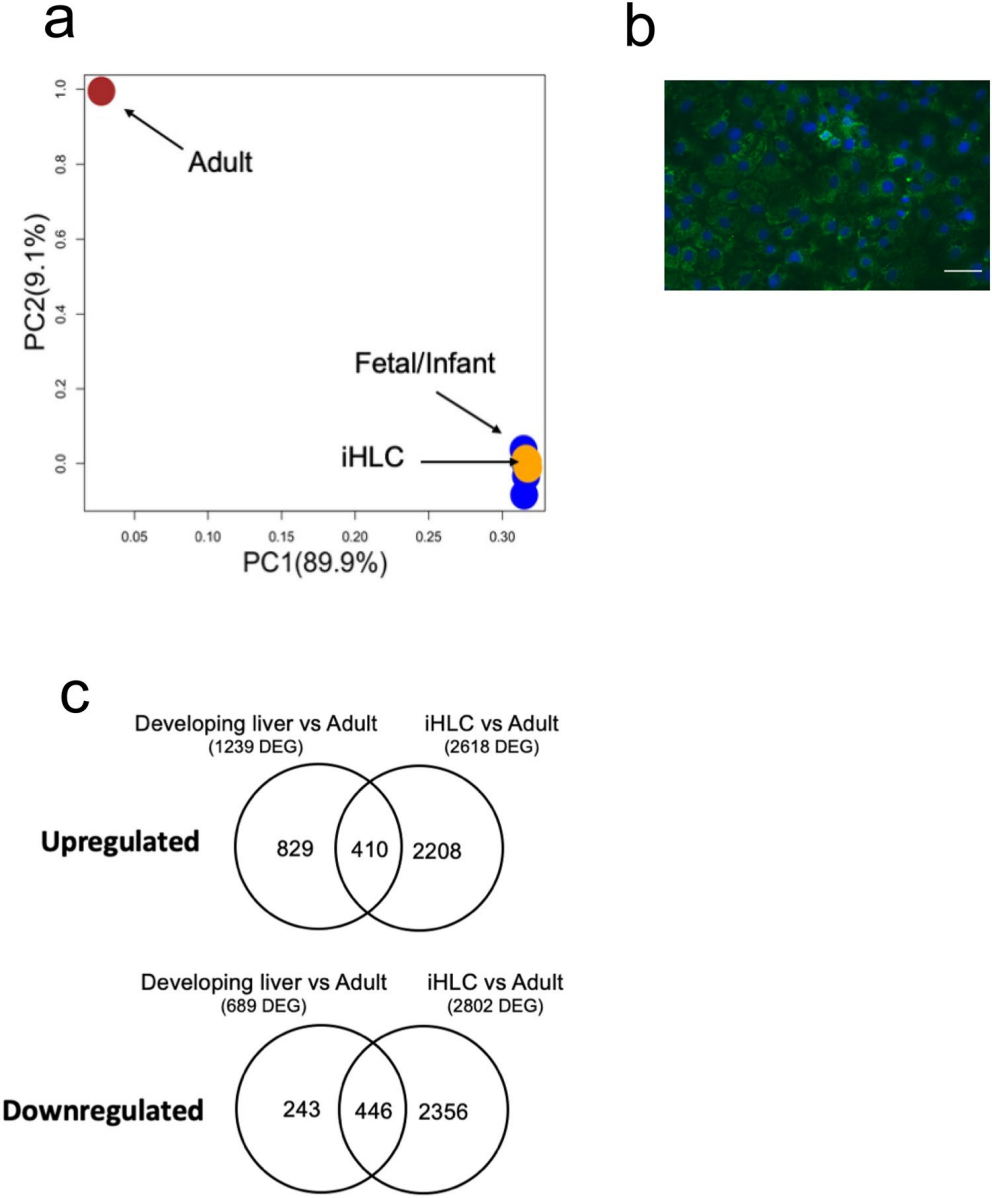
Transcriptome differences in human developing liver, iHLC, and adult liver. (**a**) Principle component analysis (PCA) plot showing variance in gene expression among iHLC from 6 independent differentiations (yellow), 4 human fetal/infant liver samples (blue), and adult liver (red). The top 2 components (PC1 and PC2) account for 99% of the variance among samples, and the variance captured within each coordinate is shown in parentheses. (**b**) Immunocytochemistry of fully differentiated iHLC showing cytoplasmic presence of alpha-fetoprotein (AFP). Scale bar: 25 μm. (**c**) Venn diagram summarizing the overlap of differentially expressed genes (DEG) that are upregulated and downregulated between developing liver vs adult liver compared with DEG in iHLC vs adult liver. DEG were genes with fold change ≥ 4 and adjusted *p*-value < 0.05 in each comparison.

We first determined whether gene transcripts determined by RNA-sequencing (RNA-seq) of human developing livers and iHLC were different from human adult liver (downloaded from the UCSD Human Reference Epigenome Mapping Project and reanalyzed in parallel to minimize differences because of analytic pipelines). For a global comparison, we first performed principal component analysis (PCA) of RNA-seq data from fetal/infant samples, IHLC from six independent differentiations, and adult liver (Fig. 1a). All 4 fetal/infant samples clustered together and were distinct from the adult liver, suggesting that the various stages of fetal/infant liver were substantially more similar to each other than to adult liver. Notably, all 6 iHLC clustered with the fetal/infant samples. Cytoplasmic protein level expression of AFP confirmed the immature status of iHLC (Fig. 1b). These data indicate that iHLC are immature and globally much more similar to human developing liver than adult liver. Given the clustering of fetal/infant samples, normalized gene expression data were averaged for fetal/infant samples to represent the developing liver stage in subsequent comparisons between developing liver and adult liver stages.

**Figure 2:**
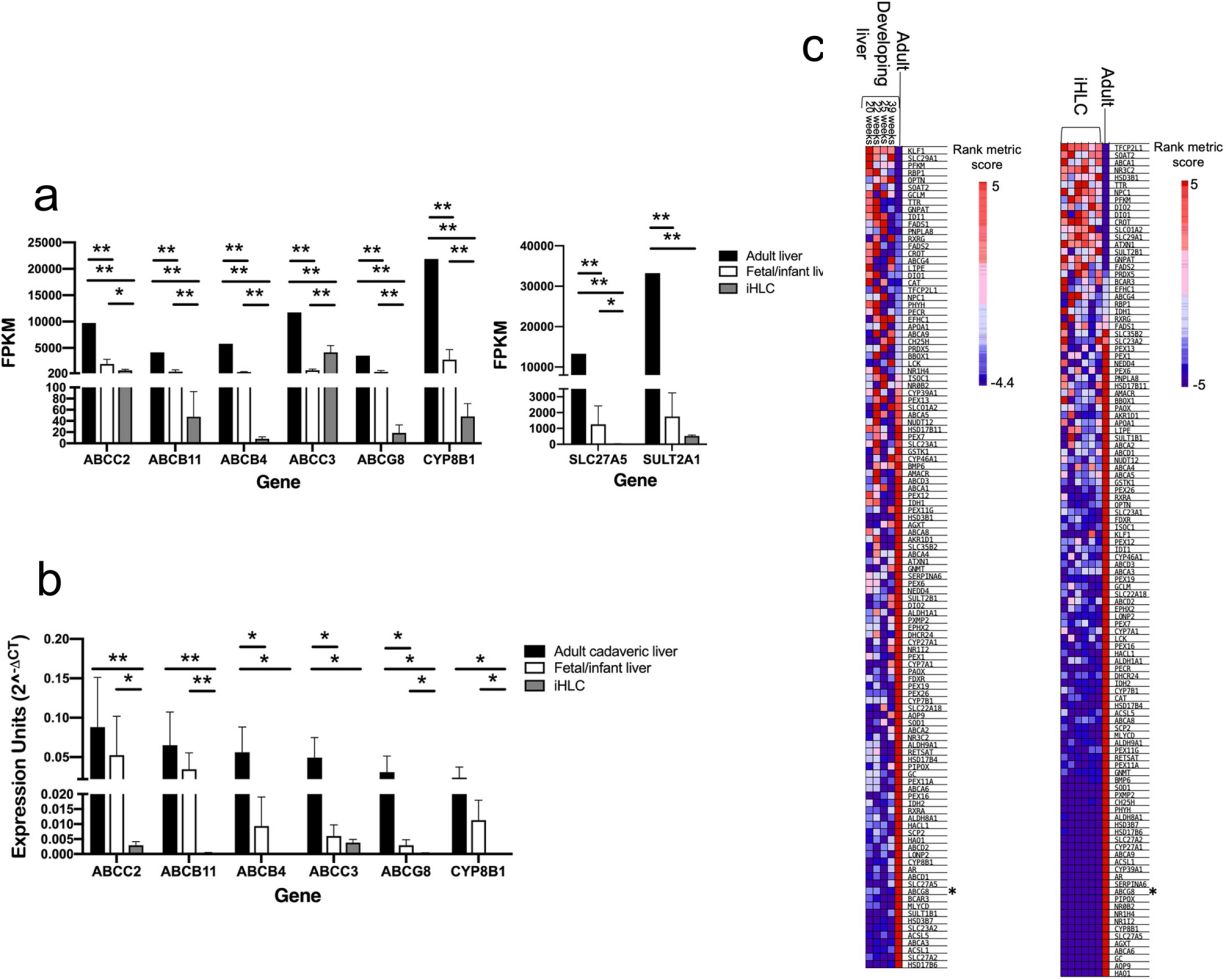
Gene expression differences in bile acid and sterol metabolism in human developing liver, iHLC, and adult liver. (**a**) Bar graphs showing expression of bile acid and sterol metabolizing genes that were among the most differentially expressed in the fetal/infant liver samples (white) and iHLC (gray), compared to adult liver (black) by RNA-sequencing. (**b**) Bar graph showing mRNA levels, by real-time qRT-PCR, of select bile acid transporters and sterol metabolizing genes in the fetal/infant samples (white) and iHLC (gray), compared to six adult cadaveric liver samples (black). Expression units (2^-ΔCT^) was calculated relative to the housekeeping reference gene, β-actin. N = 3 biological replicates each for iHLC. The bar indicates the mean and error bar indicates standard deviation (SD) for FPKM (**a**) and expression units (**b**). *P-value < 0.05, **P-value ≤ 0.01. (**c**) Gene set rank metric heatmaps of the bile acid metabolism gene set for developing liver versus adult liver and iHLC versus adult liver with asterisks indicating *ABCG8*.

To focus on the most substantial transcriptome differences between developmental stages, differentially expressed genes (DEG) were defined as those with at least a fourfold change difference in expression (|Log2FC|≥ 2) and adjusted p-value < 0.05. In developing liver compared to adult liver, a total of 1,928 genes were differentially expressed between stages (1239 (64%) were upregulated and 689 (36%) downregulated in developing liver versus adult liver). All up- and downregulated DEG in developing liver compared to adult are shown in Supplementary Tables S1 and S2, respectively. In iHLC compared to adult liver, 5420 genes were differentially expressed between stages (48% were upregulated and 52% downregulated in iHLC versus adult liver). All up- and downregulated DEG in iHLC compared to adult are shown in Supplementary Tables S3 and S4, respectively. Among the DEG in the comparisons of developing liver versus adult liver and iHLC versus adult liver, 410 genes were similarly upregulated and 446 genes were similarly downregulated in both comparisons (Fig. 1c). Interestingly, several genes involved in bile acid and sterol metabolism were among the most significantly downregulated DEG in developing liver and iHLC compared to adult liver. Specifically, developing liver and iHLC had lower expression of genes involved in canalicular transport of conjugated bilirubin and bile acids (*ABCC2*, *ABCB11*) and phospholipids (*ABCB4)*; alternative export of bile acids (*ABCC3*); elimination of sterols (*ABCG8*); conversion of cholesterol to bile acids (*CYP8B1*); and bile acid conjugation and detoxification (*SLC27A5*, *SULT2A1*) as shown in Fig. 2a. With the exception of *ABCC3*, expression of these genes was significantly lower in iHLC compared to human developing liver. The expression of select genes were validated using real-time qRT-PCR of the fetal/infant samples, iHLC, and six adult cadaveric liver samples, and confirmed significantly lower expression of *ABCB4, ABCC3,* and *ABCG8* and trends in lower expression of *ABCC2, ABCB11*, and *CYP8B1* in fetal/infant compared to adult cadaveric liver tissue; and significantly lower expression of all genes in iHLC compared to adult cadaveric liver tissue (Fig. 2b). These findings suggest decreased metabolism of bile acids and sterols in the immature liver and iHLC compared to adult liver.

### Downregulation of xenobiotic, bile acid, and fatty acid metabolism pathways in developing liver and iHLC

To examine directional enrichment of gene sets differentially expressed between developing liver and adult liver stages, untargeted analysis of the RNA-seq dataset was performed. All gene expression data were analyzed with Gene Set Enrichment Analysis software (GSEA)^22^ using the Molecular Signatures Database Hallmark gene set collection^23^. Seven gene sets were enriched in developing liver and 16 were enriched in the adult liver with FDR q-value < 0.05. Developing liver was associated with upregulation of heme metabolism and processes involved in cell division including E2F targets, G2M checkpoint, mitotic spindle, DNA repair, and spermatogenesis. In contrast, several metabolic pathways including xenobiotic, bile acid, and fatty acid metabolism and peroxisome function were among the most downregulated gene sets associated with developing liver as shown in Table 2. The full list of gene set enrichment between developing and adult liver is shown in Supplementary Table S5. Collectively, this indicates that the immature liver has inhibition of key aspects of hepatic function which may play a role in the metabolism of lipids.

**Table 2:** Most downregulated gene sets in developing liver compared to adult liver stage using the Molecular Signatures Database hallmark gene set in Gene Set Enrichment Analysis (GSEA).

Gene set	Number of mapped genes	Normalized enrichment score (NES)	Nominal *p*-value	FDR q-value
Xenobiotic metabolism	198	− 2.46	0	0
Bile acid metabolism	112	− 2.15	0	0
Coagulation	138	− 2.11	0	0
Fatty acid metabolism	157	− 2.02	0	0
Interferon alpha response	95	− 1.87	0	3.77E-04
Interferon gamma response	198	− 1.89	0	3.14E-04
Adipogenesis	193	− 1.84	0	2.69E-04
Peroxisome	104	− 1.78	0	8.55E-04

FDR, False Discovery.

Gene set enrichments that distinguished developing liver from adult liver showed a similar profile between iHLC and adult liver. Specifically, xenobiotic metabolism (normalized enrichment score (NES): − 2.37, nominal *p*-value < 0.001, FDR *q*-value < 0.001), fatty acid metabolism (NES: − 2.10, nominal *p*-value < 0.001, FDR q-value < 0.001), and bile acid metabolism (NES: − 2.09, nominal *p*-value < 0.001, FDR q-value < 0.001) were also among the most downregulated gene sets in iHLC compared to adult. The list of gene set enrichment between iHLC and adult are shown in Supplementary Table S6. Within the bile acid metabolism gene set, *ABCG8* was among the most downregulated differentially ranked genes in both developing liver and iHLC compared to adult (Fig. 2c). These data show that xenobiotic, bile acid, and fatty acid metabolism pathways are similarly downregulated in developing liver and iHLC compared to adult liver, and support iHLC as an appropriate model system for human immature liver.

### Lipid accumulation in iHLC and HepG2 cells exposed to exogenous stigmasterol

**Figure 3:**
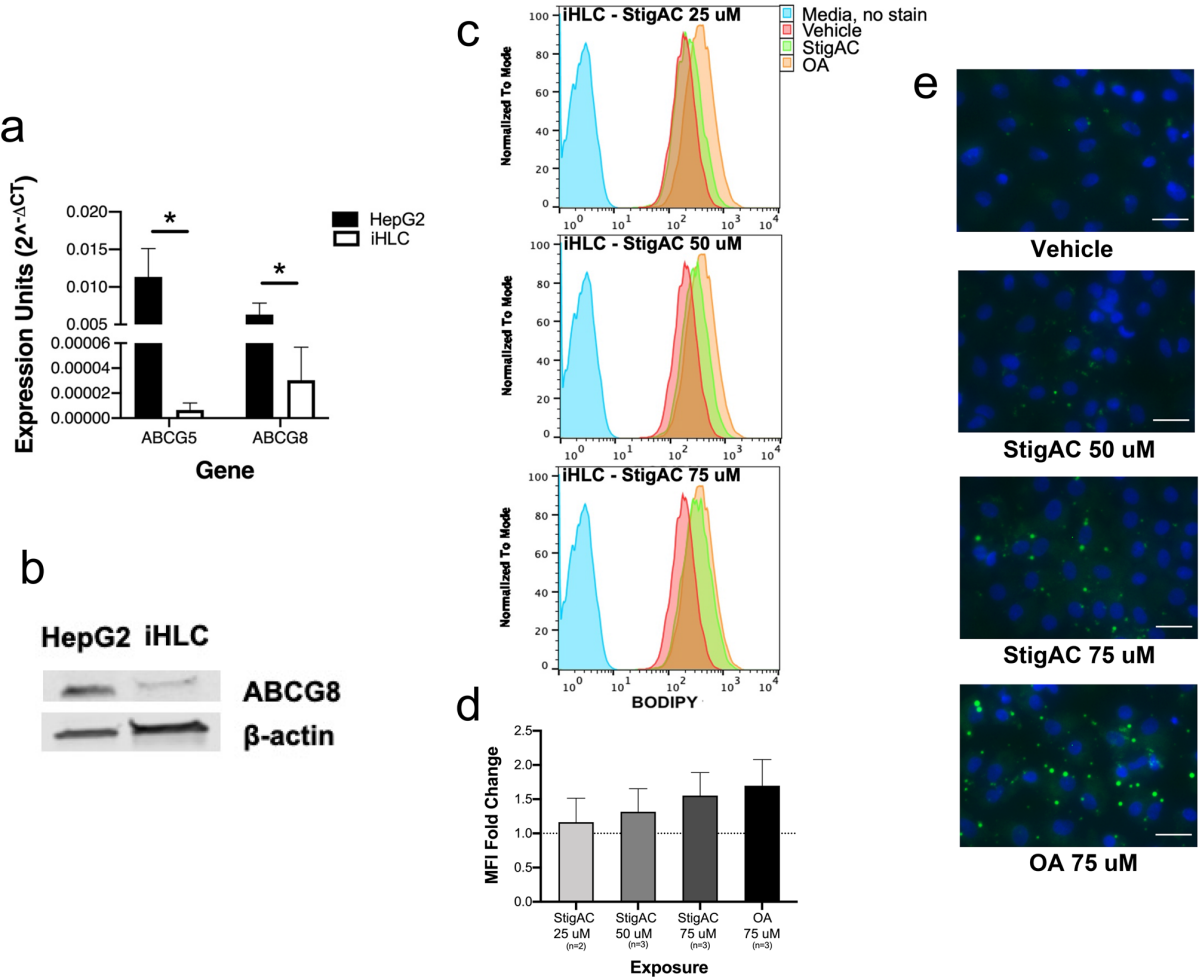
*ABCG5* and *ABCG8* expression and lipid accumulation in iHLC exposed to stigmasterol (StigAC). (**a**) Bar graph showing baseline mRNA levels, by real-time qRT-PCR, of *ABCG5* and *ABCG8* genes in iHLC (white) compared to adult-derived HepG2 hepatocytes (black). Expression units (2^-ΔCT^) was calculated relative to the housekeeping reference gene, β-actin. N = 3 biological replicates each for iHLC and HepG2 cells. Error bar indicates standard deviation (SD). *P-value < 0.05. (**b**) Western blot for baseline ABCG8 protein expression in iHLC and HepG2 cells. (**c**) Representative flow cytometry profile showing the fluorescence of BODIPY in iHLC exposed to increasing concentrations of 25, 50, and 75 μM of StigAC (green), compared to iHLC exposed to vehicle alone (red) and the positive control iHLC exposed to oleic acid (OA) (orange). (**d**) Bar graph showing the BODIPY median fluorescence intensity (MFI) of StigAC-exposed cells relative to the MFI of cells exposed to vehicle alone (dotted line). N = 3 independent experiments in all exposures with the exception of n = 2 for the StigAC 25 μM exposure experiment. Error bar indicates standard deviation (SD). (**e**) Immunocytochemistry showing intracellular lipid droplets in iHLC exposed to 50 μM and 75 μM StigAC compared to iHLC exposed to vehicle alone and the positive control (OA). Scale bar: 25 μm. StigAC, stigmasterol acetate; OA, oleic acid.

Given the similarities between developing liver and iHLC in global gene expression, gene set enrichment, and downregulation of *ABCG8*, the susceptibility to steatosis with immaturity was tested in iHLC exposed to an exogenous plant sterol, stigmasterol (represented as StigAC in figures). Stigmasterol was chosen because of its prominence in soy lipid emulsions and potential to promote PNAC^5,9,10^. Transcriptome results were first confirmed by using qRT-PCR, and Fig. 3a shows the baseline expression of *ABCG5* and *ABCG8* in iHLC compared to commonly used adult-derived HepG2 hepatocytes. Significantly lower baseline expression of *ABCG5* and *ABCG8* were seen in iHLC. Lower ABCG8 protein expression in iHLC compared to HepG2 cells was also confirmed by immunoblot (Fig. 3b and Supplementary Fig. S3).

Next, dose titrations were performed by exposing iHLC to media supplemented with increasing, clinically-relevant concentrations of stigmasterol for 24 hours^17,24^. Intracellular lipid accumulation, as assessed by BODIPY staining of intracellular lipid droplets and flow cytometry, was directly related to concentrations of stigmasterol (Fig. 3c and 3d), and prominent intracellular lipid droplets were seen with exposure to 75 μM stigmasterol by fluorescent microscopy (Fig. 3e).

**Figure 4:**
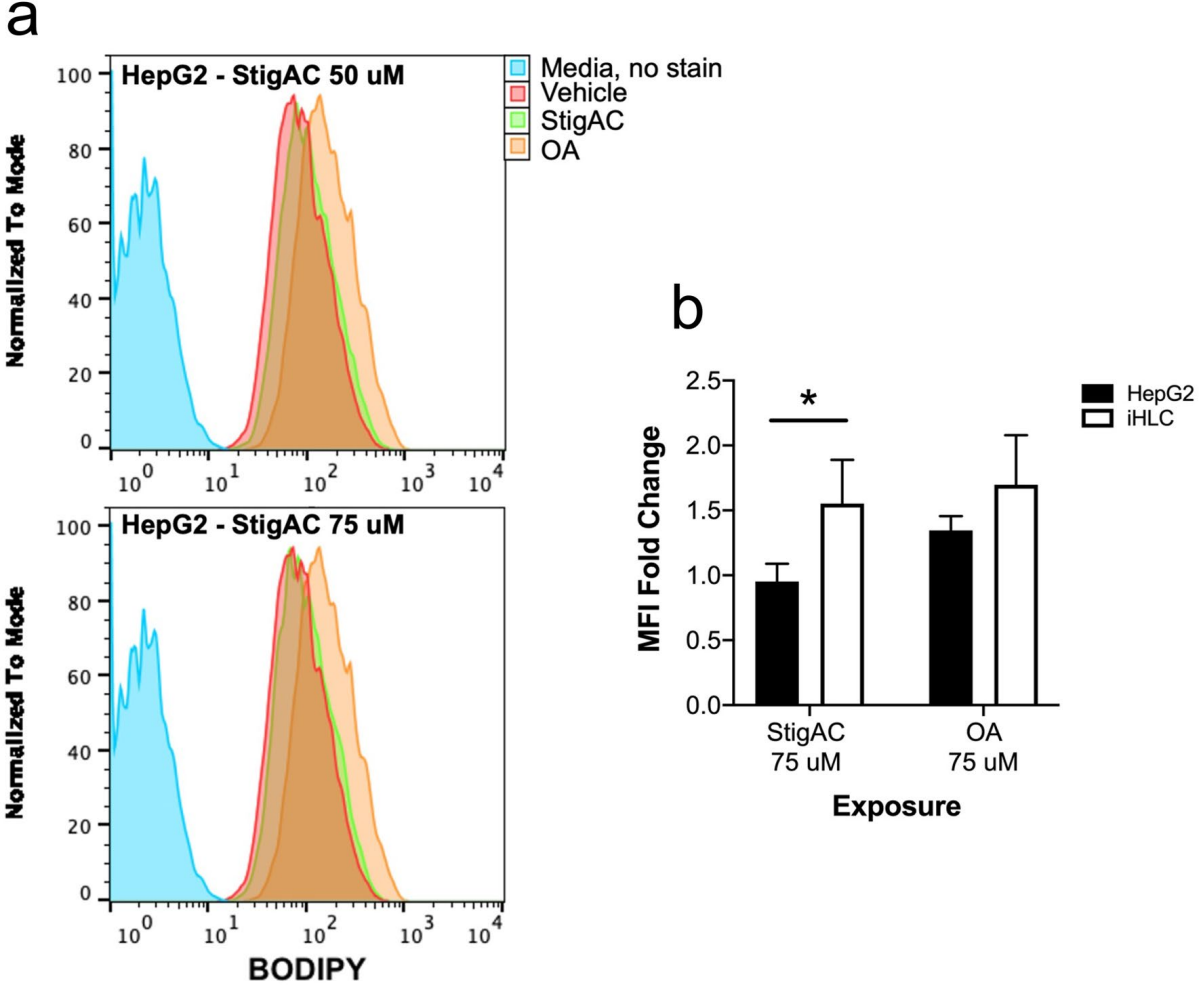
Lipid accumulation in stigmasterol-exposed iHLC and HepG2 cells. (**a**) Representative flow cytometry profile showing the fluorescence of BODIPY in HepG2 cells exposed to 50 μM and 75 μM of StigAC (green), compared to HepG2 cells exposed to vehicle alone (red) and the positive control HepG2 cells exposed to oleic acid (OA) (orange). (**b**) Bar graph showing the BODIPY MFI of iHLC (white) and HepG2 cells (black) exposed to 75 μM of StigAC and oleic acid, relative to the MFI of cells exposed to vehicle alone. N = 3 independent experiments in all exposures. Error bar indicates standard deviation (SD). *P-value < 0.05. StigAC, stigmasterol acetate; OA, oleic acid.

Intracellular lipid accumulation was then compared in iHLC and HepG2 cells exposed to stigmasterol for 24 h. While the intensity of fluorescence of iHLC increased with increasing concentrations of stigmasterol and approached that of the positive control (Fig. 3b), the fluorescence of the stigmasterol-exposed HepG2 cells resembled that of the vehicle (Fig. 4a). Notably, 75 μM stigmasterol induced a greater degree of steatosis in iHLC than in HepG2 cells (Fig. 4b).

## Discussion

In this study, we used RNA-seq to characterize developmental differences in the transcriptome of human fetal/infant livers compared to adult liver. We identified developmentally distinct gene expression profiles with significant downregulation of xenobiotic, bile acid, fatty acid metabolism and peroxisomal function in the immature liver compared to the adult. The immature liver also had significantly lower expression of the sterol metabolizing gene *ABCG8*, involved in the direct excretion of sterols from hepatocytes. In assessing iHLC as a model for the developing liver, we found that gene expression patterns in iHLC closely resemble developing liver, and exhibited a similar inhibition profile for sterol metabolizing genes compared to adult liver. When challenged with exogenous stigmasterol, we found that iHLC accumulate intracellular lipid in a concentration-dependent manner, and that the degree of stigmasterol-induced steatosis was greater in iHLC than in HepG2 cells.

Intravenous nutrition is crucial for survival and neurodevelopment of infants unable to tolerate enteral feeds^25–27^. However, PNAC significantly limits the safety of prolonged TPN in critically ill neonates, with preterm infants having the highest disease-related morbidity and mortality^1–3^. This complication is strongly associated with serum and liver accumulation of potentially hepato-toxic plant sterols in parenteral soy lipid emulsions^4,8^. Importantly, infants receiving soy lipids have two- to five- times higher plant sterol levels compared to older children with soy lipids^6^. During a short period of TPN with soy lipid, we found greater cumulative exposure to plant sterols in very preterm infants compared to more mature infants^17^. Furthermore, cholesterol response to soy lipid is inversely related to gestational age and birth weight^28^. Therefore, differences in the expression of critical sterol-regulating genes between early life and adulthood may be of particular significance in the development of PNAC in infants dependent on TPN.

Data regarding developmental changes in the human liver have been limited, with most prior reports focusing on genes in drug disposition and enterohepatic circulation of bile acids. Many cytochrome P450 enzymes have been characterized by absent to low expression and activity at birth^29^. Screening for hepatic uptake and efflux transporters in human livers from 3 developmental periods, Klaassen et al. found that only *OCTN1, ENT1, ATP8B1, MRP4*, and *ABCA1* were detected in perinatal livers and that the expression of the majority of transporters increased with progressively increasing developmental period^30^. Chen et al. found lower expression of *SLC10A1* and *ABCB4* (encoding the primary bile salt importer NTCP and multidrug resistance protein P-glycoprotein MDR3, respectively) and abnormal intracellular localization of the bile salt export pump BSEP in fetal livers at mid-gestation compared to adult liver^31^. Our study extends these findings by using RNA-seq for unbiased, comprehensive detection of transcribed genes to identify additional genes and pathways that are poorly expressed in the immature liver.

We found that the liver transcriptome and associated functional annotations of fetal/infant samples were notably distinct from adult. This suggests widespread alterations in hepatic molecular signaling between the immature and adult stage. Our data confirm prior findings of lower expression of several drug metabolizing genes including P450 enzymes (*CYP1A1, CYP26A1, CYP4F2, CYP2C18*), phase II enzymes (*UGT2B17*), and canalicular transporters (*ABCC3, ABCB4*) in the developing liver compared to adult liver. We further identified significant downregulation of functions important in lipid homeostasis in the developing liver, specifically fatty acid metabolism, and lower expression of genes involved in sterol metabolism. These results suggest that the developing liver lacks hepato-protective mechanisms against excess lipids that may predispose to steatosis and altered bile acid composition.

Of particular relevance to soy lipids and PNAC, we also found lower expression of an important sterol-regulating gene, *ABCG8*, in the developing liver. Hepatic cholesterol and plant sterol levels are maintained by direct excretion into bile via the obligate ABCG5/G8 heterodimer or conversion to bile acids^12^. Importantly, plant sterols do not get converted to bile acids and rely on ABCG5/G8 for removal from the body. Lower expression of *ABCG8* may predispose to buildup of sterols. In humans with a rare genetic disorder known as sitosterolemia (OMIM 210250), mutations in either *ABCG5* or *ABCG8* genes result in loss of biliary sterol secretion and toxic accumulation of plant sterols in the body^32,33^. Furthermore, *ABCG8* deficient knockout mice with loss of ABCG8 function were shown to have significant liver accumulation of plant sterols after being fed a diet containing plant sterols^34^. Collectively, the literature supports the role of ABCG5/G8 in eliminating plant sterols from the liver, and it is possible that poor expression of *ABCG8* may predispose the immature liver to sterol accumulation and subsequent liver injury when challenged with soy lipids enriched with plant sterols.

Our understanding of sterol accumulation in infants is currently limited by difficulties in obtaining tissue samples and performing invasive studies on asymptomatic infants and children. Animal studies of immature liver susceptibility are limited by species-specific differences in metabolic physiology^35^ and technical difficulties in placing central venous catheters for TPN infusion in fragile, small offspring. Thus, we explored the use of an in vitro model system of human hepatocyte development using iHLC to examine sterol metabolism in the immature liver. iHLC possess many characteristics of hepatocytes isolated from human livers including secretion of albumin, glycogen storage, lipid storage, uptake of low-density lipoproteins, and urea synthesis^18,21^. Despite recapitulating many human hepatocyte functions, iHLC have been reported to have a more immature phenotype resembling the fetal or neonatal liver^19,36^ and suggest that iHLC may be relevant for modeling the immature liver.

We mimicked hepatocyte development using iPSCs and compared the gene expression profiles among iHLC, developing liver, and adult liver. We found a high degree of overlap between global transcriptome profiles of fetal/infant liver samples and iHLC, indicating a large amount of similarity. By functional annotation, several downregulated pathways that distinguished developing liver from adult liver were also apparent in the comparison between iHLC and the adult liver. Notably, the expression of *ABCG8* was among the most downregulated genes in both developing liver and iHLC compared to adult comparisons. Although genes involved in bile acid and sterol metabolism were among the most significantly downregulated DEG in developing liver and iHLC compared to adult liver, the expression of the majority of these genes was significantly lower in iHLC compared to developing liver. Thus, it should be noted that iHLC are an in vitro cell culture system that cannot exactly replicate human immature liver. However, we believe that the global similarities between immature liver and iHLC and their shared gene set differences in xenobiotic, bile acid, and fatty acid metabolism when compared to adult liver support iHLC as a reasonable model for the immature liver.

Due to the similarities between iHLC and developing liver, we used iHLC to study the susceptibility to steatosis that may occur in the immature liver by exposing iHLC to an exogenous plant sterol in setting of decreased *ABCG8*. We found that iHLC accumulate intracellular lipid in a concentration-dependent manner when exposed to stigmasterol in ranges seen in the plasma of infants receiving TPN with soy lipid^17^. In addition, stigmasterol exposure induced significantly more lipid accumulation in iHLC compared to HepG2 cells. Prior studies have shown the feasibility of using iHLC to model human lipid homeostasis and diseases of lipid metabolism^21,37,38^. However, current protocols for the differentiation of iHLC still lack validated methods for obtaining mature hepatocytes that possess adult hepatocyte function^19,20,36^. By capitalizing on the immaturity of iHLC, our study adds to the literature and supports the use of iHLC in understanding the contribution of liver development in altered sterol metabolism.

Although we did not examine bilirubin or bile acid in iHLC exposed to exogenous stigmasterol, data suggest that plant sterols alone may not directly cause cholestasis and liver injury. Rather, laboratory studies indicate that plant sterols promote PNAC by inhibiting normal mechanisms of hepatoprotection when challenged with another insult, such as excess bile acids or inflammation^9,39,40^. In HepG2 cells, plant sterols alone had no significant effect on FXR activation or expression of its target genes. However, when stigmasterol was combined with the potent bile acid ligand chenodeoxycholic acid, stigmasterol suppressed the bile acid-mediated activation of FXR and target genes involved in the adaptive hepatoprotective response to bile acid overload^9^. In a mouse model of PNAC, neither 7 days of intravenous parenteral nutrition containing soy lipid (PN/soy lipid) nor dextran sulphate sodium (DSS)-induced intestinal injury alone were sufficient to induce liver injury as measured by liver enzymes and bilirubin levels; whereas the combination of 7 days PN/soy lipid and intestinal injury promoted hepatic Kupffer cell activation, cytokine production, and PNAC^39^. Further studies are needed to examine whether the higher lipid accumulation in stigmasterol-exposed iPSC compared to stigmasterol-exposed HepG2 leads to increased vulnerability in the face of a second insult.

Obtaining suitable tissue for studies examining organ changes that occur during early human development is complicated by ethical and logistical issues. Although all available, non-diseased liver tissue at clinically-relevant gestational ages from the NIH Neurobiobank were requested for this study, few samples were available and limited our sample size and our ability to assess unique differences between individual weeks of gestation. Due to the clinical relevance that infants are at highest risk for PNAC, we focused on comparisons of fetal/infant liver at clinically relevant gestational ages and adult liver. This limited our ability to examine hepatic transcriptome changes throughout gestation and later in childhood. Future studies are needed to examine changes during these later time periods. Our in vitro studies used HepG2 cells as a surrogate of more mature hepatocytes, and other aspects of the carcinoma cells could have impacted our findings. Only stigmasterol was tested and the role of other plant sterols, or combination of plant sterols, in this model are unknown. Lastly, we examined the response of hepatocytes to exogenous plant sterol exposure, however liver function is complex and the impact of other cell populations such as biliary cells on sterol metabolism require further investigation.

In conclusion, we found significant differences in gene transcripts from human fetal/infant livers compared to adult, and utilized iHLC differentiated from human iPSC to study plant sterol-induced lipid accumulation in the immature liver. Similar gene expression patterns are seen in the human developing liver and iHLC, and are associated with downregulation of functions important in lipid homeostasis and lower expression of key sterol-regulating genes compared to adult liver. Exogenous stigmasterol exposure induces significantly more lipid accumulation in iHLC than HepG2 cells. We speculate that poorly developed mechanisms for sterol metabolism may predispose infants to PNAC due to an inability to adequately eliminate xenosterols in TPN containing soy lipids.

## Methods

Use of de-identified human samples without any linkage to subject information in this study was considered exempt from review and informed consent was waived by the Children’s Wisconsin Institutional Review Board (E377: 441197–1). All experimental protocols were approved by the Medical College of Wisconsin Institutional Biosafety Committee and carried out in accordance with relevant guidelines and regulations. Frozen human liver samples used in this study were obtained from the National Institutes of Health (NIH) Neurobiobank. All available non-diseased, frozen, fetal/infant liver samples from clinically relevant gestational ages were obtained. Adult cadaveric liver samples from male and female individuals without primary liver pathology or history of diabetes or drug use, were obtained for validation studies.

### Tissue preparation and RNA isolation

Tissue samples were prepared on dry ice, rinsed with sterile DNase and RNase free 1 × PBS, then homogenized using a tissue homogenizer in buffer RLT from the RNeasy Mini Kit with on-column DNase treatment (Qiagen; Germantown, MD), and total RNA isolated as per manufacturer instructions.

### Cell Lines and Culture

All culture of human iPSC and differentiation of iHLC were conducted according to protocols approved by the MCW Human Stem Cell Research Oversight Committee (hSCRO approval #17–028). Human iPSC from the SV7 cell line (Dr. Stephen Duncan, Medical University of South Carolina) were cultured and differentiated into hepatocytes using previously published protocols. Briefly, human iPSC were cultured on a defined StemAdhere matrix (Primorigen Biosciences; Madison, WI), fed daily with mTeSR1 as previously described^41^ and supplemented with 20% mouse embryonic fibroblast (MEF)-conditioned media and 20 ng/ml fibroblast growth factor (FGF), and maintained in hypoxic culture conditions at 37 °C, 4% O_2_/5% CO_2_. iPSC were passaged as needed using Accutase (Millipore; Burlington, MA). For differentiations, cells were transferred to Matrigel (Corning; Pittston, PA) coated plates, seeded at 500,000 cells/mL, and directly differentiated into hepatocyte-like cells (iHLC) following the 20-day procedure described by Si-Tayeb^18^ with modifications as previously described^42^.

For RNA-sequencing and iHLC validation experiments, iHLC were harvested in Trizol (Life Technologies; Carlsbad, CA) and total RNA isolated and purified using the same RNeasy Mini Kit with on-column DNase treatment as noted previously. iHLC from 6 independent differentiations were used for RNA-sequencing.

Human hepatoma HepG2 cells were obtained from ATCC Cell Lines (Manassas, VA) and subcultured on a routine basis according to protocol. Cells for exposures were grown as monolayers to 75% confluence in normoxic culture conditions at 37 °C, 95% O2/5% CO2 in modified Eagle’s medium (MEM) supplemented with 10% fetal bovine serum (Life Technologies; Carlsbad, CA).

### Library preparation and sequencing of human fetal/infant liver and iHLC samples

Libraries and next-generation sequencing of human fetal/infant liver samples were prepared by BGI Americas Corporation (University of California Davis, Sacramento, CA), and iHLC samples were prepared and sequenced at the Blood Research Institute (Milwaukee, WI) with sequencing details shown in Supplementary Table S7. Briefly, total RNA samples were quality controlled prior to sequencing using an Agilent Technologies 2100 Bioanalyzer (Santa Clara, CA). Data filtering was done to remove adaptors contamination and low-quality reads from raw reads. FastQC (version 0.10.1) was used to validate raw sequence data quality.

### Computational analysis

Filtered reads from human fetal/infant liver samples and iHLC were aligned to annotated RefSeq Human reference genome build Hg19 using STAR alignment tool (version 2.5.3a). Absolute mRNA abundance was quantified with Cufflinks as Fragments Per Kilobase of transcript per Million mapped reads (FPKM), and with FeatureCounts (version 1.6.2) that counts mapped reads for genomic features. Only genes with FPKM > 0 in all stages were used in downstream analyses. The whole pipeline was run in Basepairtech (https://app.basepairtech.com). For comparison analyses, raw RNA-seq gene expression data derived from the UCSD Human Reference Epigenome Mapping Project for adult liver were downloaded and processed through the same pipeline.

Differential expression analysis of RNA-seq data was done using DESeq2 package (version 3.10) in R (version 3.6), and the Benjamini–Hochberg approach to estimate false discovery rate and adjust p-values for multiple testing.

All gene expression data were used as an input for gene set/pathway analysis with Gene Set Enrichment Analysis software (version 4.0.3) (GSEA, http://software.broadinstitute.org/gsea/index.jsp)^22^, using the Molecular Signatures Database hallmark gene set collection generated by computational methodology to include genes from well-defined biological states/processes^23^.

### Real-time quantitative RT-PCR analysis

First-strand cDNA was synthesized using M-MLV reverse transcriptase, RNase out, and random hexamers (Life Technologies; Carlsbad, CA), following manufacturer protocol. Real-time quantitative reverse transcription polymerase chain reaction (qRT-PCR) analyses were performed using primers listed in Supplementary Table S8 obtained from Integrated DNA Technologies (IDT; Coralville, IA), EvaGreen qPCR master mix (Midwest Scientific; Valley Park, MO), and run on a Bio-Rad CFX Connect Real-Time System (Hercules, CA) using a two-step melt curve. The relative threshold cycle was used to assess gene expression relative to the housekeeping reference gene (β-actin) and shown as expression units (2^-ΔCT^), or subsequently normalized to the reference stage and shown as relative fold change (2^-ΔΔCT^) as indicated in the figures. For validation of iHLC differentiation procedures, purchased human adult liver total RNA (Life Technologies; Carlsbad, CA) was used for the reference mature adult liver in qRT-PCR comparisons.

### Immunoblotting

Protein lysates were prepared by resuspending cells in RIPA lysis and extraction buffer containing HALT protease inhibitor cocktail (Life Technologies; Carlsbad, CA). Protein concentration of lysates was determined using the Pierce BCA assay (Life Technologies; Carlsbad, CA). 30ug of protein was loaded into a 4–15% Mini-PROTEAN TGX Stain-Free Gel (Bio-Rad; Hercules, CA), run at 200 V for 30 min, and subsequently transferred to an Immobilon-FL PVDF membrane (MilliporeSigma; Burlington, MA) at 100 V for 1 h at 4 °C. Blots were blocked for 1 h at room temperature using a 1:1 dilution of Odyssey Blocking Buffer (LI-COR; Lincoln, NE) and PBS. Primary antibody was then added to blocking buffer plus 0.1% Tween-20 and incubated at room temperature for 1 h. Membranes were incubated with secondary antibody in the same blocking buffer plus 0.1% Tween-20 for 1 h in a light protected box to detect signal. Blots were visualized with an Odyssey Infrared Scanner (LI-COR; Lincoln, NE). Representative images were converted to grayscale for figures. Antibodies used are listed in Supplementary Table S9.

### Stigmasterol exposure

Stigmasterol acetate (StigAC) (Steraloids Inc; Newport, RI) was prepared as previously described and dissolved in ethanol^40^. For dose titrations, iPSC-derived hepatocytes were treated for 24-h with 0.75% of stigmasterol/ethanol solutions in media to yield final added concentrations of 25 μM, 50 μM, and 75 μM. These concentrations are consistent with stigmasterol levels we found in the plasma of infants who developed cholestasis while receiving prolonged TPN^17^. For lipid accumulation studies, iHLC and HepG2 cells were treated for 24-h with 75 μM stigmasterol in media. Oleic acid (Santa Cruz Biotechnology; Dallas, TX) was prepared and dissolved in ethanol per manufacturer recommendations, and cells were treated for 24-h with 75 μM of oleic acid in media as a positive control.

### Immunocytochemistry

To validate stages of differentiation, cells were fixed using 4% paraformaldehyde in PBS for 20 min, washed with PBS, permeabilized using 0.4% Triton X-100 in PBS for 20 min, and blocked with 3% BSA in PBS for 60 min. Cells were then incubated with primary antibodies diluted in 1% BSA in PBS overnight at 4 °C. For fluorescence-labeled lipid accumulation analysis, Boron-dipyrromethene (BODIPY 493/503) (Life Technologies; Carlsbad, CA) was used to stain intracellular lipid accumulation. Cells were incubated with 2 μM BODIPY 493/503 in PBS for 20 min in the dark at 37 °C. BODIPY-stained cells were then fixed with 4% paraformaldehyde in PBS for 30 min. A 1:5000 dilution of DAPI (4′,6-diamidino-2-phenylindole) in 1% BSA was used to stain the nuclei. Antigens were visualized after using Alexa Flour 488 or 568 conjugated secondary antibodies. Supplementary Table S9 lists antibodies used. Images were acquired using a Nikon Eclipse TE2000-U inverted microscope (Nikon; Tokyo, Japan), and composed using Adobe Photoshop software.

### Flow cytometry

For albumin: cells were dissociated using 1:1 Accutase:trypsin at 37 °C, fixed with 4% paraformaldehyde in PBS for 15 min, permeabilized with 0.1% Triton X-100 in PBS for 15 min, then blocked with 3% BSA in PBS for 30 min. Cells were then incubated in the presence of primary antibody to albumin diluted in 1% BSA in PBS for 1 h. Cells were then washed twice with blocking solution followed by incubation with Alexa Fluor 488 conjugated secondary antibody for 30 min in the dark and then washed twice with block solution. For intracellular lipid: cells were incubated with 2 μM BODIPY 493/503 in PBS for 20 min in the dark at 37 °C, and dissociated with 1:1 Accutase:trypsin at 37 °C. Dissociated cells were washed once with cold PBS, fixed with 0.1% paraformaldehyde in PBS for 15 min, then washed twice with PBS before analysis. Flow cytometry performed using a LSRII flow cytometer (BD Biosciences; Franklin Lakes, NJ) and analyzed with FlowJo software (version 10.5.3; Tree Star Inc.). For comparisons of intracellular lipid accumulation, median fluorescence intensity (MFI) was normalized for the number of gated cells and shown relative to the vehicle.

### Statistical analysis

Statistical analyses were performed using two-tailed unpaired Student’s *t* test using Prism software, version 8.3.1 (Graphpad, La Jolla, CA). Assays were performed in triplicate when possible; and the number of replicates are indicated in the figure legends. In all graphs, bars show means ± SD. P < 0.05 was considered statistically significant.

### Ethics approval

Use of de-identified human samples without any linkage to subject information in this study was considered exempt from review and informed consent was waived by the Children’s Wisconsin Institutional Review Board (E377: 441197–1). All methods were carried out in accordance with relevant guidelines and regulations.

## Supplementary Information

Supplementary Information.

## Data Availability

RNA-sequencing data from this study has been deposited in the NCBI Gene Expression Omibus (GEO), accession #GSE148790.
