# Supplementary material for: Human induced pluripotent stem cell derived hepatocytes provide insights on parenteral nutrition associated cholestasis in the immature liver
Source: Sci Rep. 2021 Jun 11;11:12386. doi: 10.1038/s41598-021-90510-1 (PMC8196029; doi:10.1038/s41598-021-90510-1)
Supplement: Supplementary file 1 — Supplementary Information. [file 41598_2021_90510_MOESM1_ESM.pdf]

Human Induced Pluripotent Stem Cell Derived Hepatocytes Provide Insights on Parental Nutrition Associated Cholestasis in the Immature Liver

*T. Hang Nghiem-Rao, MD[1,2]

Courtney Pfeifer, BS[1]

Michelle Asuncion, BS[1]

Joshua Nord, PhD[2]

Daniel Schill, PhD[2]

Kirthi Pulakanti, MS[3]

Shailendra B. Patel, BM, ChB, DPhil[4]

Lisa A. Cirillo, PhD[2]

Sridhar Rao, MD, PhD[1,2,3]

Institutional Affiliations:

[1]Department of Pediatrics, Medical College of Wisconsin, Milwaukee, WI USA

[2]Department of Cell Biology, Neurobiology, and Anatomy, Medical College of Wisconsin, Milwaukee, WI USA

[3]Blood Research Institute, Versiti, Milwaukee, WI USA

[4]Department of Medicine, Medical College of Wisconsin, Milwaukee, WI, USA

*Corresponding Author:

T. Hang Nghiem-Rao, MD

Medical College of Wisconsin

PO Box 1997

999 N. 92 Street

Milwaukee, WI 53226, USA

Phone: 414-266-6820

Fax: 414-266-6979

Email: hnghiem@mcw.edu

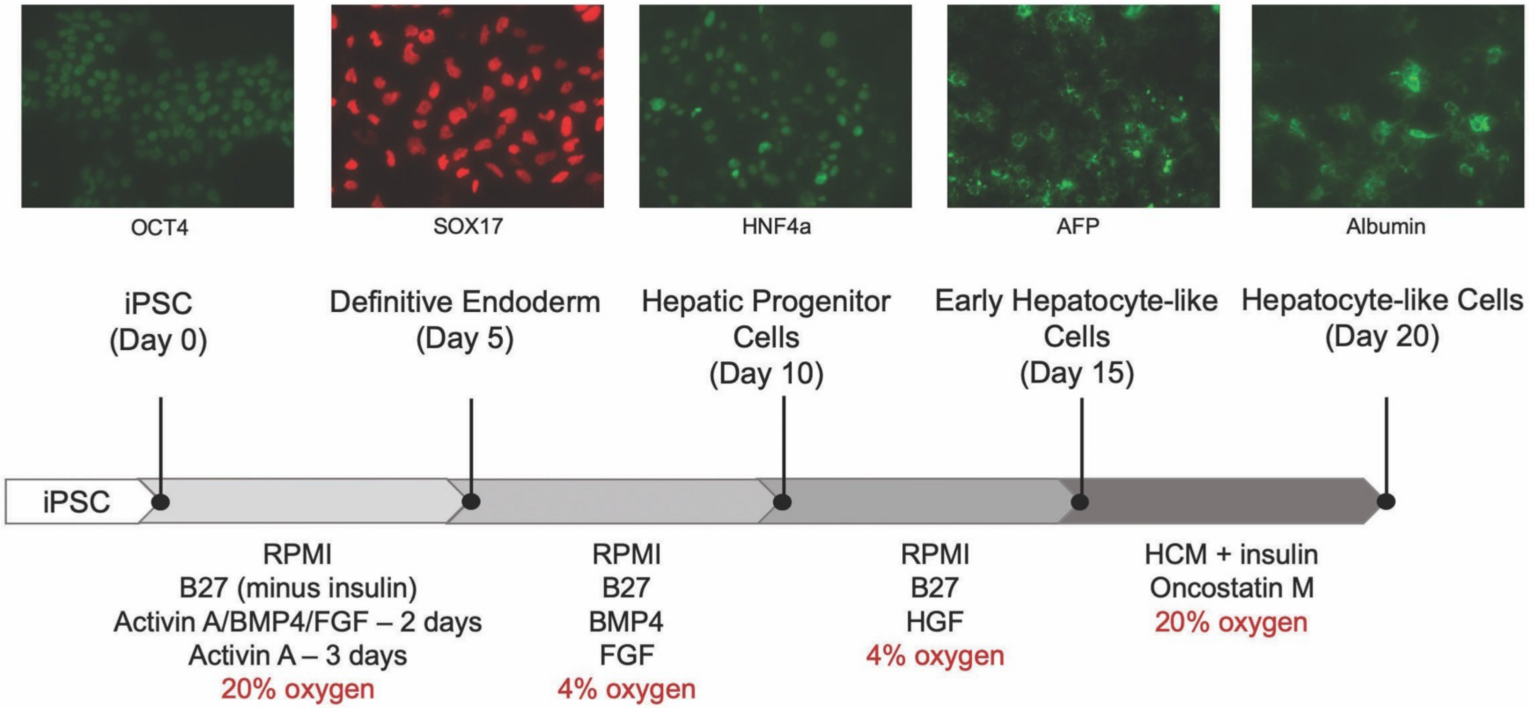

Supplementary Figure S1. Differentiation of human iPSC towards iHLC. Schematic of culture conditions used to differentiate iPSC to generate iHLC in 20 days. Hepatocyte differentiation was monitored by immunocytochemistry at days 0, 5, 10, 15, and 20 using antibodies that recognized proteins associated with each stage. OCT4, POU domain class 5 transcription factor 1; SOX17, sex determining region Y 17; HNF4a, hepatocyte nuclear factor 4a; AFP, alpha-fetoprotein;

B27,vitamin B27 ; BMP4, bone morphogenic protein 4; FGF, fibroblast growth factor 2; HGF, hepatocyte growth factor;

HCM, hepatocyte culture media.

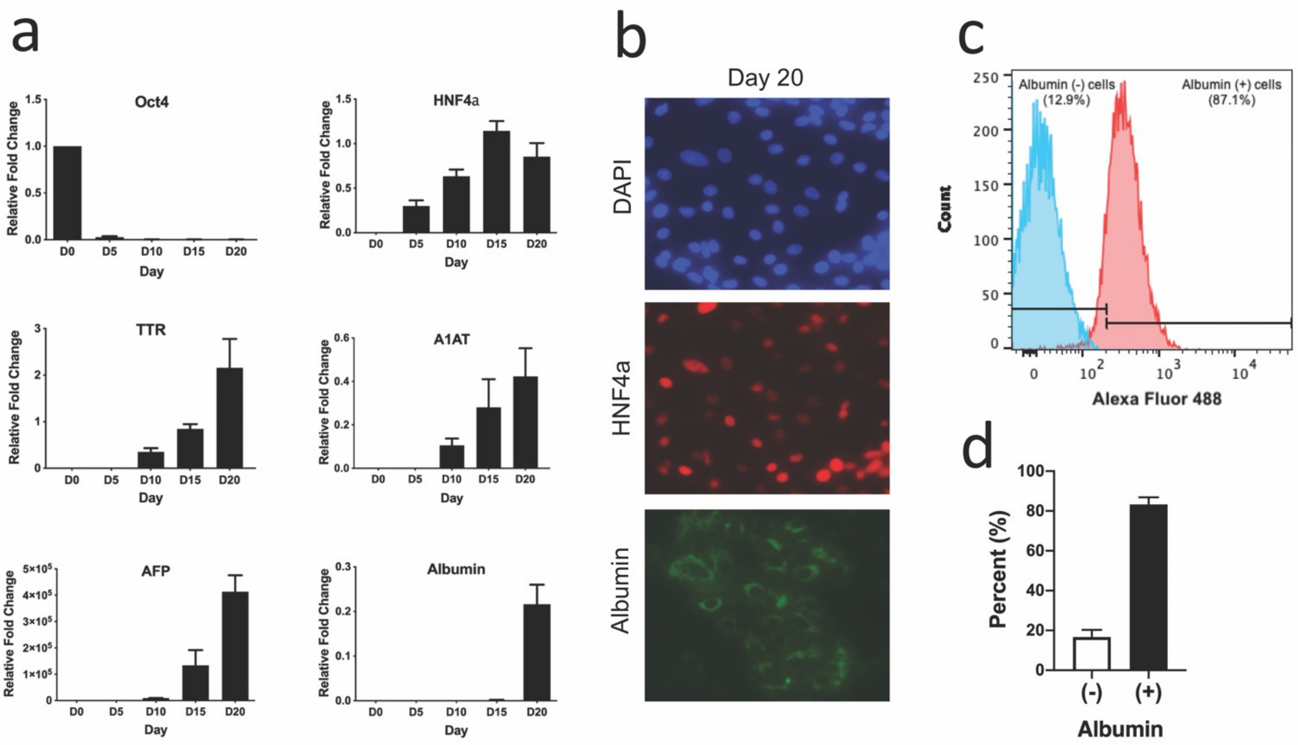

Supplementary Figure S2. Validation of iHLC differentiation. (a) Bar graphs showing mRNA levels, by real-time qRT-PCR, of *OCT4, HNF4a, TTR, A1AT, AFP*, and albumin at each stage of hepatocyte differentiation. Results are from 3 independent differentiations. In each graph, the stages of differentiation are shown on the x-axis and relative expression of each gene ($2^{-\Delta\Delta CT}$) on the y-axis. The pluripotency marker *OCT4* is shown relative to its presence in the pluripotent stage at day 0, and all other markers are shown relative to their expression in mature liver. (b) Immunocytochemistry of hepatocyte-like cells (iHLC) at the completion of the differentiation protocol demonstrate appropriate intranuclear localization of the nuclear protein HNF4a and cytoplasmic localization of albumin. DAPI was used to stain the nuclei (c) Representative flow

cytometry profile showing albumin-positive iHLC. (d) Bar graph showing the average proportion of albumin-positive iHLC in 3 independent differentiations.

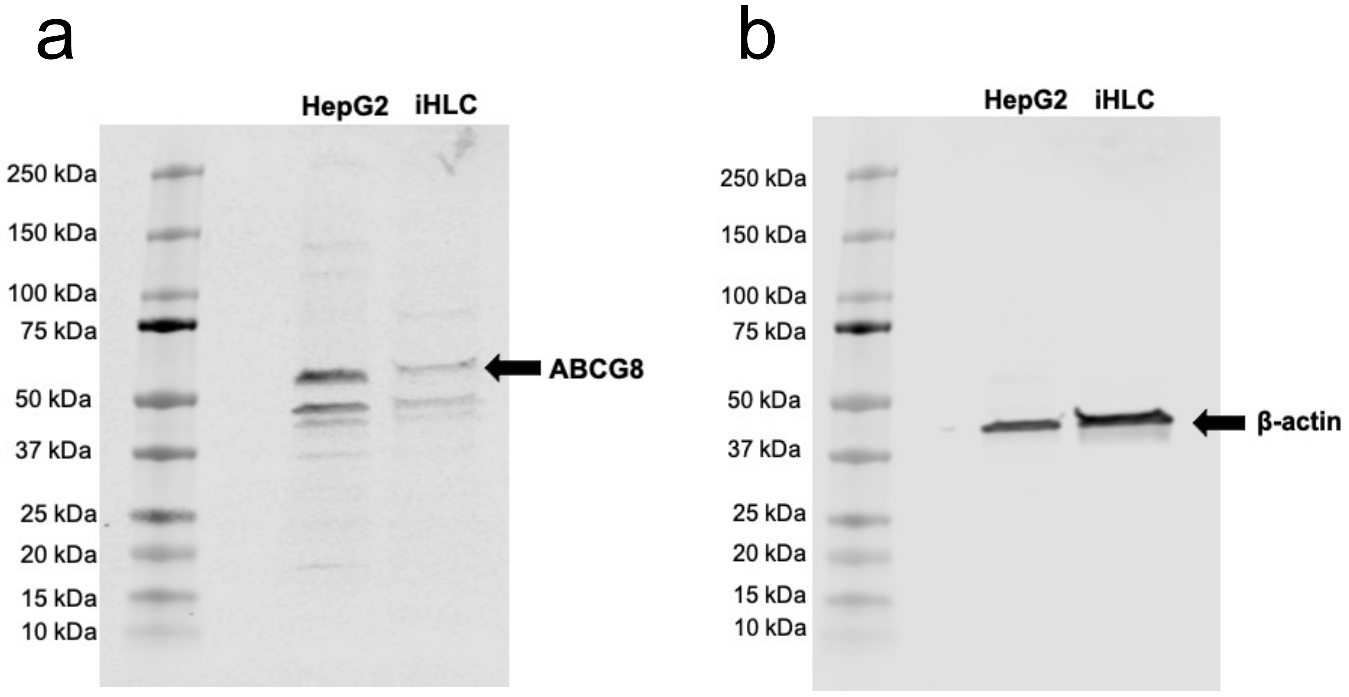

Supplementary Figure S3. Immunoblots of HepG2 and iHLC. a) Full Western blot for ABCG8 in HepG2 and iHLC with arrow indicating the location of ABCG8, as specified by the manufacturer. b) Full Western blot for ß-actin in HepG2 and iHLC with arrow indicating the location of ß-actin, as specified by the manufacturer.

Supplementary Table S1. Upregulated DEG in Developing Liver Compared to Adult Liver Stage

	Gene	Log2FC	P-value	Adjusted p-value
1	HBG2	23.665836	3.65E-29	1.07E-26
2	HBG1	19.06578	1.35E-35	6.32E-33
3	HBE1	14.802461	2.71E-10	1.61E-08
4	XIST	14.766539	2.83E-07	9.22E-06
5	C17orf99	14.499182	8.10E-12	5.98E-10
6	IFIT1B	14.302038	1.96E-10	1.20E-08
7	HBZ	13.932154	2.22E-12	1.79E-10
8	TSIX	13.60927	6.61E-07	0.00001988
9	GYPA	13.607481	1.34E-56	1.71E-53
10	HBM	13.259785	5.03E-96	2.24E-92
11	AHSP	13.239806	1.10E-90	3.25E-87
12	KLF1	13.18275	6.78E-45	4.83E-42
13	DGKK	13.061274	0.00016362	0.00225136
14	HEMGN	12.887077	6.30E-54	6.60E-51
15	EPB42	12.730087	1.28E-59	2.04E-56
16	SLC4A1	12.66168	1.35E-97	8.00E-94
17	ALAS2	12.651583	2.59E-88	6.60E-85
18	GYPB	12.636052	1.35E-15	1.59E-13
19	SPTA1	12.582512	2.69E-49	2.28E-46
20	COL2A1	12.572586	8.84E-07	0.00002586
21	AFP	12.044474	1.44E-06	0.00003894
22	IGLL1	12.000358	3.04E-06	0.00007553
23	SHISA7	11.923078	8.06E-08	2.98E-06
24	RHAG	11.767316	2.09E-51	1.86E-48
25	GKN2	11.708087	2.43E-06	0.00006209
26	NPSR1-AS1	11.663285	5.05E-06	0.00011884
27	CYP19A1	11.454214	8.24E-07	0.00002444
28	HBBP1	11.420709	2.23E-07	7.43E-06
29	ANK1	11.307107	2.91E-61	5.75E-58
30	GATA1	11.102678	4.17E-43	2.75E-40
31	BEST3	11.051931	8.82E-07	0.00002586
32	HMGA2	10.782048	1.31E-09	6.85E-08
33	TMCC2	10.757316	6.12E-63	1.36E-59
34	MTRNR2L10	10.716107	0.00009348	0.00142753
35	HBA1	10.658064	1.40E-135	2.49E-131

36	HBQ1	10.649081	3.78E-24	8.97E-22
37	IGF2BP1	10.640826	2.87E-20	5.32E-18
38	GFI1B	10.595459	1.78E-35	7.93E-33
39	BGLT3	10.557117	1.30E-06	0.00003563
40	ABCB5	10.548576	1.02E-06	0.00002905
41	HBA2	10.45548	1.81E-117	1.61E-113
42	ABCC13	10.450743	4.84E-10	2.74E-08
43	LIN28B	10.364997	6.90E-06	0.00015531
44	DNTT	10.304895	0.00010056	0.00150439
45	FAM178B	10.118184	3.05E-25	7.43E-23
46	CLDN6	9.9430497	0.00007252	0.00115908
47	VPREB1	9.916685	0.00011889	0.00172243
48	SNHG24	9.8954896	0.00007214	0.00115411
49	NKX2-8	9.8631723	0.00025198	0.00327005
50	TUBAL3	9.7589353	0.00001454	0.00029527
51	SPAG6	9.7545957	0.00034237	0.00420695
52	FAM9A	9.7399411	0.00006178	0.00101007
53	ADD2	9.6801614	1.24E-40	7.62E-38
54	EREG	9.6232154	0.00006067	0.00099372
55	ICAM4	9.480658	9.79E-35	4.25E-32
56	KEL	9.406833	2.62E-59	3.58E-56
57	TRIM58	9.3755694	1.85E-42	1.18E-39
58	CRB1	9.1970428	1.98E-09	1.00E-07
59	BTNL10	9.1485639	1.93E-08	8.26E-07
60	LANCL3	9.0586663	5.52E-08	2.13E-06
61	GYPE	9.044297	1.23E-37	6.86E-35
62	PPBP	9.0407385	7.18E-19	1.17E-16
63	PEG10	9.0277196	0.00004823	0.00081868
64	RUNDC3A	9.0223205	4.30E-37	2.32E-34
65	LOC10192883<br>4	9.0078682	0.00009826	0.0014902
66	LOC10012816<br>4	8.9972649	5.22E-09	2.43E-07
67	NFE2	8.9421228	1.89E-60	3.36E-57
68	RHD	8.9254771	2.78E-13	2.51E-11
69	SLC22A16	8.9183465	3.56E-18	5.43E-16
70	HBB	8.9147585	1.05E-12	8.87E-11
71	SLC2A14	8.8849683	1.33E-17	1.90E-15
72	CTSE	8.8824037	8.73E-16	1.04E-13

73	MFSD2B	8.84661	4.68E-27	1.24E-24
74	SLFN14	8.8237891	7.33E-16	8.88E-14
75	LINC00669	8.8026377	0.00010086	0.00150439
76	HEPACAM2	8.7959695	8.78E-10	4.77E-08
77	HIST1H1D	8.6783495	0.00022213	0.0029232
78	CLEC2L	8.6666382	0.00035387	0.00431261
79	GPC3	8.6549236	1.62E-11	1.14E-09
80	THEGL	8.5695735	0.00025129	0.0032658
81	AVP	8.5504981	0.0003826	0.00459969
82	SNCA	8.546576	8.85E-92	3.15E-88
83	FHDC1	8.5422209	1.92E-51	1.80E-48
84	DLK1	8.5168862	1.53E-16	1.96E-14
85	SERPINB10	8.3946252	0.0037527	0.02917767
86	FEZF2	8.3933581	0.00028768	0.00364827
87	TERT	8.360865	0.00115259	0.01123872
88	APELA	8.3364634	0.00617107	0.04280323
89	HTR1F	8.332958	0.00105952	0.01047456
90	PF4	8.3056989	1.69E-16	2.13E-14
91	KRT1	8.2988771	1.82E-23	4.15E-21
92	MYL4	8.2743525	1.38E-59	2.04E-56
93	TRH	8.1616114	0.00456362	0.03409787
94	SPTB	8.1492265	6.12E-36	3.03E-33
95	ELAVL2	8.1394744	0.0021135	0.01832079
96	IBA57-AS1	8.108438	0.0009558	0.00967256
97	STRA6	8.0999957	0.00025756	0.00333031
98	LOC101928002	8.0555123	0.00269913	0.02237341
99	SNCA-AS1	8.0546598	0.00066913	0.007269
100	TDH	8.0515963	4.65E-07	0.00001446
101	TCL1B	8.0509682	0.00700647	0.04718695
102	SPATA21	8.0068965	0.00029293	0.0037017
103	YPEL4	7.9712024	2.67E-47	2.16E-44
104	DUSP21	7.9598943	0.00181742	0.01623643
105	GP9	7.9241828	1.57E-15	1.83E-13
106	TCEAL5	7.9160861	2.38E-06	0.0000609
107	C7orf34	7.8735714	0.0006501	0.00710563
108	LOC101928163	7.7652939	7.11E-06	0.00015846
109	KRT6C	7.7519006	0.00002403	0.00045126

110	DUSP9	7.7488953	1.58E-07	5.52E-06
111	HIST1H2BI	7.7323245	0.00215444	0.01856718
112	IGSF1	7.7317501	1.05E-07	3.81E-06
113	LINC00618	7.73085	0.00331305	0.02631083
114	OR2W3	7.6597996	9.45E-18	1.38E-15
115	ZNF534	7.6461832	0.00141725	0.01332322
116	PHOSPHO1	7.6359797	5.05E-52	5.00E-49
117	TSPO2	7.6280837	1.42E-35	6.49E-33
118	PWAR1	7.5991145	0.00344072	0.02710712
119	IRX1	7.5713778	0.00309577	0.02492998
120	DAND5	7.5625285	0.00342677	0.02703683
121	ANKLE1	7.5482822	1.63E-40	9.65E-38
122	LOC101926996	7.5390523	0.00001053	0.00022243
123	TSPAN32	7.5089314	4.97E-21	9.52E-19
124	CA8	7.492977	2.18E-22	4.52E-20
125	CR1L	7.4607569	2.74E-18	4.28E-16
126	SLC38A5	7.4384573	5.59E-27	1.46E-24
127	FAM138E	7.4193377	0.0026857	0.02230355
128	PNMA3	7.3742612	2.12E-07	7.13E-06
129	LINC00656	7.3720972	8.13E-06	0.00017729
130	TOP1P2	7.344975	0.00318622	0.0254626
131	RNF182	7.2869013	6.82E-09	3.09E-07
132	TUBB1	7.2731773	2.00E-19	3.46E-17
133	GALNT5	7.2505911	5.60E-14	5.45E-12
134	SLC35D3	7.2150444	0.00004984	0.00084267
135	OSBP2	7.2145025	9.82E-36	4.72E-33
136	CHRNA2	7.152629	0.00477189	0.03532783
137	LPAR4	7.1404853	0.00400096	0.03062641
138	TREML2	7.1370396	2.43E-23	5.42E-21
139	CRYBA4	7.0892087	0.00577069	0.04084609
140	PIP5K1B	7.0873393	7.46E-33	2.89E-30
141	SLC2A1	7.0807315	2.90E-29	8.59E-27
142	SNORD114-1	7.0650302	0.00521933	0.03788866
143	LINC00570	7.05716	0.00001171	0.0002444
144	LINC00664	7.0390476	4.53E-09	2.13E-07
145	SCARNA22	7.0081478	0.00612492	0.04255375
146	TRIM71	6.992292	0.00017259	0.00235844
147	FMO1	6.9788341	0.00573054	0.04061793

148	SMIM5	6.9576359	2.50E-33	1.04E-30
149	FAM132B	6.9380998	0.00001402	0.00028527
150	IGF2BP3	6.9318362	6.96E-19	1.15E-16
151	CRISP2	6.9031124	0.00020468	0.00272983
152	AGPAT4-IT1	6.9006973	0.00021218	0.00281931
153	KCNH2	6.8945394	1.49E-29	4.64E-27
154	ACHE	6.8385006	5.45E-17	7.46E-15
155	E2F2	6.8119374	4.83E-45	3.58E-42
156	MYB	6.8023647	3.04E-12	2.42E-10
157	CLDN19	6.7892094	0.0001165	0.00169188
158	HIST1H1B	6.7815766	0.00014119	0.00197792
159	SLC14A1	6.7677425	9.24E-22	1.86E-19
160	TAL1	6.6918247	3.54E-47	2.74E-44
161	LINC00470	6.6914101	0.00009328	0.00142585
162	FCRLA	6.6883604	0.00004794	0.00081464
163	MT1HL1	6.6851023	3.71E-06	0.00009007
164	PAGE2B	6.6845161	0.00019522	0.00262566
165	ITGA2B	6.6547243	4.19E-15	4.55E-13
166	SORCS3	6.6482023	0.0002043	0.00272693
167	KCNN4	6.6281138	1.38E-31	5.12E-29
168	SLC10A4	6.6170242	1.23E-07	4.41E-06
169	SLC2A1-AS1	6.6033321	4.78E-06	0.00011325
170	SLC25A37	6.5679934	1.50E-21	2.93E-19
171	SNAP25-AS1	6.5643806	3.24E-06	0.00007987
172	MEP1A	6.5537365	0.00010263	0.00152405
173	NMU	6.5186252	0.00001379	0.00028187
174	TCL1A	6.5013499	1.66E-06	0.00004359
175	PRSS57	6.4939462	9.03E-08	3.33E-06
176	NWD2	6.4813963	0.0043137	0.0325309
177	SNORD17	6.4809702	0.00203318	0.01776293
178	ICAM5	6.4785572	1.70E-10	1.06E-08
179	LOC100131257	6.4609032	8.79E-08	3.25E-06
180	TREML1	6.4368013	2.33E-08	9.84E-07
181	GAD1	6.3874793	7.72E-06	0.00016946
182	FAM19A4	6.3692539	0.00001046	0.00022112
183	SLC16A12	6.3643779	0.00001245	0.00025891
184	NME8	6.362828	0.00452381	0.0338486
185	DRP2	6.3591351	0.00024101	0.00314832

186	ISX	6.3378683	9.08E-17	1.20E-14
187	RHCE	6.3366716	1.56E-16	1.98E-14
188	BCL11A	6.25078	3.56E-18	5.43E-16
189	RFESD	6.2503695	1.16E-33	4.93E-31
190	GNG4	6.2336589	0.00040993	0.00487566
191	PRND	6.2328941	0.00246971	0.02077148
192	FAM46C	6.2289846	1.42E-29	4.53E-27
193	DDN	6.1659159	0.00174318	0.01570716
194	ASIC4	6.1474456	0.00035688	0.00433301
195	MYT1	6.120954	8.50E-10	4.64E-08
196	S100A3	6.1050165	0.00469475	0.03488732
197	HIST3H2A	6.0790655	1.38E-09	7.17E-08
198	IGDCC4	6.0769326	5.05E-06	0.00011884
199	TSPAN5	6.0670151	1.45E-31	5.26E-29
200	C11orf21	6.0604578	1.82E-13	1.68E-11
201	ZFHX4-AS1	5.9915617	0.00017637	0.00240075
202	RGS6	5.9691742	2.70E-18	4.25E-16
203	SPECC1	5.9138659	3.01E-32	1.14E-29
204	ACSL6	5.9118811	3.62E-36	1.84E-33
205	MRPL23-AS1	5.9045161	2.96E-10	1.75E-08
206	RGCC	5.9042879	2.45E-39	1.41E-36
207	LINC00605	5.8969822	0.00072753	0.00778936
208	SGIP1	5.891595	4.51E-10	2.58E-08
209	UPK3A	5.875828	0.00007392	0.00117834
210	SLC6A11	5.8418043	0.00012516	0.00179134
211	MEG3	5.8295863	3.03E-08	1.25E-06
212	CCDC169	5.8265966	0.00005624	0.00093415
213	PNMT	5.8091113	1.72E-10	1.07E-08
214	ZNF730	5.7904405	0.00093838	0.0095528
215	CYTL1	5.7648381	2.78E-07	9.09E-06
216	ADAMTS16	5.7642155	0.00002268	0.00043148
217	CMTM5	5.751722	3.71E-10	2.14E-08
218	MMP11	5.7248111	1.59E-07	5.55E-06
219	GRAP2	5.7084378	4.30E-26	1.11E-23
220	NTS	5.7074666	1.03E-06	0.00002919
221	RBP2	5.6980596	0.00019343	0.00261106
222	ANKRD18B	5.6909262	0.00008141	0.00127708
223	TMEM158	5.687425	9.61E-07	0.00002774
224	FOXJ1	5.686834	0.00017927	0.00243657

225	DNAJA4	5.6562865	9.73E-31	3.46E-28
226	SPATA9	5.6344679	3.34E-25	8.05E-23
227	ZNF233	5.6222204	2.94E-06	0.00007353
228	TRIM59	5.6184346	2.78E-30	9.51E-28
229	GP1BB	5.6114468	1.23E-14	1.28E-12
230	GAL	5.60141	0.00068387	0.00740204
231	HOTS	5.5979684	3.47E-15	3.84E-13
232	TTC25	5.593328	1.17E-17	1.69E-15
233	HIST1H2BH	5.5626825	5.28E-09	2.45E-07
234	SOX11	5.5609576	0.00281412	0.02319698
235	LINC01133	5.5559749	1.85E-08	7.94E-07
236	MT1B	5.5282456	0.00119214	0.01154848
237	DNAJC6	5.5158569	7.97E-15	8.45E-13
238	FAM129C	5.5155624	2.87E-06	0.00007218
239	HIST1H3G	5.512181	0.00032915	0.00407255
240	CDCA7	5.4926813	6.05E-10	3.38E-08
241	MTRNR2L2	5.4769809	0.00290224	0.02373649
242	GCSAML	5.463358	8.01E-08	2.97E-06
243	SLC6A9	5.4610457	1.71E-18	2.74E-16
244	PAX5	5.4482447	0.00009088	0.00139858
245	SOAT2	5.4219661	6.15E-08	2.34E-06
246	HIST1H2BM	5.4116591	0.00037902	0.00455976
247	PTPRN	5.4085732	5.55E-06	0.0001279
248	EDN3	5.4082134	0.00079678	0.00835984
249	TESC	5.3874224	1.20E-36	6.26E-34
250	SLC22A4	5.377154	7.57E-23	1.62E-20
251	BEND2	5.3662091	0.00418156	0.03177661
252	BEX1	5.3642864	0.0000294	0.00054245
253	BPGM	5.3628718	7.26E-20	1.31E-17
254	SNORA76C	5.3544734	0.00380605	0.0295023
255	LYL1	5.3353275	1.59E-29	4.89E-27
256	DCAF12L1	5.3329388	2.36E-06	0.00006068
257	CCDC169-SOHLH2	5.3185395	7.41E-06	0.00016386
258	RGS10	5.3117032	2.73E-33	1.10E-30
259	MBOAT2	5.28584	2.19E-25	5.42E-23
260	EFHC2	5.2745804	5.08E-06	0.00011935
261	KIF15	5.2715708	2.27E-18	3.60E-16
262	KHDRBS2	5.2677399	0.00197806	0.01738373

263	MS4A3	5.2273612	0.00017169	0.00235155
264	MEG9	5.2168161	1.47E-07	5.16E-06
265	PCSK1N	5.2140431	3.77E-08	1.51E-06
266	SSUH2	5.2044049	6.24E-06	0.0001418
267	DPY19L2P2	5.2030319	1.93E-08	8.26E-07
268	ZNF695	5.1995604	0.00018813	0.002553
269	ALS2CR12	5.1726687	8.61E-11	5.53E-09
270	DLGAP5	5.1697763	7.51E-18	1.12E-15
271	DEPDC1B	5.1592358	2.56E-23	5.63E-21
272	PAX9	5.1530481	0.00014983	0.00208583
273	LINC01215	5.1347703	6.06E-06	0.00013822
274	DPP10-AS1	5.1340092	0.0005747	0.00641936
275	LOC10192749<br>7	5.131726	0.00212451	0.01836379
276	SHISA2	5.1309469	7.56E-06	0.00016655
277	ART5	5.11506	0.00249835	0.02094312
278	HMGB2	5.1148118	2.48E-29	7.50E-27
279	NHLRC4	5.0977882	3.80E-21	7.35E-19
280	ACSL4	5.0942724	2.57E-07	8.43E-06
281	FOXQ1	5.089603	4.19E-07	0.00001317
282	TESPA1	5.0728641	2.71E-11	1.87E-09
283	CHST2	5.0548519	3.21E-14	3.27E-12
284	SYCP2L	5.0392943	0.00116085	0.01130072
285	C15orf27	5.0142326	0.00155047	0.01427971
286	SPIB	4.9956697	0.00065309	0.00712952
287	SERPINI1	4.9707332	1.85E-09	9.40E-08
288	BANK1	4.965254	1.68E-07	5.82E-06
289	IGF2BP2	4.9522827	1.10E-19	1.95E-17
290	CCR6	4.9351254	0.00010786	0.00158846
291	H19	4.93334	8.71E-13	7.45E-11
292	GYPC	4.9106839	5.33E-24	1.25E-21
293	HFM1	4.8977509	0.00141443	0.01331071
294	NUF2	4.8908218	4.01E-28	1.12E-25
295	C8orf88	4.8363466	2.00E-15	2.30E-13
296	SMIM1	4.8323782	8.38E-17	1.13E-14
297	KIF18A	4.8235398	8.70E-17	1.16E-14
298	TMSB15B	4.8211482	0.00273482	0.02260604
299	CENPE	4.8179142	2.03E-15	2.32E-13
300	CENPF	4.812937	1.73E-20	3.24E-18

301	PTTG1	4.7957201	1.14E-29	3.77E-27
302	LOC101927854	4.7887117	0.00103922	0.010337
303	ZNF711	4.7848339	1.05E-08	4.64E-07
304	CCNE1	4.7769697	1.23E-13	1.16E-11
305	XK	4.7648517	2.38E-19	4.04E-17
306	DEFA4	4.7566925	0.00094749	0.00962355
307	KBTBD12	4.7522886	0.00238789	0.02021704
308	MYBL2	4.751676	3.53E-14	3.57E-12
309	PF4V1	4.7477572	1.57E-06	0.00004182
310	STAP1	4.7398714	0.00428424	0.0323891
311	ARL9	4.7391925	0.00001261	0.00026148
312	MED12L	4.7358248	3.53E-07	0.00001124
313	PIM1	4.7282604	3.79E-09	1.82E-07
314	RBM38	4.7148031	4.30E-28	1.18E-25
315	HIST1H4I	4.7107661	0.00067914	0.00735977
316	LOC100129617	4.6912272	0.0002241	0.00294692
317	LEF1	4.6784419	0.00027176	0.00348357
318	KCNIP1	4.670819	0.00080115	0.00839579
319	LOC100505938	4.6705969	0.00062197	0.0068696
320	DNM3	4.6673308	0.00003316	0.00060288
321	CYP2W1	4.6592928	0.00092461	0.00944224
322	USP32P1	4.6502004	0.002157	0.01858024
323	LOC643339	4.6490289	0.00046062	0.00537407
324	GP5	4.6412039	0.00049179	0.00566751
325	LINC00622	4.6093421	0.00480323	0.03550084
326	SPN	4.6066822	1.05E-08	4.64E-07
327	SHCBP1	4.6023399	3.97E-15	4.36E-13
328	CD19	4.6007502	0.0000147	0.00029703
329	SCT	4.5993362	0.00356801	0.02795733
330	NREP	4.5954737	0.00010325	0.0015307
331	MSI1	4.5938492	7.05E-06	0.00015754
332	STYK1	4.5787319	0.00005539	0.00092086
333	KIF6	4.5756242	0.00175032	0.01575556
334	3-Mar	4.5552961	3.14E-10	1.84E-08
335	SLC25A21	4.5545938	6.71E-11	4.36E-09
336	RPL41	4.5479839	4.79E-30	1.61E-27
337	CDKN2D	4.5368402	8.15E-26	2.04E-23

338	LGALS12	4.5354483	9.20E-06	0.00019678
339	ST8SIA6-AS1	4.53139	0.0033716	0.02666858
340	LOC10192727 2	4.5294315	0.00004521	0.00077924
341	DPP10	4.5292506	2.21E-07	7.39E-06
342	NRGN	4.5229875	8.45E-10	4.63E-08
343	ATP1B2	4.5157362	6.58E-16	8.02E-14
344	HMBS	4.5099853	1.12E-23	2.58E-21
345	FAM72A	4.5070001	5.17E-06	0.00012105
346	RAB27B	4.4997007	1.44E-07	5.07E-06
347	COL6A4P2	4.495586	0.00007482	0.00119054
348	HPDL	4.4930516	3.17E-06	0.00007815
349	NECAB1	4.4762372	3.74E-14	3.74E-12
350	HSPA1B	4.4761742	0.00102826	0.01024516
351	HS6ST2	4.4753046	0.00007994	0.00125845
352	OXT	4.4720769	8.84E-07	0.00002586
353	KCNE1L	4.4705666	0.00005968	0.00098378
354	LINC00853	4.4445645	0.00007859	0.00124045
355	CERKL	4.4425845	2.93E-12	2.34E-10
356	GP1BA	4.4338168	7.43E-06	0.00016386
357	GRM7	4.4320417	0.0000856	0.00133256
358	FXYD2	4.4316588	4.07E-09	1.94E-07
359	MGAT3	4.4306204	5.92E-09	2.73E-07
360	ATAD5	4.4284844	7.90E-12	5.86E-10
361	ERP27	4.4229554	0.00020547	0.00273832
362	DMC1	4.4150144	4.68E-07	0.00001452
363	DMTN	4.4129744	6.99E-26	1.78E-23
364	BLM	4.4119105	2.11E-14	2.17E-12
365	STARD4-AS1	4.411672	0.00010442	0.00154553
366	MLC1	4.4063557	1.51E-11	1.07E-09
367	LOC727896	4.4060766	0.00082444	0.00857931
368	MND1	4.4007478	1.88E-12	1.53E-10
369	KIAA0125	4.3998368	0.00006693	0.00107942
370	TAF4B	4.3979744	4.26E-06	0.00010214
371	DIO3	4.3964277	0.00133117	0.01264752
372	CAPN13	4.3936639	0.0002946	0.00372008
373	SLC6A8	4.3897829	9.28E-21	1.76E-18
374	EGF	4.3887901	0.00001538	0.00030951
375	RAB6B	4.386439	9.12E-10	4.92E-08

376	MPO	4.3764029	0.0004049	0.00482552
377	C5orf30	4.3651418	7.45E-17	1.01E-14
378	CCNB2	4.352678	3.95E-17	5.45E-15
379	PLCH1	4.3502187	0.00002143	0.00041164
380	MYO3A	4.3421487	0.0011431	0.01117064
381	CYP3A7	4.3387147	0.0000113	0.00023763
382	FCRL1	4.3246381	0.00089298	0.00918908
383	FANCB	4.3232973	4.02E-08	1.60E-06
384	ROBO2	4.3018513	0.00170345	0.01539593
385	DIAPH3	4.3004408	2.13E-15	2.42E-13
386	LINC00607	4.2898677	2.03E-06	0.00005274
387	NT5M	4.288497	6.24E-14	6.03E-12
388	C2orf48	4.2854078	0.00013746	0.00193337
389	MUSTN1	4.2804237	0.00212455	0.01836379
390	KLF3-AS1	4.2754674	3.65E-13	3.28E-11
391	MKI67	4.2673926	8.60E-14	8.23E-12
392	PRSS35	4.2589145	1.34E-15	1.59E-13
393	HK1	4.2588543	1.19E-11	8.61E-10
394	PVT1	4.2491738	2.52E-08	1.05E-06
395	PCYT1B	4.2269614	8.20E-06	0.00017837
396	CACNA1I	4.2217261	0.00034215	0.00420695
397	HIST1H4B	4.2164094	0.00488191	0.03593323
398	PPAPDC1A	4.2129109	0.00017195	0.00235325
399	OSM	4.2090958	0.00046628	0.00541209
400	HKDC1	4.1956935	4.38E-07	0.00001375
401	MCM10	4.1944025	5.75E-12	4.36E-10
402	CENPA	4.1905113	1.52E-15	1.78E-13
403	FAM3B	4.1895345	0.00083431	0.00867187
404	PRKAR2B	4.1738118	1.75E-16	2.19E-14
405	KIAA1211	4.1684563	1.10E-06	0.00003086
406	SNAP25	4.1558728	0.00003113	0.00057015
407	SEMA7A	4.1551977	2.68E-07	8.76E-06
408	WDHD1	4.1548466	4.85E-12	3.75E-10
409	DQX1	4.1458183	0.00075381	0.00799377
410	C19orf33	4.1352209	3.86E-06	0.00009324
411	KIAA0101	4.1331698	4.05E-20	7.42E-18
412	KIF11	4.1296772	3.67E-12	2.86E-10
413	PLAG1	4.1226963	1.28E-08	5.61E-07
414	PLK4	4.1220708	2.50E-13	2.29E-11

415	CTB-113P19.1	4.1188851	5.99E-07	0.00001821
416	SMOX	4.1173028	0.00004085	0.00071705
417	FIRRE	4.1067522	0.00061596	0.00682036
418	PRSS50	4.1064285	8.08E-07	0.00002403
419	ORC1	4.098154	2.12E-11	1.47E-09
420	FAM72C	4.0923314	7.84E-10	4.31E-08
421	KIF2C	4.0911021	1.31E-16	1.71E-14
422	UCP2	4.086584	1.24E-17	1.78E-15
423	BEX2	4.0842042	3.64E-07	0.00001152
424	RETN	4.0830318	0.00603088	0.0420932
425	LOC101926896	4.0802719	0.00643921	0.04423228
426	OXCT1-AS1	4.0785371	0.00058835	0.0065431
427	HIST1H2AL	4.0760801	0.00268958	0.02232542
428	TROAP	4.0760658	2.35E-19	4.03E-17
429	S100A14	4.0740908	2.83E-07	9.22E-06
430	FAM117A	4.0732933	3.57E-18	5.43E-16
431	GUCY2D	4.0705672	0.00027359	0.00349952
432	RMI2	4.0679831	2.04E-09	1.02E-07
433	RGS16	4.0663165	0.00164336	0.01497491
434	CPXM1	4.062808	2.32E-07	7.66E-06
435	THSD7B	4.0619917	0.00001428	0.00029023
436	LOC100287036	4.0552388	0.00409582	0.03121838
437	VRK1	4.0507724	1.05E-21	2.08E-19
438	CYP3A7-CYP3AP1	4.0373213	0.00042414	0.00502113
439	EGFL6	4.0372315	0.00175912	0.01582679
440	CCDC173	4.0307856	0.0005732	0.00641818
441	YOD1	4.029033	2.11E-07	7.13E-06
442	LOC100505918	4.0290062	0.00298822	0.02425038
443	TREML3P	4.0282435	0.0000798	0.00125737
444	GP6	4.0270482	0.00003155	0.0005768
445	GAL3ST1	4.0261791	1.16E-09	6.15E-08
446	NPR3	4.0113839	5.46E-09	2.52E-07
447	ANKRD9	4.0098037	4.38E-09	2.07E-07
448	MYO18B	4.0077717	3.23E-08	1.32E-06
449	AMN	3.9928085	6.39E-12	4.82E-10
450	COL9A1	3.9924122	9.82E-07	0.00002825

451	BUB1	3.9904106	5.78E-10	3.25E-08
452	PARPBP	3.9881946	3.71E-11	2.51E-09
453	C1orf145	3.9793081	0.00356905	0.02795733
454	NEIL3	3.9730415	3.74E-09	1.80E-07
455	APOBEC2	3.9728518	5.25E-07	0.00001609
456	GPC5	3.9719886	0.00063471	0.00697166
457	RAD21-AS1	3.9707822	0.00062756	0.00691782
458	DMKN	3.9669466	1.07E-06	0.00003021
459	MPP1	3.9659386	3.54E-10	2.06E-08
460	EPCAM	3.9614535	1.12E-10	7.15E-09
461	NCAPH	3.9604807	4.69E-10	2.68E-08
462	TNNI2	3.955828	0.00347357	0.02732963
463	DCDC2	3.9358533	7.46E-07	0.00002229
464	TRPC6	3.9315635	0.00002048	0.0003946
465	AGPAT4	3.9169919	4.00E-15	4.37E-13
466	HIST1H2BG	3.9120467	0.00001079	0.00022773
467	NCAPG	3.9071068	3.79E-12	2.95E-10
468	CELF3	3.8994532	0.00322113	0.0257185
469	SNORA25	3.8976018	0.00138418	0.01308833
470	C21orf58	3.8719485	1.22E-09	6.42E-08
471	GMPR	3.8691407	3.15E-07	0.00001017
472	ACSBG1	3.8683167	5.39E-06	0.00012565
473	LMO2	3.8668541	1.09E-11	7.96E-10
474	IQCA1	3.8636455	9.51E-06	0.00020284
475	COL11A1	3.8591954	0.0010569	0.0104545
476	CPOX	3.8576013	1.51E-16	1.94E-14
477	ST6GALNAC1	3.8574994	0.00001257	0.00026125
478	EDIL3	3.8522188	1.07E-06	0.00003029
479	TTK	3.8513619	7.05E-12	5.25E-10
480	CRNDE	3.8501033	0.00023971	0.00313368
481	KIAA1524	3.8433117	2.79E-10	1.66E-08
482	CDR1	3.8424041	2.86E-06	0.000072
483	TCP11L2	3.8392415	3.08E-06	0.00007638
484	FAM72D	3.8297179	4.51E-07	0.00001409
485	PPOX	3.8292304	2.21E-23	4.98E-21
486	ITGA4	3.8284352	3.47E-08	1.40E-06
487	ARL4A	3.8259868	7.61E-10	4.19E-08
488	LOC101929295	3.8180744	0.00003538	0.0006369

489	TICRR	3.8140622	4.45E-10	2.55E-08
490	TMEM178A	3.8125636	0.0007572	0.00802211
491	SGOL2	3.8081903	5.94E-09	2.73E-07
492	HES6	3.8049728	0.00049891	0.00570527
493	CTD-3080P12.3	3.7982825	0.00508629	0.03717629
494	SNHG10	3.7893862	2.34E-06	0.00006011
495	ANXA13	3.7864154	3.09E-12	2.44E-10
496	PMCH	3.7849706	2.03E-09	1.02E-07
497	HSPA6	3.7827171	0.00509648	0.03720493
498	LINC00989	3.7813257	0.00110227	0.01082511
499	FRAS1	3.779003	0.00008827	0.00136777
500	PPP1R15A	3.7786419	1.45E-06	0.000039
501	CDCA2	3.7751752	1.20E-11	8.64E-10
502	SLC7A1	3.7741281	1.15E-09	6.08E-08
503	S100A4	3.7734666	1.53E-06	0.000041
504	MSS51	3.7671831	0.00104899	0.01039361
505	CELF5	3.7670739	0.00186419	0.01658762
506	FAM65C	3.7656247	9.31E-22	1.86E-19
507	HIST1H1E	3.7648218	0.00095701	0.00967446
508	B3GALT2	3.759336	0.0019418	0.01712424
509	CDK1	3.7553359	2.50E-13	2.29E-11
510	CENPH	3.7517977	1.41E-12	1.17E-10
511	VSNL1	3.7482354	0.00258353	0.02158596
512	DTL	3.7433156	2.84E-09	1.39E-07
513	DEPDC1	3.7392916	3.89E-14	3.87E-12
514	ELOVL4	3.7311371	0.00327936	0.02607998
515	POLQ	3.7290198	3.85E-08	1.53E-06
516	ARID3A	3.7253204	0.00002155	0.00041309
517	HPGDS	3.7197984	0.0004958	0.00569186
518	CDC20	3.718807	1.52E-09	7.80E-08
519	HMGA1	3.7171974	1.70E-06	0.00004477
520	NUSAP1	3.7120868	1.49E-13	1.39E-11
521	PRKCQ	3.7114038	1.99E-08	8.46E-07
522	SLC2A4	3.7103493	2.06E-08	8.77E-07
523	LINC00152	3.7094174	0.00098106	0.00985707
524	EXO1	3.7045776	2.00E-09	1.01E-07
525	IKZF1	3.7027565	2.23E-07	7.43E-06
526	PI4KAP1	3.6948421	2.75E-06	0.00006951

527	B3GALNT1	3.6947317	1.53E-11	1.08E-09
528	IGF2	3.6933917	8.31E-07	0.00002461
529	RAD51	3.6910806	4.51E-08	1.78E-06
530	TNFSF11	3.6878407	0.00108134	0.0106607
531	NEK2	3.6874957	1.95E-15	2.25E-13
532	HJURP	3.6838402	7.97E-18	1.18E-15
533	BTG2	3.6780077	1.81E-10	1.11E-08
534	NPY6R	3.6716355	0.00105404	0.01043199
535	FLJ22184	3.6713347	0.00031103	0.00388688
536	SLC5A9	3.6707581	0.00409914	0.03123026
537	C1orf210	3.6661714	0.00044143	0.00519137
538	DDX26B	3.6658879	1.26E-12	1.05E-10
539	SNORD104	3.6654343	0.00245966	0.02071627
540	PTPN7	3.6584193	1.59E-06	0.00004207
541	INS-IGF2	3.6539912	9.19E-07	0.00002669
542	DCK	3.6500752	2.46E-10	1.48E-08
543	PRR11	3.6497946	2.69E-10	1.61E-08
544	FLJ44511	3.6497784	0.00009972	0.00150091
545	ZNF273	3.648574	8.20E-13	7.12E-11
546	CLSPN	3.6474493	1.06E-06	0.00003004
547	PIP5KL1	3.646837	0.00212465	0.01836379
548	SLC22A15	3.6381932	8.09E-13	7.06E-11
549	KIF14	3.635753	1.77E-07	6.12E-06
550	SLC12A5	3.6327466	0.00004023	0.00070855
551	CCDC28B	3.6271671	1.77E-09	9.01E-08
552	TMEM200B	3.6243232	2.18E-10	1.32E-08
553	CA14	3.6213906	0.00313137	0.02513504
554	MT1F	3.6186035	0.00001459	0.00029561
555	LXN	3.6177166	4.37E-08	1.73E-06
556	CDC7	3.6150594	9.66E-08	3.53E-06
557	KIF12	3.6096956	0.00066687	0.00724889
558	AFF2	3.608664	0.00221283	0.01902438
559	HYMAI	3.6081755	0.00743895	0.04945872
560	MLLT3	3.6041753	5.88E-09	2.71E-07
561	CCDC19	3.6023328	0.00046274	0.00538502
562	SCARNA13	3.6009658	0.0004609	0.00537407
563	FXYD6-FXYD2	3.6003957	1.18E-06	0.00003286
564	KIF23	3.5995875	5.70E-15	6.12E-13
565	FANK1	3.5967332	0.00468076	0.03481242

566	ELOVL7	3.595913	3.41E-07	0.00001091
567	CDT1	3.5814714	3.28E-11	2.24E-09
568	INHA	3.5776065	0.00297653	0.02416653
569	PRR15L	3.5740452	0.00287377	0.0235469
570	TFRC	3.572107	3.25E-11	2.23E-09
571	ZNF788	3.5665974	8.59E-10	4.68E-08
572	TMEM40	3.5636413	8.82E-06	0.00019011
573	FST	3.5609458	0.00534333	0.03850184
574	GBGT1	3.5602181	5.14E-09	2.40E-07
575	ADAM32	3.555553	0.00513165	0.03740032
576	NDC80	3.5546921	3.60E-09	1.74E-07
577	HMMR	3.5385563	6.77E-09	3.08E-07
578	PEG3	3.5363333	0.0000563	0.00093418
579	MRC2	3.5349474	9.31E-08	3.42E-06
580	YBX3	3.5348447	3.59E-07	0.0000114
581	SLC7A5P1	3.5333095	0.00445941	0.03350204
582	HOXA10-HOXA9	3.5332084	0.00046519	0.005403
583	S100A6	3.5287654	3.02E-09	1.47E-07
584	OVGP1	3.5281955	0.00025929	0.00334788
585	ASPM	3.5266127	1.55E-07	5.42E-06
586	SLC1A5	3.5239298	5.96E-07	0.00001814
587	SOSTDC1	3.5220967	0.00006235	0.0010176
588	CENPW	3.5220743	3.32E-15	3.69E-13
589	ABCB10	3.5200999	1.49E-12	1.23E-10
590	FNDC1	3.5153429	5.96E-06	0.00013633
591	CCNA2	3.5071987	1.20E-13	1.13E-11
592	TPX2	3.506384	2.08E-08	8.81E-07
593	ZGRF1	3.5062353	5.21E-11	3.45E-09
594	CCDC34	3.5053812	8.40E-07	0.0000248
595	C9orf40	3.5040236	4.53E-08	1.78E-06
596	CA12	3.5033623	0.00012332	0.00177216
597	MPL	3.502527	0.00104699	0.01038463
598	SNORA33	3.5004497	0.00148863	0.01384803
599	FAM72B	3.5002855	0.0000133	0.00027285
600	HTR2A	3.4953094	0.00184426	0.01643493
601	EMC3-AS1	3.4946937	3.34E-07	0.00001071
602	UBE2T	3.4906746	2.72E-15	3.07E-13
603	ZNF714	3.4904571	1.50E-06	0.00004028

604	CD22	3.4892389	0.00056355	0.00633866
605	KIF18B	3.4844685	4.79E-08	1.87E-06
606	E2F8	3.4833739	8.98E-06	0.00019304
607	PMAIP1	3.4814382	2.09E-07	7.09E-06
608	CDC25A	3.4801456	3.42E-07	0.00001092
609	NAALAD2	3.4780441	0.00009269	0.00142021
610	CKAP2	3.4776024	3.91E-11	2.63E-09
611	PRTN3	3.4721194	0.0055518	0.03965092
612	NCEH1	3.4615523	3.52E-07	0.00001122
613	RGS18	3.4587336	1.05E-07	3.81E-06
614	SPHK1	3.4575916	6.78E-10	3.76E-08
615	STAM-AS1	3.4561842	0.0017842	0.01597863
616	SGOL1	3.450466	2.61E-09	1.29E-07
617	CENPK	3.447734	1.57E-08	6.80E-07
618	BEX4	3.4463754	4.16E-08	1.65E-06
619	ORC6	3.4413737	3.39E-12	2.67E-10
620	BRE-AS1	3.4389946	0.00022132	0.00291629
621	HIF3A	3.4320466	0.000823	0.00857429
622	CEP152	3.4267548	2.17E-14	2.22E-12
623	MT1H	3.4248956	0.00002776	0.00051424
624	EID3	3.4229227	0.00092512	0.00944224
625	XPO7	3.4222182	2.91E-10	1.72E-08
626	NUDT1	3.4217569	1.88E-09	9.56E-08
627	ERMAP	3.4173139	2.67E-13	2.43E-11
628	LOC100288637	3.4138846	1.11E-07	3.99E-06
629	CDKN3	3.4125612	5.20E-14	5.11E-12
630	BCAT1	3.4107907	0.00001753	0.0003469
631	SGOL1-AS1	3.3860524	8.78E-06	0.00018958
632	TNFSF4	3.3854595	0.00229001	0.01959325
633	SKA1	3.3845047	2.69E-10	1.61E-08
634	AZU1	3.3842017	0.00556248	0.03968845
635	LOC100506844	3.3820389	0.00014356	0.00200638
636	MTFR2	3.3815081	9.34E-08	3.42E-06
637	TCTEX1D1	3.378247	0.00008346	0.00130469
638	KIF4A	3.3675422	6.68E-08	2.52E-06
639	CXCL5	3.3663359	0.00148218	0.01379521
640	CHEK1	3.3660562	1.67E-09	8.55E-08

641	PLEK	3.3572232	0.00001156	0.00024186
642	SLFN13	3.3499123	1.31E-08	5.73E-07
643	CCRL2	3.3495947	6.78E-09	3.08E-07
644	RPIA	3.3451838	7.02E-11	4.54E-09
645	DPPA4	3.3449368	0.00078284	0.00823482
646	BEND5	3.3428688	0.00028986	0.00366806
647	BUB1B	3.3415483	6.00E-08	2.29E-06
648	PRC1	3.3413258	2.34E-11	1.62E-09
649	BNIP3L	3.3406661	6.83E-07	0.00002048
650	CLC	3.3330944	0.00003581	0.00064184
651	WDR76	3.3324681	5.76E-08	2.22E-06
652	LOXL4	3.3299916	0.00003249	0.00059209
653	C7orf61	3.3214402	0.00319874	0.02555117
654	ABCC4	3.3154944	3.25E-06	0.00007998
655	EPOR	3.315264	1.40E-12	1.16E-10
656	TCEANC	3.3102599	0.00001022	0.00021652
657	SPC24	3.3097936	6.81E-06	0.0001536
658	NATD1	3.3063357	7.09E-08	2.66E-06
659	TOP2A	3.3062825	8.50E-07	0.00002502
660	NARF	3.2972544	1.03E-10	6.58E-09
661	TTYH1	3.2923518	0.00612554	0.04255375
662	CDCA8	3.2910995	1.74E-10	1.07E-08
663	INHBA-AS1	3.2878765	0.00584866	0.04122538
664	GINS2	3.2842751	7.06E-08	2.65E-06
665	DDX25	3.2821051	0.00228056	0.01954058
666	CMTM2	3.2810985	0.00448464	0.03362067
667	ERCC6L	3.274339	0.00006466	0.00105147
668	H2AFY2	3.2742285	0.00157613	0.01446548
669	GATA5	3.2650475	0.00006748	0.00108739
670	OXTR	3.2638443	0.00310311	0.02497781
671	SNORD47	3.2553769	0.0022273	0.019124
672	ZNF681	3.2511668	9.83E-06	0.0002091
673	ANKRD36BP2	3.2486865	0.00698112	0.04710073
674	VASH2	3.2456876	0.00014356	0.00200638
675	FAM214B	3.2444564	9.79E-14	9.27E-12
676	E2F7	3.2436738	2.66E-06	0.00006741
677	TET1	3.2422402	0.00001764	0.00034854
678	TRNP1	3.2389652	0.00395315	0.03031605
679	MZT1	3.237028	1.41E-14	1.46E-12

680	PNP	3.2315513	6.49E-07	0.00001958
681	SULT1C2	3.2312603	0.00009847	0.0014918
682	CENPU	3.2301159	4.06E-11	2.72E-09
683	ZNF93	3.2293659	0.00002355	0.00044373
684	HOXA9	3.2228138	0.00169924	0.01538132
685	LRRC17	3.2188643	0.00009891	0.00149306
686	ALOX12	3.2145397	0.00001846	0.00036248
687	MOB1B	3.2127493	0.00001613	0.0003227
688	CLUL1	3.2060963	0.00067588	0.00733336
689	SCARNA9	3.2055268	0.00533879	0.03848468
690	PGM2L1	3.2049247	9.62E-09	4.32E-07
691	LOC10050721<br>7	3.2032579	2.45E-06	0.00006251
692	CD7	3.1993325	0.00004562	0.00078401
693	SERPINE2	3.1960044	0.00002968	0.00054647
694	SCOC-AS1	3.1952021	0.00096276	0.00970112
695	UBE2C	3.1907183	1.54E-11	1.08E-09
696	MCM2	3.188693	1.35E-06	0.00003686
697	PIM2	3.1765007	7.61E-06	0.00016754
698	GPSM2	3.1725866	3.70E-10	2.14E-08
699	STK17B	3.1640088	0.00012927	0.00183991
700	HELLS	3.1618509	4.27E-09	2.03E-07
701	RAD51AP1	3.1583257	3.75E-10	2.16E-08
702	MLF1	3.1573175	0.00007178	0.0011494
703	TBC1D22B	3.1560856	1.72E-10	1.07E-08
704	CKAP2L	3.1560179	2.69E-06	0.00006807
705	DCAF12	3.1536863	2.71E-09	1.33E-07
706	GAB3	3.1531851	5.74E-12	4.36E-10
707	CKS2	3.1515248	7.33E-07	0.00002193
708	EPB41	3.1503642	4.77E-10	2.72E-08
709	TYMS	3.1502296	8.45E-06	0.00018303
710	UCA1	3.1446552	0.00313578	0.02514505
711	ABCA7	3.1441284	3.85E-11	2.60E-09
712	BMP2K	3.1421612	1.32E-07	4.68E-06
713	SULT1C4	3.1402665	5.54E-06	0.00012777
714	CLEC2D	3.1390417	4.18E-07	0.00001316
715	SOX4	3.1373795	3.54E-06	0.0000865
716	C1QTNF4	3.1364995	0.0070983	0.04767456
717	RAD54L	3.1316386	1.50E-09	7.76E-08

718	E2F1	3.1272051	3.34E-09	1.62E-07
719	TRAK2	3.1270694	5.63E-11	3.68E-09
720	ZNF321P	3.1220254	4.60E-06	0.0001094
721	HSD17B14	3.1203203	0.00198689	0.01744405
722	ARRDC2	3.1152429	6.07E-07	0.00001842
723	CD36	3.1135897	7.74E-09	3.50E-07
724	NCMAP	3.1113661	0.00007354	0.00117321
725	CCNB1	3.109159	3.82E-08	1.52E-06
726	ESCO2	3.1090735	0.00012845	0.0018297
727	ENPP5	3.1065239	0.00008324	0.00130235
728	GTSE1	3.1014702	1.19E-06	0.000033
729	SLC2A3	3.0983771	0.00090788	0.00931685
730	SESN3	3.0970058	1.47E-10	9.20E-09
731	LOC10050725 0	3.0937997	0.00430872	0.03250712
732	TYMSOS	3.0937632	0.00016199	0.00223585
733	TMPRSS13	3.0914918	0.00233187	0.01985602
734	PYGO1	3.0902663	3.62E-06	0.00008819
735	SMC4	3.0875621	2.66E-09	1.31E-07
736	BOLA3-AS1	3.0871935	5.48E-06	0.00012744
737	BTK	3.0871377	3.35E-06	0.00008197
738	ETV4	3.0840967	0.00143649	0.01346852
739	XRCC2	3.0831098	0.00039168	0.0046836
740	DNAJC27-AS1	3.0829337	0.00302638	0.02450407
741	ACYP1	3.0801069	6.53E-08	2.47E-06
742	AURKA	3.0788292	8.88E-11	5.69E-09
743	BIRC5	3.0754304	4.48E-09	2.11E-07
744	FOXO4	3.0748171	1.20E-07	4.31E-06
745	ENPP3	3.0658309	0.00127382	0.01222
746	LOC440311	3.0628351	0.00003367	0.00061052
747	ZNF215	3.0574722	0.00268314	0.02229271
748	P2RX1	3.0559413	9.08E-07	0.00002642
749	NSUN7	3.0529421	0.00049992	0.00571311
750	CDH6	3.0514095	0.00230169	0.0196634
751	MYOZ3	3.0479023	0.0041097	0.03129734
752	RUNX2	3.0462912	8.41E-06	0.00018238
753	UBE2S	3.0444909	8.33E-07	0.00002465
754	SOX6	3.0417603	5.52E-11	3.63E-09
755	STRIP2	3.0411405	3.33E-06	0.00008176

756	MAP1LC3B2	3.0395995	0.00740107	0.04931738
757	PRC1-AS1	3.0390508	2.57E-09	1.28E-07
758	CCND2	3.0375946	4.68E-06	0.00011084
759	HIST1H3H	3.0249989	0.00009081	0.00139858
760	ZNF90	3.0249774	0.00145096	0.01358271
761	FBXO43	3.0127666	0.00010149	0.00151215
762	MTHFD2	3.008759	0.00006405	0.00104248
763	ALMS1-IT1	3.007459	0.00084588	0.00877675
764	GPCPD1	3.0072923	0.00005409	0.00090175
765	PARP15	3.006356	0.00005452	0.00090717
766	FECH	3.0051896	1.01E-08	4.50E-07
767	LOC10192726 7	3.0020435	0.00001501	0.00030292
768	FUT1	2.9985305	6.94E-06	0.00015606
769	HMMR-AS1	2.9958958	0.00002445	0.00045881
770	MDFI	2.9899094	0.00004248	0.00074153
771	PKIA	2.9887577	0.00003189	0.00058231
772	CHEK2	2.9862227	9.69E-09	4.32E-07
773	AMPD3	2.9819359	1.32E-06	0.00003591
774	PLK1	2.9789892	2.27E-06	0.00005838
775	KIF20B	2.9769119	1.63E-06	0.000043
776	CCDC11	2.9729319	0.00482012	0.03558139
777	C6orf223	2.9699209	0.00030487	0.00383623
778	C18orf54	2.9688093	0.00016116	0.00222616
779	MACC1	2.9664828	0.00022709	0.00297956
780	GSG2	2.9637342	0.00008887	0.00137321
781	KNTC1	2.9636161	3.08E-08	1.26E-06
782	CDCA3	2.9632439	1.28E-07	4.56E-06
783	FBXO5	2.9618173	5.16E-07	0.00001584
784	LOC10272388 5	2.9594414	2.95E-06	0.00007373
785	LOC401052	2.959372	0.00636266	0.0438418
786	PRG2	2.9592871	0.00128916	0.01232436
787	BCL2L14	2.9584314	0.00421821	0.03201289
788	CASC5	2.9549989	0.00004734	0.0008081
789	PIF1	2.9487785	0.00003544	0.00063745
790	HOPX	2.9471757	0.00202287	0.01768148
791	FAM24B	2.9458479	0.00269316	0.02234474
792	CCDC150	2.9387855	0.00673005	0.04585859

793	FBN2	2.9346536	0.00129674	0.0123732
794	KIAA1586	2.9295814	7.20E-06	0.00016032
795	CCNDBP1	2.92869	1.28E-09	6.70E-08
796	TACC3	2.9259458	1.32E-07	4.68E-06
797	ECT2	2.9238693	1.26E-07	4.50E-06
798	DPY19L2	2.9166293	0.00092591	0.00944224
799	TRIP13	2.9154457	1.97E-07	6.72E-06
800	DYRK3	2.9138374	0.00009895	0.00149306
801	MICALCL	2.9128897	0.00001602	0.00032091
802	FANCD2	2.9128666	1.57E-06	0.00004182
803	STMN1	2.8996177	9.21E-07	0.00002671
804	DNA2	2.899566	1.39E-08	6.05E-07
805	CENPL	2.8958334	2.18E-06	0.00005633
806	LIN9	2.8900104	1.85E-07	6.37E-06
807	HIST1H2AM	2.8892059	0.00204953	0.01787944
808	ZNF154	2.8879307	9.99E-07	0.00002869
809	EHBP1L1	2.8853387	1.54E-09	7.91E-08
810	TFDP1	2.8832105	6.97E-06	0.00015627
811	CIT	2.882211	2.66E-08	1.10E-06
812	FBXL13	2.8768778	0.00197244	0.0173514
813	SMIM10	2.8741106	3.16E-06	0.00007792
814	ARHGAP11B	2.8728966	0.00127604	0.01223469
815	TANGO2	2.8666112	1.09E-12	9.18E-11
816	RPS14P3	2.8659299	0.00156938	0.0144109
817	MCM6	2.8620308	0.00001889	0.0003683
818	FBXO7	2.8606216	1.32E-10	8.35E-09
819	LDHB	2.8603768	0.0000131	0.0002699
820	LRRC7	2.8601181	0.00524922	0.03805471
821	GJC3	2.8518079	0.00336319	0.02661406
822	SEPT5-GP1BB	2.8501204	1.01E-06	0.00002894
823	F2RL3	2.8497933	0.0002203	0.00291204
824	CENPM	2.8480301	5.52E-06	0.00012744
825	CDC37L1-AS1	2.8475862	0.0029087	0.0237675
826	UBAC1	2.8457949	8.17E-11	5.27E-09
827	PTOV1-AS1	2.8449622	2.02E-07	6.86E-06
828	MYLIP	2.8413173	0.00132721	0.01262337
829	AURKB	2.8367629	7.70E-08	2.88E-06
830	ANLN	2.8340032	2.10E-06	0.00005431
831	MYEF2	2.8283379	0.00070707	0.00761605

832	MCTP2	2.8268411	2.91E-06	0.00007302
833	FGFR1OP2	2.8257511	1.19E-07	4.28E-06
834	UHRF1	2.8235602	0.00293094	0.0239145
835	CAPG	2.8216255	9.34E-06	0.00019956
836	ZNF431	2.819072	5.74E-07	0.00001755
837	STAR	2.8178934	0.00113762	0.01112929
838	BTG3	2.8104946	1.15E-09	6.08E-08
839	DLGAP1	2.8087701	0.00367682	0.02863772
840	STIL	2.8054303	5.67E-06	0.00013009
841	IL17RE	2.8043138	0.00077933	0.00822526
842	LINC00665	2.8038855	0.00001629	0.00032526
843	LRRC1	2.803579	0.00015932	0.0022041
844	RBL1	2.8031628	0.00002981	0.00054827
845	C5orf34	2.7978892	0.000066	0.00106906
846	SH3TC2	2.7973385	0.00112906	0.01105768
847	LRRN2	2.7954135	0.00432526	0.03260426
848	TRPC1	2.7921138	0.00167707	0.01521927
849	SALL4	2.7906328	0.00038909	0.00465574
850	CCNF	2.7894647	0.00013214	0.00187177
851	LAT	2.7879225	1.19E-06	0.000033
852	CDC45	2.7834224	4.99E-07	0.00001534
853	SNHG1	2.7820922	3.40E-08	1.38E-06
854	NT5C3A	2.7778675	3.04E-08	1.25E-06
855	PIP4K2A	2.7775453	1.47E-06	0.00003951
856	RACGAP1	2.7743084	1.88E-07	6.46E-06
857	GDF9	2.7690728	0.00005257	0.00088379
858	SKA3	2.7685657	0.00085157	0.00881582
859	RSRP1	2.7670678	0.00078721	0.00827411
860	IL1B	2.7628399	0.00146807	0.01370683
861	CENPQ	2.7562825	0.00004285	0.00074574
862	ATAD2	2.7519159	7.71E-07	0.000023
863	MAD2L1	2.7425956	1.24E-07	4.44E-06
864	RHOF	2.7389316	0.00003587	0.00064184
865	ZNF610	2.7382039	0.00516184	0.03760495
866	EZH2	2.7380115	1.38E-07	4.87E-06
867	DZIP3	2.7374607	0.00009256	0.00141956
868	SLC16A9	2.7359935	1.17E-08	5.16E-07
869	ARHGAP11A	2.7234979	2.42E-06	0.00006178
870	GINS1	2.722298	0.00012014	0.00173765

871	TMC5	2.7183544	0.00012056	0.0017414
872	MDK	2.7175067	0.00328283	0.02609411
873	CSF3R	2.7158044	0.00001461	0.00029567
874	DLEU2	2.7091744	9.01E-06	0.00019359
875	YPEL3	2.7084144	0.00001986	0.00038434
876	ACOT11	2.702768	0.00202112	0.01767489
877	ELFN2	2.7025373	0.00590562	0.04151185
878	CDCA4	2.7014535	1.40E-06	0.00003783
879	BCL2L1	2.7005074	0.00004545	0.0007818
880	MOSPD1	2.6987153	0.00047767	0.00552266
881	WEE1	2.6950875	6.61E-06	0.00014976
882	ATP1B3	2.694934	0.00001534	0.00030902
883	SLC25A39	2.6948199	1.28E-09	6.72E-08
884	CEP135	2.692485	4.60E-06	0.00010945
885	HIST1H2AE	2.6884518	0.00076011	0.00804621
886	SASS6	2.6877788	1.17E-06	0.00003254
887	BSPRY	2.6872942	0.00003671	0.00065619
888	ZNF816-ZNF321P	2.6856751	0.00001974	0.00038252
889	NMNAT3	2.6806277	6.28E-06	0.0001425
890	NTM	2.6799724	0.00404258	0.0308554
891	LOC100131564	2.6799038	0.00010488	0.00155096
892	ZIK1	2.6743473	0.00051619	0.00587643
893	LOC728392	2.6737747	0.00047558	0.00550567
894	STK33	2.6723605	0.00433402	0.03265649
895	POMC	2.6700291	0.00636916	0.04385263
896	MTBP	2.6667305	0.00004492	0.00077506
897	PTK7	2.6650475	0.00003835	0.0006814
898	CCDC8	2.6638198	0.00275329	0.02274815
899	C16orf59	2.6620032	0.00186564	0.01659227
900	APOBEC3C	2.6616048	0.00028283	0.00359439
901	OXCT1	2.6608438	0.00011407	0.00166482
902	GNB3	2.6574789	0.00128373	0.01228859
903	OAT	2.6551695	1.63E-06	0.00004301
904	OGN	2.6527485	0.00294441	0.02398228
905	HIST2H2BE	2.6512936	0.0010475	0.01038463
906	LRRC75A	2.646453	0.00012361	0.00177354
907	H3F3A	2.6460164	4.45E-07	0.00001393

908	H3F3AP4	2.6460164	4.45E-07	0.00001393
909	GSTP1	2.6432839	0.00007838	0.00123834
910	SMC2	2.6393816	0.00005393	0.0008999
911	H1FX	2.6392217	9.13E-10	4.92E-08
912	C2orf82	2.6376878	0.00020696	0.00275614
913	HMGN3-AS1	2.6364746	0.00003116	0.00057015
914	BRIP1	2.6297201	0.00049582	0.00569186
915	ZWINT	2.6288084	2.65E-06	0.00006709
916	CRLF3	2.6245567	8.25E-06	0.00017939
917	APOBEC3A_B	2.6219935	0.00564465	0.04010495
918	CCNE2	2.6195755	5.50E-06	0.00012744
919	ACE2	2.6173438	0.0048282	0.03561149
920	ROBO1	2.6124605	0.00619697	0.04296611
921	MTL5	2.6105246	0.00101249	0.01010499
922	MXI1	2.6087581	0.00013759	0.00193359
923	CDC25C	2.6055541	5.11E-06	0.00011977
924	PKMYT1	2.6042475	1.11E-06	0.00003107
925	AMMECR1	2.5972565	2.59E-06	0.00006589
926	ST6GALNAC4	2.5956381	1.08E-09	5.73E-08
927	RAVER2	2.5928116	0.00097402	0.00979797
928	LDOC1	2.5926607	0.00490968	0.03609042
929	BORA	2.5904974	2.16E-08	9.15E-07
930	DLG3	2.5902594	0.00035206	0.00429928
931	H1F0	2.5888436	0.00025735	0.00333006
932	JUND	2.5870957	0.0001094	0.00160715
933	ZNF850	2.58461	3.42E-07	0.00001092
934	ZNF85	2.5843986	0.00013269	0.00187509
935	MKRN1	2.5833584	8.77E-07	0.00002576
936	GAB1	2.5824704	4.15E-07	0.00001309
937	FBXO30	2.5784405	0.00259188	0.02164556
938	HIST2H2AC	2.5774983	0.00187631	0.01667049
939	HAGHL	2.5769258	0.00466221	0.03473252
940	FERMT3	2.5760283	1.76E-06	0.00004597
941	DCLRE1B	2.5743119	0.00002345	0.00044281
942	GMNN	2.5724119	5.65E-10	3.19E-08
943	RAB2B	2.5698762	0.00063144	0.00693998
944	CHTF18	2.5637858	0.00027156	0.00348351
945	KIF20A	2.5616353	8.03E-06	0.00017613
946	NCAPG2	2.5588653	0.0000188	0.00036741

947	ZNF430	2.5559566	0.00037338	0.00450718
948	MCM7	2.5494566	0.0000933	0.00142585
949	OSBPL3	2.5470193	0.00001459	0.00029561
950	SUV420H2	2.5426618	7.29E-06	0.00016181
951	PTN	2.5421148	3.64E-08	1.47E-06
952	ASF1B	2.5415115	0.0003542	0.00431361
953	UBE2O	2.5351419	9.73E-08	3.55E-06
954	PRDM5	2.5350432	0.00020431	0.00272693
955	ZNF137P	2.5315732	0.00286089	0.02348463
956	BRCA2	2.5313733	0.00424928	0.03218626
957	SUZ12P1	2.5302978	0.00057351	0.00641818
958	CDCA5	2.5274923	8.99E-07	0.00002621
959	APOBEC3B	2.5268422	1.97E-06	0.00005118
960	LUC7L3	2.5256693	0.00098155	0.00985707
961	TMED6	2.5233429	0.00010741	0.0015831
962	R3HDM4	2.5231004	8.48E-07	0.00002498
963	SNHG9	2.5222807	0.00112515	0.01102552
964	UBALD2	2.5202764	6.70E-06	0.0001514
965	DBF4	2.519398	9.65E-09	4.32E-07
966	CNRIP1	2.5188618	0.00379174	0.02941697
967	RSAD2	2.5151103	0.0045667	0.03410659
968	GALNT6	2.5140024	0.00527128	0.03815865
969	MARCKS	2.512648	0.00002058	0.00039614
970	TCF3	2.5115502	5.10E-06	0.00011955
971	MCM4	2.5065037	0.00084896	0.00880353
972	RAB30-AS1	2.5031646	0.00001643	0.00032716
973	ATG14	2.5009575	0.0000945	0.00144178
974	HCN3	2.500561	0.00045901	0.00535909
975	ARL6IP6	2.4976945	0.00001099	0.00023158
976	NCAPD2	2.4955147	0.00017475	0.0023824
977	LRRCC1	2.4947384	0.00040696	0.00484675
978	PINLYP	2.493783	0.00548109	0.03927192
979	DNAJC9	2.4934547	4.09E-09	1.95E-07
980	ARHGAP19	2.4931999	4.95E-07	0.00001527
981	SFR1	2.4846298	8.12E-06	0.00017729
982	POLE2	2.4826555	0.00008562	0.00133256
983	ZNF547	2.4810318	0.00016317	0.00224684
984	UROD	2.4787687	1.94E-07	6.64E-06

985	APOBEC3B-AS1	2.4725918	0.00466578	0.03473816
986	SPIN4	2.4719754	4.11E-06	0.00009892
987	ZNF43	2.4711497	0.00002656	0.00049568
988	RECQL4	2.4698996	0.00080488	0.00842998
989	TNFAIP8	2.4685058	0.00104224	0.01035235
990	PBK	2.4682551	2.60E-09	1.29E-07
991	MELK	2.462146	1.90E-06	0.00004942
992	CMPK2	2.4595785	2.43E-06	0.00006198
993	FAM83D	2.4583816	3.78E-08	1.51E-06
994	KBTBD8	2.456957	0.00034838	0.00426905
995	ATP7A	2.4558258	0.00003781	0.00067326
996	RNF139-AS1	2.4540128	0.00472905	0.03506902
997	PTGER3	2.4537291	0.00116691	0.01134723
998	EXOSC9	2.4427755	4.56E-07	0.00001422
999	CCDC14	2.4398798	0.00001264	0.00026148
1000	SNORA7A	2.4394467	0.00541423	0.0388868
1001	CHST10	2.4383521	0.00035489	0.00431911
1002	CLIC2	2.4357603	0.00499999	0.03665084
1003	SCLT1	2.4350033	4.61E-07	0.00001434
1004	LY9	2.4307416	0.00010054	0.00150439
1005	METTL9	2.4303457	0.00059936	0.00664485
1006	SLC25A36	2.4251484	0.00006042	0.00099051
1007	UBE2H	2.4233151	0.00010423	0.00154401
1008	8-Mar	2.4215697	1.31E-06	0.00003568
1009	JAZF1	2.4213829	0.00001381	0.00028202
1010	MMS22L	2.4196873	0.00058804	0.0065431
1011	SP4	2.4192667	0.00001392	0.00028359
1012	CCDC15	2.4180542	0.0048026	0.03550084
1013	MNS1	2.4176648	0.00006639	0.00107359
1014	GLRX5	2.4172579	0.00008893	0.00137321
1015	ICA1L	2.4131497	0.00030673	0.00385138
1016	SLC4A5	2.4121806	0.00006587	0.0010681
1017	SIAH2	2.4120708	0.00045829	0.00535425
1018	KAAG1	2.4089381	0.00080797	0.00844824
1019	ATP2A3	2.4086965	0.00076383	0.00808084
1020	IPW	2.4067746	0.00178498	0.01597863
1021	FANCI	2.4060691	6.98E-06	0.00015627
1022	TRAIP	2.4040681	0.00015233	0.00211609

1023	KCNE3	2.4037673	0.00006682	0.0010787
1024	ITM2A	2.4028819	0.00215159	0.01855157
1025	SLC25A21-AS1	2.4025509	0.00149753	0.01390825
1026	ADAMTS3	2.4019106	0.00004139	0.00072458
1027	RPS29	2.3994432	2.45E-08	1.03E-06
1028	PRKCQ-AS1	2.3942265	0.00009012	0.00138932
1029	S1PR4	2.3918557	0.00482614	0.03561103
1030	ADORA2B	2.3877528	0.00647276	0.04440292
1031	CAMKMT	2.3850041	3.75E-06	0.00009076
1032	ZFAS1	2.3847827	0.00017108	0.00234494
1033	ARHGAP33	2.3834771	0.00010201	0.00151868
1034	MIAT	2.3831338	0.00070912	0.00762897
1035	CHAF1A	2.3819737	0.00009857	0.0014918
1036	DPF3	2.379853	0.0002149	0.00284911
1037	C17orf53	2.3750301	0.00184871	0.01646634
1038	LDB1	2.3716792	0.00001886	0.00036829
1039	CHAF1B	2.3700237	0.00027064	0.00347668
1040	KAT2B	2.367925	0.00003895	0.0006906
1041	TMPO	2.3670849	0.00001877	0.00036731
1042	RFX3	2.3638917	5.52E-06	0.00012744
1043	MST4	2.3638814	0.00101381	0.01011251
1044	RFXAP	2.3620217	0.00010072	0.00150439
1045	FAM210B	2.3617764	1.94E-07	6.64E-06
1046	PDE6B	2.3615065	0.00110034	0.01081216
1047	SPINT2	2.3605569	4.42E-06	0.00010566
1048	REC8	2.3601338	0.00020005	0.00268414
1049	ANKRD32	2.3555285	0.00002261	0.00043096
1050	LMNB1	2.3546341	0.00141677	0.01332322
1051	ESPL1	2.3536534	0.00035246	0.00430122
1052	RAD54B	2.3525435	9.95E-06	0.00021142
1053	RAB38	2.346343	0.00478226	0.03537522
1054	USP15	2.3436086	0.00001326	0.00027238
1055	ZNF83	2.3412062	0.00120114	0.01162296
1056	TMEM120B	2.3375938	0.00014744	0.00205411
1057	MAPK13	2.333766	0.00033115	0.00409045
1058	CPEB4	2.3337072	0.00013978	0.00196118
1059	ARHGAP15	2.3336469	0.00005322	0.00089059
1060	TERF2IP	2.3329737	0.0004451	0.00523101

1061	CHST11	2.3322337	0.00004342	0.00075208
1062	CTF1	2.3310781	0.00173309	0.01562413
1063	MAP2K3	2.3265761	1.06E-09	5.68E-08
1064	TRIM52-AS1	2.3265445	0.00355445	0.02787973
1065	WNK4	2.3211163	0.00406353	0.0309988
1066	CDC6	2.3161006	0.00046119	0.00537407
1067	TOP1	2.3156974	0.00004171	0.00072873
1068	GALNT7	2.3154047	0.00101091	0.01009494
1069	JARID2	2.3151597	0.00004293	0.00074647
1070	LRMP	2.3142403	0.00284411	0.02336839
1071	ANP32B	2.3141501	2.54E-07	8.33E-06
1072	ZC3H6	2.3127893	0.00005973	0.00098378
1073	SLC45A4	2.3102071	0.00003458	0.00062524
1074	MORC3	2.3097018	0.00011609	0.00168864
1075	ZNF100	2.3075274	0.00027942	0.00356328
1076	MSH2	2.3057134	0.00150047	0.0139218
1077	STK17A	2.3027181	0.00007955	0.00125448
1078	FGFBP3	2.3020699	0.00323709	0.02582972
1079	FAM110A	2.3017788	6.09E-07	0.00001845
1080	LOC374443	2.2962299	0.00167871	0.01522073
1081	SPDL1	2.2958873	0.00055215	0.00623801
1082	CENPI	2.2951887	0.00177592	0.01593731
1083	WHSC1	2.2949796	0.00016263	0.00224119
1084	CCDC23	2.2928128	1.79E-07	6.17E-06
1085	GLIPR2	2.2927195	0.00005803	0.00096032
1086	CENPJ	2.2924595	1.30E-06	0.00003563
1087	KMT2E-AS1	2.29031	0.00151736	0.01403612
1088	TUBB6	2.2896163	4.29E-06	0.00010274
1089	MIS18BP1	2.2892279	0.00008116	0.00127469
1090	NUDT4	2.2837244	0.00316612	0.0253133
1091	DDIAS	2.2792394	0.00014724	0.00205288
1092	RAB3IL1	2.277642	0.00037822	0.00455627
1093	MT1G	2.275133	0.00106539	0.01052091
1094	ZMYND8	2.2731186	4.62E-06	0.00010976
1095	RNF19A	2.2731136	0.00091579	0.0093818
1096	CD24	2.2723467	0.00073233	0.00781957
1097	PRELID2	2.2709545	0.00004674	0.00080014
1098	FBXO34	2.2684013	0.00035968	0.00435951
1099	POSTN	2.2664746	0.00386327	0.02985485

1100	WDR62	2.2663178	0.00021975	0.00290685
1101	OIP5	2.2645254	0.00023866	0.00312223
1102	MAP9	2.2584526	0.0003242	0.00401978
1103	SEC14L5	2.2492588	0.00708239	0.04758564
1104	BRCA1	2.2433229	0.00700715	0.04718695
1105	JAK2	2.2431022	0.00013639	0.00192124
1106	HIC2	2.2424097	0.00308547	0.0248808
1107	HAUS3	2.2391183	7.91E-08	2.94E-06
1108	CDC14A	2.2368185	0.00262407	0.02188366
1109	SSBP2	2.2354092	0.00011477	0.00167355
1110	CCP110	2.235382	0.00012598	0.00179881
1111	PRIM1	2.2321573	0.00174696	0.01573322
1112	SYCE2	2.2317401	0.00272322	0.02254161
1113	C12orf75	2.2296806	0.00008444	0.00131883
1114	AKAP8L	2.2295461	9.55E-06	0.00020345
1115	CLK1	2.2295213	0.0009581	0.00967608
1116	BIN2	2.2276696	0.00007655	0.0012137
1117	DACH1	2.2250188	0.0024506	0.02064974
1118	GNAZ	2.2228961	0.00614156	0.04264837
1119	UBE2B	2.2228195	0.00022131	0.00291629
1120	CTBP2	2.2225604	0.00038456	0.0046139
1121	SH3YL1	2.2219484	0.00184134	0.01641712
1122	MCM8	2.2213085	0.00049383	0.00567712
1123	PCNA	2.2192007	0.00004278	0.00074574
1124	BLVRA	2.2185954	0.00013572	0.00191634
1125	ZNF451	2.2153999	0.00094735	0.00962355
1126	IER5	2.2148081	0.00008789	0.00136309
1127	C20orf196	2.2140138	0.00034218	0.00420695
1128	APLF	2.2127051	0.00065371	0.00713197
1129	CDC27	2.2083994	0.00004039	0.00071
1130	HENMT1	2.2028855	0.00028733	0.00364636
1131	SAMD9	2.1996132	0.00050056	0.00571675
1132	MDM1	2.1940952	0.00078157	0.00823482
1133	ATG4D	2.1932084	3.39E-08	1.38E-06
1134	SPATC1L	2.1929429	0.00286524	0.02350847
1135	ASAP1	2.1913128	0.00001658	0.00032941
1136	TMEM136	2.1894311	0.00126196	0.01214378
1137	ADAMTS6	2.1890354	0.007115	0.04771471
1138	RBX1	2.188689	0.00008181	0.00128222

1139	SERTAD2	2.1886113	0.00068145	0.00738026
1140	RFC3	2.1876716	0.00027958	0.00356328
1141	C1GALT1	2.1834226	3.27E-07	0.00001053
1142	HAUS4	2.1814204	5.28E-06	0.00012329
1143	E2F4	2.1807043	2.60E-08	1.08E-06
1144	SWT1	2.1806154	0.0000247	0.00046203
1145	UROS	2.1774484	1.54E-06	0.00004107
1146	ZDHHC17	2.1766272	0.00015824	0.00219251
1147	FAM104A	2.1736595	1.03E-06	0.00002936
1148	PAK6	2.1723693	0.00155108	0.01427971
1149	PRDX2	2.1674468	0.00064233	0.00703647
1150	ANP32E	2.1665941	0.00001599	0.00032052
1151	BSG	2.1662142	0.00116314	0.01131677
1152	PET100	2.165759	0.00075738	0.00802211
1153	B3GNT5	2.165498	0.00527483	0.03815865
1154	DSCC1	2.1648401	0.00093482	0.00952198
1155	TMEM217	2.1606162	0.00679536	0.04617992
1156	LOC10050742 4	2.1550832	0.00169287	0.01533919
1157	BICC1	2.1390676	0.00031868	0.00397068
1158	PAK1	2.1372925	7.39E-06	0.00016386
1159	GTPBP2	2.1342227	0.00001989	0.00038447
1160	NDN	2.1313225	0.00707879	0.04757941
1161	FAR1	2.1288234	4.81E-06	0.00011368
1162	LOC81691	2.1286253	0.00645095	0.04427877
1163	DCLRE1C	2.1272426	0.00001909	0.00037191
1164	MFAP2	2.1255637	0.00052974	0.00601144
1165	LOC10028701 5	2.1251725	0.00165086	0.01503506
1166	TGFBR3L	2.1228804	0.00140734	0.01326507
1167	STK39	2.1214589	0.00001374	0.00028142
1168	LIG1	2.1168636	0.0004985	0.00570527
1169	C12orf76	2.1165301	0.00513071	0.03740032
1170	FSBP	2.1070105	0.00090916	0.00932461
1171	HAUS1	2.1067707	0.00004538	0.00078146
1172	TET3	2.1053001	0.0033329	0.02643311
1173	RFC4	2.1050959	0.0003719	0.00449538
1174	ABCC5	2.1044565	0.00005816	0.00096156
1175	POC1B	2.1043843	0.0005562	0.00627174

1176	ZNF738	2.0985852	0.00057465	0.00641936
1177	LRRC34	2.0983967	0.00559318	0.03985941
1178	DBF4B	2.0949204	0.00004601	0.00079002
1179	CD83	2.0946857	0.00108889	0.01071737
1180	PPME1	2.0910589	0.0000711	0.00114044
1181	H1FX-AS1	2.0891823	0.00004744	0.0008091
1182	TBPL1	2.0889348	0.00001534	0.00030902
1183	DAPP1	2.0877771	0.00434207	0.03270327
1184	GATA2	2.0835359	0.00688516	0.0466833
1185	FAM89B	2.0812151	7.65E-06	0.00016809
1186	CIR1	2.080642	0.00004281	0.00074574
1187	ICA1	2.0801198	0.00011663	0.00169239
1188	WDR26	2.0793287	0.0000987	0.0014918
1189	UXT-AS1	2.0770951	0.00002889	0.00053364
1190	ARHGAP6	2.0767059	0.00741547	0.04938761
1191	TFDP2	2.0766533	0.00078233	0.00823482
1192	LBR	2.0756476	0.00013617	0.00192015
1193	FAXDC2	2.0731251	0.00003851	0.00068355
1194	C10orf12	2.0686332	0.00053445	0.0060534
1195	TRO	2.0658607	0.00177678	0.01593731
1196	C7orf73	2.0646432	0.00426616	0.03229548
1197	RABGAP1L	2.0631594	1.17E-06	0.00003254
1198	ZNF701	2.0627389	0.00012496	0.0017899
1199	HECTD4	2.062724	0.0002424	0.00316414
1200	INCENP	2.0620683	0.00501615	0.03673902
1201	SSX2IP	2.0614572	0.00002045	0.00039447
1202	MAP4K5	2.061085	0.00029925	0.00377086
1203	ZNF37BP	2.0593966	0.00106368	0.01050986
1204	RPS6KL1	2.0536904	0.0027333	0.022604
1205	ZNF675	2.051014	0.00009563	0.00145281
1206	FKBP1B	2.0509892	0.00159114	0.01457321
1207	PDCD10	2.050453	0.00301611	0.02445437
1208	ISYNA1	2.048319	1.40E-06	0.00003797
1209	SORT1	2.0479802	0.00072332	0.00775825
1210	RRM2	2.0471209	0.00100933	0.01009045
1211	POLR1D	2.0431553	0.00145681	0.01363036
1212	RAD23A	2.0415453	0.00001931	0.00037536
1213	SGMS1	2.0370074	0.00020719	0.00275714
1214	RHNO1	2.0364142	0.00010908	0.00160376

1215	SYNGR1	2.0323761	0.00007691	0.00121828
1216	LUC7L	2.0310403	0.00097698	0.00982223
1217	RYBP	2.0308794	0.00055767	0.00628045
1218	SAMD1	2.0307348	0.00722368	0.04831617
1219	CETN3	2.0291215	0.00019424	0.00261809
1220	SLC7A5	2.0268936	0.00007496	0.0011917
1221	NRSN2	2.0255816	0.00011346	0.00165756
1222	PAN3	2.0220957	0.00001318	0.00027105
1223	HLTF	2.0205774	0.00019553	0.00262753
1224	C2orf88	2.0201494	0.00022144	0.00291629
1225	JPX	2.0179481	0.00054758	0.00619029
1226	CLDN15	2.0141668	0.00045156	0.00529991
1227	SUPT3H	2.012463	0.00720598	0.0482159
1228	EDNRA	2.0109927	0.00005277	0.00088631
1229	RIMKLB	2.0100717	0.00012187	0.00175821
1230	TOPORS-AS1	2.0093429	0.00031108	0.00388688
1231	FAM131A	2.0080632	0.00058474	0.00651108
1232	CDYL	2.0080025	0.00021916	0.00290149
1233	FAM122B	2.0052056	0.00004696	0.00080312
1234	SLC29A1	2.0049074	0.00003078	0.00056498
1235	MINPP1	2.0041648	0.00001968	0.00038179
1236	CD58	2.0038283	0.00003237	0.00059052
1237	MARK3	2.0021793	0.00042945	0.00507393
1238	PCDH18	2.0007152	0.00062917	0.00692787
1239	FAM118A	2.0000037	0.00200802	0.01759488

Differentially expressed genes were genes with at least a 4-fold change in gene expression ($|Log2FC| \geq 2$) and adjusted p-value < 0.05.
DEG, differentially expressed genes.

Supplementary Table S2. Downregulated DEG in Developing Liver Compared to Adult Liver Stage

	Gene	Log2FC	P-value	Adjusted p-value
1	SAA2	-12.678057	1.48E-17	2.08E-15
2	HSD11B1	-11.792171	1.81E-43	1.24E-40
3	MFSD2A	-11.330058	3.32E-54	3.70E-51
4	SAA1	-11.194327	0.00228213	0.01954467
5	HEPACAM	-10.632096	2.11E-16	2.63E-14
6	NNMT	-9.8568296	4.46E-13	3.99E-11
7	CNDP1	-9.6370371	1.48E-22	3.11E-20
8	SAA3P	-9.627496	1.63E-12	1.33E-10
9	HEPN1	-9.582032	2.42E-12	1.94E-10
10	AOX1	-9.3265316	0.00002172	0.00041574
11	FAM169B	-9.2036558	6.27E-11	4.09E-09
12	MYH6	-9.1144259	1.34E-10	8.42E-09
13	LOC101927136	-9.0858185	6.57E-15	7.01E-13
14	LOC400867	-9.0231623	0.00115368	0.01124319
15	FAM83A-AS1	-8.8274219	4.68E-13	4.16E-11
16	CYP2B7P	-8.806984	3.56E-14	3.58E-12
17	SCUBE1	-8.7704385	3.18E-07	0.00001027
18	KCNB1	-8.4780813	3.40E-12	2.67E-10
19	AKR7A3	-8.4728663	0.00025568	0.00331086
20	CYP2C8	-8.4646319	0.0003055	0.00383867
21	FGF21	-8.4572401	2.69E-08	1.11E-06
22	C5orf27	-8.440509	3.16E-23	6.85E-21
23	LOC145837	-8.381211	4.80E-08	1.87E-06
24	SPRNP1	-8.3435558	8.12E-06	0.00017729
25	WISP2	-8.2675539	1.11E-07	3.99E-06
26	LINC01018	-8.1606974	1.21E-14	1.27E-12
27	UGT3A1	-8.0964056	5.50E-22	1.13E-19
28	IGSF9	-7.9850341	8.38E-19	1.36E-16
29	REG1A	-7.953063	1.93E-08	8.26E-07
30	PAPPA2	-7.8708143	1.75E-19	3.06E-17
31	CES1	-7.8639015	1.15E-12	9.67E-11
32	SPRR3	-7.6854186	0.00045459	0.00532147
33	C9	-7.591858	2.10E-07	7.12E-06
34	AVPR1A	-7.5426882	1.46E-17	2.07E-15
35	SLITRK3	-7.5204716	2.42E-10	1.46E-08

36	RPL7	-7.5136686	2.77E-19	4.66E-17
37	CRNN	-7.4891919	0.00118618	0.01150703
38	PRSS1	-7.3749478	0.0000392	0.00069311
39	MYH7	-7.3593092	0.00210978	0.01829779
40	SYT9	-7.3449626	1.82E-13	1.68E-11
41	KCNK3	-7.2226008	6.06E-09	2.77E-07
42	LINC00890	-7.1854592	3.65E-06	0.00008873
43	MB	-7.1805083	0.00011253	0.00164772
44	SRCIN1	-7.1371199	3.67E-08	1.47E-06
45	REG1B	-7.0962694	0.00592053	0.04155108
46	REG3A	-7.0962694	0.00592053	0.04155108
47	C4BPA	-7.0943732	4.69E-08	1.84E-06
48	DGCR5	-7.0934948	3.41E-09	1.65E-07
49	CES5A	-7.0534255	7.01E-06	0.00015685
50	B3GAT1	-7.0366979	4.77E-13	4.23E-11
51	GPR37	-6.8872484	3.45E-16	4.23E-14
52	SPINK1	-6.8424481	4.47E-09	2.11E-07
53	CYP2C18	-6.8345665	1.42E-29	4.53E-27
54	KNDC1	-6.748647	9.31E-18	1.37E-15
55	HPR	-6.730822	3.82E-55	4.54E-52
56	CFHR5	-6.6976808	1.57E-12	1.28E-10
57	ADCY1	-6.6801123	5.48E-20	9.95E-18
58	ZIC1	-6.6375156	0.00006827	0.00109801
59	RPS26	-6.6327034	4.07E-33	1.61E-30
60	MAPK4	-6.6299729	3.23E-09	1.57E-07
61	DNAH5	-6.624534	7.77E-08	2.90E-06
62	ADRA1A	-6.5446038	0.0071868	0.04814184
63	CPA1	-6.5181364	0.0021416	0.0184834
64	FLJ22763	-6.5064116	3.28E-06	0.00008053
65	HSD17B13	-6.4582443	2.21E-17	3.08E-15
66	LRG1	-6.368421	4.01E-19	6.67E-17
67	CHRDL2	-6.3538875	9.06E-06	0.00019434
68	LOC100506281	-6.2573698	0.00537067	0.0386363
69	SLC1A1	-6.2569154	2.36E-07	7.77E-06
70	SELE	-6.2243847	8.88E-06	0.0001912
71	SRD5A2	-6.1832006	4.79E-10	2.72E-08
72	ADH1C	-6.1804736	2.34E-07	7.73E-06
73	SYCE1	-6.1711893	7.72E-08	2.88E-06
74	ADTRP	-6.15806	0.00128954	0.01232436

75	GNAO1	-6.15492	5.33E-12	4.09E-10
76	CXCL13	-6.1284218	0.00009506	0.00144779
77	PITX1	-6.1098171	0.00073084	0.00781361
78	NTN1	-6.0683459	1.06E-16	1.39E-14
79	LOC101929337	-6.0475269	0.00001663	0.00033009
80	SLC22A1	-6.0202234	0.00005981	0.00098423
81	CYP26A1	-6.0057728	0.00028862	0.003655
82	INS	-6.0042295	0.00114419	0.01117515
83	GDA	-5.9428123	2.09E-11	1.46E-09
84	APOA5	-5.942439	5.53E-12	4.22E-10
85	ESR1	-5.8971196	0.0000761	0.00120852
86	ANO3	-5.8898283	0.00014108	0.00197784
87	TRPM8	-5.8833395	1.65E-07	5.73E-06
88	PCP4	-5.7956626	0.00239289	0.02024974
89	ORM1	-5.7707543	1.22E-10	7.75E-09
90	LINC00659	-5.7608985	0.0000232	0.00043898
91	APOF	-5.710877	7.00E-14	6.74E-12
92	C14orf180	-5.687436	9.94E-09	4.42E-07
93	ITIH4-AS1	-5.681917	2.02E-28	5.72E-26
94	LOC729083	-5.6484446	1.38E-09	7.18E-08
95	CYP4F11	-5.6319552	1.48E-07	5.19E-06
96	MTUS2	-5.6272271	1.01E-06	0.00002902
97	CLRN3	-5.6004166	6.22E-13	5.48E-11
98	ATOH8	-5.5863852	9.14E-10	4.92E-08
99	RET	-5.5823576	6.37E-08	2.41E-06
100	LOC100132111	-5.5771733	0.00080805	0.00844824
101	THRSP	-5.541947	0.00020309	0.00271643
102	NAT2	-5.5392505	0.00002299	0.00043541
103	GP2	-5.5235267	0.00201799	0.0176562
104	FBXO16	-5.4862282	0.00222764	0.019124
105	HPGD	-5.4597215	2.33E-09	1.16E-07
106	BCO2	-5.4551968	7.25E-13	6.36E-11
107	F8A2	-5.4432035	0.00008118	0.00127469
108	F8A3	-5.4432035	0.00008118	0.00127469
109	UGT2B17	-5.3279114	1.82E-06	0.00004765
110	HTRA1	-5.2787848	1.61E-19	2.84E-17
111	RPL21P28	-5.2289889	8.47E-13	7.28E-11
112	RPL21	-5.2289889	8.47E-13	7.28E-11
113	CYP4F2	-5.2051106	3.68E-08	1.47E-06

114	KCND3	-5.2028135	0.00003898	0.0006906
115	CHL1	-5.1941579	0.00267575	0.02225206
116	COL8A1	-5.1824699	0.00231641	0.01974324
117	EPHB1	-5.1326974	7.40E-06	0.00016386
118	NOMO3	-5.1049857	3.00E-06	0.00007473
119	MARCO	-5.086933	3.59E-06	0.00008746
120	RPS27	-5.0767315	8.06E-16	9.70E-14
121	CSDC2	-5.0206818	4.51E-14	4.46E-12
122	SLC35C1	-4.9937308	2.21E-30	7.73E-28
123	LY6E	-4.9829188	1.48E-11	1.05E-09
124	HLA-F	-4.8627824	0.00008615	0.00133904
125	AHNAK2	-4.8595515	2.15E-12	1.74E-10
126	ILDR2	-4.8569579	0.00022064	0.00291221
127	TSKU	-4.845715	0.00005322	0.00089059
128	TPRG1-AS1	-4.8386442	2.90E-06	0.00007278
129	UBE2QL1	-4.8237064	1.26E-06	0.0000345
130	PZP	-4.8123632	0.00064033	0.00702035
131	LGALS4	-4.7807312	0.00004758	0.00081074
132	LAMA5-AS1	-4.767155	0.00575875	0.04080162
133	TMPRSS2	-4.7501534	0.00001912	0.00037208
134	CFHR4	-4.7454179	0.00008479	0.00132312
135	C1R	-4.7447509	1.03E-06	0.00002919
136	ASPA	-4.7318571	0.00049304	0.00567712
137	KCNN2	-4.7033975	0.00012999	0.0018471
138	ORM2	-4.7008675	2.75E-15	3.08E-13
139	IGF1	-4.6886899	5.90E-10	3.30E-08
140	TMEM171	-4.6727334	0.00392377	0.03019131
141	LRCOL1	-4.6438222	0.0000165	0.0003282
142	SLPI	-4.617772	0.00207155	0.01801857
143	C9orf163	-4.6171926	8.14E-06	0.00017729
144	ISM1	-4.612473	0.00188078	0.01668523
145	C1S	-4.604437	4.08E-18	6.15E-16
146	NAP1L2	-4.5748486	0.00199783	0.01752286
147	PSMC1	-4.5536849	5.38E-14	5.26E-12
148	CYB561	-4.5320818	4.54E-09	2.13E-07
149	KRT5	-4.5308566	0.00662562	0.04523359
150	ACSM5	-4.5235734	4.98E-07	0.00001534
151	BHLHA15	-4.5210054	1.34E-06	0.00003658
152	UGT2A2	-4.5133249	0.00130144	0.01240963

153	PTPRT	-4.501644	0.00001263	0.00026148
154	SPRN	-4.4970457	1.93E-11	1.35E-09
155	HLA-H	-4.4552112	3.09E-06	0.00007642
156	UGT2A1	-4.4529819	0.00132944	0.01263786
157	DBH	-4.4508388	1.09E-06	0.00003067
158	FAM198A	-4.415625	2.56E-08	1.06E-06
159	SLC5A6	-4.4008099	8.02E-28	2.17E-25
160	TAT	-4.3862092	1.23E-06	0.00003403
161	CAMK2B	-4.3742218	0.00660533	0.04514696
162	DMRTA1	-4.3715974	0.00002391	0.00044959
163	HRG	-4.3586487	0.00018348	0.00249184
164	PANX2	-4.3566836	2.05E-09	1.02E-07
165	SLC41A2	-4.3232264	0.00002567	0.00047961
166	FICD	-4.3202165	3.09E-06	0.00007642
167	SPTBN2	-4.2932276	6.97E-12	5.22E-10
168	FAM83G	-4.2925748	1.63E-06	0.00004301
169	OLFM2	-4.2836349	0.00012311	0.00177053
170	ADAMTS17	-4.2751891	0.00025514	0.00330626
171	GSTA2	-4.2732153	0.0008215	0.00856374
172	CYP2D7P	-4.2581084	0.00295582	0.02405322
173	SULT2A1	-4.2405588	0.00064562	0.00706102
174	RPS28	-4.2351519	0.00095356	0.00966519
175	TNK1	-4.2270229	1.52E-06	0.00004084
176	PLEKHF1	-4.1547935	0.00007616	0.00120852
177	IRF6	-4.1330496	0.00004403	0.00076046
178	CES2	-4.13141	1.35E-07	4.78E-06
179	SCARA5	-4.1213325	0.00446843	0.03354608
180	KDM8	-4.1068897	1.02E-06	0.00002905
181	KLHL25	-4.1045326	1.02E-11	7.47E-10
182	SYT12	-4.0933698	1.53E-08	6.64E-07
183	SYNPO	-4.0917141	0.00306001	0.0246867
184	SRD5A1	-4.0909081	4.31E-15	4.66E-13
185	ANKRD35	-4.081271	0.00007738	0.00122379
186	ITIH4	-4.0689458	4.85E-07	0.00001499
187	OGDHL	-4.0575455	5.03E-12	3.88E-10
188	RTN4RL1	-4.0105323	0.00205061	0.01788006
189	ACACB	-4.0067098	4.53E-06	0.00010788
190	C3	-3.9989077	1.16E-14	1.22E-12
191	ABCC3	-3.9339332	5.23E-11	3.45E-09

192	KCTD14	-3.9310638	0.00002697	0.0005029
193	SAA2-SAA4	-3.9303585	0.00006021	0.00098813
194	ALPL	-3.9202307	0.00019421	0.00261809
195	TBX15	-3.9181524	0.00353731	0.02776974
196	LAMB3	-3.9119344	1.60E-06	0.00004233
197	SLC27A2	-3.9009702	8.67E-06	0.0001874
198	CP	-3.8978628	0.00286079	0.02348463
199	KCNE1	-3.8895537	0.00491138	0.03609042
200	ALG1L9P	-3.8870154	0.00634697	0.0438015
201	MRC1	-3.883773	0.00194753	0.01716621
202	UGT2B7	-3.8729246	0.0001225	0.0017633
203	MYRIP	-3.8551353	0.00028399	0.00360653
204	C6	-3.8516278	2.01E-06	0.00005212
205	C1orf226	-3.8453763	3.34E-11	2.27E-09
206	DES	-3.835616	0.00016579	0.00227768
207	ACTR3C	-3.8299962	3.56E-06	0.00008699
208	ALAS1	-3.826774	0.000031	0.0005685
209	LRP2	-3.804841	0.00044898	0.00527315
210	HLA-C	-3.7925779	0.0000466	0.0007994
211	LOC100132529	-3.7882593	0.00164234	0.01497491
212	MRO	-3.7871963	1.28E-08	5.60E-07
213	CUX2	-3.7813713	0.00075369	0.00799377
214	SUSD2	-3.7798129	1.08E-06	0.00003029
215	CLDN2	-3.7751016	0.00001386	0.00028259
216	TMEM45A	-3.7699407	1.14E-11	8.31E-10
217	TREH	-3.7413719	0.00466817	0.03473816
218	CNTNAP3P2	-3.7137302	0.000309	0.00386901
219	LOC100506022	-3.6892391	0.00009867	0.0014918
220	ABCB4	-3.6874262	3.23E-16	3.99E-14
221	LOC100652824	-3.6782123	0.00695118	0.04695419
222	SSTR1	-3.6765411	0.00032937	0.00407255
223	ALDH1A3	-3.6621547	0.00066349	0.00721653
224	CYP1A1	-3.6574691	0.00122499	0.0118281
225	GABRB3	-3.6381362	0.00296087	0.02408328
226	FGL1	-3.6308829	0.0012774	0.01224119
227	BST2	-3.6276587	0.00010085	0.00150439
228	SLAMF8	-3.5993563	0.00003912	0.00069233
229	SLC2A10	-3.5969141	2.17E-10	1.32E-08
230	ACAA2	-3.5915688	1.02E-12	8.65E-11

231	SNX29P2	-3.5892025	0.00238037	0.02017249
232	SLC46A3	-3.568231	1.37E-11	9.84E-10
233	MOCOS	-3.5579925	6.66E-06	0.00015057
234	SGK2	-3.5559213	5.80E-08	2.22E-06
235	CNTNAP3B	-3.5350021	0.0004092	0.00487014
236	RASL10B	-3.5086496	1.54E-06	0.00004111
237	CFHR3	-3.4891393	1.13E-06	0.00003154
238	STOX2	-3.4778401	0.0023456	0.01993483
239	MCHR1	-3.465135	0.00031086	0.00388688
240	PGAM1	-3.462702	7.00E-08	2.64E-06
241	ANO1	-3.4329579	0.00025152	0.00326652
242	TPSG1	-3.4323847	0.0039434	0.03030155
243	HPX	-3.4289861	5.52E-06	0.00012744
244	PLA2G16	-3.4264359	1.74E-06	0.00004559
245	ALDH3A1	-3.4252206	0.00199003	0.01746304
246	ENTPD5	-3.4242023	0.00003584	0.00064184
247	CCDC113	-3.4180575	0.00001962	0.00038087
248	KCNIP3	-3.4113332	0.00035559	0.00432172
249	GRAMD4	-3.4054655	3.61E-08	1.46E-06
250	LOC100128531	-3.4020813	0.00066311	0.00721653
251	SLC27A5	-3.4002933	0.00074126	0.00789362
252	PLGLB2	-3.3934704	0.00010088	0.00150439
253	PLGLB1	-3.3934704	0.00010088	0.00150439
254	CLDN3	-3.3786514	0.00014415	0.0020115
255	ALDH1L1	-3.37764	5.59E-06	0.00012847
256	CD14	-3.3753589	0.00003732	0.0006652
257	ANGPTL4	-3.3752778	0.00378845	0.02940427
258	FBP1	-3.3576919	3.81E-06	0.00009214
259	TSLP	-3.3557945	0.00018827	0.002553
260	NBPF8	-3.3451828	0.00001119	0.00023542
261	AR	-3.3419211	0.00745357	0.04951894
262	FGG	-3.3376946	0.00010255	0.00152405
263	NUCB1-AS1	-3.3329708	9.05E-14	8.62E-12
264	PTGR1	-3.3325634	2.12E-07	7.13E-06
265	PC	-3.3313502	3.39E-10	1.98E-08
266	ECHDC3	-3.3080498	0.00001317	0.00027105
267	LOC286190	-3.3026595	0.00010261	0.00152405
268	LOC389641	-3.2981048	0.00080694	0.0084465
269	ACE	-3.2861723	5.23E-06	0.00012215

270	GPR64	-3.2852887	0.00057388	0.00641832
271	SMPD3	-3.2816932	0.00628267	0.04344192
272	FLJ23867	-3.279676	5.75E-07	0.00001755
273	LOC100507642	-3.2778824	0.0069413	0.04693883
274	DHX58	-3.2760719	0.00005645	0.00093582
275	MDGA1	-3.273061	0.00092344	0.00943903
276	PCDH1	-3.2708415	5.63E-08	2.17E-06
277	SYTL5	-3.2654021	4.50E-06	0.00010732
278	TLCD2	-3.2625025	0.00015521	0.00215398
279	REEP6	-3.2596523	0.00004795	0.00081464
280	LOC100129083	-3.2587965	0.00049638	0.00569464
281	CREB3L3	-3.2528211	0.00003504	0.00063271
282	SYNM	-3.2486366	6.17E-10	3.43E-08
283	STARD5	-3.2368021	3.33E-07	0.00001071
284	LOC100507472	-3.223085	3.35E-08	1.36E-06
285	SERPING1	-3.2225983	1.55E-06	0.00004128
286	SLC16A13	-3.2147166	0.00030525	0.00383822
287	SCNN1A	-3.212875	0.00012561	0.0017958
288	CPT2	-3.2095069	0.00002268	0.00043148
289	MPZ	-3.2092654	0.0000177	0.00034934
290	MGC32805	-3.195629	0.00532891	0.03842903
291	GPC1	-3.1905312	0.00048846	0.00563282
292	CPN2	-3.1896585	0.0000823	0.00128884
293	ASPG	-3.1826007	1.08E-06	0.00003029
294	LOC101929372	-3.1789533	0.00001811	0.00035679
295	RTN4R	-3.173696	0.00099048	0.00992985
296	CPLX1	-3.1499504	0.0029715	0.02413663
297	A1BG-AS1	-3.1453065	0.00156901	0.0144109
298	ACSM2B	-3.1347094	0.0000539	0.0008999
299	WNT3	-3.1339316	0.00099719	0.00998589
300	CHPF	-3.1318874	9.71E-12	7.15E-10
301	F9	-3.127475	2.06E-09	1.03E-07
302	ADRA2B	-3.1265609	0.00020111	0.00269588
303	LINC00963	-3.1257918	0.00005153	0.00086887
304	ATP6V0E2	-3.1228173	1.73E-08	7.46E-07
305	SAMD12	-3.1198703	0.00280103	0.02311047
306	SLC27A4	-3.1108693	1.45E-11	1.03E-09
307	PPM1E	-3.1062851	0.00140951	0.01327142
308	RPL18A	-3.106207	2.29E-07	7.59E-06

309	SLC6A13	-3.1024835	0.00563765	0.04009321
310	SULT1A2	-3.1019657	0.00013248	0.00187507
311	RGPD1	-3.0999447	0.00028257	0.00359365
312	ARHGEF35	-3.0968281	0.00585624	0.04126254
313	ETNPPL	-3.0922175	0.00061803	0.00683903
314	NOMO2	-3.0891774	0.00173125	0.01561541
315	LIPG	-3.0884359	9.14E-08	3.36E-06
316	PDE4DIP	-3.0865705	1.24E-06	0.00003403
317	C17orf96	-3.0864486	0.00025902	0.00334672
318	TRIM16L	-3.0848355	7.27E-06	0.0001616
319	HTATSF1P2	-3.0797214	0.00028845	0.003655
320	C1orf204	-3.0784606	0.00572934	0.04061793
321	MVP	-3.0748141	0.00289317	0.02367319
322	RPS3A	-3.0726046	1.24E-06	0.00003403
323	CACNA1H	-3.0669272	0.00002012	0.00038847
324	HLA-B	-3.0653233	0.00201261	0.01762296
325	CNTNAP2	-3.0627649	0.00078266	0.00823482
326	EFEMP1	-3.0615284	0.00129148	0.01233622
327	NAV2-AS2	-3.0611105	0.0021269	0.01836538
328	MYOM1	-3.0505358	0.00006813	0.00109679
329	AKR1C2	-3.0428223	0.00027242	0.00348845
330	SERPINA10	-3.0415232	1.64E-07	5.70E-06
331	SLC1A2	-3.0375833	0.00040168	0.00479031
332	CEP170B	-3.0335851	1.46E-09	7.55E-08
333	ABCB11	-3.0293167	0.000387	0.00463759
334	SLC22A18AS	-3.0102084	0.00001284	0.00026488
335	SIRPA	-3.0055124	5.16E-11	3.43E-09
336	VNN1	-3.0053017	0.00304749	0.02461915
337	G0S2	-2.9988337	0.00026109	0.00336863
338	TMTC1	-2.9980619	2.18E-06	0.0000564
339	SNHG22	-2.9893424	0.00335119	0.0265309
340	ABCC9	-2.9881865	0.0005597	0.00629927
341	HYOU1	-2.985058	2.26E-06	0.00005838
342	RAPGEF4	-2.9832713	0.00004369	0.00075601
343	H6PD	-2.9806025	1.23E-06	0.00003401
344	CYP8B1	-2.9777401	0.00069447	0.00750301
345	SLC25A34	-2.9774632	0.00101	0.01009149
346	ACAT1	-2.9774512	1.59E-10	9.93E-09
347	LOC102724009	-2.9758806	6.28E-07	0.00001897

348	LRFN3	-2.973694	0.0000696	0.00111842
349	ZBTB16	-2.9731703	0.00390306	0.030097
350	STAB1	-2.9728666	0.000697	0.00752129
351	ANG	-2.9685864	2.19E-10	1.32E-08
352	CYP4F3	-2.9614231	0.00006989	0.00112204
353	HELZ2	-2.9525499	0.00005233	0.00088157
354	SOD2	-2.9461881	0.0003495	0.00427687
355	C8A	-2.9375112	1.89E-06	0.00004935
356	ABCG8	-2.9367566	0.00109297	0.01074559
357	TNFRSF10A	-2.9321596	0.00241257	0.02038723
358	TPSAB1	-2.9283605	0.00314041	0.02515785
359	RARRES3	-2.9271565	0.00259835	0.02168944
360	PLA1A	-2.924413	0.00002236	0.0004267
361	ASB13	-2.9176198	3.08E-10	1.81E-08
362	PDE11A	-2.9067283	0.00741929	0.04938761
363	FGB	-2.9046517	0.00344507	0.02712936
364	POR	-2.9030258	0.00002146	0.00041165
365	TDO2	-2.9017111	4.77E-09	2.23E-07
366	ACSL1	-2.8910829	1.00E-07	3.64E-06
367	STEAP4	-2.8888792	6.08E-06	0.00013836
368	ERN1	-2.8884146	1.17E-06	0.00003253
369	SATB2	-2.8874437	0.00105028	0.01040054
370	MLYCD	-2.8811541	8.54E-06	0.00018472
371	LOC100133286	-2.8792071	6.51E-07	0.00001962
372	CCNT2-AS1	-2.8730545	1.74E-07	6.01E-06
373	CD1D	-2.8724546	0.00183375	0.01635766
374	CFI	-2.8670679	9.38E-06	0.00020029
375	RAB43	-2.8593095	3.99E-09	1.91E-07
376	PLEC	-2.8590758	0.0001057	0.00155922
377	GAS6-AS1	-2.8586171	0.00163675	0.01494482
378	INHBE	-2.855523	0.00751874	0.04988313
379	NBPF15	-2.8493703	0.00032189	0.00399943
380	GPT2	-2.84667	0.00385015	0.0297922
381	GPR153	-2.8462691	0.00043185	0.00509206
382	SLC25A22	-2.845018	5.99E-08	2.29E-06
383	TMEM38A	-2.8442941	0.00010001	0.001504
384	B4GALT1-AS1	-2.8442523	1.39E-06	0.00003774
385	WDR72	-2.8330558	0.00181678	0.01623643
386	FGGY	-2.8314166	0.00006076	0.00099438

387	FGA	-2.8293126	0.0015246	0.01408905
388	SOWAHB	-2.8252684	0.00246215	0.02071769
389	SHMT1	-2.8246337	2.92E-07	9.47E-06
390	RPL36A	-2.8230343	2.19E-07	7.32E-06
391	GCKR	-2.8099609	7.42E-06	0.00016386
392	ETFDH	-2.7929738	0.00001717	0.00034044
393	IFI6	-2.7917777	0.00086769	0.00895608
394	HPN-AS1	-2.7821565	2.56E-06	0.000065
395	ROM1	-2.780466	0.00018869	0.0025548
396	HPS5	-2.7793152	1.32E-08	5.73E-07
397	PITPNM2	-2.7750319	4.81E-06	0.00011368
398	HAO1	-2.7686023	0.00302774	0.02450407
399	ABHD2	-2.7679334	2.42E-08	1.02E-06
400	CD99L2	-2.7667054	0.00001223	0.00025505
401	DHODH	-2.7660198	0.00211756	0.01834708
402	RPSAP58	-2.7581898	0.00488692	0.03594038
403	PTPRF	-2.7536395	9.68E-09	4.32E-07
404	TPSB2	-2.7518106	0.00003341	0.00060631
405	ITIH3	-2.7507171	4.75E-11	3.17E-09
406	CAND2	-2.7417903	0.00002763	0.00051249
407	SIAE	-2.7398507	9.63E-07	0.00002774
408	PRDX4	-2.7395834	0.00005246	0.00088283
409	NOL3	-2.739547	0.00180914	0.01617869
410	CDHR2	-2.7363911	0.00693337	0.04693134
411	KCTD21-AS1	-2.732119	0.00002337	0.00044175
412	LAP3	-2.7309739	4.89E-08	1.90E-06
413	TBC1D2B	-2.7292776	0.00001277	0.00026369
414	ACMSD	-2.7292033	7.47E-10	4.13E-08
415	ABLIM3	-2.7283265	0.00267292	0.02224934
416	IL1RL2	-2.7245811	0.00544366	0.03906664
417	C4BPB	-2.7218128	4.81E-07	0.0000149
418	LINC00987	-2.7213956	0.00012834	0.0018295
419	EXPH5	-2.7185722	0.00089336	0.00918908
420	SEC16A	-2.7149295	6.28E-09	2.87E-07
421	GALNT2	-2.7109935	0.00006522	0.00105855
422	LRP3	-2.7041672	0.00001835	0.00036069
423	ABCA3	-2.7000208	0.0000288	0.00053255
424	LRP1	-2.6938471	0.00003691	0.00065915
425	C5	-2.6899847	2.86E-06	0.00007212

426	EFNA2	-2.6891668	0.00071023	0.00763626
427	PGLYRP2	-2.6867848	0.00460944	0.0343969
428	C1RL	-2.6847155	5.82E-07	0.00001775
429	PITPNM3	-2.6841014	0.00055339	0.006248
430	GMPPB	-2.681714	4.60E-08	1.81E-06
431	NOS1AP	-2.6812459	0.0020132	0.01762296
432	ACOT4	-2.6800476	0.00001586	0.00031871
433	ENPEP	-2.6773163	0.00013621	0.00192015
434	TGM2	-2.6770383	0.00147307	0.01374123
435	SNED1	-2.6734959	0.00071798	0.0077103
436	MYO1E	-2.6724338	0.00012195	0.00175821
437	ADAMTSL5	-2.6699408	0.00223608	0.01917794
438	MGLL	-2.659949	0.00013018	0.00184838
439	AKR1C3	-2.6596634	0.00140816	0.01326579
440	CCL23	-2.6518323	0.00074774	0.00795311
441	HFE	-2.6463737	0.00144344	0.01352654
442	ALDH2	-2.6460898	6.64E-12	4.99E-10
443	KCNS3	-2.6441249	0.00700231	0.04718695
444	NACC2	-2.6433397	3.69E-07	0.00001165
445	PPL	-2.6418618	0.00008834	0.00136778
446	OPLAH	-2.6411768	5.79E-08	2.22E-06
447	A1BG	-2.6387109	0.0000483	0.00081903
448	ACSL5	-2.6377898	2.31E-07	7.66E-06
449	CFH	-2.6377632	0.00144591	0.01354259
450	PCSK6	-2.6356145	1.15E-06	0.00003221
451	HTATIP2	-2.6261981	1.79E-07	6.17E-06
452	LOC100294362	-2.6184212	0.00208124	0.01808519
453	GK	-2.615621	0.00062416	0.00688549
454	ABAT	-2.6139215	6.97E-06	0.00015627
455	IL4I1	-2.6104082	0.00015729	0.00218112
456	SEC16B	-2.6100448	0.00022057	0.00291221
457	FUT6	-2.6083153	0.00439262	0.03302814
458	RDH12	-2.6069496	0.00158758	0.01455214
459	CD81	-2.5937028	9.59E-09	4.32E-07
460	EGFR	-2.5918663	0.00002763	0.00051249
461	SHROOM3	-2.591196	5.24E-08	2.03E-06
462	MAN2B2	-2.5901407	0.00007274	0.00116154
463	IGFBP6	-2.5801775	0.0013988	0.01319155
464	GPR125	-2.5740871	0.00003572	0.00064111

465	ADAMTSL2	-2.5645778	0.00314613	0.02518642
466	SMPD1	-2.5644182	2.12E-07	7.13E-06
467	PLXNB2	-2.5419585	0.00024862	0.00324057
468	SUSD4	-2.5408371	0.00346421	0.02726805
469	SETBP1	-2.5399326	5.56E-06	0.00012798
470	DCPS	-2.5360135	0.00001376	0.00028165
471	SLC23A2	-2.5357733	2.47E-08	1.04E-06
472	MYO1B	-2.5347936	0.00001165	0.00024344
473	AMOTL1	-2.5310568	0.00025008	0.00325245
474	MSTO1	-2.5282609	9.07E-06	0.00019434
475	HSD3B7	-2.5233989	1.26E-08	5.56E-07
476	NBPF10	-2.5231042	0.00070449	0.00759284
477	SLC13A3	-2.5229106	0.0005176	0.00588879
478	METTL7B	-2.5197016	0.00528583	0.03821123
479	GRTP1-AS1	-2.5192661	0.00010541	0.0015562
480	SLC25A47	-2.5189489	0.00751959	0.04988313
481	TBC1D9B	-2.5172718	0.00002273	0.00043191
482	LRP5	-2.5171355	0.00022589	0.00296826
483	SLFN5	-2.5106295	0.00116012	0.01129973
484	UGT2B4	-2.5065647	0.00079483	0.00834433
485	GAA	-2.5057303	2.62E-08	1.08E-06
486	F11	-2.5054784	0.00061941	0.00685009
487	DNAJC22	-2.4990013	0.00019807	0.00265959
488	LOC101928303	-2.4966351	0.00546627	0.03921309
489	DPP9-AS1	-2.4942512	0.00002872	0.00053148
490	VWA1	-2.4896312	6.23E-08	2.37E-06
491	TPPP	-2.4878043	0.0034332	0.02707183
492	PTPN3	-2.4872637	0.00003041	0.00055873
493	DDR2	-2.4853044	0.00158063	0.01449926
494	KIAA1671	-2.4810482	7.09E-06	0.00015807
495	SLC38A7	-2.4802245	0.00001618	0.00032341
496	FKBP11	-2.4792759	0.00005889	0.00097181
497	BOK	-2.4768577	0.0031366	0.02514505
498	STEAP3	-2.4688655	0.00125118	0.0120483
499	SYNPO2	-2.4611193	0.0000275	0.00051114
500	MAT1A	-2.4571622	0.0016644	0.01511969
501	ANKEF1	-2.4505984	0.00019525	0.00262566
502	OAS3	-2.4483742	0.00636044	0.0438418
503	TRAM2	-2.4475454	0.00005282	0.00088631

504	SDF2L1	-2.4462842	0.00062194	0.0068696
505	ACOT2	-2.4419956	0.00034777	0.00426613
506	FBXL8	-2.4326882	0.00107304	0.0105906
507	GDPGP1	-2.4326779	0.00278502	0.02299971
508	PARP3	-2.4305182	0.00010058	0.00150439
509	IVD	-2.4284702	0.00054596	0.00617584
510	UGDH	-2.4262449	0.00508935	0.03718136
511	NFIC	-2.4182645	0.00094106	0.00957456
512	PCK2	-2.4177421	2.06E-07	6.98E-06
513	ABCB1	-2.4137827	0.0030952	0.02492998
514	QSOX1	-2.4122044	1.07E-09	5.71E-08
515	CRELD2	-2.4120243	0.00016056	0.00221951
516	LTBR	-2.4104193	0.00448032	0.03361655
517	C19orf66	-2.4085648	0.00034941	0.00427687
518	NKX3-1	-2.4075719	0.00049878	0.00570527
519	LOC100506083	-2.3997401	0.0013337	0.01266329
520	HLA-A	-2.3989324	0.00481653	0.03556958
521	STAT3	-2.3931675	0.00209205	0.0181702
522	EEF1A1	-2.3927237	0.00001638	0.00032662
523	KIAA1804	-2.3916615	0.0029549	0.02405322
524	DDT	-2.3879686	0.00191327	0.01690607
525	IKBKG	-2.3876201	0.00108761	0.01071069
526	SMIM14	-2.386135	0.0001626	0.00224119
527	ITPRIPL2	-2.3822644	0.00069023	0.00746632
528	CPT1A	-2.3815571	0.00011033	0.00161818
529	ECHS1	-2.3731769	2.13E-07	7.13E-06
530	RALGAPA2	-2.373068	0.0000247	0.00046203
531	A2M	-2.3714119	0.00236008	0.02004195
532	GPX3	-2.3711405	0.00011918	0.00172522
533	MSTO2P	-2.3710695	0.00004326	0.00075004
534	BCAR3	-2.3710325	2.38E-06	0.0000609
535	ASRGL1	-2.3701916	0.00021918	0.00290149
536	HIP1	-2.368423	6.25E-06	0.00014201
537	MASP2	-2.3620462	1.43E-06	0.00003863
538	NPPA	-2.3618915	0.00359388	0.02812703
539	HIPK2	-2.3613149	0.00004672	0.00080014
540	NQO1	-2.358846	0.00091639	0.00938254
541	CPEB3	-2.3587548	1.39E-06	0.00003774
542	PODN	-2.3521413	0.00009129	0.00140237

543	PDIA4	-2.3521121	0.00153689	0.01418579
544	P4HB	-2.3505037	0.00001154	0.00024176
545	SEC24D	-2.348022	0.00003969	0.00069976
546	NOMO1	-2.3474559	0.00073255	0.00781957
547	HPD	-2.3419906	0.00458726	0.03424579
548	DDTL	-2.3388941	0.00264408	0.02204017
549	MAPK8IP1	-2.3382591	0.00510773	0.03727176
550	PROS1	-2.3330666	0.00003949	0.00069746
551	MGST1	-2.3327607	0.00006011	0.0009874
552	MOCS1	-2.3324159	0.00009459	0.00144192
553	NTN4	-2.3312821	0.00031385	0.00391871
554	ABCC11	-2.3276101	0.00056848	0.00637795
555	MTHFD1	-2.3241242	0.0003234	0.0040126
556	MFSD9	-2.3165618	0.00038705	0.00463759
557	ABCC2	-2.3158824	0.00081534	0.00851445
558	SYNE2	-2.3108971	0.00023578	0.00308904
559	PIK3AP1	-2.3006883	0.00047918	0.00553291
560	NLN	-2.299993	0.00001801	0.00035511
561	CSRP2	-2.2998467	0.00008619	0.00133904
562	SERPINC1	-2.294848	0.00145891	0.01364277
563	FRK	-2.2931372	0.00377149	0.02928535
564	CYB5A	-2.2845256	1.73E-06	0.00004553
565	FGFRL1	-2.2844327	0.00151254	0.01401183
566	PITPNM1	-2.2839561	0.00284395	0.02336839
567	SETD7	-2.2834009	0.00001045	0.00022112
568	SLC31A2	-2.2807202	0.00265532	0.02212351
569	TNS3	-2.279722	0.00003413	0.00061825
570	DAG1	-2.2760059	9.57E-07	0.00002765
571	ABHD6	-2.2748445	0.00085202	0.00881582
572	ST6GAL1	-2.2661187	0.00001013	0.000215
573	ALDH4A1	-2.2658727	0.00148017	0.01379092
574	PAPSS2	-2.2650779	0.00027088	0.00347735
575	FITM1	-2.254732	0.00012567	0.0017958
576	CNDP2	-2.2527729	0.00004028	0.00070862
577	PDIA3	-2.2520983	0.00034234	0.00420695
578	LOC100288162	-2.2520129	0.00626247	0.04333587
579	HSPA4L	-2.247325	0.00096038	0.00969368
580	NUCB1	-2.2434724	1.04E-07	3.76E-06
581	NEURL1B	-2.2428122	0.00445102	0.03345311

582	TNFSF13	-2.2409525	0.0001759	0.00239624
583	PCCA	-2.2356941	0.0000351	0.00063324
584	ABHD17C	-2.2336089	0.00220768	0.01898927
585	NOTCH1	-2.2326863	0.00582562	0.04112814
586	C8B	-2.2321431	0.00151752	0.01403612
587	BCL9L	-2.2272748	0.00020322	0.00271643
588	APOL6	-2.2244806	0.00095376	0.00966519
589	PIM3	-2.2184256	0.00679508	0.04617992
590	RAB11FIP5	-2.2157615	0.0017624	0.01584028
591	IPO4	-2.2133277	0.0013515	0.01280657
592	CASKIN2	-2.2121088	0.00450953	0.03377877
593	GNE	-2.2098754	0.00041095	0.00488091
594	MPV17L2	-2.2064882	0.00028068	0.00357216
595	PPIA	-2.2048645	0.00001814	0.00035688
596	BCKDHA	-2.2044796	0.00009545	0.0014526
597	MYLK-AS1	-2.2031268	0.00148145	0.01379521
598	COG3	-2.2002292	7.82E-07	0.00002329
599	CLUH	-2.1980781	0.00018942	0.00256273
600	NUAK1	-2.1974283	0.00233878	0.01988632
601	LONP2	-2.1961181	0.00004088	0.00071705
602	MMACHC	-2.1957118	0.000089	0.00137321
603	CLIP4	-2.1955674	0.00079261	0.00832593
604	MYH14	-2.1944591	0.00083761	0.00870109
605	EDEM1	-2.1943936	0.00004155	0.00072672
606	HGD	-2.1896369	0.00004403	0.00076046
607	ZBTB47	-2.187694	0.00002229	0.00042577
608	PEPD	-2.1858515	6.78E-07	0.00002035
609	FAM20A	-2.184477	0.0033826	0.02673204
610	SCP2	-2.1839787	0.00027956	0.00356328
611	RPS10	-2.1786142	4.03E-06	0.00009701
612	CAPN5	-2.1781559	0.00661118	0.04516962
613	ZBTB7B	-2.1766938	0.00389537	0.03006375
614	VSIG10	-2.1730373	0.00041038	0.00487768
615	PKD1P1	-2.1718826	0.00001592	0.00031957
616	CHST3	-2.1684765	0.00123665	0.01192767
617	SRPR	-2.1671872	2.48E-07	8.16E-06
618	L3HYPDH	-2.1619786	0.00147329	0.01374123
619	PTPRH	-2.1615625	0.00023825	0.00311909
620	MCCC2	-2.1612801	0.0001095	0.0016073

621	MYD88	-2.1578379	0.00004306	0.00074792
622	CHDH	-2.1556202	0.00098636	0.00989419
623	DOLK	-2.1552693	0.00033796	0.00416716
624	SHMT2	-2.1504416	0.0000115	0.0002412
625	ADCY9	-2.1467107	0.00280874	0.02316332
626	VKORC1	-2.1453018	0.00003318	0.00060288
627	IGFLR1	-2.1441899	0.00094872	0.00962641
628	SLC4A2	-2.1421903	3.09E-06	0.00007643
629	KLHDC7A	-2.1407811	0.00341253	0.02694459
630	TPTE2P5	-2.1361092	0.00186942	0.01661757
631	LACTB2	-2.134983	0.00046384	0.00539083
632	RNF185	-2.133314	8.97E-07	0.00002619
633	RAB15	-2.1278069	0.00204336	0.01783434
634	CECR5	-2.1266412	4.14E-06	0.00009942
635	PTPRU	-2.1245663	0.00172016	0.01552326
636	SH3BP4	-2.1188459	0.00008533	0.00133038
637	NRXN2	-2.1135881	0.00230458	0.01967065
638	TFR2	-2.1120741	9.40E-07	0.00002723
639	IGFBP7-AS1	-2.1120196	0.00182727	0.016308
640	OAS1	-2.1102436	0.00386066	0.02984761
641	CBR1	-2.1099133	6.89E-06	0.00015519
642	PGBD5	-2.1093864	0.00002742	0.00051064
643	SDHA	-2.107897	0.00002299	0.00043541
644	SALL1	-2.1036501	0.00004769	0.00081172
645	PTPRD	-2.0983327	0.00046927	0.00543971
646	ABCD1	-2.0955635	0.00001868	0.00036627
647	FAAH2	-2.0937456	0.0012627	0.01214378
648	RPL7L1	-2.0929695	0.00521469	0.03788866
649	SPIRE1	-2.0917534	0.00653736	0.04475115
650	SLC25A42	-2.0894613	0.00323943	0.02582983
651	SARDH	-2.0891875	2.94E-06	0.00007357
652	IGF2-AS	-2.0868207	0.00135481	0.01283105
653	HNF4A	-2.0816685	0.00033409	0.00412224
654	ERGIC1	-2.0791597	0.00006653	0.00107492
655	SLC25A18	-2.0781647	0.00084226	0.00874427
656	TRIM35	-2.0765846	0.00035554	0.00432172
657	C14orf132	-2.076426	0.00270996	0.02245265
658	IMPA2	-2.0762905	0.00026866	0.00345629
659	PITRM1-AS1	-2.0761441	0.00008767	0.00136098

660	MYLK	-2.0742359	0.0001326	0.00187509
661	CD151	-2.074149	0.00114614	0.01118803
662	RCN1	-2.0703974	0.00104544	0.01037569
663	APMAP	-2.0692048	4.86E-06	0.0001146
664	FAM83H	-2.0686576	0.00010013	0.00150439
665	VPS18	-2.0684353	0.00075235	0.00798783
666	F2	-2.0682182	0.00348569	0.02741288
667	HS6ST1	-2.0664906	0.00288255	0.02360797
668	OSBP	-2.054479	5.68E-06	0.00013018
669	C17orf107	-2.0530386	0.00126681	0.01216587
670	GJD3	-2.049965	0.00577563	0.04085622
671	LOC101929441	-2.0485879	0.00716183	0.04799264
672	MTSS1L	-2.0435761	0.00002354	0.00044373
673	HSPD1	-2.0435237	0.00059122	0.00657097
674	CFHR1	-2.0433395	0.00662277	0.04523146
675	KCTD21	-2.0374964	3.69E-06	0.00008966
676	VWF	-2.0362242	0.00164025	0.0149691
677	DTX4	-2.0356516	0.00724537	0.04844301
678	BTN3A3	-2.0322542	0.00165265	0.01504168
679	FITM2	-2.0303586	0.00057616	0.00643167
680	MANEA	-2.0284878	0.00323796	0.02582972
681	CTSD	-2.0281269	0.0000412	0.00072196
682	GALNS	-2.0247603	0.00180358	0.01613702
683	AMOTL2	-2.023913	0.00005391	0.0008999
684	PNPLA3	-2.023601	0.00043524	0.00512865
685	DPAGT1	-2.0228216	0.00025424	0.00329692
686	ABHD8	-2.0131484	0.00013191	0.00187099
687	PON3	-2.006879	0.00019518	0.00262566
688	IRAK1	-2.0066551	3.89E-06	0.00009388
689	RAB27A	-2.0033053	0.00366143	0.02859288

Differentially expressed genes were genes with at least a 4-fold change in gene expression (|Log2FC| $\geq$ 2) and adjusted p-value < 0.05.
DEG, differentially expressed genes.

Supplementary Table S3. Upregulated DEG in iHLC Compared to Adult Liver Stage

	Gene	Log2FC	P-value	Adjusted p-value
1	CLDN6	16.2457483	2.2304E-15	1.1058E-14
2	AFP	15.2699276	0	0
3	SP8	14.9337587	3.2203E-13	1.3937E-12
4	RFX6	14.4703368	9.6281E-11	3.515E-10
5	LOC440602	14.350923	7.5534E-12	2.98E-11
6	CYMP	14.3410531	7.4316E-12	2.9344E-11
7	LIN28A	14.0420559	1.1318E-11	4.4192E-11
8	UNC13C	13.8264828	3.0632E-11	1.162E-10
9	ERVV-1	13.8215232	2.1511E-11	8.2425E-11
10	DRD2	13.783215	1.6946E-11	6.539E-11
11	OTX2	13.7715665	2.0839E-11	7.9929E-11
12	COL2A1	13.3351187	6.266E-50	1.1607E-48
13	NPSR1-AS1	12.9976281	1.3157E-09	4.4015E-09
14	ERVV-2	12.9737575	3.0726E-10	1.0811E-09
15	ZFP42	12.9067034	2.8705E-10	1.0118E-09
16	B4GALNT2	12.8725672	5.7594E-10	1.9827E-09
17	IGF2BP1	12.8078229	6.9148E-52	1.3674E-50
18	TRIML2	12.8045867	5.2477E-10	1.8134E-09
19	CCKBR	12.6516134	6.6472E-17	3.5673E-16
20	SLC5A5	12.5715639	2.3035E-17	1.2719E-16
21	ADAMTS18	12.5433721	1.5238E-09	5.0757E-09
22	IGDCC3	12.4770816	5.0234E-09	1.6037E-08
23	VSIG1	12.2664576	3.4585E-09	1.1202E-08
24	HSD3B1	12.1702658	1.1747E-15	5.9224E-15
25	FAM19A3	12.1490963	3.83E-09	1.2337E-08
26	GPR111	12.1242542	1.6897E-07	4.7511E-07
27	CTD-2297D10.2	12.067347	6.3712E-09	2.0192E-08
28	LINC01108	11.9837482	5.6367E-38	7.3383E-37
29	GSG1	11.9100921	1.5623E-14	7.3369E-14
30	PANX3	11.8794668	6.7266E-09	2.1263E-08
31	MUC19	11.7903178	1.0398E-08	3.2365E-08
32	LAMA1	11.7655065	9.374E-178	1.535E-175
33	ATP12A	11.7185659	3.2427E-34	3.6961E-33
34	URAD	11.5718523	2.1262E-08	6.4415E-08
35	HMGA2	11.5212359	4.6118E-14	2.1094E-13
36	MUC16	11.4521408	3.31E-08	9.8915E-08
37	ISL1	11.325242	8.5401E-08	2.4611E-07
38	LOC100132735	11.249993	5.8889E-08	1.7228E-07
39	PDE6A	11.1681559	6.6902E-52	1.3243E-50
40	HS3ST4	11.1654712	6.398E-08	1.8655E-07
41	HTR1E	11.1632454	7.2007E-08	2.0882E-07
42	FABP7	11.1488257	1.3943E-07	3.9544E-07

43	LINC01021	11.1037156	6.2402E-08	1.8211E-07
44	SOX11	11.0477387	1.3649E-13	6.0495E-13
45	LIN28B	10.9910065	2.3678E-07	6.5735E-07
46	APELA	10.9405897	1.411E-07	4.0001E-07
47	GADL1	10.8890268	4.5593E-07	1.2361E-06
48	TAC3	10.846952	8.6337E-14	3.8782E-13
49	LOC440416	10.8060405	1.4765E-07	4.1771E-07
50	PSG4	10.8022598	1.3968E-07	3.961E-07
51	RS1	10.785193	6.7592E-07	1.8002E-06
52	MNX1	10.6302737	2.5689E-07	7.1063E-07
53	UGT8	10.5955545	1.1047E-20	7.2359E-20
54	ADAMTS16	10.4401125	1.747E-21	1.1859E-20
55	LHX8	10.4052138	4.763E-07	1.2895E-06
56	ZNF826P	10.359974	4.6821E-07	1.2682E-06
57	HTR3B	10.3520773	1.8039E-06	4.6387E-06
58	TMEM215	10.3220797	1.499E-06	3.8807E-06
59	NPFFR2	10.3189393	7.5128E-07	1.9951E-06
60	LPPR3	10.3009475	2.9888E-12	1.2101E-11
61	MEP1A	10.2774014	2.9401E-52	5.8559E-51
62	NYAP2	10.266224	4.1147E-06	1.0217E-05
63	GPR115	10.2185417	4.1849E-20	2.6553E-19
64	C6orf222	10.2127985	1.1287E-06	2.9557E-06
65	SLC22A6	10.2058766	9.8982E-07	2.6046E-06
66	TRIM71	10.1577635	4.9049E-12	1.9616E-11
67	LINC00616	10.1231881	2.1116E-06	5.395E-06
68	C4orf26	10.0667156	1.7776E-06	4.5736E-06
69	TMEM207	10.0635945	1.4392E-06	3.7318E-06
70	NDP	10.0323934	2.425E-06	6.159E-06
71	CHRNA2	10.0104293	2.3118E-06	5.8861E-06
72	OPRK1	9.89731275	7.8299E-06	1.8941E-05
73	KCNF1	9.87731804	2.0621E-06	5.2725E-06
74	XKRX	9.87209016	5.9709E-40	8.2163E-39
75	GLRA2	9.77569756	6.577E-06	1.6042E-05
76	ZSCAN4	9.751747	3.3494E-06	8.3932E-06
77	WIF1	9.70472084	1.4909E-10	5.3641E-10
78	CDH3	9.6792432	3.3782E-44	5.2634E-43
79	PSAPL1	9.65131113	2.9297E-06	7.3824E-06
80	ABCA12	9.64514274	3.1435E-06	7.9037E-06
81	CDH7	9.63943471	4.9418E-06	1.2183E-05
82	SLC18A3	9.63770447	5.7579E-06	1.4101E-05
83	KLK6	9.6200398	2.7257E-09	8.8938E-09
84	ALPK2	9.6080497	1.187E-136	1.141E-134
85	CLDN18	9.5935719	6.809E-10	2.3271E-09
86	RIMS4	9.59093781	2.0791E-09	6.8477E-09
87	SVOPL	9.58862647	4.8671E-06	1.2008E-05

88	SYT6	9.55131046	5.4547E-19	3.279E-18
89	NKAIN4	9.52262295	1.0787E-10	3.9242E-10
90	CDX2	9.47971643	1.5559E-51	3.0425E-50
91	HSD3BP4	9.46410736	9.877E-10	3.3361E-09
92	KHDRBS2	9.44641783	8.8769E-16	4.5073E-15
93	KLK7	9.4336079	1.0742E-05	2.5634E-05
94	CSMD3	9.41256249	1.2428E-05	2.9505E-05
95	BANCR	9.38835405	1.0916E-05	2.6027E-05
96	PPEF1	9.38262933	7.2697E-06	1.7642E-05
97	NLRP7	9.37281336	6.207E-17	3.3432E-16
98	KCNG3	9.36035048	6.718E-06	1.6371E-05
99	FAR2P1	9.35246249	2.3684E-10	8.3991E-10
100	SYT16	9.34851682	1.1404E-05	2.7144E-05
101	DGKK	9.34453598	7.6494E-06	1.8524E-05
102	PTPN5	9.3357809	1.3223E-09	4.423E-09
103	PCDH15	9.31670757	1.7867E-08	5.4499E-08
104	PRR15	9.30452942	3.5121E-56	7.8534E-55
105	FLJ16779	9.27627996	4.6195E-10	1.6023E-09
106	SIM1	9.26419891	1.132E-05	2.6955E-05
107	KCNJ13	9.23086415	2.1294E-41	3.0771E-40
108	LINC00032	9.23074897	1.3652E-05	3.2304E-05
109	SPAG6	9.22418031	5.3952E-05	0.00011991
110	LIX1	9.21181842	0.00015521	0.00032771
111	LGSN	9.18312778	8.8107E-97	4.7284E-95
112	BLACAT1	9.16633152	1.4693E-05	3.4656E-05
113	VRTN	9.16627735	1.0941E-05	2.6083E-05
114	PSG5	9.15053315	9.7874E-06	2.3464E-05
115	EQTN	9.13959241	2.1026E-05	4.8819E-05
116	MYO3A	9.12996297	2.2209E-26	1.8877E-25
117	FOXE1	9.11442721	1.2987E-05	3.0798E-05
118	CHST6	9.09433336	1.8341E-05	4.2895E-05
119	LPO	9.09390216	2.4607E-05	5.6745E-05
120	CHRNA6	9.07957948	1.6934E-09	5.6215E-09
121	TTTY16	9.04051795	2.5208E-05	5.8062E-05
122	LINC00491	8.99299953	1.6606E-05	3.8996E-05
123	OTOG	8.99272022	6.5058E-05	0.00014334
124	STRA6	8.98366054	5.288E-08	1.5534E-07
125	HAVCR1	8.96837034	1.0626E-48	1.8939E-47
126	ATP6V0A4	8.95663118	1.686E-05	3.9564E-05
127	ZNF804B	8.94725899	2.5969E-05	5.9743E-05
128	EGF	8.9444213	2.2169E-85	9.8012E-84
129	MALRD1	8.94223974	1.7351E-23	1.2923E-22
130	CDH9	8.92834707	2.2698E-05	5.2542E-05
131	LOC101927378	8.9173828	1.8369E-05	4.2953E-05
132	PTCHD4	8.91512544	1.0887E-17	6.1249E-17

133	CDH17	8.91359434	8.6534E-82	3.542E-80
134	SLC2A7	8.89914028	1.771E-05	4.1484E-05
135	NR0B1	8.89577603	2.8686E-05	6.5719E-05
136	GRID2	8.88609278	6.3017E-25	5.0476E-24
137	KCNH5	8.88248583	2.0821E-05	4.8373E-05
138	DPEP1	8.87533289	4.8659E-74	1.6908E-72
139	ITGB6	8.86067709	1.0009E-74	3.5224E-73
140	CDH10	8.84900444	3.4401E-05	7.8164E-05
141	FLJ22447	8.83465984	1.0719E-27	9.6591E-27
142	CLDN19	8.79811299	2.878E-31	2.9513E-30
143	ATP13A4-AS1	8.79589605	3.2766E-05	7.4643E-05
144	TNNT1	8.77417823	5.2378E-17	2.8346E-16
145	SYNPR	8.73451432	4.656E-05	0.00010437
146	MMP16	8.73027707	3.0206E-30	2.979E-29
147	S100A14	8.72218232	4.615E-268	1.682E-265
148	LINC01012	8.68047695	3.6219E-05	8.212E-05
149	BIRC7	8.664068	5.9505E-05	0.00013176
150	ELAVL2	8.65254381	0.00032897	0.00066902
151	TGFB2-AS1	8.64832331	3.4697E-05	7.8808E-05
152	CCDC27	8.61281496	3.6742E-05	8.3257E-05
153	FAM19A4	8.57888738	1.0714E-27	9.659E-27
154	TMIGD1	8.56804489	8.076E-07	2.1406E-06
155	LOC100132146	8.54610581	6.1677E-05	0.00013632
156	TGM7	8.54074963	9.7222E-05	0.0002102
157	ALX1	8.53389586	5.1775E-05	0.00011537
158	CACNG4	8.51104737	1.2151E-41	1.7704E-40
159	ZNF730	8.49622358	8.7476E-09	2.7347E-08
160	BBOX1-AS1	8.48833596	5.9763E-05	0.00013231
161	HYAL4	8.48413866	9.0005E-05	0.0001954
162	FAM101A	8.48296533	4.0965E-38	5.3476E-37
163	AMER3	8.47635045	7.5623E-05	0.00016539
164	TRIM60	8.47233025	0.00014094	0.00029923
165	SERTM1	8.45880974	4.8942E-05	0.00010933
166	SCUBE2	8.41763781	1.2453E-17	6.9757E-17
167	IGSF1	8.41475054	3.1527E-75	1.1197E-73
168	TUBB4A	8.41216908	2.175E-150	2.458E-148
169	SCGB2A1	8.41008909	1.3973E-06	3.6265E-06
170	PCSK1N	8.40991536	2.6486E-49	4.8184E-48
171	MAGEA10-MAGEA5	8.40454946	0.00011075	0.00023811
172	FRMPD2	8.3970363	0.00013353	0.00028455
173	FRRS1L	8.39387129	6.7894E-05	0.00014933
174	SELV	8.39342045	7.7758E-05	0.00016994
175	LCN9	8.39133715	0.00012193	0.00026081
176	SLC30A8	8.39015005	8.0484E-05	0.00017568

177	ARHGAP40	8.38181866	1.524E-12	6.2995E-12
178	KCNK12	8.3721206	6.0858E-07	1.6283E-06
179	LINC00479	8.36844828	3.1862E-47	5.414E-46
180	ZBTB8B	8.36618451	7.1414E-05	0.00015666
181	LOC101928266	8.34499982	8.4803E-05	0.00018459
182	C7orf34	8.34367207	0.00014955	0.00031639
183	PIFO	8.33171569	2.4164E-38	3.1845E-37
184	BPIFA2	8.32870244	0.00012974	0.00027679
185	PITX2	8.3158323	7.949E-289	3.656E-286
186	ZMAT4	8.30626706	2.8842E-14	1.334E-13
187	MAGEA5	8.29441393	0.00016684	0.00035093
188	MFSD6L	8.29101813	5.251E-44	8.129E-43
189	PSG9	8.29089115	0.00010235	0.00022068
190	C9orf135	8.27704547	8.3765E-05	0.00018241
191	FSHR	8.26843098	0.00010233	0.00022066
192	HEPACAM2	8.26541864	1.8399E-13	8.1029E-13
193	LINC00837	8.21885304	0.00010828	0.00023305
194	ST8SIA2	8.20128407	2.2841E-08	6.9047E-08
195	LRRTM3	8.19743802	0.00017061	0.00035856
196	LOC283299	8.18394898	0.00010397	0.00022406
197	FLJ31356	8.18291882	0.00019942	0.00041561
198	LINC00461	8.18192502	0.00032138	0.00065433
199	AFF2	8.17765138	1.2623E-62	3.3967E-61
200	KLRG2	8.17520605	6.8058E-18	3.8684E-17
201	HOXC8	8.16858372	0.00017251	0.00036244
202	KLHL1	8.16092902	0.00080791	0.00156669
203	SI	8.159461	6.5524E-59	1.5765E-57
204	HOXC5	8.15253863	0.00015779	0.00033289
205	ATP6V0D2	8.13771783	0.00051094	0.00101358
206	LOC101927780	8.11870008	9.3512E-08	2.6865E-07
207	HHLA2	8.11778478	1.205E-42	1.8019E-41
208	GPC3	8.06146626	4.963E-113	3.4E-111
209	GABRB1	8.03957494	1.8701E-19	1.1547E-18
210	ISM2	8.03229974	3.5339E-11	1.3327E-10
211	FOLR1	8.02744465	7.8908E-28	7.1438E-27
212	PSG8	8.02046627	0.00021787	0.00045168
213	PRTG	8.0082747	5.3301E-46	8.7343E-45
214	CALB1	8.00663328	3.9894E-20	2.537E-19
215	CCDC129	7.99606097	0.00015604	0.00032929
216	FMO9P	7.99301742	0.00023052	0.00047664
217	ELSPBP1	7.96738328	0.0002029	0.00042242
218	LOC100506801	7.95866234	0.00018945	0.00039599
219	ZG16	7.95708313	1.7555E-25	1.4323E-24
220	LINC01356	7.95441267	4.6275E-62	1.2264E-60
221	CTSV	7.91908338	6.081E-130	5.439E-128

222	GIF	7.91261037	0.00074498	0.00145062
223	IGFL2	7.91077553	0.00019447	0.00040578
224	OLFM3	7.91016512	0.00042809	0.00085751
225	COL11A1	7.90102958	4.8202E-40	6.6471E-39
226	LINGO2	7.89585117	1.1351E-07	3.2393E-07
227	CXCL17	7.88492942	1.3776E-15	6.9166E-15
228	SPAM1	7.87239896	0.00022773	0.00047102
229	EDA2R	7.86864152	7.4497E-31	7.4846E-30
230	SLC10A4	7.86267048	2.5341E-12	1.0312E-11
231	EPS8L3	7.85699056	5.192E-111	3.471E-109
232	GJB7	7.85049766	0.00025405	0.00052293
233	LOC285000	7.83703294	0.00021105	0.00043816
234	MS4A15	7.83492526	0.00026565	0.00054575
235	FMO1	7.82477331	1.0423E-77	3.9564E-76
236	CFC1	7.81309898	0.00035133	0.0007109
237	ANK1	7.8125739	2.3892E-76	8.7588E-75
238	GPR101	7.77505476	0.00029159	0.00059646
239	CHRNB3	7.77259246	0.00035485	0.00071757
240	GABRA2	7.77121491	2.1959E-22	1.5534E-21
241	ADCY2	7.77017783	2.7925E-29	2.6696E-28
242	SLC2A14	7.75993669	1.6428E-19	1.0163E-18
243	LRTM2	7.75575628	0.00026446	0.00054338
244	TUBB3	7.75029142	6.033E-134	5.63E-132
245	OVOL1	7.74036279	2.7258E-46	4.532E-45
246	CFC1B	7.73258191	0.00045683	0.00091131
247	PRSS16	7.72963708	1.464E-46	2.4447E-45
248	IGDCC4	7.71407722	2.6736E-82	1.1037E-80
249	SLC7A11	7.71151705	1.0365E-87	4.7564E-86
250	LOC284578	7.70990554	0.00031384	0.00063981
251	CDHR3	7.70453993	1.964E-13	8.6276E-13
252	SCN2A	7.68835859	2.4883E-15	1.2289E-14
253	IGF2BP3	7.68110124	4.52E-121	3.654E-119
254	MEIS1-AS3	7.67765661	0.00031398	0.00063989
255	LINC00613	7.65984871	0.00088324	0.0017054
256	IL36G	7.65319816	1.4192E-06	3.6815E-06
257	LINC01212	7.63801977	0.00073481	0.00143153
258	KC6	7.63421589	2.8386E-12	1.1509E-11
259	DAW1	7.63262182	0.00032382	0.0006589
260	KCTD8	7.62377886	2.0154E-06	5.1567E-06
261	SCG2	7.60387985	9.7123E-06	2.3298E-05
262	MDFI	7.5866219	1.243E-132	1.149E-130
263	LOC101926889	7.5768077	0.00034175	0.00069254
264	BPIFA1	7.57296807	0.0027185	0.0049182
265	DUOX2	7.57228331	5.569E-14	2.5346E-13
266	LOC101927501	7.56117689	0.00062506	0.00122799

267	RGS17	7.47851943	2.4267E-18	1.4148E-17
268	BEST3	7.46754726	0.00050844	0.00100904
269	PEG10	7.45246122	1.5875E-78	6.1095E-77
270	SPHKAP	7.45121344	6.2644E-07	1.674E-06
271	RGAG1	7.44843035	9.9155E-61	2.5206E-59
272	ADAMTS19	7.43505399	2.452E-09	8.0294E-09
273	LIPH	7.43429742	5.3669E-55	1.1445E-53
274	GAD2	7.42873412	0.00061247	0.00120485
275	ADCY8	7.41948474	2.8547E-05	6.5408E-05
276	NEUROD1	7.41792811	0.0010323	0.00197213
277	S100A3	7.41781404	9.6638E-07	2.5461E-06
278	SLC26A7	7.4034144	3.7183E-30	3.6558E-29
279	SLC6A15	7.40044321	0.00473298	0.00825355
280	GAS2L1P2	7.39845792	0.00056816	0.00112101
281	LINC00836	7.3894798	0.00126127	0.00238386
282	SLCO6A1	7.38687933	0.00066888	0.00130957
283	ALPP	7.38151915	0.0023307	0.00425485
284	PRSS22	7.36264689	1.0475E-79	4.1216E-78
285	MMP10	7.35676102	0.00114714	0.00217815
286	SLC10A2	7.32398297	0.00064215	0.00125966
287	TMEM255A	7.32010489	7.6478E-59	1.8377E-57
288	SHISA9	7.31124156	1.2287E-48	2.1859E-47
289	ESRP1	7.30986415	8.029E-232	2.155E-229
290	SLC36A2	7.29669039	0.00068432	0.00133884
291	DNAH8	7.2829172	2.7409E-05	6.2906E-05
292	NOL4	7.27385403	4.0573E-16	2.0959E-15
293	DLL3	7.26814485	0.00116679	0.00221351
294	LOC642366	7.25121066	0.00139418	0.00261867
295	MNX1-AS1	7.24995963	0.00086097	0.00166405
296	ZNF534	7.24376925	0.00087338	0.00168704
297	ZIC5	7.23834326	2.2074E-06	5.633E-06
298	LOC101928441	7.2347993	0.00071659	0.00139873
299	PCYT1B	7.2346639	1.4251E-72	4.8731E-71
300	HOXC6	7.23228005	2.7675E-06	6.9893E-06
301	GAL3ST3	7.22654774	1.4889E-06	3.8549E-06
302	LINC01016	7.20672023	0.00216862	0.00397625
303	ERC2	7.20004631	1.5061E-06	3.8984E-06
304	MYH4	7.19546991	0.00298512	0.00537039
305	TYRP1	7.19388605	1.6215E-13	7.1687E-13
306	ADAMTS6	7.19252923	1.1101E-66	3.3459E-65
307	LOC100130673	7.18553129	0.00089772	0.00173215
308	SOHLH2	7.17779643	3.4282E-06	8.5803E-06
309	C10orf82	7.17158474	5.4768E-11	2.0337E-10
310	LCT	7.17120633	1.4046E-12	5.8133E-12
311	LINC01164	7.16649324	0.00322623	0.00577404

312	UNC5B-AS1	7.16535826	0.00100599	0.0019251
313	EPCAM	7.14172491	0	0
314	UNC5D	7.13949328	1.4027E-05	3.3157E-05
315	ITIH6	7.13877346	0.00158088	0.00295097
316	FBN3	7.12789977	6.4615E-32	6.8031E-31
317	DUSP9	7.12321237	1.872E-174	2.941E-172
318	CST5	7.12300559	0.00154962	0.00289683
319	LOC101927482	7.11121555	9.2712E-09	2.8937E-08
320	SLC4A8	7.10028795	3.586E-118	2.706E-116
321	ZNF663P	7.08310985	0.00107478	0.00204881
322	LOC101927131	7.07583987	0.00183437	0.00339431
323	FLJ42969	7.07375533	0.00146999	0.00275543
324	PTPRZ1	7.06181253	3.3546E-11	1.2673E-10
325	ART5	7.05498284	6.9336E-12	2.7422E-11
326	LOC101928569	7.05362782	0.0012462	0.00235605
327	SNORD107	7.03879173	0.00150674	0.00282099
328	PHEX	7.03080054	9.0253E-15	4.3022E-14
329	KEL	7.02855266	5.4975E-45	8.785E-44
330	IRX2	7.02848222	0.00148414	0.00278033
331	CCDC178	7.02549826	3.692E-07	1.0096E-06
332	C1QL4	7.01301631	0.00118445	0.00224547
333	SLC7A11-AS1	7.00766325	3.4368E-11	1.2971E-10
334	SPRR2F	7.00367961	0.00209858	0.00385405
335	GRIN2A	6.99762788	1.8496E-05	4.3242E-05
336	CAPN13	6.97597761	1.2675E-26	1.0932E-25
337	LMNTD1	6.96974981	0.00285145	0.00514428
338	ERVMER34-1	6.95721353	4.7841E-42	7.0449E-41
339	ZNF702P	6.9497077	7.829E-197	1.559E-194
340	HS6ST2	6.93630866	3.9896E-50	7.4186E-49
341	DDX4	6.93327664	0.00226673	0.00414434
342	ZCCHC12	6.9310911	3.8791E-06	9.6578E-06
343	SEMA3A	6.92928449	1.9357E-49	3.5315E-48
344	KLHDC8A	6.9292286	3.5096E-31	3.5857E-30
345	FSIP2	6.91802915	1.0531E-24	8.3525E-24
346	TMEM229A	6.91292354	5.5354E-10	1.9087E-09
347	CER1	6.91216774	0.00320893	0.0057468
348	ALPI	6.90782993	1.6003E-24	1.2553E-23
349	LCN15	6.90474865	5.0843E-06	1.2515E-05
350	FRAS1	6.8955494	1.476E-160	1.953E-158
351	SLC34A3	6.89342167	2.3603E-14	1.0965E-13
352	C3orf52	6.89014724	1.375E-64	3.8669E-63
353	DNAH2	6.88901974	8.5174E-36	1.033E-34
354	SERPINB7	6.88390563	0.01445383	0.02333288
355	MED12L	6.87883737	6.5949E-85	2.8761E-83
356	PHF21B	6.8701374	1.4392E-05	3.398E-05

357	SYNPR-AS1	6.86362102	0.00181826	0.00336804
358	TFCP2L1	6.85782151	2.092E-198	4.3E-196
359	CACNA1B	6.85720855	1.4474E-05	3.416E-05
360	MGC45800	6.8562175	2.2095E-10	7.8555E-10
361	LMO1	6.83785809	0.00351611	0.00625979
362	GRIP1	6.83412003	3.8022E-54	7.9328E-53
363	GUCA1A	6.83301375	5.1774E-06	1.2737E-05
364	LY6H	6.83292924	0.00593997	0.01019366
365	LGALS2	6.82755308	1.219E-127	1.076E-125
366	STK39	6.82445324	2.94E-161	3.917E-159
367	FBN2	6.80621505	3.327E-138	3.247E-136
368	LINC00475	6.79269837	0.0021293	0.00390712
369	SLC22A8	6.78977411	0.00325676	0.0058249
370	LOC440910	6.78724175	0.00202795	0.00373285
371	LRRC37A11P	6.77849569	2.4022E-05	5.5442E-05
372	CATSPERG	6.7779079	5.6088E-63	1.5241E-61
373	POM121L2	6.77682757	0.00203624	0.00374704
374	HOXB9	6.76877131	0.00391154	0.00691156
375	NYX	6.767875	0.00412327	0.00726243
376	C1QL2	6.76677967	0.00218149	0.00399832
377	ABCC12	6.76314356	0.00245046	0.00446294
378	LOC101927630	6.74927169	2.5324E-10	8.9593E-10
379	PRSS35	6.73908288	3.4796E-51	6.736E-50
380	GLP1R	6.73895645	3.8E-07	1.0381E-06
381	VCAN	6.73729088	1.7237E-74	6.033E-73
382	ALK	6.72877814	9.2225E-06	2.2159E-05
383	CAPN14	6.72634641	6.3278E-31	6.3773E-30
384	TRPV6	6.7202346	2.194E-99	1.2249E-97
385	ALOX12B	6.71524523	3.5909E-09	1.1601E-08
386	SLC22A2	6.7087116	6.1929E-06	1.5126E-05
387	GRAMD2	6.68853904	1.5292E-13	6.7669E-13
388	NXPH2	6.68590958	2.954E-12	1.1965E-11
389	SORCS3	6.6797237	1.1196E-09	3.7669E-09
390	C18orf63	6.66962488	0.00383825	0.00678888
391	PGC	6.66034015	1.3215E-07	3.7563E-07
392	RAB25	6.64947828	1.403E-248	4.169E-246
393	AVP	6.64726667	0.0065509	0.01117854
394	RHOXF1	6.63672266	0.00341477	0.0060878
395	PHEX-AS1	6.63110914	0.00288775	0.00520588
396	LINC01517	6.62335418	0.00411935	0.00725618
397	NR2E3	6.62149463	4.5926E-08	1.3557E-07
398	NPNT	6.61200632	3.602E-100	2.0289E-98
399	SPINK13	6.60890411	0.00322546	0.00577319
400	CDH6	6.60806273	5.5981E-97	3.0127E-95
401	OR2A12	6.60708397	0.00455166	0.00795963

402	NPPB	6.6069573	5.3762E-59	1.2967E-57
403	MUM1L1	6.60277917	2.584E-103	1.527E-101
404	CPA2	6.60196142	1.7887E-13	7.8845E-13
405	L1TD1	6.60022009	2.6032E-16	1.3564E-15
406	CEACAM18	6.59448319	0.00330749	0.00590906
407	DPYSL3	6.58390383	0	0
408	PRKCG	6.58249964	0.00287083	0.0051773
409	DPPA3	6.57881798	0.00441907	0.00774178
410	KIAA0087	6.5654442	0.00376666	0.0066739
411	LOC101927196	6.55386563	0.00321507	0.0057562
412	AGPAT4-IT1	6.5529604	1.5837E-05	3.7258E-05
413	IL11	6.54502365	6.5614E-12	2.5998E-11
414	MAGEC2	6.54410778	0.0038628	0.00683044
415	RMST	6.54093175	0.00291709	0.00525583
416	SYCP2L	6.54047271	3.0378E-09	9.8771E-09
417	AMBN	6.53076656	5.3138E-05	0.00011821
418	SLC15A2	6.53068826	4.8088E-10	1.6656E-09
419	RBP2	6.52951111	2.8975E-23	2.1391E-22
420	DUOX1	6.5281376	5.1654E-41	7.3704E-40
421	LOC643711	6.52673362	0.00389307	0.00688017
422	FMN2	6.52055458	1.416E-08	4.3549E-08
423	GGTLC2	6.51847556	0.00940986	0.01565608
424	FAT3	6.51831524	9.0012E-39	1.201E-37
425	SCGB1D1	6.51235351	0.0135697	0.02199938
426	AACSP1	6.51205491	1.552E-05	3.6531E-05
427	HTR1B	6.4999343	0.00046771	0.00093176
428	KRTAP5-1	6.48818284	0.00673372	0.01147126
429	IL1A	6.48810076	7.293E-16	3.7187E-15
430	LOC100288748	6.47708939	1.6533E-05	3.8839E-05
431	RGS9BP	6.47605955	3.0578E-14	1.412E-13
432	LDLRAD1	6.47396474	2.3988E-08	7.2403E-08
433	ADAMTS19-AS1	6.47143067	0.01237156	0.02018737
434	FAM26D	6.46795761	0.0035829	0.00637036
435	BFSP2	6.46206645	0.00423437	0.00744385
436	SBK1	6.45695047	2.8592E-98	1.5783E-96
437	PLET1	6.45625072	0.00541124	0.00934524
438	GJA10	6.4528113	0.00799908	0.01347126
439	GPRIN2	6.45099958	1.7985E-66	5.3706E-65
440	DYNAP	6.43855794	0.01011526	0.01674035
441	KCNJ6	6.43433053	1.1635E-07	3.3189E-07
442	RLN3	6.42036167	0.00737355	0.01249075
443	PAPL	6.41095278	0.00438765	0.00769372
444	PTPN13	6.40833083	2.902E-295	1.438E-292
445	LINC01468	6.39779361	0.01017367	0.01682549
446	MOB3B	6.39768481	1.153E-238	3.229E-236

447	TFAP2C	6.39481441	2.4297E-05	5.6063E-05
448	SNORD116-21	6.37882647	0.00507173	0.00880138
449	SERPINB13	6.37118242	1.553E-07	4.382E-07
450	LINC01208	6.35601196	0.00613449	0.01050882
451	NBPF18P	6.35561379	0.00478643	0.00833699
452	SLC1A3	6.35508827	4.855E-262	1.646E-259
453	PSG1	6.35316444	0.00923333	0.0153849
454	ASIC2	6.35117708	4.0401E-05	9.1153E-05
455	DPPA2	6.3479892	0.00922255	0.0153696
456	CHI3L2	6.34339035	1.42E-19	8.8013E-19
457	C12orf36	6.34057312	2.6323E-05	6.0529E-05
458	PKP3	6.3366273	4.194E-163	6.002E-161
459	TBX4	6.33544118	9.5951E-45	1.517E-43
460	DDIT4L	6.33199762	4.1231E-34	4.683E-33
461	FAM189A1	6.32814479	5.1567E-14	2.3508E-13
462	RNF224	6.32187633	1.6319E-12	6.7325E-12
463	LOC100128076	6.30426016	0.01336553	0.02169569
464	SOX4	6.3032615	3.609E-157	4.469E-155
465	CLPSL2	6.2962669	0.01237146	0.02018737
466	CA4	6.29255962	4.2348E-39	5.6856E-38
467	CACNB4	6.29148694	1.002E-28	9.4115E-28
468	TOPAZ1	6.28197007	0.00694016	0.01179796
469	CLVS2	6.27874807	0.01041476	0.01719771
470	LHX1	6.27796813	0.01008658	0.01669574
471	LINC01411	6.27377376	0.00814603	0.01369963
472	WNT8B	6.26652113	0.01533665	0.02465092
473	CLEC2A	6.25426499	0.02057443	0.03235111
474	LOC100506178	6.24962962	4.8906E-08	1.4405E-07
475	LHFPL3	6.24597622	6.0593E-05	0.00013403
476	NTF4	6.2401269	2.0505E-08	6.22E-08
477	CTNND2	6.23718799	2.4257E-42	3.605E-41
478	SPINT1	6.23295426	8.045E-226	1.993E-223
479	PLSCR5	6.23258212	0.02462475	0.03816688
480	DSCAM	6.22623764	7.7406E-15	3.7118E-14
481	LINC01284	6.2240941	0.01004152	0.01662686
482	PNMA5	6.22093084	7.2674E-05	0.0001593
483	HAND1	6.21958522	3.0241E-12	1.2233E-11
484	LINC01122	6.21420464	0.00637392	0.01089289
485	DLX6	6.21370421	0.0074384	0.01259178
486	GUCY2C	6.21296248	2.8172E-37	3.5903E-36
487	EFNA5	6.20937088	1.261E-121	1.033E-119
488	C1orf106	6.2029804	4.419E-163	6.277E-161
489	ZDHHC8P1	6.18816749	2.9775E-17	1.6366E-16
490	VANGL2	6.17543171	1.955E-110	1.293E-108
491	AIM1L	6.17337857	6.7577E-51	1.2939E-49

492	SNORD64	6.16937192	0.00663561	0.0113121
493	LINC00488	6.16480713	0.02294619	0.03577472
494	TCF24	6.15610064	0.01423998	0.02301244
495	LRRC1	6.15361754	3.391E-224	8.189E-222
496	ANXA3	6.14367146	3.755E-203	7.972E-201
497	TLX2	6.1393028	0.00692546	0.01177816
498	ZNF695	6.13742598	1.9318E-08	5.8719E-08
499	CKB	6.12781821	2.982E-146	3.216E-144
500	TRIM17	6.12105386	3.8604E-14	1.7745E-13
501	FLJ22184	6.115602	1.8225E-15	9.0758E-15
502	CCDC144NL	6.1150463	0.01037188	0.01713128
503	KLRF2	6.11319469	0.01445945	0.02333806
504	CCDC169-SOHLH2	6.11158447	9.2777E-10	3.1424E-09
505	KLK13	6.10416236	1.0238E-10	3.7329E-10
506	CDH12	6.10386879	2.2716E-22	1.6041E-21
507	LOC101927069	6.10166763	1.817E-41	2.6295E-40
508	LOC339260	6.10056306	0.00010311	0.00022228
509	NFKBIL1	6.0987551	0.00834009	0.01400166
510	FOXL2	6.09615448	0.00692413	0.01177693
511	TRIM39-RPP21	6.09243042	0.01215551	0.0198684
512	LINC00458	6.0876516	0.00930418	0.01549226
513	MSX2	6.08747732	1.7732E-45	2.8693E-44
514	ZNF280A	6.08745996	0.01163627	0.01906971
515	CCDC144CP	6.08629313	0.01084598	0.01785179
516	HAPLN1	6.08050798	5.36E-11	1.9918E-10
517	LANCL3	6.07965402	0.00013031	0.00027794
518	NR2E1	6.07409742	0.01560687	0.02504774
519	THSD7B	6.06915745	3.5531E-26	2.9989E-25
520	LHFPL3-AS1	6.06873231	0.01310502	0.02130683
521	C6orf147	6.06623227	8.1853E-05	0.00017855
522	UTS2R	6.06408745	0.00012607	0.00026935
523	DLX4	6.06322797	7.9538E-12	3.1342E-11
524	CLCNKA	6.06116009	5.8227E-07	1.5616E-06
525	AQP10	6.05671649	2.0031E-08	6.0801E-08
526	LOC101927735	6.05031484	0.00918181	0.01530698
527	ATP1A4	6.04728229	3.2881E-13	1.4212E-12
528	C12orf75	6.04304309	5.866E-230	1.511E-227
529	ATP13A4	6.04187522	3.0292E-65	8.7351E-64
530	LYPD6B	6.04053241	6.8639E-20	4.3042E-19
531	FAM83B	6.03329137	6.729E-79	2.6105E-77
532	SMTNL2	6.0332762	4.3877E-18	2.517E-17
533	SP9	6.03192856	0.00725078	0.01229504
534	PIP5K1B	6.02930153	2.483E-99	1.3827E-97
535	ALOXE3	6.02672177	7.4801E-05	0.00016366

536	TRH	6.02127174	0.01839689	0.02918607
537	KIF26B	6.01777325	4.095E-115	2.909E-113
538	MED15P9	6.01137213	0.0088203	0.01474119
539	TFF1	6.00896307	5.8839E-16	3.0145E-15
540	MCOLN3	6.00806848	1.3485E-38	1.7893E-37
541	PLA2G10	6.00403163	1.6949E-14	7.9462E-14
542	LOC100507600	5.9946686	0.02268161	0.03540508
543	PDE6C	5.99424438	1.0355E-10	3.7705E-10
544	C1orf195	5.99162088	0.01973423	0.03114658
545	GABRQ	5.98937612	0.01057073	0.01743588
546	PCSK5	5.98728542	4.577E-230	1.195E-227
547	GPRC5A	5.98459968	1.0802E-61	2.8279E-60
548	BIN3-IT1	5.97880327	0.00807484	0.01359055
549	LINC00379	5.97525188	0.01103788	0.01814776
550	GLYATL2	5.97517296	8.6736E-05	0.00018869
551	FLRT2	5.97358116	5.553E-198	1.129E-195
552	INSM1	5.97109329	0.03094803	0.04714682
553	PDZD3	5.95688947	2.5882E-23	1.9174E-22
554	GGNBP1	5.95322392	0.01122861	0.01842977
555	NRIP3	5.95109506	6.1088E-52	1.2105E-50
556	B4GALT6	5.94808979	1.0321E-93	5.1792E-92
557	LOC100505817	5.94728984	0.00026425	0.00054301
558	ADAM18	5.94413699	0.01712539	0.02728538
559	LINC00673	5.94142273	9.9071E-33	1.0729E-31
560	ARHGDIG	5.94137381	7.4696E-10	2.5448E-09
561	TMEM200A	5.93835091	3.8309E-55	8.2236E-54
562	ZNF90	5.93799361	5.171E-32	5.4593E-31
563	SYT14	5.93538657	0.02194795	0.03432365
564	ECEL1P2	5.93426662	0.00033503	0.00068014
565	SYT3	5.93227838	0.00026396	0.00054247
566	MYBPC3	5.93129324	7.8E-12	3.0748E-11
567	KLK8	5.93071039	0.00016528	0.00034781
568	PAGE4	5.92615314	0.01864091	0.02954894
569	GALNT13	5.92213286	1.0123E-07	2.9018E-07
570	RBP3	5.91766719	0.00014928	0.00031592
571	SRY	5.91216386	9.1297E-05	0.00019805
572	ZNF365	5.9112312	0.00014004	0.00029741
573	GABRR3	5.90959376	0.01183626	0.01937445
574	DHX16	5.90704624	0.01047807	0.01729043
575	ANKRD1	5.90399653	1.8439E-94	9.3746E-93
576	LOC400706	5.89971929	0.02885025	0.04417044
577	INA	5.89957492	3.1506E-10	1.1071E-09
578	LGI2	5.88254675	1.6207E-42	2.4198E-41
579	LUZP2	5.88104252	1.6328E-07	4.5972E-07
580	NANOS3	5.87881019	0.0270029	0.04156609

581	PCDHB2	5.87144063	2.8717E-41	4.1435E-40
582	OR13A1	5.86561413	0.01975278	0.03117077
583	LINC00086	5.85692671	2.2859E-89	1.0693E-87
584	HKDC1	5.85294329	1.4705E-84	6.3845E-83
585	KIF1A	5.85274736	5.8717E-28	5.3485E-27
586	DPPA4	5.83700586	3.0857E-25	2.5028E-24
587	AP1M2	5.83316829	6.998E-172	1.082E-169
588	NOX4	5.83015981	4.2954E-33	4.7045E-32
589	LOC101927416	5.82465193	0.02379413	0.03698331
590	MSTN	5.82383886	0.01262819	0.02057313
591	DQX1	5.80322583	2.894E-13	1.2553E-12
592	CLEC2L	5.79872386	0.01416137	0.02289329
593	KRT79	5.79788935	0.01705435	0.02718341
594	KLHL41	5.79678316	7.2646E-33	7.8938E-32
595	LOC101929412	5.79665056	0.01328044	0.02157025
596	PCDH10	5.79148787	3.0094E-50	5.6229E-49
597	VGF	5.79063736	0.01876293	0.02972772
598	GJA1	5.78825264	3.437E-157	4.284E-155
599	FILIP1L	5.78172674	0	0
600	OR10V2P	5.77073562	0.01420014	0.02295404
601	LINC00176	5.76438561	2.8456E-06	7.178E-06
602	TMPRSS7	5.75343209	0.03044151	0.04644109
603	LOC100131047	5.75198284	0.01706853	0.02720377
604	ANKRD63	5.74999371	0.01828113	0.02901433
605	RPRM	5.74357244	1.4934E-07	4.2239E-07
606	LAMC2	5.74282417	2.589E-138	2.539E-136
607	PLEKHG4B	5.7394825	1.6645E-08	5.0907E-08
608	B4GALNT4	5.73866734	3.0023E-21	2.0211E-20
609	SCIN	5.73465302	1.782E-21	1.2093E-20
610	ROCK1P1	5.73269837	5.8953E-17	3.1797E-16
611	MAGEB17	5.73209974	0.00023252	0.00048036
612	SNCA-AS1	5.72927989	0.01810495	0.02875124
613	UPK3A	5.72882006	2.764E-18	1.6056E-17
614	LOC388882	5.72552145	0.00024785	0.0005106
615	PSG3	5.70892503	0.01753388	0.02790171
616	MGAT4EP	5.70833067	0.01322375	0.02148396
617	SPACA6P-AS	5.70654958	0.00030463	0.00062181
618	LINC00494	5.69980047	0.0002598	0.00053436
619	DCC	5.69372081	4.0666E-16	2.1002E-15
620	LOC440173	5.69272611	0.00067338	0.00131811
621	RHO	5.69077334	0.01810103	0.02874739
622	COX7B2	5.68994534	0.01535509	0.02467851
623	BRSK1	5.68189876	2.6254E-61	6.7994E-60
624	ARGFXP2	5.68121226	0.02366552	0.03680397
625	FRZB	5.680632	1.8883E-09	6.2417E-09

626	UCN3	5.67977082	0.01570757	0.02520098
627	OR2A14	5.67953055	0.0158501	0.02540697
628	SNORD116-24	5.67883052	0.01952716	0.03084496
629	HTR2C	5.67844961	0.02946026	0.04504277
630	LOC100507388	5.67749018	0.02140448	0.03355795
631	CALCR	5.67405244	2.1056E-07	5.877E-07
632	CES5AP1	5.66574739	0.01487973	0.02395837
633	SLC7A7	5.65441035	4.827E-192	9.054E-190
634	TMEM132D	5.64923968	6.1463E-17	3.3123E-16
635	MSI1	5.64583439	6.16E-58	1.4408E-56
636	SEMA3C	5.64569194	8.229E-163	1.16E-160
637	LOC101927391	5.64341806	2.9939E-17	1.6442E-16
638	CTSE	5.64307353	1.6841E-12	6.9373E-12
639	PARM1	5.64217929	3.844E-131	3.47E-129
640	PWAR1	5.63958913	0.02046298	0.03220207
641	C6orf132	5.63462853	4.807E-119	3.657E-117
642	CUBN	5.63280548	3.2181E-16	1.67E-15
643	PRIMA1	5.62488873	1.2842E-41	1.8682E-40
644	PCDH7	5.61569165	1.873E-139	1.875E-137
645	SLCO5A1	5.61568305	1.3773E-11	5.3454E-11
646	ARID3A	5.61305381	3.13E-296	1.635E-293
647	XIST	5.60121646	0.00134712	0.00253608
648	LOC101928372	5.60026578	3.2854E-08	9.821E-08
649	CFTR	5.59330896	1.9275E-54	4.0565E-53
650	RPLP0P2	5.59321991	3.2632E-23	2.4008E-22
651	DRD5	5.59316282	0.00045738	0.00091231
652	F3	5.58617721	1.7162E-30	1.7083E-29
653	BRSK2	5.58183014	6.7482E-13	2.8541E-12
654	CRABP2	5.57908299	3.5542E-47	6.0079E-46
655	EDARADD	5.57821922	1.994E-104	1.207E-102
656	TAS2R3	5.57640284	0.02597071	0.04007621
657	LOC100129550	5.56916322	1.4092E-54	2.9722E-53
658	GALNT12	5.56596508	6.4518E-57	1.4613E-55
659	TMEM52B	5.56385252	0.00073115	0.00142513
660	MARK2P9	5.56382298	0.02725126	0.04191501
661	MMP11	5.56054576	1.23E-85	5.4883E-84
662	GPR78	5.55250659	0.00139171	0.00261478
663	PRSS12	5.54988387	3.0185E-25	2.4493E-24
664	MMP1	5.5457579	1.83E-12	7.5095E-12
665	DUOXA2	5.54270017	4.5591E-12	1.8263E-11
666	KCNV1	5.54191315	0.02503942	0.03874432
667	LSAMP	5.53853505	3.8681E-34	4.3986E-33
668	STK32A	5.53624688	2.9938E-44	4.6683E-43
669	TMEM125	5.5300734	5.0188E-65	1.4365E-63
670	ISX	5.52380068	4.6885E-16	2.4142E-15

671	INHA	5.52327803	3.0652E-13	1.3284E-12
672	C1orf116	5.51816123	8.422E-108	5.352E-106
673	PTCHD1-AS	5.51521524	0.02210828	0.0345604
674	C1orf110	5.50676085	0.02361475	0.03673406
675	FOXJ1	5.49702386	9.9259E-13	4.1562E-12
676	SLC2A5	5.48470489	2.7338E-35	3.2463E-34
677	GPR87	5.48298613	4.9267E-12	1.9695E-11
678	SALL4	5.48081331	1.5606E-71	5.2255E-70
679	MAGEL2	5.48011697	1.9183E-26	1.637E-25
680	SLC9C1	5.47937493	0.00033144	0.00067362
681	DUOXA1	5.47593084	1.0804E-18	6.399E-18
682	ITLN2	5.47572068	1.6011E-21	1.0896E-20
683	DPP10	5.473591	4.8591E-30	4.7606E-29
684	JPH3	5.46957711	8.4047E-19	4.9963E-18
685	TRPM6	5.46873878	1.1027E-26	9.5279E-26
686	ZNF714	5.46703112	8.6526E-75	3.0505E-73
687	LOC728739	5.45528306	0.02276215	0.03551072
688	REEP1	5.45290797	1.16E-150	1.319E-148
689	DLX6-AS1	5.44890782	0.0235637	0.0366576
690	GNAT3	5.44478828	0.02502287	0.03872181
691	HPDL	5.44434527	2.5148E-30	2.4864E-29
692	ARHGAP8	5.44346492	4.637E-137	4.479E-135
693	DDN	5.44068909	0.0006844	0.00133886
694	ENTPD3	5.43758013	4.726E-14	2.1596E-13
695	PENK	5.43261875	0.00130901	0.00246828
696	UCA1	5.43015729	2.347E-06	5.9717E-06
697	LMTK3	5.4217965	3.1973E-53	6.5436E-52
698	CACHD1	5.42105155	3.555E-146	3.795E-144
699	DCAF8L2	5.42083899	0.03084961	0.04700429
700	RPL13AP3	5.4200647	0.03268161	0.04956112
701	LINC00890	5.41433011	1.8629E-27	1.6616E-26
702	SLC38A5	5.41209915	8.9463E-50	1.6445E-48
703	COL22A1	5.41149454	1.8831E-10	6.7263E-10
704	PTPRN	5.40641767	7.7164E-11	2.8412E-10
705	CHRNA1	5.4061703	0.00131014	0.00246994
706	KLK10	5.40001092	1.4887E-16	7.8518E-16
707	ARHGAP28	5.39567844	1.945E-92	9.513E-91
708	CERS1	5.39523151	3.21E-13	1.3896E-12
709	NEFM	5.38118628	4.0945E-07	1.1135E-06
710	PROM1	5.37281614	9.0386E-83	3.7635E-81
711	HEPH	5.37194028	8.5171E-34	9.5392E-33
712	USH1C	5.36843573	1.6598E-26	1.4202E-25
713	NAALAD2	5.362379	2.5107E-76	9.1869E-75
714	FLRT3	5.36074626	1.7568E-72	5.9862E-71
715	CCDC169	5.35446302	1.5975E-05	3.7569E-05

716	C1orf145	5.34340174	6.0284E-08	1.7615E-07
717	GUCY2D	5.34310188	2.7759E-13	1.2049E-12
718	HAPLN3	5.34121135	9.459E-43	1.4177E-41
719	SLC39A2	5.34119565	7.4966E-09	2.357E-08
720	NWD2	5.34113932	0.01001013	0.01658199
721	DCAF12L2	5.34110749	2.1876E-05	5.0713E-05
722	KRT19	5.33742572	9.462E-163	1.325E-160
723	KCNJ16	5.33704423	7.6976E-41	1.0895E-39
724	DLGAP3	5.33080026	2.0337E-20	1.3127E-19
725	MYBPH	5.32883879	0.00082404	0.00159652
726	SLC7A3	5.32707452	0.0012797	0.00241656
727	SMPDL3B	5.32475098	8.2253E-61	2.0992E-59
728	CASQ1	5.3218076	4.262E-13	1.8233E-12
729	OR8G5	5.31967817	0.0302374	0.04615522
730	TNIP3	5.31957259	3.1218E-07	8.5756E-07
731	OSR2	5.31899839	1.4875E-26	1.2767E-25
732	LOC441204	5.31259388	8.9336E-27	7.7294E-26
733	SH2D5	5.30739252	0.000809	0.00156864
734	LOC100287225	5.30588289	0.02665287	0.04106328
735	PABPC4L	5.3043979	1.1517E-75	4.1359E-74
736	CD200	5.30376179	2.9764E-35	3.5236E-34
737	SSTR5-AS1	5.30104744	0.00075276	0.00146458
738	CLIC6	5.29855248	4.4601E-71	1.4755E-69
739	ZNF711	5.29771885	1.2809E-89	6.0359E-88
740	MAP9	5.29654902	3.72E-144	3.884E-142
741	MYEF2	5.29439852	2.2423E-34	2.5817E-33
742	CLCNKB	5.28995111	2.3826E-06	6.056E-06
743	PRSS46	5.2893717	2.3698E-05	5.4726E-05
744	DRP2	5.28540413	0.00091547	0.00176305
745	HCAR3	5.28536163	9.3806E-22	6.468E-21
746	OLR1	5.28337781	5.277E-30	5.1647E-29
747	KRT6A	5.28262813	7.5268E-16	3.8348E-15
748	MIXL1	5.26774876	0.00083198	0.00161048
749	LINC01330	5.25861204	0.00649217	0.01108615
750	SPINT2	5.25585711	0	0
751	GIPR	5.24958018	3.033E-28	2.8023E-27
752	LAMA3	5.24826713	5.2895E-49	9.4974E-48
753	SLITRK4	5.24622293	5.2484E-11	1.9522E-10
754	GRAMD1B	5.23931471	6.977E-113	4.763E-111
755	CD109	5.23806551	1.091E-107	6.886E-106
756	MYO18B	5.23269023	3.0202E-23	2.2254E-22
757	CAPN6	5.22691372	2.435E-19	1.4949E-18
758	GAD1	5.22163123	2.9303E-06	7.383E-06
759	APLP1	5.21744053	1.132E-54	2.3929E-53
760	DRAXIN	5.21337548	0.0013246	0.00249622

761	PGM2L1	5.21260975	5.3127E-75	1.8799E-73
762	TMEFF1	5.21128864	3.23E-40	4.4926E-39
763	FER1L6	5.21055797	0.00181365	0.0033608
764	SPECC1	5.21029558	3.618E-156	4.396E-154
765	DLX3	5.20623007	0.00132216	0.00249186
766	PLA2G1B	5.19807297	1.586E-17	8.8378E-17
767	ARL14	5.1965029	8.3826E-06	2.0221E-05
768	AGPAT1	5.1935913	1.3497E-08	4.1589E-08
769	FGF9	5.19342184	3.704E-08	1.1023E-07
770	SOSTDC1	5.1924026	2.6858E-09	8.7697E-09
771	LINC00648	5.18653754	1.3338E-07	3.789E-07
772	SRRM4	5.18577291	0.0010266	0.00196181
773	B3GALT1	5.17477941	1.2928E-14	6.1156E-14
774	VWA2	5.16881578	3.5172E-18	2.0309E-17
775	NODAL	5.16569046	1.7445E-05	4.0883E-05
776	RAB3B	5.16469049	4.019E-249	1.233E-246
777	LEF1-AS1	5.15136424	0.00114651	0.00217717
778	CPE	5.15021871	7.2563E-60	1.8066E-58
779	PPP1R14C	5.14646393	1.2947E-11	5.0402E-11
780	GPR119	5.14178718	1.6382E-09	5.4455E-09
781	PP14571	5.13688117	0.00150687	0.00282099
782	EDN3	5.13678025	0.00077879	0.00151217
783	IGSF3	5.13472749	3.142E-266	1.124E-263
784	KCNH6	5.13067242	0.00229985	0.00420015
785	HTR1D	5.12998434	1.402E-05	3.3145E-05
786	NKAIN1	5.1170295	0.00155811	0.00291156
787	LINC00842	5.11224841	1.5746E-21	1.0719E-20
788	RBBP8NL	5.11151604	3.6378E-12	1.4639E-11
789	BCAT1	5.10739968	1.4132E-32	1.5185E-31
790	FLVCR1-AS1	5.1055125	1.5215E-40	2.1347E-39
791	AGR3	5.09508008	1.0829E-07	3.095E-07
792	PRDM1	5.09130529	7.026E-196	1.371E-193
793	PRRT2	5.09112789	1.0777E-12	4.5039E-12
794	LHFPL3-AS2	5.09106161	1.8947E-14	8.8548E-14
795	YBX2	5.07371804	1.3553E-28	1.2644E-27
796	MACC1	5.06942999	2.5497E-26	2.1615E-25
797	MYCN	5.06186705	3.6852E-40	5.1038E-39
798	RAB11FIP4	5.06112457	9.744E-235	2.652E-232
799	FUT8-AS1	5.05733618	6.0308E-23	4.3803E-22
800	DSC3	5.05619636	3.9992E-07	1.0895E-06
801	PCED1B	5.05032959	7.9807E-44	1.2276E-42
802	TMEM190	5.04917421	1.0594E-05	2.5303E-05
803	KCP	5.0439456	4.7059E-08	1.3876E-07
804	RIMS1	5.04342569	9.9797E-05	0.00021545
805	MLLT11	5.0429031	5.378E-168	7.992E-166

806	CTB-113P19.1	5.03649861	5.4169E-38	7.0569E-37
807	PLA2G4E	5.0326112	0.00139363	0.00261788
808	FREM2	5.0233022	1.1869E-52	2.3885E-51
809	GDF1	5.02200197	5.1537E-10	1.7815E-09
810	CEACAM19	5.01808377	2.8265E-60	7.1383E-59
811	DMKN	5.00837971	1.1717E-80	4.677E-79
812	TIGD3	5.00768245	1.5432E-22	1.0989E-21
813	FAM131B	5.00011244	1.3162E-27	1.1822E-26
814	NBPF7	4.99798093	0.00206103	0.00379048
815	MFGE8	4.99264389	1.3489E-68	4.2307E-67
816	CASC18	4.99184621	8.5223E-06	2.0542E-05
817	LGALS3	4.99102328	3.2072E-10	1.1258E-09
818	SPATA22	4.98653872	0.00449149	0.00786101
819	ATP13A5	4.98642075	0.0015822	0.00295316
820	CALB2	4.98354497	1.9605E-05	4.5678E-05
821	ANKRD29	4.98344995	2.4737E-47	4.2145E-46
822	KLF5	4.98312283	6.9151E-44	1.0671E-42
823	CST1	4.98212247	1.1901E-06	3.1084E-06
824	ACTA1	4.98149039	1.2189E-15	6.1388E-15
825	LINC00941	4.97925524	8.564E-13	3.5953E-12
826	PITX1	4.97776967	1.9082E-39	2.5926E-38
827	SLC6A6	4.95842597	5.023E-136	4.805E-134
828	DPYSL5	4.9563385	5.9983E-05	0.00013278
829	ZNF486	4.94754646	2.0876E-57	4.7844E-56
830	LRRN1	4.94313751	2.8874E-44	4.5096E-43
831	TNFRSF19	4.9420008	1.2389E-77	4.6931E-76
832	UGT2B11	4.9402349	1.6041E-32	1.7198E-31
833	CGB7	4.93397263	0.00511816	0.0088732
834	GUCA2A	4.92560377	1.4117E-07	4.0016E-07
835	PTK7	4.92545279	2.996E-146	3.216E-144
836	PLCD3	4.91732508	4.7132E-58	1.1064E-56
837	RAB27B	4.91679198	5.199E-148	5.806E-146
838	GPR64	4.91545568	5.0954E-19	3.0668E-18
839	CCR6	4.90827953	2.2515E-24	1.7526E-23
840	IQCA1	4.90241979	1.4403E-41	2.0937E-40
841	LOC285696	4.89793555	7.5869E-08	2.1953E-07
842	COL18A1-AS1	4.89456845	2.4418E-05	5.6321E-05
843	PRR15L	4.89263173	2.847E-114	2E-112
844	NME8	4.89248752	0.00541538	0.00935072
845	GXYLT2	4.89035268	3.7578E-14	1.7278E-13
846	MURC	4.88325855	1.3491E-18	7.9634E-18
847	IHH	4.88145036	8.3163E-43	1.2484E-41
848	ITPR3	4.87639447	1.58E-295	8.034E-293
849	ANKDD1B	4.87008686	1.6159E-23	1.2054E-22
850	LOC100130899	4.863908	6.3366E-21	4.1983E-20

851	TGFB2	4.86143558	7.0965E-42	1.041E-40
852	LOC100131315	4.85843249	0.0027786	0.00501893
853	CAMKV	4.85661643	0.01020646	0.01687393
854	SLC2A3	4.85136826	3.802E-151	4.399E-149
855	NNAT	4.84738669	1.3055E-65	3.8099E-64
856	EEF1A2	4.84350191	2.8892E-54	6.0541E-53
857	ANKRD33B	4.84089252	4.7196E-53	9.6084E-52
858	ZNF93	4.83697856	3.577E-39	4.8192E-38
859	KCNE1L	4.83655473	7.7122E-12	3.0408E-11
860	KLHL13	4.83400674	1.66E-168	2.486E-166
861	CFL1P1	4.83317145	0.00290238	0.0052303
862	FSCN2	4.83218419	8.3367E-09	2.6096E-08
863	C19orf33	4.82706714	1.3883E-26	1.1937E-25
864	MKRN2OS	4.82491548	8.1577E-15	3.9002E-14
865	POT1-AS1	4.82065176	8.5114E-18	4.8181E-17
866	LOC100128770	4.81751396	4.8532E-09	1.5509E-08
867	GATA5	4.81650876	8.062E-101	4.568E-99
868	GPR89B	4.80844644	0.00478713	0.00833745
869	KCNK10	4.80398687	1.0506E-06	2.7605E-06
870	PTHLH	4.79976512	0.00066059	0.00129399
871	FANK1	4.7979233	1.7262E-10	6.1804E-10
872	KRT8P41	4.79366615	0.00310099	0.00556589
873	CPA6	4.79222402	0.00604866	0.0103719
874	GALNT5	4.78823438	1.2731E-09	4.2636E-09
875	NDNF	4.78557653	1.6059E-26	1.3746E-25
876	P2RY6	4.78108385	9.951E-122	8.181E-120
877	LGI4	4.78044039	1.205E-111	8.137E-110
878	ZNF204P	4.77958004	1.308E-77	4.9455E-76
879	PWRN1	4.77185874	0.00296203	0.0053338
880	SLC16A12	4.76868032	3.5416E-13	1.5259E-12
881	CREG2	4.76143503	0.00288203	0.00519604
882	GCNT3	4.7572858	7.264E-42	1.0648E-40
883	IGSF11	4.75574645	3.6965E-09	1.1927E-08
884	SH2D7	4.75571189	4.0084E-07	1.0915E-06
885	P2RY4	4.7547152	4.288E-07	1.165E-06
886	PCDHB3	4.75462836	3.8506E-41	5.5228E-40
887	FAM163A	4.75288527	5.6447E-05	0.00012524
888	INPP5F	4.75061768	0	0
889	LOC100132287	4.747033	0.00542427	0.00936439
890	LOC100132062	4.747033	0.00542427	0.00936439
891	LINC00930	4.74007786	1.0962E-06	2.8752E-06
892	TAC4	4.73528368	0.00021501	0.00044605
893	AKAP5	4.72819485	1.1269E-58	2.6944E-57
894	TMPRSS13	4.72694665	1.2085E-16	6.4163E-16
895	LOC101929555	4.72028977	8.6717E-07	2.2919E-06

896	TEKT2	4.71898309	0.00379001	0.00671032
897	TGFB2-OT1	4.71822925	4.1644E-20	2.644E-19
898	ZNF280B	4.7161964	6.7239E-27	5.8675E-26
899	DBN1	4.71552461	8.544E-188	1.514E-185
900	CTXN1	4.7149524	3.1033E-16	1.6113E-15
901	EFNB2	4.71069481	4.1921E-21	2.7967E-20
902	PAPPA-AS1	4.70739955	4.8229E-09	1.5417E-08
903	TMSB15B	4.69794202	6.1469E-05	0.00013588
904	STC2	4.69180571	4.6228E-41	6.606E-40
905	SLC26A2	4.69018114	3.87E-140	3.935E-138
906	CPXM1	4.6856343	7.6397E-13	3.2213E-12
907	ZNF114	4.68517352	6.115E-19	3.6685E-18
908	MFAP2	4.6810843	4.1359E-39	5.5606E-38
909	COL4A5	4.68074843	2.6343E-83	1.1064E-81
910	NRADDP	4.6806762	0.00415068	0.00730671
911	FKBP1AP1	4.67595207	2.2168E-10	7.8801E-10
912	MKRN7P	4.67443835	0.00552321	0.00951988
913	SYNGR3	4.6742169	3.0524E-22	2.1484E-21
914	SLC7A6	4.67344068	1.363E-139	1.379E-137
915	PAK6	4.67329104	5.9343E-26	4.9504E-25
916	AQP4	4.67307902	3.5443E-38	4.6427E-37
917	FAM46B	4.67040063	3.9278E-79	1.5361E-77
918	CXCL5	4.66676114	1.4325E-07	4.058E-07
919	KIAA0319	4.66418256	1.0509E-13	4.6878E-13
920	SLC24A5	4.65345928	5.9403E-10	2.0421E-09
921	FA2H	4.65322939	1.5211E-17	8.484E-17
922	TRHDE	4.65313014	2.0677E-22	1.466E-21
923	KCTD16	4.65003258	0.00033298	0.00067668
924	CCDC141	4.64980056	5.7423E-35	6.7238E-34
925	CLDN4	4.64701676	1.7697E-65	5.1491E-64
926	LDHB	4.64635506	1.352E-264	4.749E-262
927	B3GNT5	4.64580728	2.8087E-58	6.6338E-57
928	MDK	4.64434642	5.1141E-29	4.8481E-28
929	HNRNPKP3	4.64354068	0.00436303	0.00765333
930	ACAN	4.64297949	4.4431E-06	1.1004E-05
931	ZNF431	4.64225854	1.7279E-98	9.5651E-97
932	CTSK	4.64153072	4.3901E-44	6.8016E-43
933	CDH8	4.64112974	0.00019123	0.00039959
934	GRHL2	4.63989123	1.1034E-47	1.9016E-46
935	TMEM132A	4.62895606	2.281E-182	3.934E-180
936	HES7	4.62713734	0.00875751	0.01464766
937	DUSP13	4.62470613	0.00011704	0.00025088
938	TSPAN2	4.62278337	7.768E-68	2.3898E-66
939	COL4A3	4.6159707	4.203E-11	1.5752E-10
940	LEFTY2	4.61345767	2.2312E-06	5.6913E-06

941	LOC100506725	4.61260417	7.2807E-17	3.903E-16
942	GSTP1	4.61095024	0	0
943	GPR160	4.60442276	4.3861E-44	6.8009E-43
944	EML6	4.60169864	1.1869E-65	3.4742E-64
945	SOAT2	4.59863255	3.4095E-59	8.2963E-58
946	ARHGAP31-AS1	4.5977227	0.0086898	0.01454448
947	ITPKA	4.5961945	6.258E-157	7.701E-155
948	ACTBL2	4.59607655	0.01518445	0.02441847
949	CREB3L1	4.59316352	3.7505E-52	7.4624E-51
950	OSBPL3	4.59302854	4.4553E-51	8.5649E-50
951	EXTL1	4.58725106	7.3311E-08	2.1244E-07
952	NKX3-2	4.58347787	5.1633E-05	0.0001151
953	GULP1	4.58026582	2.674E-117	1.979E-115
954	EPPK1	4.57054553	5.494E-105	3.348E-103
955	ATP10B	4.56861392	1.1775E-15	5.9352E-15
956	SLN	4.56265645	6.2874E-07	1.6797E-06
957	BAI2	4.55957189	1.4589E-16	7.6988E-16
958	VASH2	4.55793747	4.2545E-16	2.1954E-15
959	C15orf27	4.55477397	1.5037E-11	5.8243E-11
960	MYH15	4.55241234	1.6296E-07	4.5888E-07
961	TRNP1	4.54863833	4.7566E-36	5.8273E-35
962	CEACAM22P	4.54506644	1.7107E-06	4.4061E-06
963	C1orf210	4.54421211	1.756E-113	1.216E-111
964	LOC101928103	4.54162503	2.3238E-10	8.2468E-10
965	ILDR2	4.5389015	1.744E-108	1.123E-106
966	CDC42P3	4.53707251	0.00719025	0.0122027
967	LINC01099	4.53389204	0.00567695	0.00976745
968	WNT16	4.53344129	0.00593053	0.01017837
969	HR	4.53009451	2.6728E-09	8.7285E-09
970	TET1	4.52975349	1.621E-66	4.8554E-65
971	GRM1	4.52529471	3.9432E-05	8.9081E-05
972	NFE2L3	4.52192726	7.244E-43	1.0917E-41
973	ST6GALNAC2	4.51990709	2.351E-22	1.6583E-21
974	TUBB2B	4.51927876	4.5301E-73	1.5629E-71
975	IGF2BP2	4.51918947	4.726E-204	1.014E-201
976	PLEKHH1	4.51714329	2.6727E-58	6.328E-57
977	GDPD2	4.51050692	1.71E-08	5.2248E-08
978	GCNT4	4.50477236	2.3787E-42	3.5378E-41
979	COLQ	4.50423779	5.8219E-17	3.141E-16
980	FAM3B	4.49980909	4.3419E-46	7.1332E-45
981	LRCH2	4.49942123	2.9602E-42	4.3859E-41
982	POU2F3	4.49436896	0.00029844	0.00060989
983	SCNN1A	4.49402302	3.4796E-53	7.1138E-52
984	FIGNL2	4.4904943	1.5481E-94	7.9125E-93
985	RASGRF2	4.48757389	2.6902E-31	2.7617E-30

986	KISS1R	4.48322338	0.00011246	0.00024158
987	LINC01512	4.48237864	0.00016112	0.0003395
988	ZNF233	4.48081421	2.4592E-08	7.4192E-08
989	MGAT3	4.47341693	3.46E-28	3.1847E-27
990	IL17D	4.47181608	4.1583E-32	4.4045E-31
991	HIST3H2A	4.46779654	5.3665E-19	3.2269E-18
992	AMIGO2	4.46488472	3.4607E-20	2.2103E-19
993	SPX	4.46056941	7.5833E-23	5.4831E-22
994	KIAA1211	4.45795594	1.858E-71	6.1889E-70
995	ANO2	4.45540032	4.7899E-18	2.7452E-17
996	MALAT1	4.45489955	4.3178E-60	1.0876E-58
997	UGT3A2	4.45329902	1.116E-27	1.0052E-26
998	CELF4	4.45152707	3.4737E-10	1.2156E-09
999	OVCH2	4.44941468	4.3622E-06	1.0812E-05
1000	CCNJL	4.44776231	5.1071E-91	2.4494E-89
1001	EMX2	4.43894914	6.0421E-06	1.4767E-05
1002	C19orf26	4.43682002	4.068E-20	2.5862E-19
1003	CIB2	4.43382797	5.2748E-31	5.3496E-30
1004	PURG	4.43186411	5.0191E-05	0.00011201
1005	COL19A1	4.42781665	4.3147E-07	1.1719E-06
1006	DPP10-AS1	4.42347175	2.2908E-07	6.3717E-07
1007	RCOR2	4.42261599	5.2667E-15	2.5521E-14
1008	TMC7	4.42245056	8.0416E-38	1.0385E-36
1009	BASP1	4.4167579	7.1628E-62	1.8828E-60
1010	RASGRF1	4.41431369	0.01169858	0.01916369
1011	TMEM88	4.41360782	6.7914E-65	1.9295E-63
1012	CATIP-AS2	4.40880937	0.01721216	0.02742136
1013	NSUN7	4.40649172	5.9224E-73	2.0359E-71
1014	CCDC136	4.40291365	3.2274E-19	1.9676E-18
1015	EFR3B	4.40284013	2.873E-11	1.0922E-10
1016	MATN3	4.40181288	1.2859E-24	1.0148E-23
1017	LOC101929128	4.40088706	2.3333E-06	5.9384E-06
1018	EGFL6	4.39575964	0.00111523	0.00212132
1019	PDE5A	4.3935504	4.203E-116	3.053E-114
1020	ZNF827	4.39349771	6.4371E-93	3.1888E-91
1021	LOC101926996	4.37612863	0.01450966	0.02341128
1022	CNNM1	4.37351861	2.7904E-14	1.2914E-13
1023	CDKL5	4.37093879	1.2829E-36	1.595E-35
1024	DAB1	4.36930109	2.887E-34	3.3062E-33
1025	ANKRD30B	4.36642178	0.01546729	0.0248485
1026	PPP2R3A	4.36340004	3.4281E-86	1.5474E-84
1027	COL4A6	4.36251822	2.7362E-61	7.0768E-60
1028	POTEF	4.36170656	1.3927E-07	3.9506E-07
1029	INE2	4.36160621	8.0029E-27	6.9428E-26
1030	NMUR1	4.35987707	6.626E-49	1.1864E-47

1031	CXXC4	4.35593168	3.5474E-23	2.6059E-22
1032	KAZALD1	4.3541712	1.8489E-45	2.9892E-44
1033	DNMT3B	4.34849857	8.5536E-48	1.4834E-46
1034	DCX	4.34112538	4.1872E-07	1.1381E-06
1035	RTKN2	4.33468537	1.2329E-24	9.742E-24
1036	OXTR	4.33359701	6.1517E-12	2.442E-11
1037	KIF25-AS1	4.33125002	0.00969466	0.01609806
1038	SBSPON	4.32070593	3.9019E-54	8.1321E-53
1039	LINC01449	4.32041169	0.01130738	0.0185559
1040	MYL7	4.31847448	2.418E-26	2.0525E-25
1041	RGS7	4.31813513	0.01369148	0.02218006
1042	ATP8B5P	4.31799005	0.00014953	0.00031638
1043	KLHL31	4.31648285	3.0333E-11	1.1516E-10
1044	FER1L5	4.31563796	1.9274E-05	4.4967E-05
1045	ZNF578	4.31547179	1.2124E-31	1.2634E-30
1046	TRHDE-AS1	4.31170784	3.7692E-10	1.3149E-09
1047	SHISA2	4.31132975	0.00034932	0.00070714
1048	NCCRP1	4.30793632	2.3445E-12	9.5579E-12
1049	TRPC4	4.305876	3.2411E-06	8.1407E-06
1050	LOC102724105	4.3032642	6.4576E-34	7.2577E-33
1051	HOXD11	4.29811656	1.7225E-05	4.0391E-05
1052	MISP	4.29486918	1.9287E-14	9.0069E-14
1053	ANO9	4.29342203	4.6108E-31	4.6811E-30
1054	ZNF737	4.29268901	1.1432E-70	3.7434E-69
1055	BRD2	4.2907092	1.222E-08	3.7787E-08
1056	NEK5	4.29005051	0.0201535	0.03175644
1057	MEX3A	4.28562706	3.7759E-53	7.7033E-52
1058	SEMA3D	4.28545232	8.9604E-21	5.8883E-20
1059	SLC34A2	4.28401537	6.1922E-06	1.5126E-05
1060	PLXDC1	4.28127492	2.5371E-10	8.9724E-10
1061	ESYT3	4.28056158	1.3476E-24	1.0626E-23
1062	DNAH12	4.27915463	4.917E-14	2.2437E-13
1063	FAXC	4.27464456	2.1578E-27	1.9203E-26
1064	PTN	4.27295726	1.5212E-14	7.1473E-14
1065	CAND1.11	4.26859492	0.00974196	0.01617242
1066	EPHX4	4.26428318	1.4657E-09	4.8874E-09
1067	TC2N	4.26231089	1.7397E-44	2.7326E-43
1068	FRMD5	4.26102869	1.4133E-09	4.7182E-09
1069	SESN3	4.25946016	1.4534E-78	5.6047E-77
1070	EBF2	4.25487321	3.6091E-05	8.184E-05
1071	CACNA2D1	4.25283742	1.0412E-05	2.4874E-05
1072	SHISA6	4.25238178	0.00134803	0.00253691
1073	LINC00511	4.24513965	0.01312896	0.02134215
1074	SPINK1	4.24492455	1.3382E-22	9.5717E-22
1075	12-Sep	4.23674033	0.00030372	0.00062008

1076	ELOVL4	4.23642877	4.819E-32	5.0932E-31
1077	ADRBK2	4.23113233	3.7652E-28	3.464E-27
1078	S100Z	4.22683155	9.6609E-08	2.7722E-07
1079	LOC101927230	4.21740735	0.00132876	0.00250357
1080	PGF	4.21666592	1.2415E-52	2.4959E-51
1081	TAS2R4	4.20959601	0.01068777	0.01761089
1082	CA5B	4.20703681	1.7176E-61	4.4721E-60
1083	DAGLA	4.20544707	2.922E-124	2.476E-122
1084	MYO9A	4.20057155	1.123E-104	6.826E-103
1085	GPX8	4.19693817	1.333E-182	2.321E-180
1086	MICALCL	4.19375439	4.5535E-24	3.498E-23
1087	TYRO3	4.19168831	5.254E-124	4.432E-122
1088	PLD5	4.19058006	5.946E-12	2.3637E-11
1089	PNMAL1	4.19011896	8.708E-50	1.6023E-48
1090	C22orf24	4.18729282	2.476E-05	5.707E-05
1091	PCSK2	4.18726753	0.00070272	0.00137289
1092	SH3GL1P2	4.17996669	0.0005506	0.00108813
1093	CAMK2A	4.17771085	6.1901E-09	1.9641E-08
1094	SEZ6L2	4.17198317	2.7419E-27	2.4322E-26
1095	IL37	4.16921992	0.01267341	0.02063982
1096	SYT4	4.16732352	0.00343728	0.00612565
1097	CHST9	4.16488891	1.1013E-46	1.8422E-45
1098	TMC4	4.16251797	4.0915E-94	2.0585E-92
1099	LOC728485	4.16075169	1.4616E-06	3.7874E-06
1100	IFNE	4.16038244	0.01593925	0.0255324
1101	COX6B2	4.15954547	0.02208521	0.03452713
1102	ZNF154	4.15935884	3.6723E-81	1.475E-79
1103	FIRRE	4.15749367	3.6278E-07	9.9248E-07
1104	LOC101929374	4.15578133	0.00050988	0.0010116
1105	FGF18	4.15415074	0.00052245	0.00103557
1106	EPS8L1	4.15335356	2.265E-103	1.342E-101
1107	B4GALNT3	4.1532928	3.469E-65	9.9733E-64
1108	BMP8A	4.1497368	4.1717E-20	2.6477E-19
1109	C15orf56	4.14555094	2.0642E-05	4.7967E-05
1110	MYOF	4.14417314	4.569E-164	6.587E-162
1111	SYT13	4.13713282	5.1688E-19	3.11E-18
1112	ZNF391	4.13257296	1.4662E-24	1.1534E-23
1113	SLC27A6	4.13112898	0.00152404	0.00285121
1114	BCL11A	4.12997323	2.7129E-07	7.4877E-07
1115	BMP7	4.12611876	2.5403E-09	8.31E-09
1116	TFAP2A	4.12527224	0.00210825	0.00386997
1117	SCRN1	4.12279243	1.037E-268	3.929E-266
1118	BEND6	4.12181762	1.6344E-06	4.2175E-06
1119	TMC1	4.11791031	0.00074966	0.001459
1120	IL31RA	4.11583262	0.02446638	0.03795187

1121	TNFSF9	4.1138013	4.602E-21	3.0659E-20
1122	SLC2A1	4.11324341	3.3193E-40	4.6103E-39
1123	RGPD2	4.10963092	0.0005447	0.00107724
1124	SCUBE3	4.10319138	1.445E-22	1.0313E-21
1125	DIAPH3	4.10269636	1.0547E-43	1.6159E-42
1126	BDKRB1	4.10044724	0.00012793	0.00027307
1127	SERPINA12	4.09507511	1.5754E-16	8.3003E-16
1128	EPHX3	4.09292692	2.1787E-28	2.0218E-27
1129	SNORD116-20	4.09256308	0.01423644	0.02300887
1130	PAPPA	4.09239109	1.6168E-09	5.3771E-09
1131	PLCE1	4.0914735	2.5885E-55	5.594E-54
1132	LRMP	4.09071737	4.7803E-82	1.965E-80
1133	AMHR2	4.09012785	1.6588E-31	1.7193E-30
1134	SLC5A9	4.08954922	6.5125E-48	1.1345E-46
1135	ZSCAN1	4.08799173	2.8915E-05	6.622E-05
1136	NOG	4.08739458	0.00011612	0.000249
1137	LOC101927901	4.08705661	0.01678522	0.02678094
1138	EPSTI1	4.08450372	1.4039E-40	1.9726E-39
1139	PIGZ	4.08306694	1.7626E-62	4.7296E-61
1140	NUDT11	4.08169063	2.7768E-09	9.0531E-09
1141	ISL2	4.08122024	3.1147E-05	7.1096E-05
1142	FAM212B-AS1	4.08030427	2.5103E-11	9.581E-11
1143	ZNF793	4.07925275	1.4011E-50	2.6436E-49
1144	CEL	4.07400074	1.0197E-23	7.6899E-23
1145	TRIM6	4.07227334	8.6837E-93	4.2798E-91
1146	DNASE1	4.0721513	2.688E-49	4.8762E-48
1147	C10orf95	4.07017367	5.5657E-62	1.469E-60
1148	TUB	4.0689119	1.1679E-14	5.5344E-14
1149	HIF3A	4.06790587	1.8965E-33	2.1046E-32
1150	DNAH9	4.06682465	0.02500141	0.038701
1151	PLCE1-AS1	4.06518932	2.3483E-09	7.7042E-09
1152	GRPR	4.06288139	7.3079E-07	1.9421E-06
1153	RASSF10	4.06264115	3.9328E-09	1.2659E-08
1154	CCSER1	4.06159511	5.4616E-24	4.179E-23
1155	SLC35F2	4.05841929	3.4155E-20	2.1821E-19
1156	PIEZO2	4.05837748	1.8113E-48	3.2076E-47
1157	TSIX	4.05574409	0.00651177	0.01111668
1158	LINC01508	4.05062454	0.01650652	0.02637329
1159	TSPAN5	4.04472247	6.5207E-65	1.8554E-63
1160	MYOZ3	4.03902718	6.3237E-08	1.8444E-07
1161	IL1RAPL1	4.03685468	0.00107958	0.00205735
1162	PLCH1	4.0365268	1.0041E-15	5.0847E-15
1163	C5orf46	4.03613122	0.0240908	0.03741463
1164	FOSL2	4.03591962	8.7213E-38	1.1248E-36
1165	TUBA3E	4.03582127	1.9577E-05	4.5619E-05

1166	PLXNC1	4.03120383	1.1819E-19	7.3471E-19
1167	EDIL3	4.03027274	3.8732E-20	2.4656E-19
1168	DOCK3	4.02797281	7.1488E-14	3.23E-13
1169	WEE2	4.02780993	4.541E-12	1.8194E-11
1170	MEGF11	4.02668251	0.01727236	0.02751274
1171	H2AFY2	4.02566507	9.9791E-50	1.8327E-48
1172	ANXA13	4.01915191	1.9932E-16	1.0439E-15
1173	FUT2	4.01882025	3.7052E-24	2.8611E-23
1174	TMEM178A	4.01597112	2.7932E-19	1.7093E-18
1175	EPHA7	4.0126983	3.4076E-28	3.138E-27
1176	FAM169A	4.00811802	2.054E-144	2.168E-142
1177	BTN1A1	4.00616328	0.00171176	0.00318145
1178	RNF183	4.00407748	0.00072501	0.00141402
1179	ZNF321P	3.99686909	6.5699E-38	8.536E-37
1180	GRIP2	3.99049245	2.5835E-15	1.2752E-14
1181	SNORD17	3.98619468	0.01840867	0.02920235
1182	GPC2	3.98090091	4.5831E-12	1.8355E-11
1183	CDR1	3.97558179	2.4444E-13	1.0658E-12
1184	HCAR1	3.97052664	0.00602512	0.01033611
1185	C9orf84	3.97038654	0.01616402	0.02586886
1186	SLC45A2	3.96354081	1.6715E-05	3.9234E-05
1187	SEMA3B	3.96030691	1.0568E-82	4.3909E-81
1188	SKA1	3.95785886	2.171E-30	2.1531E-29
1189	FAM181B	3.95418276	7.396E-05	0.00016193
1190	ITGB8	3.94736994	7.7704E-50	1.4339E-48
1191	LOC101927354	3.94710857	9.2879E-05	0.00020126
1192	GOLM1	3.94628703	3.445E-261	1.148E-258
1193	ST6GALNAC1	3.93063777	3.6935E-19	2.2425E-18
1194	RALGPS1	3.93050167	7.7962E-54	1.6144E-52
1195	CD24	3.92952102	2.1013E-55	4.5564E-54
1196	CDC42BPG	3.92738218	1.884E-147	2.068E-145
1197	PPP1R9A	3.9244685	4.4885E-95	2.3307E-93
1198	TRO	3.92257868	4.5685E-59	1.1047E-57
1199	ERBB4	3.92151283	2.2407E-06	5.7142E-06
1200	ERMN	3.91763824	0.00020168	0.00042005
1201	KIAA1324	3.91646624	1.4275E-08	4.3888E-08
1202	FLNC	3.90784502	4.9896E-56	1.1093E-54
1203	ABCC13	3.90735707	0.03127712	0.04760312
1204	CTHRC1	3.89983461	2.6568E-13	1.155E-12
1205	LOC645166	3.89694713	2.6022E-14	1.2065E-13
1206	OCA2	3.89668824	1.6712E-09	5.5495E-09
1207	EFHC2	3.8951503	0.00050882	0.0010097
1208	MS4A8	3.89489898	7.3572E-06	1.7843E-05
1209	CRABP1	3.89448423	0.00494858	0.00859849
1210	SSTR5	3.89255348	0.0024567	0.00447262

1211	LOC101927668	3.89213233	0.00181744	0.00336686
1212	FXYD3	3.89091504	4.8993E-61	1.257E-59
1213	ZNF320	3.88249112	3.514E-132	3.232E-130
1214	GFRA3	3.88224901	7.551E-10	2.5716E-09
1215	CYP27C1	3.88008095	2.7846E-08	8.363E-08
1216	MFI2	3.87586479	4.8616E-51	9.3367E-50
1217	ZIK1	3.8754856	7.3384E-74	2.5454E-72
1218	ALG1L2	3.87039416	0.02086871	0.03278447
1219	ROS1	3.86660053	0.00028358	0.00058093
1220	LINC01057	3.85898432	3.009E-22	2.1186E-21
1221	STX3	3.85664821	1.866E-177	3.03E-175
1222	ELOVL7	3.85552944	1.622E-36	2.0087E-35
1223	CDKN2B	3.85356848	1.6368E-21	1.1131E-20
1224	MCF2L2	3.85073057	2.7117E-08	8.1528E-08
1225	TNFSF15	3.85046403	2.3425E-05	5.4123E-05
1226	HCAR2	3.84151869	6.7477E-09	2.1319E-08
1227	RASGRF2-AS1	3.84107968	0.0023438	0.00427796
1228	UBE2Q2	3.84106714	4.478E-79	1.7442E-77
1229	IGF1R	3.84076867	4.695E-109	3.044E-107
1230	UNC93A	3.84027381	1.478E-157	1.879E-155
1231	SORCS1	3.83256554	1.4601E-09	4.8696E-09
1232	HS3ST5	3.83151275	0.00271515	0.00491259
1233	ANKK1	3.82896285	7.2509E-08	2.1022E-07
1234	C15orf48	3.82653805	7.1206E-13	3.0077E-12
1235	BMP8B	3.82511152	7.0675E-13	2.9859E-12
1236	SKP2	3.82205554	8.569E-189	1.562E-186
1237	SLC5A3	3.82162058	9.7484E-63	2.6268E-61
1238	CDC42EP3	3.8203642	1.8214E-17	1.0109E-16
1239	KHDC1	3.81805489	1.2658E-09	4.2406E-09
1240	FAAHP1	3.81762913	0.02462861	0.0381698
1241	LRRC19	3.81658199	1.323E-38	1.7568E-37
1242	DLK1	3.80922529	3.938E-10	1.3721E-09
1243	PRSS50	3.80706577	5.3584E-11	1.9916E-10
1244	SNORD116-28	3.80611449	0.02739405	0.04211116
1245	FUT8	3.80262958	5.2462E-41	7.4802E-40
1246	ZNF665	3.80198495	5.2727E-12	2.1047E-11
1247	CCDC183-AS1	3.80018966	3.3084E-32	3.5082E-31
1248	ANP32A-IT1	3.79998137	0.00017672	0.00037067
1249	LOC100288152	3.79391005	3.381E-13	1.4597E-12
1250	KIAA1024	3.79361831	2.4505E-15	1.211E-14
1251	PCDHA12	3.792729	2.2238E-73	7.6858E-72
1252	SPTSSB	3.79250983	2.568E-19	1.574E-18
1253	PRSS45	3.78993065	2.3949E-15	1.1846E-14
1254	GYG2P1	3.78962651	5.4005E-23	3.9313E-22
1255	GALNT7	3.78695229	4.2797E-98	2.3556E-96

1256	SLIT3	3.78540114	1.032E-147	1.139E-145
1257	IMPG2	3.77958258	2.5924E-15	1.279E-14
1258	CTB-178M22.2	3.77955073	0.02909104	0.04452851
1259	NRARP	3.77906784	7.3775E-54	1.5293E-52
1260	PTGS1	3.77839839	0.00094262	0.0018128
1261	CACNB3	3.77487395	1.5821E-46	2.6373E-45
1262	USP46	3.77309651	1.8493E-65	5.3728E-64
1263	LEF1	3.76940276	3.7757E-12	1.5178E-11
1264	CGA	3.76892348	0.03176476	0.04826544
1265	LOC100129924	3.76824101	0.02666836	0.04108386
1266	EYA1	3.7677552	0.00453313	0.00793082
1267	DUSP15	3.76747553	9.7824E-24	7.3855E-23
1268	HK2	3.76175272	2.1811E-69	6.9081E-68
1269	ZRANB3	3.75679544	2.0518E-35	2.4515E-34
1270	ZNF816-ZNF321P	3.75562583	8.0747E-51	1.5431E-49
1271	TCEAL5	3.75024136	0.00251686	0.00457568
1272	ZNF85	3.74946871	1.2933E-30	1.2913E-29
1273	SERPINE2	3.74437205	3.388E-14	1.5611E-13
1274	CNTN5	3.74402764	0.00013269	0.00028283
1275	ZNF738	3.74286567	2.9486E-68	9.1441E-67
1276	CCDC183	3.74137625	2.8478E-29	2.7197E-28
1277	PCDHA4	3.7393006	1.8161E-63	4.9629E-62
1278	TMEM130	3.7383594	0.02970323	0.04540082
1279	WNT10A	3.73830894	0.0002668	0.00054784
1280	ZNF43	3.73351996	1.0436E-57	2.4175E-56
1281	C9orf131	3.732667	0.031496	0.04789475
1282	NETO2	3.73236158	1.4649E-47	2.5158E-46
1283	UACA	3.72590553	1.019E-114	7.212E-113
1284	HOMER1	3.72317492	7.9105E-69	2.4851E-67
1285	PCDHA10	3.72156265	3.6027E-58	8.478E-57
1286	ARSI	3.71049557	4.1905E-19	2.5348E-18
1287	GGT3P	3.71022217	0.00052524	0.001041
1288	HSD3B2	3.70391487	3.827E-07	1.0445E-06
1289	FKBP10	3.7038134	2.104E-81	8.5397E-80
1290	ZNF415	3.70158539	3.4921E-70	1.1244E-68
1291	RAB19	3.69861832	0.00011295	0.00024252
1292	MGAT5	3.69840586	5.9543E-20	3.7508E-19
1293	TMEM65	3.69681188	2.928E-152	3.429E-150
1294	SH3YL1	3.69615428	1.2092E-95	6.3484E-94
1295	TEKT3	3.69370331	0.00463872	0.00809723
1296	ABO	3.69340525	2.828E-21	1.9048E-20
1297	TMEM51	3.69297896	7.5282E-44	1.1589E-42
1298	RHOF	3.6914434	1.616E-166	2.347E-164
1299	ITGA11	3.69027769	6.3888E-23	4.6368E-22

1300	ZNF670-ZNF695	3.68974417	3.634E-08	1.0825E-07
1301	TNC	3.68778787	5.8755E-15	2.8364E-14
1302	LINC01214	3.68692034	0.00015234	0.00032198
1303	KLK11	3.68638141	5.2877E-09	1.6847E-08
1304	AMN	3.68630753	1.996E-148	2.242E-146
1305	HUNK	3.68215281	5.8208E-14	2.6448E-13
1306	BEST4	3.68154894	0.00062047	0.00121982
1307	ID4	3.67923178	2.3191E-44	3.6279E-43
1308	LOC646903	3.67922282	4.349E-07	1.1809E-06
1309	SALL2	3.6790835	8.3198E-59	1.9968E-57
1310	GCSAM	3.67853449	7.2338E-18	4.1069E-17
1311	PNLIPRP2	3.67818282	1.6401E-07	4.6164E-07
1312	PRKG2	3.67740038	7.3875E-05	0.00016177
1313	FBLL1	3.67571989	1.761E-05	4.1265E-05
1314	ROBO2	3.67215514	9.2415E-15	4.4009E-14
1315	WIPF3	3.66745605	7.6445E-50	1.412E-48
1316	CPA4	3.66598918	2.2209E-08	6.721E-08
1317	OTUD3	3.65930713	2.6121E-60	6.6229E-59
1318	OVOL2	3.65847565	1.0566E-08	3.2863E-08
1319	LNX1-AS2	3.65349303	0.00640882	0.0109506
1320	AGPAT4	3.65183064	2.296E-70	7.4427E-69
1321	NRXN3	3.65129743	2.2391E-23	1.6639E-22
1322	STYK1	3.6506807	3.7294E-10	1.3022E-09
1323	ROR2	3.64815164	8.6083E-30	8.3827E-29
1324	TSPEAR-AS1	3.64782292	8.7279E-18	4.9377E-17
1325	ZNF732	3.64412435	4.088E-07	1.1119E-06
1326	PDCL3P4	3.64328524	2.8979E-19	1.7723E-18
1327	PCDHA11	3.64243014	2.1224E-65	6.1567E-64
1328	NECAB1	3.64085357	4.4307E-18	2.5408E-17
1329	HFM1	3.63534485	0.00071742	0.00140005
1330	MOBP	3.61606934	0.00322993	0.00578013
1331	ZNF660	3.61554151	1.4155E-18	8.3452E-18
1332	ABCC4	3.61508738	3.5259E-94	1.7786E-92
1333	LRRN2	3.6140254	1.2362E-21	8.475E-21
1334	PKIB	3.61340522	5.714E-93	2.8452E-91
1335	DPF1	3.60669186	0.00853131	0.01429408
1336	HOXD10	3.60606701	0.00121743	0.00230392
1337	BCL2L14	3.60586575	2.0329E-11	7.8067E-11
1338	ZNF664-FAM101A	3.59860841	7.8504E-16	3.9965E-15
1339	CLIC5	3.59388255	7.1289E-28	6.4693E-27
1340	PTPN14	3.59337598	9.9644E-77	3.688E-75
1341	CARD11	3.59272504	1.2794E-16	6.7775E-16
1342	SYTL5	3.58991592	6.0158E-56	1.3298E-54
1343	BMF	3.58863416	8.3362E-45	1.3223E-43

1344	LINC00954	3.58602636	1.22E-07	3.4771E-07
1345	SLC9A3	3.58493059	6.9697E-12	2.7559E-11
1346	TIAM2	3.58127835	1.2205E-67	3.7428E-66
1347	AGAP7P	3.58110549	9.28E-05	0.00020113
1348	PRDM5	3.57737982	6.1473E-33	6.7024E-32
1349	KLF8	3.5749052	1.5366E-46	2.5637E-45
1350	BARX2	3.57482567	0.00170469	0.00316957
1351	GNAS-AS1	3.57471955	0.00042067	0.00084318
1352	PCDHA2	3.57444926	8.556E-66	2.5199E-64
1353	ZNF610	3.57422584	1.5543E-10	5.5825E-10
1354	GABRA4	3.56856466	0.00028537	0.00058428
1355	DSCAML1	3.56501021	2.6403E-28	2.4431E-27
1356	ZNF607	3.56442067	2.457E-33	2.7234E-32
1357	TSPY26P	3.55887533	3.0241E-20	1.9359E-19
1358	TENM4	3.5579057	0.00015022	0.00031774
1359	LOC102723354	3.55638126	9.1063E-08	2.6173E-07
1360	PCDHB5	3.55483846	2.8015E-28	2.5909E-27
1361	POU4F1	3.55398818	0.0002206	0.00045704
1362	SLC39A10	3.5520304	1.5766E-51	3.0798E-50
1363	FANCB	3.55053984	3.9349E-09	1.2662E-08
1364	ZSCAN23	3.54935293	1.2071E-10	4.3763E-10
1365	PLEKHG6	3.54742185	1.0932E-63	3.0043E-62
1366	ZNF462	3.54623698	1.8895E-75	6.7479E-74
1367	SPON1	3.54484321	6.252E-05	0.00013806
1368	STC1	3.5431781	1.5637E-10	5.6152E-10
1369	4-Mar	3.54140211	0.00950692	0.01580396
1370	CCDC162P	3.54124071	1.6969E-06	4.3723E-06
1371	LPAR2	3.53706483	1.6791E-32	1.7992E-31
1372	ERP27	3.53670112	5.474E-11	2.033E-10
1373	GALNT3	3.53574155	1.6756E-31	1.7349E-30
1374	PCDHA3	3.53206429	1.1666E-55	2.5525E-54
1375	LRP4	3.5315844	1.3134E-51	2.5787E-50
1376	MYBPC1	3.53130678	0.03270762	0.04959278
1377	C9orf172	3.52886475	8.1931E-19	4.878E-18
1378	LOC254896	3.52424413	1.0286E-18	6.0959E-18
1379	MTMR2	3.52396161	6.5698E-56	1.4473E-54
1380	TMEM178B	3.51839059	8.0037E-10	2.7219E-09
1381	PLAG1	3.51516472	1.0663E-64	3.0032E-63
1382	RAD21-AS1	3.51348556	8.887E-10	3.0138E-09
1383	ZNF813	3.51312354	4.2918E-55	9.1999E-54
1384	MAP1LC3C	3.50865884	3.7584E-10	1.3114E-09
1385	PNMA3	3.50723543	9.5357E-06	2.2886E-05
1386	COL12A1	3.50268581	2.2247E-22	1.5727E-21
1387	PCDHA5	3.50062155	1.1323E-57	2.6136E-56
1388	SV2A	3.50001417	5.0893E-25	4.0935E-24

1389	PCDHA7	3.49791621	1.2594E-59	3.1075E-58
1390	PCDHA8	3.49412206	1.3738E-53	2.8295E-52
1391	PCDHA9	3.49379875	1.6709E-55	3.6352E-54
1392	PRR5-ARHGAP8	3.49241702	9.503E-119	7.2E-117
1393	DIRC2	3.49118663	6.6236E-22	4.5998E-21
1394	HRASLS5	3.48837058	1.8067E-09	5.979E-09
1395	KPNA7	3.48447116	5.1361E-07	1.3849E-06
1396	LYPD1	3.48426035	1.294E-34	1.5015E-33
1397	LOC101927043	3.48287487	0.00048473	0.00096387
1398	PCDHA6	3.48195649	8.5134E-53	1.7223E-51
1399	DNM1	3.48163874	6.2452E-18	3.555E-17
1400	ZNF223	3.4794273	2.6206E-71	8.6842E-70
1401	S100A4	3.47807942	5.9469E-11	2.2031E-10
1402	RHBG	3.47654359	2.4384E-22	1.7187E-21
1403	TTN	3.47418791	1.8529E-43	2.8254E-42
1404	MCTP2	3.47221798	2.1707E-45	3.5036E-44
1405	CHST10	3.47125944	7.6668E-17	4.1077E-16
1406	PCDHA1	3.47074352	8.6704E-54	1.7935E-52
1407	KANK4	3.46816841	4.4589E-28	4.0886E-27
1408	TMEM164	3.46691046	2.695E-105	1.658E-103
1409	HSD17B14	3.46541867	6.184E-153	7.285E-151
1410	TUBBP5	3.46417615	5.8029E-23	4.2163E-22
1411	HOOK3	3.45989499	2.0915E-38	2.7582E-37
1412	LYPD6	3.45663698	6.817E-10	2.3294E-09
1413	RNF182	3.45498983	8.1931E-05	0.0001787
1414	NEXN-AS1	3.45457927	3.8679E-09	1.2455E-08
1415	ABCA7	3.45457587	4.5279E-54	9.4266E-53
1416	PDZK1IP1	3.45396338	2.7649E-15	1.3624E-14
1417	MTRNR2L1	3.45386825	5.2601E-23	3.832E-22
1418	SHOX2	3.45102475	0.00028775	0.00058905
1419	LOC101929633	3.44793565	9.5443E-09	2.978E-08
1420	NSG1	3.44732121	1.1564E-18	6.8467E-18
1421	C21orf90	3.44660109	2.1641E-10	7.7027E-10
1422	C17orf53	3.44614141	5.3122E-25	4.2692E-24
1423	SLC25A36	3.4399548	3.2238E-50	6.0003E-49
1424	C2orf15	3.43993524	1.3315E-27	1.1948E-26
1425	TRIM9	3.4376645	1.5247E-21	1.0398E-20
1426	PPM1J	3.43373222	1.1011E-16	5.8541E-16
1427	ZNF761	3.43307569	2.705E-117	1.995E-115
1428	STARD4-AS1	3.43096152	5.1449E-63	1.402E-61
1429	LAMC1	3.42856612	3.99E-118	2.999E-116
1430	LOC284581	3.4278234	1.6321E-08	4.9924E-08
1431	ITGA3	3.42737638	9.2763E-60	2.3006E-58
1432	MTCL1	3.42665839	9.0089E-41	1.2723E-39

1433	NINL	3.41929055	5.453E-116	3.946E-114
1434	JAKMIP2-AS1	3.41797856	0.00184587	0.00341395
1435	UCHL1	3.41551604	4.1171E-25	3.3226E-24
1436	CSPP1	3.41413242	2.5648E-33	2.8364E-32
1437	ESRRG	3.41290624	2.1003E-17	1.163E-16
1438	NPY2R	3.41236823	0.0144553	0.02333331
1439	RASEF	3.41022834	9.143E-15	4.3573E-14
1440	PCLO	3.40964058	1.1644E-08	3.6099E-08
1441	ADAMTS3	3.40827816	2.7708E-19	1.6968E-18
1442	HS6ST3	3.40750703	0.000483	0.00096053
1443	S100A10	3.40622058	3.8181E-31	3.8865E-30
1444	RBM46	3.40495632	2.5293E-06	6.4103E-06
1445	CLIP2	3.4046828	6.0687E-63	1.6444E-61
1446	LAYN	3.40380436	6.5503E-21	4.3355E-20
1447	TTC39A	3.40377391	4.2986E-39	5.7673E-38
1448	RIMS2	3.40313475	0.00814234	0.01369581
1449	ADAM23	3.39806349	4.9959E-16	2.5684E-15
1450	GAREML	3.39425801	4.587E-14	2.0985E-13
1451	HERC2P10	3.39417343	0.00167648	0.00312189
1452	RGS20	3.39404296	0.01179898	0.01931506
1453	KIAA1377	3.39048032	3.4828E-16	1.8035E-15
1454	RNF212	3.3806323	9.9733E-11	3.639E-10
1455	CPEB1	3.3769265	2.1878E-07	6.0949E-07
1456	SORT1	3.37413564	1.593E-106	9.931E-105
1457	ENPP5	3.37370274	1.9327E-20	1.2484E-19
1458	HOXB8	3.3730762	0.00992202	0.01644168
1459	SH2D3A	3.37169381	3.3468E-68	1.0362E-66
1460	KRTAP5-AS1	3.36888928	1.5582E-09	5.1851E-09
1461	PCDHA13	3.36860636	9.3288E-53	1.8833E-51
1462	UG0898H09	3.36844745	0.00065859	0.00129046
1463	INPP4B	3.3681239	3.6934E-12	1.4857E-11
1464	HOXA10-HOXA9	3.36503944	7.4078E-08	2.1457E-07
1465	CCDC8	3.36364897	2.2291E-33	2.4722E-32
1466	C20orf144	3.36262843	0.00223395	0.00408828
1467	AMOT	3.36202807	1.8091E-35	2.1682E-34
1468	CAGE1	3.36149193	0.00030662	0.00062567
1469	SLC14A2	3.3591436	0.00306416	0.00550745
1470	ZNF614	3.35879581	3.1029E-91	1.495E-89
1471	PRSS8	3.35546596	2.0262E-69	6.4278E-68
1472	RANBP17	3.35401093	1.3954E-17	7.794E-17
1473	STIL	3.35215084	1.0727E-29	1.0398E-28
1474	C20orf202	3.35044386	0.00011356	0.0002437
1475	SNCA	3.34940429	4.1452E-46	6.8217E-45
1476	CHST3	3.34830985	3.0438E-38	3.9977E-37
1477	FAM84A	3.34755383	2.2463E-12	9.1731E-12

1478	LINC00540	3.34658041	8.4743E-05	0.00018448
1479	ZNF677	3.34591739	7.5114E-20	4.7087E-19
1480	BICD1	3.34583896	1.2421E-36	1.5452E-35
1481	TSGA13	3.34072459	0.00030325	0.0006192
1482	CMTM4	3.33987684	1.2058E-56	2.7248E-55
1483	LOC100129940	3.33868685	0.02501644	0.03871542
1484	MFI2-AS1	3.33852438	7.0236E-16	3.5851E-15
1485	HAS2	3.33505277	0.0020621	0.00379173
1486	ZNF611	3.32967384	1.3624E-85	6.0647E-84
1487	GNG13	3.32656626	0.00144947	0.00271933
1488	SYT10	3.31521893	5.0197E-05	0.00011201
1489	C4orf48	3.31514166	2.5843E-24	2.0068E-23
1490	FAM131C	3.31370202	0.00016237	0.00034191
1491	FAM227A	3.31086209	5.8216E-07	1.5616E-06
1492	C10orf126	3.30754107	0.02775272	0.04261844
1493	PWAR5	3.30636311	1.4667E-18	8.6372E-18
1494	ARL15	3.30615566	2.4905E-50	4.6624E-49
1495	LINC00400	3.3046394	0.00032972	0.00067046
1496	WNT4	3.30427694	2.6472E-09	8.648E-09
1497	VIL1	3.30266534	1.037E-144	1.101E-142
1498	TMSB15A	3.30202338	0.00085838	0.00165922
1499	ANKRD18B	3.29391423	0.01646546	0.02631422
1500	EZR-AS1	3.29368972	3.7445E-40	5.1822E-39
1501	LOC100133669	3.29203844	4.2137E-28	3.8674E-27
1502	LINC00880	3.29156382	8.0559E-06	1.946E-05
1503	ASRGL1	3.29118337	1.4943E-23	1.1164E-22
1504	SRSF12	3.29051285	1.1416E-34	1.3278E-33
1505	CASK	3.28949205	2.4424E-64	6.8289E-63
1506	HBQ1	3.28895529	0.00418167	0.00735858
1507	ROR1	3.28768856	4.4437E-10	1.5433E-09
1508	SRGAP1	3.28450568	1.368E-99	7.6834E-98
1509	FOXD1	3.28193878	0.02051723	0.03228217
1510	TEX41	3.27717466	0.00559877	0.00964324
1511	HOXC4	3.27624164	0.00040353	0.00081049
1512	CHD3	3.27543247	1.8153E-89	8.5124E-88
1513	CA11	3.27506992	1.6313E-15	8.1521E-15
1514	PAQR7	3.27403061	2.6661E-49	4.8456E-48
1515	KIAA1549	3.27273942	8.6549E-51	1.6507E-49
1516	INPP5J	3.27026742	1.3367E-11	5.1971E-11
1517	GSN	3.26872973	1.0592E-44	1.6719E-43
1518	GDNF-AS1	3.26740376	0.0328067	0.0497352
1519	UCN2	3.26727295	0.0057215	0.00983883
1520	PCDHAC1	3.26553581	3.8361E-51	7.3966E-50
1521	C5orf34	3.26338642	3.9369E-17	2.1462E-16
1522	LOC101929378	3.26256119	1.1547E-05	2.747E-05

1523	PLXNA4	3.26171206	3.3199E-13	1.434E-12
1524	FSD1	3.26159029	1.9451E-05	4.5343E-05
1525	DIO3	3.26033214	7.8315E-09	2.4558E-08
1526	CACNA1D	3.25923357	3.9587E-48	6.9341E-47
1527	FHDC1	3.25812697	1.2645E-20	8.2311E-20
1528	ORC6	3.25441545	1.792E-32	1.9191E-31
1529	BNC1	3.24948389	6.9884E-05	0.00015346
1530	DNAJC6	3.24946966	6.231E-54	1.2944E-52
1531	SLC37A1	3.24687604	1.6582E-41	2.4051E-40
1532	HSF5	3.2468109	0.02560019	0.03953919
1533	SCN3A	3.24353249	1.9659E-11	7.5586E-11
1534	ALX4	3.24020863	0.01870271	0.0296396
1535	ARRB1	3.23684329	3.346E-101	1.907E-99
1536	FBF1	3.23603444	9.7329E-32	1.0192E-30
1537	SCD5	3.23601277	0.00065877	0.00129069
1538	ZNF724P	3.23332256	0.00097412	0.00186874
1539	FAM151A	3.23248003	2.9744E-39	4.0158E-38
1540	DIAPH3-AS1	3.23184513	0.00030717	0.00062667
1541	LCMT1-AS2	3.22547465	0.02366731	0.03680397
1542	CDH11	3.22132252	5.1464E-15	2.4957E-14
1543	CELF3	3.21959785	0.00012979	0.00027686
1544	SLC16A4	3.21869421	2.2316E-58	5.29E-57
1545	LAMA5	3.21775887	6.8472E-95	3.5277E-93
1546	SLC45A4	3.2168563	1.3677E-51	2.6826E-50
1547	FAM171A2	3.21621991	6.7488E-15	3.2459E-14
1548	LINC00665	3.215602	1.8375E-20	1.1881E-19
1549	OLFML3	3.2140524	1.3865E-18	8.1818E-18
1550	NRCAM	3.21325326	3.2896E-25	2.6659E-24
1551	FLJ44511	3.21182973	9.8115E-06	2.3516E-05
1552	PDP1	3.21166529	8.393E-124	7.05E-122
1553	ZNF488	3.21055542	3.5232E-06	8.8126E-06
1554	DLEU7	3.21024144	4.3211E-09	1.3865E-08
1555	LOXL1-AS1	3.20936688	2.2017E-19	1.3555E-18
1556	SKIL	3.20873468	6.5774E-27	5.7422E-26
1557	GSN-AS1	3.20805233	7.5175E-23	5.4396E-22
1558	SOWAHA	3.20772682	4.2657E-29	4.0478E-28
1559	GPR161	3.20687689	2.1917E-32	2.342E-31
1560	FAM46A	3.20413299	2.638E-32	2.8081E-31
1561	SLC44A5	3.20119725	1.4071E-13	6.2324E-13
1562	FZD3	3.19944721	2.0328E-31	2.0968E-30
1563	SLC51B	3.19821027	2.3049E-14	1.0712E-13
1564	BARD1	3.19817128	1.6496E-61	4.3069E-60
1565	LOC654342	3.19761009	1.1491E-32	1.2396E-31
1566	FAM162B	3.19676406	6.9404E-06	1.6896E-05
1567	LZTS3	3.19029282	8.258E-121	6.62E-119

1568	JAKMIP2	3.18978903	1.6832E-13	7.428E-13
1569	LOC100131289	3.18949443	0.00096944	0.00186049
1570	MYL4	3.18914404	3.1728E-09	1.0299E-08
1571	TMEM136	3.18296868	4.1272E-19	2.4981E-18
1572	COL5A2	3.1810819	6.4579E-23	4.6834E-22
1573	SLC4A3	3.17982323	1.7864E-13	7.876E-13
1574	ZYG11A	3.17688624	2.2852E-27	2.0327E-26
1575	SLC35F3	3.1765681	6.6178E-05	0.00014569
1576	NEDD4L	3.17623415	3.805E-115	2.713E-113
1577	FAM155A	3.17506425	6.935E-07	1.8455E-06
1578	CASC15	3.17376408	3.455E-07	9.4683E-07
1579	SYTL1	3.16976477	4.7581E-21	3.1666E-20
1580	CNKSR1	3.1649278	1.9348E-36	2.3931E-35
1581	LAMP3	3.16378739	5.0677E-11	1.8868E-10
1582	S100A13	3.16335984	1.9627E-47	3.3586E-46
1583	TMEM63C	3.16252038	1.0857E-06	2.8499E-06
1584	HDGFRP3	3.16247547	4.5422E-50	8.4299E-49
1585	PYCR1	3.16150452	4.4997E-95	2.3307E-93
1586	SPATS2	3.1586038	1.7357E-45	2.8109E-44
1587	LRRTM4	3.15793041	0.00012554	0.00026826
1588	SNAP25-AS1	3.15733564	0.00158933	0.0029656
1589	HRASLS	3.15506997	9.7544E-07	2.5693E-06
1590	SDK2	3.15466739	2.1934E-19	1.3509E-18
1591	KIAA1324L	3.1540416	1.7218E-39	2.341E-38
1592	C3orf58	3.15229293	2.1245E-59	5.1824E-58
1593	APOA4	3.15170636	0.00113161	0.00215058
1594	BDNF	3.15101389	9.8294E-18	5.5479E-17
1595	ZNF681	3.15082896	1.8845E-18	1.1049E-17
1596	3-Sep	3.14722769	9.6794E-05	0.0002093
1597	C5orf30	3.13859122	4.3149E-46	7.0947E-45
1598	RALYL	3.1357343	8.3131E-05	0.00018113
1599	CDS1	3.13326626	2.6662E-81	1.0754E-79
1600	LEPREL1	3.13053469	7.6185E-26	6.3171E-25
1601	CCNE1	3.12836695	9.326E-49	1.6652E-47
1602	IL18	3.12563486	2.4123E-43	3.6669E-42
1603	HOXA9	3.12459368	2.4368E-07	6.7555E-07
1604	RPS6KA6	3.123521	1.3276E-39	1.8102E-38
1605	ACPP	3.12148028	2.9302E-05	6.7068E-05
1606	AGBL3	3.11995297	2.1146E-14	9.8469E-14
1607	GLS	3.11625971	5.019E-129	4.448E-127
1608	GBGT1	3.1145802	3.0135E-34	3.445E-33
1609	ENAH	3.11454841	2.7637E-72	9.3841E-71
1610	LOC100289333	3.11365947	0.02083109	0.03273336
1611	CADPS	3.10968341	3.0333E-09	9.8643E-09
1612	LOC101926926	3.10929167	0.00162235	0.00302459

1613	ZNF430	3.10912715	7.1289E-46	1.1652E-44
1614	PEX5L	3.10761949	0.00054141	0.00107107
1615	LOC101928674	3.10717011	3.2154E-22	2.2615E-21
1616	ARTN	3.10601719	0.0045734	0.00799186
1617	IPO5P1	3.10449171	6.5129E-57	1.4734E-55
1618	B3GNT8	3.10309817	9.7637E-30	9.4744E-29
1619	LOC101928269	3.09998845	0.03009928	0.04596981
1620	ITGA2	3.09997046	1.333E-21	9.1295E-21
1621	CAPN8	3.09941057	9.7336E-09	3.0365E-08
1622	CDH24	3.09835769	3.8548E-23	2.8264E-22
1623	DRICH1	3.09588737	0.00101506	0.00194053
1624	RAB38	3.09381869	1.6819E-13	7.4242E-13
1625	APBB1	3.09369125	2.157E-101	1.233E-99
1626	RIMKLA	3.09187415	1.1139E-13	4.9623E-13
1627	FZD7	3.09031919	5.9232E-08	1.732E-07
1628	BAMBI	3.08898758	6.666E-22	4.6276E-21
1629	COCH	3.08721912	6.0256E-37	7.6038E-36
1630	ZNF382	3.08628	4.3943E-19	2.6563E-18
1631	PDPN	3.08502784	7.3047E-06	1.7723E-05
1632	KSR2	3.08375654	0.00182674	0.00338129
1633	CLCN1	3.08315031	0.00512348	0.00887922
1634	OBSCN	3.07905003	2.3691E-25	1.9256E-24
1635	LOC103344931	3.07903759	4.1554E-31	4.2254E-30
1636	PDE6B	3.07892811	7.0652E-22	4.8995E-21
1637	RAB34	3.07788173	2.9151E-53	5.9724E-52
1638	SCEL	3.07578619	1.0583E-07	3.0268E-07
1639	LOC100506985	3.07175569	6.4528E-09	2.0437E-08
1640	DCLK3	3.06674859	0.00536364	0.00926799
1641	LINC00312	3.06581735	1.7966E-05	4.2048E-05
1642	OTOGL	3.06317626	2.1753E-07	6.0618E-07
1643	ESM1	3.05915165	0.01085895	0.0178701
1644	HES4	3.05911564	2.7695E-51	5.3829E-50
1645	LOC440028	3.05780878	8.1661E-05	0.00017817
1646	FGF17	3.05759568	0.00029828	0.00060962
1647	FBLIM1	3.05747531	5.938E-53	1.2051E-51
1648	ACOT11	3.05607474	1.5517E-53	3.1892E-52
1649	SNX32	3.05398196	0.00835603	0.01402594
1650	GOLGA2P5	3.05013184	1.9189E-21	1.3003E-20
1651	GRID2IP	3.04999317	2.0531E-05	4.7721E-05
1652	SNORD30	3.04997999	0.00643587	0.01099584
1653	CRISPLD1	3.04961615	2.5125E-10	8.8904E-10
1654	GALNT6	3.04739444	6.2318E-62	1.6425E-60
1655	PIK3C2B	3.04596596	1.0489E-13	4.6802E-13
1656	WFDC2	3.04533339	4.7018E-23	3.4305E-22
1657	ANO4	3.04513157	0.00027419	0.00056253

1658	NEB	3.04502687	4.4289E-07	1.2018E-06
1659	C18orf54	3.04447219	3.7023E-11	1.3935E-10
1660	GPR98	3.04354603	8.3305E-19	4.9552E-18
1661	MAP7D2	3.04310502	3.5752E-13	1.5401E-12
1662	LOC101929439	3.04261214	0.02410475	0.03742998
1663	LPHN3	3.04162908	5.9528E-18	3.3926E-17
1664	DNMT3A	3.04072507	2.7462E-26	2.326E-25
1665	NCK2	3.03844397	1.427E-140	1.459E-138
1666	LINC00605	3.03685887	0.00680518	0.01158686
1667	MB21D2	3.03675814	3.6456E-46	6.0355E-45
1668	SOX9	3.03596163	4.0866E-38	5.3383E-37
1669	LOC642852	3.03354638	6.7275E-53	1.3639E-51
1670	C12orf49	3.0326215	4.4917E-85	1.9678E-83
1671	NHLH1	3.02947237	0.03156175	0.04798339
1672	PDE9A	3.02772929	1.1963E-79	4.6978E-78
1673	NLRP2	3.02587973	6.926E-07	1.8434E-06
1674	NME9	3.02499665	0.0027235	0.00492586
1675	LOC101928053	3.02104513	0.02280942	0.03557871
1676	SPACA6P	3.02023933	2.0555E-14	9.5833E-14
1677	SYT2	3.01989754	0.00030442	0.00062145
1678	MECOM	3.01902597	8.4123E-50	1.5493E-48
1679	DCDC2	3.01861558	2.9353E-68	9.1172E-67
1680	GAS7	3.01800349	3.2683E-32	3.4675E-31
1681	COL17A1	3.01724394	1.2331E-05	2.9289E-05
1682	CCDC160	3.01664728	0.00169129	0.00314759
1683	HCN4	3.0152397	1.3929E-05	3.2938E-05
1684	ADAM22	3.01354479	2.0872E-07	5.8263E-07
1685	TLN2	3.01337251	1.6987E-20	1.0994E-19
1686	PLEKHB1	3.01050104	3.2453E-47	5.5096E-46
1687	LOC644762	3.00945579	0.00490027	0.00852221
1688	IPW	3.00941337	5.0979E-65	1.457E-63
1689	ADAMTS14	3.00778413	1.6217E-17	9.0289E-17
1690	HSPA12A	3.00735692	2.0312E-18	1.1892E-17
1691	PFKP	3.00690037	8.4394E-84	3.5914E-82
1692	SPATA18	3.00584758	2.1302E-35	2.542E-34
1693	TRPC1	3.00310322	3.1184E-29	2.9752E-28
1694	PHGR1	3.0022463	5.1515E-09	1.6432E-08
1695	CCDC186	2.99973703	1.5261E-21	1.04E-20
1696	MAPK4	2.99806093	5.0379E-18	2.8822E-17
1697	PCNXL2	2.99703476	3.0578E-21	2.0563E-20
1698	LRP4-AS1	2.99613777	5.149E-09	1.6427E-08
1699	FZD2	2.99589343	1.3813E-10	4.9855E-10
1700	SPG20OS	2.99504847	0.00020993	0.00043612
1701	WNT5A	2.99452334	6.5696E-07	1.7521E-06
1702	KCTD12	2.99225205	0.00041334	0.00082892

1703	WDHD1	2.99144414	2.4801E-34	2.8521E-33
1704	NPAP1	2.9878947	0.01117876	0.01835574
1705	USP44	2.98706777	2.5382E-08	7.649E-08
1706	CLDN7	2.98667452	1.9176E-70	6.2267E-69
1707	DLEU7-AS1	2.98541916	0.01085872	0.0178701
1708	GPRC5B	2.98494031	1.243E-101	7.214E-100
1709	FAM69B	2.9846564	1.8287E-96	9.7328E-95
1710	OGFRL1	2.98444561	1.117E-23	8.407E-23
1711	LHFPL2	2.98210462	3.774E-154	4.501E-152
1712	PNPLA1	2.98172716	1.3465E-05	3.1885E-05
1713	LOC102723809	2.98141953	5.452E-05	0.00012111
1714	SLC52A1	2.97998243	9.355E-06	2.2466E-05
1715	ZNF525	2.97844957	2.653E-35	3.1562E-34
1716	TTC39A-AS1	2.97474596	0.00925772	0.0154189
1717	USP51	2.97396126	3.2707E-35	3.8671E-34
1718	KCNE2	2.97373119	4.5207E-05	0.00010147
1719	RASL12	2.97317429	1.0277E-22	7.3891E-22
1720	SLC7A6OS	2.9729995	7.1966E-55	1.5296E-53
1721	NREP	2.97133583	2.697E-75	9.6138E-74
1722	MITF	2.96683038	9.4348E-37	1.1806E-35
1723	KIAA1958	2.96670269	3.7071E-29	3.523E-28
1724	MYLK3	2.96659521	1.6339E-07	4.5995E-07
1725	CAPRIN2	2.96386817	5.2937E-35	6.2135E-34
1726	C3orf35	2.96159551	1.5266E-07	4.3113E-07
1727	NFKBIZ	2.96128465	7.0278E-66	2.0825E-64
1728	SEMA5B	2.96026465	0.00084673	0.00163769
1729	ZNF816	2.9600329	1.5959E-57	3.6706E-56
1730	MSS51	2.95732009	1.2197E-07	3.4767E-07
1731	RHOBTB3	2.95730893	1.8398E-61	4.7777E-60
1732	TMEM144	2.9571006	6.4855E-58	1.5151E-56
1733	SEC14L5	2.95666738	7.5389E-11	2.777E-10
1734	SLC25A24	2.9552293	5.378E-108	3.441E-106
1735	ADD3	2.95462337	8.2822E-75	2.9253E-73
1736	ZNF597	2.9544512	5.7659E-29	5.4527E-28
1737	PDLIM4	2.95437432	8.5942E-28	7.7698E-27
1738	SERTAD4	2.95364391	0.02490583	0.03856851
1739	SLMO1	2.95329496	1.1719E-05	2.7852E-05
1740	SPOCK3	2.952916	7.7447E-08	2.2393E-07
1741	LINC01279	2.95284299	2.5646E-13	1.1175E-12
1742	FLVCR1	2.95268018	3.8763E-56	8.6379E-55
1743	SLC22A17	2.95194334	4.8325E-11	1.8028E-10
1744	USP46-AS1	2.95066492	1.0724E-33	1.199E-32
1745	FGD6	2.94883914	1.2887E-42	1.9256E-41
1746	LINC00294	2.94832193	6.5119E-64	1.7973E-62
1747	F2RL1	2.94793954	1.0763E-94	5.5156E-93

1748	COL6A4P2	2.94634958	0.00334591	0.0059722
1749	TMEM98	2.94006899	4.5456E-24	3.4932E-23
1750	LINC00087	2.93560755	1.7941E-47	3.0729E-46
1751	COL7A1	2.93459856	0.0032243	0.00577165
1752	LOXL1	2.93361432	6.0547E-20	3.8091E-19
1753	KCNH8	2.92998557	4.2981E-06	1.0657E-05
1754	HRH2	2.92936726	4.0206E-19	2.4358E-18
1755	FAR2	2.92794832	2.3863E-14	1.1083E-13
1756	LINC00862	2.92729459	0.00213913	0.00392441
1757	FGF11	2.92689075	4.0898E-17	2.2264E-16
1758	MRAP2	2.92618921	0.00090348	0.00174169
1759	ZNF28	2.92608599	1.6741E-94	8.5338E-93
1760	GOLGA8A	2.9258767	1.1296E-32	1.2213E-31
1761	NR6A1	2.92337535	1.4565E-14	6.8599E-14
1762	TRPV5	2.92325396	0.00551747	0.00951254
1763	MYH7	2.91981052	0.01496986	0.02409345
1764	TBC1D26	2.91824477	0.0238627	0.03708691
1765	GRIK4	2.91758483	3.2655E-07	8.963E-07
1766	C11orf63	2.91752858	1.5451E-18	9.0955E-18
1767	FAM83C	2.91685581	0.00027257	0.00055937
1768	ACHE	2.91600678	1.0212E-17	5.7569E-17
1769	RAD54B	2.91216321	9.9793E-15	4.7441E-14
1770	NARR	2.9118463	2.8289E-35	3.3551E-34
1771	WFDC3	2.91097791	0.00026212	0.00053885
1772	COL4A2	2.9093119	2.4488E-69	7.7433E-68
1773	PFKFB4	2.90856049	7.8184E-53	1.5833E-51
1774	FBLN1	2.9078154	1.9911E-57	4.5687E-56
1775	TMEM158	2.90637214	7.174E-05	0.00015736
1776	ZFHX2	2.90360999	1.2815E-20	8.339E-20
1777	GRM6	2.90187764	0.02071853	0.03256708
1778	MTL5	2.90009676	9.4426E-16	4.7882E-15
1779	LOC100506071	2.89943619	6.9015E-19	4.1293E-18
1780	CELSR3	2.89801813	2.3252E-27	2.0674E-26
1781	CCDC108	2.89705876	0.01060649	0.01748442
1782	TENM3	2.89652074	2.2992E-13	1.0045E-12
1783	SPDEF	2.89612147	8.1727E-05	0.00017829
1784	HIF1A	2.89574535	3.6428E-33	4.0147E-32
1785	CENPE	2.89503836	4.141E-16	2.1374E-15
1786	CSRNP3	2.89486368	1.1688E-09	3.9272E-09
1787	NLGN1	2.89376847	5.4929E-06	1.3479E-05
1788	NRGN	2.89372101	2.2421E-14	1.0428E-13
1789	ICAM5	2.89309495	0.00025221	0.0005192
1790	IL12A	2.89282412	0.0018145	0.00336206
1791	MTHFD2	2.88704298	2.9865E-29	2.8508E-28
1792	STOX1	2.88649935	1.6036E-22	1.1407E-21

1793	LOC644554	2.88504025	0.00014248	0.00030231
1794	SH3RF2	2.8818991	7.487E-66	2.2151E-64
1795	ZNF251	2.88179888	1.4017E-68	4.3891E-67
1796	LOC101928489	2.88118387	0.03227771	0.04897938
1797	TFEC	2.87898339	8.8981E-07	2.3501E-06
1798	PLAGL2	2.87867245	3.4389E-66	1.0221E-64
1799	ENO2	2.87785225	1.1134E-12	4.65E-12
1800	SNORD97	2.8750987	0.0050762	0.00880677
1801	PAK1	2.87502018	1.7038E-97	9.2726E-96
1802	EPHB2	2.87120187	1.5013E-26	1.288E-25
1803	BCAM	2.87057066	2.0747E-48	3.6639E-47
1804	ENC1	2.8693235	4.3639E-16	2.2501E-15
1805	NCMAP	2.86921202	6.997E-38	9.0726E-37
1806	MST4	2.86810982	3.8011E-70	1.2219E-68
1807	MAP2K6	2.86671498	6.2812E-13	2.6612E-12
1808	SLC12A5	2.86304673	1.1867E-05	2.82E-05
1809	PKDREJ	2.86057183	3.6032E-05	8.1717E-05
1810	PLEKHG2	2.86034813	1.8978E-68	5.9234E-67
1811	MUSTN1	2.85775163	4.5385E-32	4.7993E-31
1812	OCLM	2.85694936	0.00441518	0.00773574
1813	SLC44A3	2.85487471	4.2811E-48	7.4851E-47
1814	REM2	2.85404349	0.0001088	0.00023405
1815	HIF1A-AS2	2.85324258	1.9259E-30	1.9121E-29
1816	MMD	2.8516374	2.5184E-56	5.6577E-55
1817	KRTAP5-2	2.85122283	7.2721E-06	1.7646E-05
1818	STAC	2.84977518	1.1452E-28	1.0714E-27
1819	C20orf196	2.84908139	2.6842E-34	3.0795E-33
1820	RFX3-AS1	2.84875824	0.00038917	0.00078289
1821	OSBPL10	2.84830538	8.5064E-55	1.8E-53
1822	BEX1	2.84515144	7.8358E-08	2.2639E-07
1823	SPTLC3	2.83836739	3.2584E-08	9.7465E-08
1824	LOC100996291	2.83776844	0.01883087	0.02982558
1825	TNFSF4	2.8373351	1.3443E-20	8.7386E-20
1826	LOC100128398	2.83414089	1.0758E-11	4.2058E-11
1827	ASIC1	2.83294436	1.3124E-06	3.4154E-06
1828	MGAM	2.83230987	0.00224396	0.00410542
1829	EPB41L1	2.83076993	2.4204E-67	7.3526E-66
1830	CYP1A1	2.82976537	1.2788E-07	3.6398E-07
1831	DLG3	2.82880824	2.9554E-49	5.3512E-48
1832	WTIP	2.82531597	1.3036E-50	2.4692E-49
1833	MARCKS	2.82118957	2.404E-117	1.793E-115
1834	FSBP	2.81927735	2.5048E-10	8.8646E-10
1835	SERPINA5	2.81274198	2.0974E-09	6.9055E-09
1836	SOX30	2.81121618	0.0139481	0.02256928
1837	GABRB2	2.80891032	0.02324086	0.03619617

1838	EMX2OS	2.80723796	7.9019E-08	2.282E-07
1839	LINC00964	2.80658065	0.01030772	0.01703115
1840	TNFRSF10C	2.80647589	1.0471E-44	1.6541E-43
1841	FAM160A1	2.80578215	9.1883E-39	1.2243E-37
1842	MICAL1	2.8050471	6.5313E-49	1.1705E-47
1843	LOC441666	2.80124213	6.9741E-10	2.3814E-09
1844	MPPED2	2.8003989	7.297E-05	0.00015992
1845	DKK1	2.7977536	7.046E-05	0.00015464
1846	OSBPL8	2.79760836	2.0359E-10	7.2517E-10
1847	LOC643339	2.79567522	0.01457554	0.02350776
1848	RAVER2	2.79344012	2.1426E-24	1.6699E-23
1849	NXN	2.7918358	1.577E-156	1.928E-154
1850	SGIP1	2.79168517	2.1479E-05	4.9841E-05
1851	CHRNA7	2.79025621	0.02635937	0.04064675
1852	SNTB2	2.7894369	8.9197E-24	6.7395E-23
1853	LOC101927604	2.7890286	0.01535841	0.0246818
1854	LINC00632	2.78897392	0.00050428	0.00100121
1855	LOC101929340	2.78862199	0.00023199	0.00047937
1856	SPTB	2.78776568	2.2658E-13	9.9039E-13
1857	BSPRY	2.78535624	2.4265E-23	1.8003E-22
1858	ZNF215	2.78534305	4.0317E-05	9.0975E-05
1859	FAM24B	2.78512779	8.8326E-07	2.3331E-06
1860	IMPG1	2.78292712	0.00183692	0.00339838
1861	ARHGAP26	2.78196209	1.1317E-55	2.479E-54
1862	MAP3K1	2.77373555	2.276E-106	1.414E-104
1863	DTNA	2.77361468	4.5306E-40	6.2567E-39
1864	FBXO2	2.77197027	2.4707E-50	4.6298E-49
1865	CCDC80	2.7705336	1.7277E-14	8.0957E-14
1866	FAM126A	2.76871736	1.3723E-55	2.9993E-54
1867	ZNF83	2.76573897	7.8512E-50	1.4474E-48
1868	ZNF701	2.76497055	9.1604E-44	1.4057E-42
1869	ARHGEF10	2.76269057	4.9444E-57	1.1252E-55
1870	MRC2	2.75801616	4.2196E-39	5.6692E-38
1871	CENPA	2.75735205	2.099E-17	1.1626E-16
1872	RAC3	2.7558068	8.2378E-43	1.2376E-41
1873	KIAA1045	2.75568457	9.8172E-05	0.00021218
1874	LRFN1	2.75481846	2.8166E-58	6.6444E-57
1875	ARID3B	2.75470784	6.3908E-76	2.3122E-74
1876	MXD1	2.75455946	3.028E-36	3.7215E-35
1877	ADAMTSL5	2.75352307	7.394E-38	9.5745E-37
1878	DOK6	2.7533056	7.1485E-11	2.6357E-10
1879	B3GALTL	2.75215095	5.6223E-08	1.6476E-07
1880	CYP2W1	2.75169815	0.00147133	0.00275767
1881	LOC81691	2.75106353	3.7605E-06	9.3746E-06
1882	TES	2.75022509	1.1457E-78	4.4268E-77

1883	TMEM51-AS1	2.74962831	2.1557E-41	3.1127E-40
1884	MERTK	2.74890722	3.7086E-36	4.555E-35
1885	JAG2	2.74876597	2.2344E-47	3.8168E-46
1886	RYR3	2.74872925	1.6536E-12	6.8135E-12
1887	TPM3P9	2.74841069	9.6049E-58	2.2331E-56
1888	SCTR	2.74768227	7.2818E-29	6.866E-28
1889	SPG20	2.74677005	5.4882E-32	5.7909E-31
1890	UAP1L1	2.74663257	4.5675E-53	9.3085E-52
1891	ALOX15B	2.74552108	1.9271E-05	4.4965E-05
1892	GPSM1	2.74487793	1.711E-20	1.107E-19
1893	STK17B	2.74387141	1.8254E-12	7.4925E-12
1894	ANKRD20A8P	2.74280338	0.00316801	0.0056801
1895	CD3D	2.74052553	1.3145E-12	5.4604E-12
1896	FILIP1	2.73916131	4.6983E-24	3.6035E-23
1897	COL28A1	2.73566226	1.5278E-05	3.5988E-05
1898	RAB11FIP1	2.73532262	7.9288E-43	1.1921E-41
1899	PIP5KL1	2.73513664	0.00135836	0.00255487
1900	LRP2	2.73490191	2.1199E-07	5.9151E-07
1901	ZCCHC18	2.73441351	0.00893055	0.01491385
1902	PDGFRL	2.73352962	4.4809E-08	1.324E-07
1903	PAPSS1	2.73318112	9.9193E-82	4.0516E-80
1904	LRP8	2.73001463	1.671E-09	5.5495E-09
1905	CDH2	2.72974233	6.868E-110	4.529E-108
1906	NKPD1	2.72823572	0.01968253	0.0310726
1907	GYPA	2.7280816	0.0117588	0.01925418
1908	FAM212B	2.726576	4.2801E-22	2.9971E-21
1909	ZNF878	2.72577517	0.02177913	0.03409687
1910	LEPREL4	2.72559661	7.2056E-26	5.9876E-25
1911	RCAN3	2.72507555	2.0647E-10	7.3529E-10
1912	CSF3R	2.72192583	8.8669E-18	5.0149E-17
1913	MEIS3	2.71545428	8.6385E-10	2.9337E-09
1914	LOC100288911	2.71450749	5.702E-12	2.2706E-11
1915	MREG	2.7144082	3.4881E-27	3.0786E-26
1916	PVRL4	2.71323819	0.00244508	0.00445399
1917	MOSPD1	2.71300707	9.213E-37	1.1536E-35
1918	ZNF600	2.71263202	1.5284E-44	2.4066E-43
1919	PLXNA1	2.71252205	3.5444E-45	5.6922E-44
1920	CNKSR2	2.71151313	3.2135E-18	1.861E-17
1921	LOC400043	2.71143418	6.8763E-05	0.0001511
1922	CCDC154	2.71019186	3.4162E-06	8.5516E-06
1923	CDK2	2.70977277	9.0806E-21	5.9652E-20
1924	IL17RD	2.70780706	1.2375E-12	5.1514E-12
1925	BMPR2	2.70684744	1.5724E-16	8.2867E-16
1926	ANGPT1	2.7056014	6.266E-16	3.2077E-15
1927	LMCD1	2.70408634	3.1487E-20	2.0144E-19

1928	HMGA1	2.70283717	1.3941E-21	9.5275E-21
1929	OSBP2	2.70257311	5.045E-19	3.0374E-18
1930	SV2C	2.70062168	3.1281E-11	1.1855E-10
1931	SRC	2.69827772	4.0502E-85	1.7784E-83
1932	DIO2	2.69772881	5.7722E-05	0.00012801
1933	DEFB1	2.69753716	3.2203E-43	4.8798E-42
1934	ZFP82	2.69743492	3.3294E-21	2.235E-20
1935	PTGS2	2.69585968	1.0859E-11	4.2444E-11
1936	JMJD1C-AS1	2.69574822	4.5251E-19	2.7329E-18
1937	ZNF876P	2.69119454	1.3237E-07	3.762E-07
1938	RASGEF1A	2.69074403	1.0254E-10	3.7372E-10
1939	HIC2	2.68923863	1.7805E-59	4.3598E-58
1940	BIN1	2.68547403	2.588E-121	2.101E-119
1941	MSANTD3-TMEFF1	2.6818818	1.0996E-29	1.0643E-28
1942	CDC42EP2	2.68146478	5.9136E-66	1.755E-64
1943	LOC101927795	2.67900517	6.8205E-28	6.1923E-27
1944	KDM5B	2.67878525	3.7039E-81	1.4846E-79
1945	ARHGEF16	2.67798297	1.193E-85	5.3355E-84
1946	STK33	2.67772136	1.9378E-05	4.5194E-05
1947	TMEM120B	2.677055	3.097E-147	3.38E-145
1948	FAM189A2	2.6753715	8.7757E-08	2.5257E-07
1949	PBX1	2.6719526	9.0857E-10	3.079E-09
1950	FZD6	2.6711661	5.1525E-56	1.1442E-54
1951	LOX	2.66984591	1.1261E-07	3.2149E-07
1952	B4GALT4	2.66912378	7.6131E-45	1.2096E-43
1953	SERPINI1	2.66899231	5.03E-19	3.0293E-18
1954	RHOBTB1	2.66729814	2.109E-59	5.1512E-58
1955	RAB3D	2.66683723	1.9838E-42	2.9528E-41
1956	TUFT1	2.66657804	3.8113E-46	6.2935E-45
1957	GLCCI1	2.66538068	2.1706E-15	1.0766E-14
1958	AP3B2	2.66479104	8.8997E-06	2.1413E-05
1959	LOC102723703	2.66414334	0.0001105	0.0002376
1960	FGF13	2.66384319	1.9118E-14	8.9323E-14
1961	LOC100128288	2.66342173	5.5428E-12	2.2093E-11
1962	TMC3	2.66269694	1.9843E-10	7.0757E-10
1963	PRKAA2	2.65924065	1.3384E-44	2.1108E-43
1964	LOC101927746	2.6564214	3.7815E-06	9.4245E-06
1965	LOC644919	2.65629973	2.5078E-06	6.3591E-06
1966	USP27X	2.65429866	1.045E-33	1.1691E-32
1967	PLBD1-AS1	2.65285681	2.4189E-07	6.7108E-07
1968	MAP3K9	2.65035849	5.7694E-45	9.2119E-44
1969	SNORA60	2.65033677	0.00813542	0.01368656
1970	LOC100130705	2.64955346	0.00261373	0.00474109
1971	GATS	2.64908682	2.1624E-80	8.5961E-79

1972	GPR39	2.64783751	3.2708E-37	4.1546E-36
1973	IGFBPL1	2.64684438	0.02246953	0.03510807
1974	SLC29A2	2.64441608	9.4961E-38	1.2239E-36
1975	GREM1	2.64425061	2.5349E-06	6.4238E-06
1976	KLF3-AS1	2.64317041	1.2761E-14	6.0382E-14
1977	FAM71F2	2.64205253	2.2837E-08	6.9046E-08
1978	C19orf81	2.64076143	0.02499473	0.03869377
1979	DBNDD1	2.63988699	7.9342E-37	9.9538E-36
1980	TMEM74B	2.63920826	3.5695E-06	8.9239E-06
1981	ABR	2.63816228	7.7716E-36	9.4314E-35
1982	GPR143	2.63705049	9.7223E-12	3.8108E-11
1983	ZNF137P	2.6347843	4.0332E-18	2.3177E-17
1984	CCDC114	2.6260495	0.01521576	0.02446475
1985	TRIM59	2.62592592	1.5644E-12	6.4638E-12
1986	AGL	2.6256614	5.6945E-13	2.418E-12
1987	KCNQ1OT1	2.62250325	7.4529E-35	8.7003E-34
1988	CDC14A	2.61968399	2.9926E-31	3.0656E-30
1989	PTPRR	2.61725604	0.00482257	0.00839615
1990	THSD7A	2.61584378	8.7531E-19	5.1986E-18
1991	PPP3CA	2.61064324	1.6644E-57	3.8235E-56
1992	EPB41L4A	2.60918912	8.846E-62	2.3221E-60
1993	LLGL1	2.60780521	5.2763E-48	9.2084E-47
1994	FBXL19-AS1	2.60754555	8.0815E-25	6.4385E-24
1995	SH3D21	2.60657526	1.2082E-07	3.4447E-07
1996	LOC102467147	2.60612415	0.01005817	0.01665015
1997	ZNF608	2.6045246	3.4868E-13	1.5034E-12
1998	CTNNA2	2.60350272	0.02880667	0.04411071
1999	VCAM1	2.60302787	0.0006556	0.00128513
2000	DLG1-AS1	2.60273868	6.5794E-06	1.6046E-05
2001	LEPREL2	2.60145901	9.5491E-12	3.7475E-11
2002	USP54	2.59989732	5.1384E-40	7.0758E-39
2003	SGOL2	2.59880957	1.9479E-15	9.6841E-15
2004	BEND5	2.59854028	1.0131E-06	2.6645E-06
2005	COL26A1	2.59838013	8.3933E-11	3.0805E-10
2006	GGN	2.59824436	0.00058798	0.00115893
2007	SLC5A12	2.59785017	0.00053418	0.00105742
2008	NOTCH3	2.59596261	4.7511E-26	3.9857E-25
2009	APOBEC2	2.59496864	1.6352E-05	3.8431E-05
2010	TAS2R5	2.5932645	0.00039312	0.00079058
2011	RASL11B	2.59277213	1.306E-22	9.3455E-22
2012	CAPN2	2.59259425	3.3868E-39	4.5662E-38
2013	LAMB1	2.59178836	8.7435E-36	1.0598E-34
2014	PHLDA3	2.59142207	3.2566E-51	6.317E-50
2015	CNR1	2.59082836	0.00079627	0.00154488
2016	AP1S3	2.5896919	3.0163E-36	3.7094E-35

2017	NR4A3	2.58955266	0.00241915	0.00440841
2018	LOC729603	2.58884989	3.4321E-10	1.2019E-09
2019	LINC01004	2.58853493	2.9041E-08	8.707E-08
2020	MYRF	2.58710801	8.079E-132	7.362E-130
2021	SLFNL1	2.58578298	4.0611E-07	1.1048E-06
2022	HAVCR1P1	2.58315296	0.02704594	0.04162903
2023	ATP1B3	2.58048035	1.6952E-59	4.1669E-58
2024	SPATA32	2.58009766	0.00217614	0.00398927
2025	SH3GL3	2.57846536	0.00768961	0.01298744
2026	LPHN1	2.57729793	2.0225E-44	3.1716E-43
2027	MLLT4-AS1	2.57328861	1.1004E-11	4.2993E-11
2028	NPTN-IT1	2.57307259	2.1299E-13	9.3351E-13
2029	ATP1A1	2.57279239	1.6475E-45	2.6726E-44
2030	PDE7A	2.57108901	1.6186E-56	3.649E-55
2031	SLC28A2	2.57047459	0.00232902	0.00425219
2032	SEMA3B-AS1	2.57014314	0.0007767	0.00150828
2033	ZNF404	2.56999417	1.3884E-11	5.3874E-11
2034	TRIM45	2.56859474	2.4352E-13	1.062E-12
2035	EFS	2.56797357	2.4334E-05	5.6141E-05
2036	LIF	2.56717808	7.5331E-11	2.7753E-10
2037	KIF3C	2.5662696	1.2157E-24	9.6101E-24
2038	GREB1L	2.56283856	1.0127E-19	6.3136E-19
2039	LOC728392	2.56197716	8.2573E-08	2.3821E-07
2040	CLMP	2.56177828	2.3632E-07	6.5617E-07
2041	THRB	2.56125953	1.2209E-72	4.1821E-71
2042	ARL13A	2.56090158	0.00938991	0.01562709
2043	SPRED3	2.5578615	5.7593E-06	1.4103E-05
2044	SCAI	2.5557517	5.5283E-11	2.052E-10
2045	LRRN4	2.55570989	1.4625E-07	4.139E-07
2046	DEPTOR	2.55536355	5.6095E-26	4.6875E-25
2047	VGLL1	2.55469771	0.02041496	0.03213173
2048	ZNF439	2.5546421	1.04E-13	4.6435E-13
2049	TNFRSF9	2.55353606	9.304E-05	0.00020156
2050	C14orf178	2.55317136	0.00221888	0.00406261
2051	ZNF682	2.55281633	2.054E-15	1.0204E-14
2052	SPEG	2.54832659	1.936E-16	1.0153E-15
2053	EZR	2.54663628	1.862E-113	1.284E-111
2054	ZNF221	2.54648965	5.4835E-14	2.4975E-13
2055	ZNF853	2.54541663	3.5354E-09	1.1437E-08
2056	SNORD22	2.54536116	3.6321E-10	1.2698E-09
2057	CASR	2.54392271	0.00036532	0.0007372
2058	TRIM46	2.54384255	7.0299E-07	1.8695E-06
2059	SMOX	2.54238915	2.6729E-85	1.179E-83
2060	CLHC1	2.5423642	5.2665E-20	3.3273E-19
2061	PARD6B	2.54121139	1.9553E-25	1.5912E-24

2062	SH3PXD2B	2.54065375	2.7193E-39	3.679E-38
2063	EDA	2.53782153	5.721E-51	1.0976E-49
2064	GPR141	2.53719748	1.8269E-05	4.2732E-05
2065	SMAP1	2.53647041	9.6242E-36	1.1629E-34
2066	ZNF468	2.53602557	2.6952E-48	4.7338E-47
2067	LOC653602	2.53498726	4.0991E-06	1.0183E-05
2068	NAT8L	2.53489199	2.1598E-07	6.0203E-07
2069	ASNS	2.53424424	1.5502E-26	1.3282E-25
2070	PMEPA1	2.53355124	1.2908E-12	5.3676E-12
2071	MYZAP	2.53350407	1.2971E-36	1.6105E-35
2072	LINC00641	2.5325352	5.5261E-11	2.0516E-10
2073	AQP4-AS1	2.53132446	0.00235988	0.00430446
2074	MRPS6	2.53012169	2.9224E-27	2.59E-26
2075	LOC648987	2.52989494	6.3096E-28	5.7392E-27
2076	MAPKBP1	2.52792766	4.8039E-17	2.6041E-16
2077	C22orf23	2.52684563	1.0355E-29	1.0043E-28
2078	ALPK3	2.52594323	3.1527E-50	5.8794E-49
2079	SNORD116-4	2.52579737	4.1716E-05	9.3999E-05
2080	MBOAT4	2.52397348	0.01001522	0.01658757
2081	TEX19	2.52358173	0.02832681	0.04342756
2082	ST8SIA4	2.52262157	9.5581E-10	3.2346E-09
2083	ADAM19	2.52251343	2.6747E-49	4.8566E-48
2084	FAM127C	2.52160975	2.8278E-39	3.8204E-38
2085	TENM2	2.5202211	0.01042759	0.01721595
2086	THOC2	2.51765629	6.7375E-38	8.7479E-37
2087	CDCA7	2.51360463	0.0013583	0.00255487
2088	STX1A	2.51280666	1.8932E-16	9.9337E-16
2089	PTGES3L	2.5097497	1.2353E-09	4.1406E-09
2090	ISYNA1	2.50661809	7.7519E-29	7.2986E-28
2091	SLC6A17	2.50554819	0.03136463	0.04771751
2092	ANXA8L1	2.5037552	0.00100134	0.00191714
2093	PCAT6	2.50286319	4.2272E-08	1.2511E-07
2094	IGLON5	2.50226774	0.00049039	0.00097453
2095	DUSP4	2.50214076	6.1298E-10	2.1046E-09
2096	BLM	2.50136945	9.8158E-09	3.0607E-08
2097	LRRC9	2.5009731	0.0043188	0.00758125
2098	CNTRL	2.4994988	9.1951E-11	3.3646E-10
2099	GPC6	2.49740893	4.7681E-15	2.3175E-14
2100	PCDHAC2	2.49735948	3.0498E-31	3.1225E-30
2101	C6orf211	2.49682391	2.0617E-50	3.8673E-49
2102	DHDH	2.49507949	0.00245793	0.00447401
2103	TSPEAR	2.4933091	7.2974E-05	0.00015992
2104	FLJ44635	2.49218981	1.1814E-12	4.9242E-12
2105	ANKS1B	2.49049304	2.6888E-34	3.083E-33
2106	TLL1	2.49043537	0.00018522	0.00038782

2107	ZMAT3	2.48990941	6.4833E-88	2.9823E-86
2108	ICA1	2.4884942	1.5849E-26	1.3573E-25
2109	ZNF547	2.48657976	1.3473E-14	6.3625E-14
2110	ANKRD53	2.48330014	2.24E-09	7.3586E-09
2111	BIK	2.48181809	0.00028267	0.00057919
2112	CORO2A	2.48146422	1.0331E-75	3.7168E-74
2113	CXCR5	2.48034476	4.1599E-05	9.3747E-05
2114	SATB2	2.47985809	2.1094E-29	2.0265E-28
2115	LRRC75A	2.47960517	4.2434E-24	3.2676E-23
2116	CCDC64B	2.47863223	2.9412E-12	1.1915E-11
2117	ADAM9	2.47749185	6.9501E-84	2.9839E-82
2118	SNX30	2.47477398	3.2923E-30	3.2419E-29
2119	ARHGAP18	2.474044	7.8601E-26	6.5119E-25
2120	C6orf164	2.4736802	0.00394722	0.00696887
2121	KLRAP1	2.47322921	5.2942E-07	1.4248E-06
2122	HAGHL	2.47270531	2.1088E-09	6.9406E-09
2123	PARPBP	2.47053042	1.8319E-26	1.5647E-25
2124	ATP8B2	2.47051713	2.7862E-49	5.0496E-48
2125	ZNF14	2.4695718	2.2575E-16	1.1794E-15
2126	ARL4C	2.46876235	2.5765E-31	2.6506E-30
2127	SPOCD1	2.46814788	4.3352E-06	1.0748E-05
2128	PLK2	2.46767159	1.567E-10	5.6263E-10
2129	GABRB3	2.46586543	2.3511E-07	6.5302E-07
2130	KCNN1	2.46556091	0.00135102	0.00254194
2131	MDGA2	2.46382933	0.00248635	0.00452447
2132	RNF157-AS1	2.46302523	2.1417E-12	8.7572E-12
2133	WBP5	2.46213659	5.015E-127	4.384E-125
2134	GSTA2	2.46202352	1.5834E-24	1.2425E-23
2135	ZFP28	2.45602541	9.234E-33	1.0011E-31
2136	TMC5	2.45586607	3.6452E-11	1.3728E-10
2137	SNX27	2.45309716	3.268E-31	3.3441E-30
2138	NUDT18	2.44969465	1.6029E-17	8.9294E-17
2139	CENPQ	2.44932834	1.3555E-26	1.167E-25
2140	ATAD5	2.44823398	1.1428E-19	7.1156E-19
2141	PTAR1	2.44792428	3.4187E-55	7.3634E-54
2142	DEPDC1B	2.4441451	1.6622E-06	4.287E-06
2143	PCDHB9	2.44321943	0.00869978	0.01455488
2144	PKDCC	2.44216284	2.6076E-31	2.6797E-30
2145	NPAS1	2.44100928	2.039E-07	5.6945E-07
2146	KIT	2.44042879	3.9327E-07	1.0727E-06
2147	ZNF518B	2.44030135	8.5269E-84	3.6206E-82
2148	XRCC2	2.43912454	3.1379E-08	9.3947E-08
2149	HBEGF	2.43531023	1.7302E-10	6.1926E-10
2150	SPIN4	2.43475304	7.3072E-39	9.7699E-38
2151	PAQR8	2.43369099	1.7485E-17	9.7129E-17

2152	PPP2R2B	2.4333469	0.00030318	0.00061919
2153	COLEC12	2.43318275	1.0186E-05	2.4361E-05
2154	NMRK2	2.43314638	4.3928E-05	9.8753E-05
2155	SEMA6C	2.43289794	2.0428E-11	7.8433E-11
2156	ATL1	2.4327288	8.709E-12	3.422E-11
2157	AIF1L	2.43224932	3.2747E-12	1.3214E-11
2158	LINC01160	2.43130028	0.00837178	0.01404993
2159	LGALSL	2.4289552	5.0063E-30	4.9023E-29
2160	RASSF8	2.42819113	1.528E-77	5.7434E-76
2161	SLC9A5	2.42653225	0.00013877	0.00029495
2162	MARK1	2.42581676	4.6561E-08	1.374E-07
2163	CHAF1B	2.425159	1.2521E-17	7.0112E-17
2164	PLA2G4F	2.42376631	0.00887823	0.01483159
2165	LNX1	2.41972695	1.0807E-57	2.5005E-56
2166	STAT4	2.41875834	0.00013243	0.00028232
2167	FABP5	2.41750291	4.4446E-12	1.7819E-11
2168	COL4A2-AS1	2.41718958	4.1087E-46	6.7673E-45
2169	ZNF229	2.4169226	5.5979E-32	5.9035E-31
2170	CENPI	2.41461794	1.2376E-10	4.4852E-10
2171	YPEL2	2.41290563	1.4993E-15	7.5118E-15
2172	ANKS1A	2.4120671	1.7439E-10	6.2403E-10
2173	ZNF613	2.41192001	4.4983E-25	3.6226E-24
2174	HSPBAP1	2.41056733	2.3695E-18	1.3822E-17
2175	OAT	2.4078034	5.3049E-33	5.8003E-32
2176	RAD54L	2.40776913	7.8127E-10	2.6579E-09
2177	POMC	2.40744288	3.6666E-06	9.15E-06
2178	BOLA3-AS1	2.40742278	3.5882E-10	1.2547E-09
2179	MTFR2	2.40658071	5.5443E-10	1.9114E-09
2180	CDH4	2.40573244	5.925E-06	1.4497E-05
2181	MYLIP	2.4048088	3.6452E-15	1.7825E-14
2182	PMAIP1	2.40343418	5.1104E-05	0.00011397
2183	AMH	2.40196617	0.01041892	0.0172031
2184	TULP2	2.40013378	0.00517312	0.00895964
2185	GNRHR	2.39899058	0.01103913	0.01814806
2186	CDK5RAP2	2.39895712	3.2733E-15	1.6063E-14
2187	ZNF117	2.39891598	3.6262E-31	3.7009E-30
2188	SMIM22	2.39815762	0.00033898	0.00068761
2189	NACAD	2.39780422	4.1356E-14	1.8992E-13
2190	SLC4A11	2.3968177	5.1065E-06	1.2568E-05
2191	DLG5-AS1	2.39671622	0.00018384	0.00038502
2192	ZNF347	2.39343226	1.3794E-38	1.8291E-37
2193	CA3	2.39315445	7.2226E-14	3.2626E-13
2194	ZFAS1	2.39165545	1.9214E-50	3.6111E-49
2195	ADD3-AS1	2.39138501	2.1632E-12	8.8414E-12
2196	GRAMD1A	2.39075635	2.7539E-40	3.836E-39

2197	CDK18	2.39020273	1.2572E-23	9.4251E-23
2198	NYNRIN	2.38793332	4.0115E-11	1.5052E-10
2199	ZDHHC13	2.38717413	3.0266E-50	5.6497E-49
2200	USP9Y	2.38600496	1.0108E-59	2.5004E-58
2201	ZNF528	2.3853502	2.8568E-65	8.25E-64
2202	ZNF765	2.38424075	1.1641E-30	1.1629E-29
2203	ARID5B	2.38394071	3.8488E-28	3.5359E-27
2204	ZNF626	2.38299411	2.0934E-28	1.9435E-27
2205	ASB9P1	2.38256919	0.02708662	0.04168168
2206	ATP1B1	2.3822447	3.0819E-28	2.8449E-27
2207	SCN3B	2.38056062	1.1944E-09	4.0112E-09
2208	PTPRM	2.3798657	2.1827E-18	1.2744E-17
2209	ANKMY2	2.37646324	5.9778E-19	3.5889E-18
2210	SPAG5	2.37612774	1.6958E-10	6.0727E-10
2211	KIAA1804	2.37459988	6.0175E-15	2.9028E-14
2212	CYP4X1	2.37444559	0.00829428	0.01393317
2213	CCNG2	2.37248242	4.8582E-40	6.6948E-39
2214	LOC100129175	2.37239598	0.0036473	0.00647842
2215	RCN2	2.37194532	1.9809E-45	3.1999E-44
2216	DUSP8	2.37141371	1.0037E-18	5.9521E-18
2217	PXYLP1	2.37061453	1.0104E-14	4.7998E-14
2218	EPHB3	2.36994737	1.1337E-28	1.0612E-27
2219	PKN3	2.36799036	1.277E-50	2.4239E-49
2220	GPR157	2.36665206	1.8575E-35	2.2235E-34
2221	SLC6A9	2.36647662	8.7337E-17	4.6689E-16
2222	KITLG	2.36613333	7.534E-12	2.973E-11
2223	SFRP1	2.36534235	4.497E-10	1.5615E-09
2224	TMED8	2.36357531	6.2787E-19	3.7625E-18
2225	MAATS1	2.36229417	0.00156629	0.00292572
2226	CHRNA10	2.36015133	9.9865E-05	0.00021557
2227	GJC1	2.359914	1.0346E-10	3.7678E-10
2228	MSI2	2.35869973	6.5569E-60	1.6346E-58
2229	GRB7	2.35792412	6.874E-32	7.2295E-31
2230	COL4A1	2.35765893	1.2294E-25	1.0103E-24
2231	GNRH1	2.35744111	2.4527E-06	6.2251E-06
2232	KIF18A	2.35621536	8.785E-11	3.2194E-10
2233	B3GALT2	2.35439061	0.00042354	0.00084884
2234	NT5DC2	2.35371015	2.3747E-29	2.2757E-28
2235	PAQR6	2.35288022	4.8398E-13	2.0655E-12
2236	PRPF39	2.34851028	1.7618E-24	1.3775E-23
2237	RPS15AP10	2.3463749	0.02099129	0.03296097
2238	LOC100130331	2.34570498	0.01336423	0.0216954
2239	ZSCAN12P1	2.34551769	1.3198E-12	5.4802E-12
2240	FNBP1L	2.34513794	4.6722E-59	1.1283E-57
2241	FAM46C	2.34500572	8.1616E-17	4.3667E-16

2242	MEX3C	2.34383719	1.3568E-23	1.0156E-22
2243	SUSD3	2.34226857	3.0377E-15	1.4941E-14
2244	SLC16A10	2.33997573	4.1959E-36	5.1502E-35
2245	FAM65B	2.33964366	7.122E-16	3.6344E-15
2246	SLC19A3	2.33824739	8.6416E-73	2.9655E-71
2247	HSPB8	2.3380491	2.1145E-11	8.1089E-11
2248	SDHAP3	2.33730248	2.1505E-24	1.6753E-23
2249	ARHGEF4	2.33547172	9.1701E-08	2.6352E-07
2250	FAR1	2.3336662	2.9857E-24	2.3111E-23
2251	LOC100506606	2.33125041	1.3298E-06	3.4582E-06
2252	ZNF124	2.3305413	6.9086E-34	7.7601E-33
2253	TNFSF11	2.33052061	0.02522431	0.03900541
2254	ZNF432	2.32949601	6.9783E-38	9.0545E-37
2255	DACT2	2.32932515	6.6181E-15	3.1854E-14
2256	ZNF334	2.32906612	1.2761E-11	4.9706E-11
2257	ZGRF1	2.32809833	1.6289E-12	6.7217E-12
2258	SLC4A7	2.32564944	2.5455E-07	7.0458E-07
2259	PCDHB8	2.32402734	0.00535147	0.0092478
2260	WNK3	2.32283096	1.6373E-19	1.0132E-18
2261	KPNA5	2.32274663	7.4847E-16	3.8144E-15
2262	SEMA4D	2.32107471	1.0733E-29	1.0399E-28
2263	EBF4	2.32069601	2.871E-07	7.9137E-07
2264	LOC399815	2.3205522	0.01262973	0.0205739
2265	FAM174B	2.32027209	3.2678E-17	1.79E-16
2266	SPATA17	2.31946472	0.00738568	0.01250801
2267	MAP1B	2.31703577	3.5467E-11	1.337E-10
2268	MMS22L	2.31600441	6.2403E-15	3.0072E-14
2269	PRICKLE2-AS1	2.31451567	1.0513E-21	7.2308E-21
2270	PDGFA	2.31391492	3.1359E-21	2.1073E-20
2271	PYGB	2.31196132	1.4639E-58	3.4874E-57
2272	CABYR	2.31120351	3.9459E-07	1.0759E-06
2273	KDM6B	2.31035816	7.7862E-76	2.8118E-74
2274	PMFBP1	2.30929061	0.00487138	0.00847808
2275	CDCP1	2.30893405	3.9545E-10	1.3776E-09
2276	ARNT2	2.30753319	1.3262E-06	3.45E-06
2277	PODXL	2.30680298	4.2991E-08	1.2718E-07
2278	LOC101927811	2.30641231	0.00067215	0.00131584
2279	FAM171B	2.30618564	4.9412E-11	1.8419E-10
2280	ZNF818P	2.30355282	1.055E-32	1.1413E-31
2281	ZNF549	2.3031135	5.9107E-31	5.9694E-30
2282	GPRIN3	2.30181192	3.9923E-14	1.8338E-13
2283	GDPD1	2.30160746	1.5659E-12	6.4684E-12
2284	LOC102723385	2.3007335	0.00096404	0.00185142
2285	CHIA	2.30045212	0.02038345	0.03208521
2286	TCP11L1	2.29942804	3.2801E-55	7.0727E-54

2287	TSPAN18	2.2987594	7.2237E-28	6.5522E-27
2288	RNFT2	2.29751982	3.9468E-05	8.9153E-05
2289	RPS6KA5	2.29609462	6.3244E-23	4.5918E-22
2290	GDF15	2.29585207	1.6923E-25	1.3813E-24
2291	NDN	2.29472392	1.6117E-71	5.3781E-70
2292	RAP1GAP2	2.29464033	7.4118E-09	2.3333E-08
2293	ZNF454	2.28973271	2.6389E-07	7.2896E-07
2294	LOC441178	2.2888468	0.00527618	0.00912423
2295	CENPH	2.28855039	1.3657E-12	5.661E-12
2296	MTA3	2.28709882	1.0159E-69	3.2388E-68
2297	PPP1CC	2.28706472	3.048E-41	4.3913E-40
2298	PDE4D	2.28573843	7.102E-23	5.1428E-22
2299	GATA6-AS1	2.2836993	3.4433E-07	9.4373E-07
2300	BEX4	2.28176336	1.7234E-66	5.1541E-65
2301	PRKCD	2.27897492	1.5948E-50	3.003E-49
2302	STARD4	2.27854105	2.3111E-32	2.4683E-31
2303	PRSS23	2.27770912	1.517E-17	8.4632E-17
2304	SNORD31	2.27671773	7.206E-06	1.7505E-05
2305	FAM198B	2.27383264	1.3882E-27	1.2445E-26
2306	PTP4A3	2.27377685	4.8146E-13	2.0552E-12
2307	TRPV3	2.27119332	4.7728E-34	5.4082E-33
2308	CCDC19	2.27019725	0.01077719	0.01774462
2309	LOC101927911	2.26780728	0.00048268	0.00095999
2310	CILP2	2.26740554	2.54E-06	6.4357E-06
2311	GUCY2EP	2.2628892	0.00043434	0.00086931
2312	DLG5	2.26149523	1.937E-76	7.1146E-75
2313	EDN1	2.26094984	1.9217E-20	1.2417E-19
2314	IKZF2	2.25989436	2.4473E-08	7.3855E-08
2315	WNT5B	2.25803596	5.6335E-07	1.5131E-06
2316	ZNF483	2.25607032	1.6963E-11	6.5442E-11
2317	PRICKLE2	2.25321102	2.3597E-30	2.3391E-29
2318	ITPRIPL1	2.24706122	6.479E-08	1.888E-07
2319	NDRG4	2.24601531	1.3059E-24	1.0303E-23
2320	MSX1	2.24592888	1.4621E-05	3.4495E-05
2321	DYNC2H1	2.24472244	9.8441E-14	4.4035E-13
2322	CXorf57	2.24398308	0.00160547	0.00299456
2323	FAM19A5	2.24353307	2.6422E-05	6.0741E-05
2324	KIF5C	2.2435214	5.6633E-05	0.00012562
2325	DKK3	2.2405149	1.1787E-35	1.4206E-34
2326	TNFRSF21	2.24042248	2.4906E-33	2.7559E-32
2327	RFX3	2.23915402	4.8508E-12	1.9403E-11
2328	PWARSN	2.23804905	2.5193E-08	7.5933E-08
2329	DHX32	2.2369836	7.3839E-31	7.4223E-30
2330	TOB2P1	2.23611811	1.1641E-05	2.7686E-05
2331	OCLN	2.23606431	2.4649E-30	2.4384E-29

2332	DNAJC15	2.2357278	5.2584E-20	3.3232E-19
2333	SLC4A5	2.23313602	5.3482E-08	1.5703E-07
2334	BICC1	2.23272281	1.3515E-10	4.8804E-10
2335	RBM11	2.23195741	0.001288	0.00243152
2336	MYSM1	2.23123876	5.425E-49	9.7317E-48
2337	CCDC14	2.23087077	1.1194E-23	8.4219E-23
2338	ZNF625	2.23070385	1.9737E-09	6.516E-09
2339	PTPDC1	2.23017843	7.1665E-24	5.4446E-23
2340	TIGD4	2.2301051	0.03172818	0.04821746
2341	B3GALNT1	2.22977985	1.0588E-09	3.5707E-09
2342	ZNF283	2.2283126	3.959E-18	2.2771E-17
2343	8-Sep	2.22830447	3.8751E-72	1.3112E-70
2344	SOCS2	2.22811677	2.2629E-36	2.7936E-35
2345	DUSP26	2.2278849	0.00436252	0.00765313
2346	SMIM1	2.22697044	1.1864E-31	1.2376E-30
2347	CYSRT1	2.22613509	8.8124E-06	2.121E-05
2348	NQO1	2.22574572	7.1703E-08	2.0797E-07
2349	PROCA1	2.22573679	0.00119583	0.0022657
2350	PARD3B	2.22507821	3.1785E-34	3.625E-33
2351	CKMT2	2.22472593	1.726E-08	5.2706E-08
2352	CCND2	2.2240498	0.00135764	0.00255401
2353	LOC100287846	2.22331648	0.00048141	0.00095776
2354	PRR19	2.22303104	6.5594E-18	3.7306E-17
2355	LOC100996634	2.22300722	7.5926E-06	1.8394E-05
2356	TSPAN13	2.22257346	7.1752E-92	3.4743E-90
2357	PLEKHA5	2.22238075	3.1876E-51	6.1894E-50
2358	SGTB	2.22195481	3.0314E-21	2.0392E-20
2359	ZNF329	2.22181025	4.1068E-42	6.0661E-41
2360	TMEM240	2.22177728	0.0004732	0.00094221
2361	TUSC3	2.22149715	5.8789E-08	1.7207E-07
2362	EMC3-AS1	2.21965896	1.9548E-07	5.4671E-07
2363	GPR56	2.21851341	1.2029E-13	5.3386E-13
2364	GNPDA1	2.21829301	3.9402E-41	5.643E-40
2365	ZNF211	2.21743659	9.3011E-36	1.1259E-34
2366	MAPRE1	2.21703669	3.4491E-41	4.9582E-40
2367	HELLS	2.21676801	4.7084E-10	1.6317E-09
2368	RASGRP1	2.21635177	4.3591E-10	1.5141E-09
2369	TEX22	2.21507322	0.01076294	0.01772568
2370	LINC00854	2.21497369	4.4097E-05	9.9064E-05
2371	N4BP2	2.21496606	4.0484E-24	3.1211E-23
2372	GNB3	2.21447016	0.00141776	0.00266192
2373	PLCG1	2.21421867	2.228E-19	1.3708E-18
2374	C11orf84	2.21406269	3.5602E-46	5.9042E-45
2375	ICA1L	2.21382525	2.4314E-10	8.6162E-10
2376	ZNF532	2.21267647	2.974E-37	3.7876E-36

2377	TTK	2.21197397	2.4624E-07	6.8221E-07
2378	KIF3A	2.21084559	7.4152E-32	7.7945E-31
2379	RPL13P5	2.21057464	2.2592E-10	8.0264E-10
2380	MAML2	2.20968517	3.0234E-06	7.6078E-06
2381	COL9A2	2.20947535	2.8495E-08	8.5511E-08
2382	SGK223	2.20817674	2.5722E-38	3.3875E-37
2383	ASH2L	2.20752735	1.64E-16	8.6313E-16
2384	GPR114	2.2075273	9.5988E-10	3.2467E-09
2385	GNB4	2.20511843	3.5697E-12	1.4371E-11
2386	RPL41	2.2034346	1.8349E-48	3.2463E-47
2387	LDHAL6A	2.20114728	0.00651786	0.01112609
2388	ORC2	2.19995063	9.6522E-44	1.48E-42
2389	SCN5A	2.19750969	0.01068888	0.01761122
2390	GAL3ST1	2.19711729	1.8025E-21	1.2228E-20
2391	TP53INP1	2.19628457	1.5792E-06	4.0827E-06
2392	PSPH	2.19622649	2.082E-61	5.3991E-60
2393	CLUL1	2.19440246	0.0025095	0.00456277
2394	NBEAL2	2.19062516	2.1332E-17	1.1802E-16
2395	LOC100131347	2.19037388	0.00336451	0.00600373
2396	FAM83F	2.18980903	1.0536E-07	3.0146E-07
2397	OFD1	2.18912619	3.9938E-21	2.6681E-20
2398	TBRG1	2.18866238	2.7733E-36	3.4149E-35
2399	ASPH	2.18767922	9.9237E-52	1.9544E-50
2400	FRMD6	2.18659952	7.3603E-19	4.3984E-18
2401	LRRC16A	2.18659076	5.4513E-34	6.1518E-33
2402	PHKA1	2.1851721	9.4417E-36	1.1422E-34
2403	FAM150B	2.18470705	8.2008E-05	0.00017883
2404	SMAD3	2.1841041	1.5958E-55	3.4759E-54
2405	GATA3	2.18374154	2.7486E-18	1.5971E-17
2406	MLLT3	2.18356213	4.9205E-13	2.0981E-12
2407	SNHG18	2.18355024	1.7558E-14	8.2237E-14
2408	TUBA3D	2.17944972	0.00036735	0.00074107
2409	NTNG2	2.17905618	0.00577794	0.0099306
2410	DESI2	2.17865642	2.1912E-21	1.4812E-20
2411	AFAP1L2	2.17857222	1.0411E-15	5.2612E-15
2412	OXCT1	2.17784507	2.4275E-18	1.4148E-17
2413	THOC5	2.17753554	5.6578E-24	4.3256E-23
2414	SNHG11	2.17627264	5.3197E-10	1.8366E-09
2415	CENPJ	2.17578357	2.5883E-12	1.0521E-11
2416	NFAT5	2.17529986	2.6934E-58	6.3693E-57
2417	SGK1	2.17375846	6.2152E-17	3.3466E-16
2418	GABRE	2.17368609	0.00019865	0.00041407
2419	CCDC181	2.17358678	0.03196565	0.04853252
2420	LOXL4	2.17354644	1.3538E-07	3.844E-07
2421	PELI2	2.17345149	1.2186E-21	8.3607E-21

2422	PALMD	2.17186749	0.01520293	0.02444616
2423	ZNF256	2.17012025	6.0952E-20	3.8333E-19
2424	SLC2A12	2.16985215	5.736E-10	1.975E-09
2425	SLC22A20	2.16855165	0.00058125	0.00114636
2426	ZSWIM4	2.16618522	2.6593E-27	2.3611E-26
2427	MYBL2	2.16509271	1.1997E-15	6.0456E-15
2428	RPL23AP64	2.16459789	3.6236E-06	9.0497E-06
2429	PRICKLE1	2.16458309	3.2088E-12	1.2955E-11
2430	GAB1	2.16335352	2.699E-32	2.8714E-31
2431	LOC728175	2.162868	0.00196548	0.00362408
2432	SHCBP1	2.16232233	4.6109E-10	1.5996E-09
2433	METTL9	2.16140071	8.565E-133	7.955E-131
2434	AGRN	2.16058799	4.4507E-23	3.2521E-22
2435	ZNF675	2.15757814	4.2171E-13	1.8049E-12
2436	OVGP1	2.15727685	1.4216E-14	6.7019E-14
2437	SAMD15	2.15720662	0.00089623	0.00172943
2438	BANK1	2.15602612	0.00032974	0.00067046
2439	DNAH10	2.15421452	3.4233E-11	1.2925E-10
2440	TTTY15	2.15348736	4.1836E-27	3.6857E-26
2441	PNISR	2.15230914	1.0423E-32	1.1282E-31
2442	NOVA1	2.15208341	0.00111591	0.0021224
2443	PKM	2.14923154	4.0073E-49	7.2221E-48
2444	BRIP1	2.14911856	3.6187E-13	1.5581E-12
2445	CDR2L	2.1483435	4.5858E-37	5.8021E-36
2446	ZNF852	2.14732308	3.2034E-23	2.3586E-22
2447	PLEKHA8	2.14673007	1.9643E-32	2.1002E-31
2448	CKAP2L	2.14531787	0.00011186	0.00024038
2449	MESP2	2.14513973	0.03071691	0.04682058
2450	MMP14	2.14503194	9.3147E-49	1.6648E-47
2451	KCTD7	2.14342292	7.1947E-18	4.0859E-17
2452	GNG4	2.14325445	0.03127452	0.0476029
2453	TEAD1	2.14321893	2.5641E-26	2.1727E-25
2454	SP6	2.14237706	0.000566	0.00111709
2455	C15orf39	2.14107542	4.9609E-97	2.6772E-95
2456	EVC2	2.14069592	1.1704E-17	6.5753E-17
2457	ANKRD6	2.13634852	6.899E-51	1.3197E-49
2458	FAM60A	2.13615043	7.1546E-46	1.1684E-44
2459	GS1-259H13.2	2.13454765	0.01167444	0.01912577
2460	ZNF284	2.13393992	6.2116E-14	2.8151E-13
2461	RAB6B	2.13385161	8.2532E-18	4.6787E-17
2462	SYNDIG1	2.12987957	0.01943107	0.03071578
2463	DDX3Y	2.12930403	4.2655E-41	6.0999E-40
2464	S100A16	2.12914523	5.1565E-18	2.9457E-17
2465	SMARCD3	2.12760351	1.0009E-30	1.003E-29
2466	MID1	2.12729131	5.5396E-12	2.2085E-11

2467	ANKRD36	2.12665133	6.9223E-09	2.1856E-08
2468	GPR133	2.12588735	1.4202E-18	8.3678E-18
2469	EBLN2	2.12537061	1.6807E-07	4.7273E-07
2470	DRAM1	2.12494153	9.9951E-41	1.4085E-39
2471	SPATC1L	2.12363694	9.5701E-16	4.8516E-15
2472	DNAJC18	2.12337325	7.4209E-22	5.1425E-21
2473	EFNB3	2.12228359	6.2191E-06	1.5186E-05
2474	DGKA	2.12206877	1.6088E-24	1.2609E-23
2475	TULP4	2.12013162	2.1224E-41	3.0693E-40
2476	USP2	2.11833066	8.0116E-12	3.1556E-11
2477	MAP1A	2.1166856	1.384E-08	4.262E-08
2478	PRRG1	2.1156336	1.5254E-21	1.0399E-20
2479	SASS6	2.11551719	2.9786E-23	2.1973E-22
2480	ZNF519	2.11418006	1.4491E-06	3.7559E-06
2481	KIAA1524	2.11358102	1.3987E-12	5.7915E-12
2482	ZMIZ1-AS1	2.11242573	0.00664286	0.01132147
2483	TNFAIP8	2.10931805	1.3066E-12	5.4322E-12
2484	ZNF112	2.10826815	5.9629E-14	2.7073E-13
2485	SCMH1	2.1079336	7.9328E-19	4.7289E-18
2486	CHN1	2.10489118	1.0949E-06	2.8725E-06
2487	GLRB	2.10486561	4.1528E-06	1.0303E-05
2488	ZNF135	2.10298262	1.3738E-12	5.693E-12
2489	SPIB	2.10273633	0.02738204	0.04209831
2490	CAPG	2.10183252	2.6167E-23	1.9377E-22
2491	NR2C2	2.10157279	1.5584E-32	1.6727E-31
2492	TSC22D1-AS1	2.1007839	6.5931E-08	1.9184E-07
2493	IQCJ-SCHIP1	2.10043468	6.899E-22	4.786E-21
2494	TRPS1	2.09995449	4.1843E-17	2.2766E-16
2495	SNORD29	2.09958327	0.00175512	0.00325766
2496	SCHIP1	2.09952979	5.6935E-22	3.9639E-21
2497	HES2	2.09765983	0.00346398	0.0061721
2498	ARHGAP39	2.09687657	2.0876E-20	1.3462E-19
2499	POLQ	2.09558261	1.6948E-12	6.9784E-12
2500	ERMP1	2.09481082	5.2826E-31	5.3547E-30
2501	CCDC88C	2.09470971	9.6972E-54	2.0038E-52
2502	SLC35G1	2.092066	6.5169E-08	1.8979E-07
2503	CAMK2D	2.09166586	2.1458E-29	2.0604E-28
2504	SYNE1	2.0909683	4.6621E-39	6.2506E-38
2505	COLGALT2	2.09042165	3.9684E-05	8.963E-05
2506	LY75	2.08932536	8.8769E-09	2.7733E-08
2507	C19orf40	2.08900315	1.4826E-07	4.1939E-07
2508	CHMP1B2P	2.08700227	0.03290552	0.04986936
2509	TRIM3	2.08484633	1.5287E-13	6.7664E-13
2510	LOC101927720	2.08400065	2.7703E-05	6.3542E-05
2511	RCBTB1	2.08173615	8.118E-14	3.6508E-13

2512	SSR4P1	2.0814745	8.6041E-09	2.6907E-08
2513	POMK	2.08117217	2.0279E-12	8.3006E-12
2514	ZNF821	2.08080904	1.2725E-19	7.9025E-19
2515	ATP1A1-AS1	2.07775982	1.5804E-27	1.4142E-26
2516	NRP2	2.07770722	9.6188E-07	2.5353E-06
2517	ZNF100	2.07743457	1.2462E-24	9.8431E-24
2518	PLK4	2.07557491	2.6474E-10	9.3507E-10
2519	RIMKLB	2.07501402	1.316E-11	5.1221E-11
2520	CCDC39	2.07500052	9.1322E-10	3.0942E-09
2521	NRSN2	2.07492792	4.371E-55	9.3519E-54
2522	PRDM16	2.07476438	0.00210796	0.0038698
2523	NLGN4Y	2.07442045	1.8567E-30	1.8453E-29
2524	KRBA2	2.07433154	5.6237E-13	2.3889E-12
2525	MAP4K4	2.07415833	2.559E-37	3.2634E-36
2526	SLC13A4	2.07238893	0.02117319	0.03322499
2527	GRB10	2.0721081	7.428E-52	1.4674E-50
2528	LINC01123	2.07173617	0.00022458	0.00046494
2529	TPM1	2.07040355	1.3205E-19	8.1981E-19
2530	DZIP1L	2.07022183	4.9966E-17	2.7056E-16
2531	ACVR2B	2.06923231	8.4864E-21	5.5825E-20
2532	STK31	2.06864948	0.02184956	0.03419192
2533	CATSPERB	2.06765899	0.02374383	0.03691404
2534	TPBG	2.06758201	1.1988E-41	1.7479E-40
2535	DST	2.06631931	1.4147E-33	1.5735E-32
2536	SAPCD2	2.06533111	2.9814E-05	6.8199E-05
2537	DLEU2	2.06475405	1.92E-14	8.9688E-14
2538	FGF2	2.06426944	1.9611E-18	1.1488E-17
2539	LINC00571	2.06375695	0.00259074	0.00470337
2540	SENP7	2.06281739	7.7767E-38	1.005E-36
2541	C3orf20	2.0624825	0.01115065	0.01831581
2542	C4orf36	2.06212025	0.00478602	0.00833699
2543	ZNF185	2.06046985	1.1734E-21	8.0588E-21
2544	SOX6	2.05965682	0.00024955	0.00051395
2545	PRRC2C	2.05901243	3.747E-31	3.8181E-30
2546	LRRC37BP1	2.05813712	1.0902E-14	5.1698E-14
2547	SUSD1	2.05728614	1.9901E-40	2.78E-39
2548	SLC9A1	2.05639332	7.4174E-19	4.4298E-18
2549	MKL2	2.05621358	7.405E-28	6.7135E-27
2550	WDR66	2.05586787	8.9157E-10	3.023E-09
2551	ZNF708	2.05584269	2.781E-23	2.0554E-22
2552	SPAG1	2.05449323	2.5151E-24	1.9546E-23
2553	SAP30L-AS1	2.05408241	0.01216211	0.01987751
2554	NABP1	2.05346509	4.0941E-33	4.4967E-32
2555	ZNF184	2.0529074	1.688E-20	1.0933E-19
2556	PRKCE	2.05160083	1.0008E-21	6.8885E-21

2557	MTF2	2.05156819	3.9609E-47	6.6775E-46
2558	ZNF879	2.05131023	2.1728E-11	8.3226E-11
2559	NUDT4	2.05126557	1.0193E-30	1.0209E-29
2560	ZFP14	2.04923616	2.2766E-16	1.1884E-15
2561	GSG2	2.04896868	1.7844E-07	5.0086E-07
2562	ARSJ	2.04827799	7.8966E-15	3.7819E-14
2563	LOC101927204	2.04625254	3.4863E-13	1.5034E-12
2564	CLIP3	2.04512818	7.3741E-19	4.4053E-18
2565	MIA2	2.04432345	5.578E-17	3.0111E-16
2566	DZIP3	2.04264466	2.504E-32	2.6684E-31
2567	RALGAPB	2.04077627	7.4015E-33	8.0381E-32
2568	ACRC	2.04034227	4.1656E-09	1.3384E-08
2569	SEMA6A	2.03889329	4.3791E-23	3.2023E-22
2570	TFF2	2.03768834	6.4211E-06	1.5671E-05
2571	DDX25	2.03660512	0.01471023	0.0237052
2572	LOC100506127	2.03615942	0.00287066	0.0051773
2573	MAFG-AS1	2.03614197	3.4372E-05	7.8107E-05
2574	ATE1-AS1	2.0332464	3.8708E-05	8.7537E-05
2575	STMN1	2.03231416	2.6256E-12	1.0666E-11
2576	LGR6	2.03202123	1.1033E-06	2.8915E-06
2577	NHS	2.03111183	3.9149E-05	8.8494E-05
2578	PRSS30P	2.0309849	0.01221877	0.01995829
2579	LINC00341	2.02929049	1.0903E-12	4.5546E-12
2580	WARS	2.02856788	7.7616E-13	3.272E-12
2581	HSF2BP	2.02821682	2.1103E-06	5.3924E-06
2582	SPHK1	2.02802248	6.5229E-11	2.411E-10
2583	ZNF883	2.02722516	0.02447885	0.03796816
2584	SUPT3H	2.02413015	1.5995E-13	7.0747E-13
2585	JARID2	2.02372816	3.8739E-23	2.8393E-22
2586	C7orf31	2.02370414	7.4266E-16	3.7858E-15
2587	LOC440600	2.02364889	1.5469E-05	3.6415E-05
2588	RBM20	2.02361515	0.0001406	0.00029853
2589	PHIP	2.02317384	2.8376E-30	2.8014E-29
2590	MYO5C	2.02310935	1.0476E-30	1.0486E-29
2591	NLGN2	2.02275524	2.3123E-08	6.9878E-08
2592	ARHGAP44	2.02232391	9.0436E-07	2.3879E-06
2593	GCNT2	2.02170487	4.9141E-14	2.2429E-13
2594	DSTNP2	2.01955802	1.3945E-14	6.5799E-14
2595	DBP	2.01832765	2.061E-21	1.3952E-20
2596	FRMD6-AS1	2.01801004	0.00197954	0.00364791
2597	CCDC62	2.01778938	0.02830099	0.04339141
2598	CMTM3	2.01593622	3.1165E-62	8.328E-61
2599	NEU3	2.01565284	4.2708E-20	2.7089E-19
2600	COL4A4	2.01559448	0.00095667	0.00183799
2601	PLXNA3	2.01522732	2.5705E-21	1.734E-20

2602	ARHGAP24	2.01437472	1.3412E-14	6.337E-14
2603	ADAMTS9	2.0133611	2.096E-09	6.9021E-09
2604	LOC54944	2.01283043	2.0253E-12	8.2917E-12
2605	FGFR2	2.01113393	0.01564599	0.02510844
2606	CBX2	2.01112144	2.2143E-05	5.1296E-05
2607	SNAI3	2.00919659	1.1302E-07	3.2259E-07
2608	CACNB1	2.00892185	1.7943E-06	4.6154E-06
2609	BMP2K	2.00875538	2.6029E-31	2.6763E-30
2610	LOC100506639	2.00851288	6.3372E-13	2.6844E-12
2611	VSTM2L	2.00843997	0.00775864	0.0130983
2612	CEP350	2.00660019	1.4843E-14	6.9792E-14
2613	KAL1	2.00653994	5.145E-16	2.6415E-15
2614	TMCC2	2.00572931	5.5103E-10	1.901E-09
2615	C8orf31	2.00513692	1.3701E-07	3.8882E-07
2616	APOBEC3D	2.00419957	6.1768E-10	2.1204E-09
2617	ATP9A	2.00364404	4.4639E-36	5.4723E-35
2618	ZNF841	2.00067677	5.4218E-26	4.5326E-25

Differentially expressed genes were genes with at least a 4-fold change in gene expression ($|Log2FC| \geq 2$) and adjusted p-value < 0.05.
DEG, differentially expressed genes.

Supplementary Table S4. Downregulated DEG in iHLC Compared to Adult Liver Stage

	Gene	Log2FC	P-value	Adjusted p-value
1	CRP	-21.504866	1.196E-138	1.184E-136
2	APCS	-17.509055	2.3837E-92	1.16E-90
3	F9	-16.336965	1.4394E-80	5.7337E-79
4	HSD11B1	-16.130659	2.3945E-78	9.179E-77
5	AZGP1	-16.123863	4.6249E-97	2.5029E-95
6	CFHR2	-15.584448	4.9407E-73	1.7015E-71
7	SDS	-15.455619	1.5068E-77	5.6748E-76
8	CFHR5	-15.326264	1.4661E-70	4.7846E-69
9	C1QB	-15.266667	5.4142E-70	1.7318E-68
10	CFHR1	-15.13436	4.797E-69	1.5094E-67
11	C1QC	-15.040473	7.5221E-68	2.3178E-66
12	ACSM5	-14.707254	4.2418E-65	1.2177E-63
13	HAO1	-14.692133	5.8344E-65	1.6625E-63
14	MT1G	-14.654494	0.00325524	0.00582272
15	C1QA	-14.625818	5.822E-64	1.6092E-62
16	FCN2	-14.546141	3.2148E-63	8.7726E-62
17	ADH1A	-14.435881	3.4015E-62	9.0645E-61
18	CYP4F11	-14.42827	4.0022E-62	1.0636E-60
19	MT1E	-14.268455	1.2093E-60	3.0701E-59
20	CYP2A6	-14.217822	3.3293E-15	1.6313E-14
21	MT1M	-14.217808	3.5531E-60	8.9617E-59
22	GSTT1	-14.137835	1.9446E-59	4.7557E-58
23	AKR1C4	-14.127207	1.4785E-64	4.1519E-63
24	CD5L	-14.010412	2.9042E-58	6.8426E-57
25	SLCO1B1	-13.893623	1.1901E-71	3.9988E-70
26	CYP2B6	-13.805591	1.6623E-43	2.5368E-42
27	CES1	-13.715108	0	0
28	ADH1B	-13.70262	8.3308E-13	3.5035E-12
29	ARG1	-13.694783	1.1268E-38	1.4993E-37
30	FCGR3A	-13.64236	6.9789E-55	1.4849E-53
31	CLEC4G	-13.597027	1.8173E-54	3.8289E-53
32	AQP9	-13.403139	1.5814E-66	4.7443E-65
33	IGFBP1	-13.397983	1.0893E-57	2.5174E-56
34	C3P1	-13.328948	9.8835E-66	2.9064E-64
35	SLCO1B3	-13.281415	1.4172E-51	2.7768E-50
36	SLC25A47	-13.187291	3.9599E-10	1.3792E-09
37	SPP2	-13.158497	1.896E-50	3.5667E-49
38	MT1H	-13.134174	3.1681E-50	5.9024E-49
39	SLC6A1	-13.118665	4.3952E-50	8.1649E-49
40	TAT	-13.038335	1.1863E-28	1.1093E-27
41	VSIG4	-12.928793	2.4263E-48	4.2653E-47
42	SIGLEC1	-12.858593	1.0709E-47	1.8473E-46

43	DTX1	-12.830125	2.2316E-48	3.9339E-47
44	MARCO	-12.819299	5.7934E-07	1.5547E-06
45	MT1A	-12.813248	3.1361E-48	5.5031E-47
46	ADRA1A	-12.811436	3.2468E-48	5.6923E-47
47	TRPM8	-12.802413	3.5169E-47	5.9551E-46
48	CYP1A2	-12.770756	7.3726E-48	1.2821E-46
49	UGT1A4	-12.727447	0	0
50	ADH4	-12.67938	0.01097269	0.01805113
51	UROC1	-12.675111	5.0436E-47	8.4955E-46
52	UGT1A1	-12.648665	0	0
53	CLEC4M	-12.632369	1.2932E-45	2.1049E-44
54	HFE2	-12.568013	6.457E-77	2.3944E-75
55	C9	-12.531376	2.8579E-20	1.8319E-19
56	UGT1A9	-12.486835	0	0
57	FAM83A-AS1	-12.461245	3.7091E-45	5.9468E-44
58	UGT1A3	-12.458333	0	0
59	MS4A6A	-12.453014	4.6653E-49	8.3845E-48
60	UGT1A5	-12.443435	0	0
61	UGT1A8	-12.443166	0	0
62	UGT1A7	-12.443155	0	0
63	RORC	-12.442024	7.4021E-44	1.1404E-42
64	CFHR4	-12.433346	5.5305E-65	1.5783E-63
65	UGT1A10	-12.412602	0	0
66	GC	-12.408079	3.5609E-31	3.6362E-30
67	UGT1A6	-12.325673	0	0
68	FCGR2B	-12.224119	7.7598E-42	1.1366E-40
69	IL33	-12.208832	1.0764E-41	1.573E-40
70	PON1	-12.203643	8.168E-136	7.773E-134
71	LOC100132529	-12.183863	3.5298E-72	1.1964E-70
72	CYP4F2	-12.1251	6.2506E-93	3.1044E-91
73	C4BPA	-12.092774	0	0
74	HBB	-12.080854	1.6741E-40	2.3438E-39
75	RAMP3	-12.070848	2.0755E-40	2.8952E-39
76	MT2A	-12.039568	1.4972E-40	2.1022E-39
77	FCN3	-12.018541	6.302E-132	5.77E-130
78	GDF2	-11.925135	1.761E-40	2.4636E-39
79	VNN1	-11.906504	1.573E-99	8.8088E-98
80	COL5A3	-11.834369	1.0941E-39	1.4949E-38
81	G6PC	-11.824649	2.9018E-19	1.7741E-18
82	MT1X	-11.82156	2.692E-195	5.201E-193
83	PTPRC	-11.817295	4.9023E-38	6.3952E-37
84	LILRB5	-11.804004	6.5349E-38	8.4962E-37
85	ACSM2B	-11.762114	7.117E-167	1.042E-164
86	OAS2	-11.699317	1.655E-38	2.1901E-37
87	TMEM176B	-11.696692	2.02E-157	2.534E-155

88	LINC01485	-11.672971	1.1176E-36	1.3931E-35
89	S100A9	-11.547911	1.2717E-40	1.7895E-39
90	LOC101927136	-11.522669	2.9464E-35	3.4902E-34
91	TYROBP	-11.508421	7.7171E-37	9.694E-36
92	IGLL5	-11.42352	2.5631E-34	2.9458E-33
93	CCL21	-11.384934	5.9561E-34	6.7058E-33
94	ADIRF	-11.366325	1.3555E-35	1.6327E-34
95	CD14	-11.362161	1.0376E-34	1.2084E-33
96	FOLR2	-11.353308	1.7479E-35	2.0962E-34
97	CYP2A7	-11.340145	1.8441E-10	6.594E-10
98	ORM1	-11.313835	2.519E-42	3.735E-41
99	PCK1	-11.313313	1.337E-47	2.2981E-46
100	CFHR3	-11.275852	6.081E-78	2.3127E-76
101	CYP2E1	-11.274782	6.447E-255	2.076E-252
102	C8G	-11.231924	1.301E-270	5.128E-268
103	C7	-11.192064	6.0915E-14	2.7633E-13
104	KLKB1	-11.155217	4.0556E-67	1.2281E-65
105	RDH16	-11.117508	0	0
106	CD84	-11.059175	7.5479E-31	7.5793E-30
107	SRGN	-11.049454	0.00039144	0.00078737
108	ABCA6	-10.913261	7.5861E-24	5.7544E-23
109	PEG3	-10.898926	1.6814E-31	1.7399E-30
110	CYP3A4	-10.886532	4.1662E-13	1.7847E-12
111	CR1	-10.883778	3.584E-29	3.411E-28
112	CD209	-10.866834	5.2052E-29	4.9296E-28
113	GBP7	-10.854571	0.00772337	0.01304103
114	LILRB2	-10.848455	7.8025E-29	7.3427E-28
115	MRC1	-10.817508	2.1079E-78	8.0965E-77
116	HEPACAM	-10.80584	6.1653E-34	6.9373E-33
117	S100A8	-10.804639	2.0482E-28	1.9025E-27
118	LINC01093	-10.7656	7.2769E-34	8.1691E-33
119	LILRB1	-10.711445	1.5949E-27	1.4266E-26
120	TMEM176A	-10.677323	0	0
121	HLA-DRA	-10.665991	4.3384E-27	3.8186E-26
122	IFI44	-10.658977	5.0626E-27	4.4418E-26
123	CYP4A11	-10.62449	0	0
124	TTPA	-10.619048	1.271E-83	5.3733E-82
125	F11-AS1	-10.586708	1.3569E-58	3.2404E-57
126	CYP4A22	-10.582731	3.5936E-06	8.9782E-06
127	ORM2	-10.562689	1.3824E-34	1.6012E-33
128	C3	-10.549066	1.9408E-19	1.1976E-18
129	FAM180A	-10.509472	1.3548E-25	1.111E-24
130	OTC	-10.487551	2.4928E-64	6.9596E-63
131	SAA2-SAA4	-10.481182	5.9815E-31	6.0359E-30
132	MT1L	-10.469943	3.2256E-25	2.6151E-24

133	DOK2	-10.447325	5.2966E-25	4.2585E-24
134	SYT12	-10.446857	3.2543E-49	5.876E-48
135	F11	-10.40872	6.7511E-29	6.375E-28
136	FPR1	-10.389177	1.8927E-24	1.4775E-23
137	FAM169B	-10.330354	6.8466E-24	5.2098E-23
138	SPI1	-10.330294	4.8588E-30	4.7606E-29
139	ITGAD	-10.305418	1.1798E-23	8.8656E-23
140	GIMAP1-GIMAP5	-10.265711	2.9492E-05	6.7478E-05
141	HEPN1	-10.227833	1.1842E-25	9.7443E-25
142	TNFSF14	-10.225638	5.8741E-11	2.177E-10
143	SAA2	-10.221896	2.3157E-21	1.5649E-20
144	C8A	-10.208755	1.1116E-37	1.4308E-36
145	CP	-10.200493	0	0
146	HK3	-10.15304	5.5687E-08	1.6326E-07
147	CXCR2P1	-10.127837	5.5785E-22	3.8867E-21
148	LILRA2	-10.111489	7.9805E-22	5.5125E-21
149	SASH3	-10.10531	9.1192E-22	6.2922E-21
150	HCK	-10.102453	2.0135E-27	1.7926E-26
151	PLEK	-10.100077	1.5009E-24	1.1797E-23
152	SLC22A1	-10.08532	0	0
153	FPR3	-10.085158	1.9587E-24	1.5283E-23
154	ABCB1	-10.076189	1.8637E-84	8.0733E-83
155	LOC100507389	-10.052766	2.8286E-21	1.9048E-20
156	IGFALS	-10.043253	3.354E-27	2.9643E-26
157	PIGR	-10.041738	8.4538E-16	4.2958E-15
158	RET	-10.037902	5.0767E-24	3.8875E-23
159	NCKAP1L	-10.03028	2.317E-32	2.4731E-31
160	C19orf80	-10.024634	8.2703E-90	3.9067E-88
161	GIMAP7	-10.023888	6.4167E-24	4.8981E-23
162	LINC01272	-10.011512	6.8585E-21	4.5332E-20
163	HAMP	-9.9708628	3.0402E-14	1.4049E-13
164	FGD2	-9.9417298	5.1373E-26	4.3003E-25
165	FCGR2A	-9.9401364	3.4878E-23	2.5631E-22
166	KRT13	-9.9307224	7.3035E-26	6.0638E-25
167	VNN3	-9.8946536	8.0235E-23	5.7928E-22
168	CHL1	-9.8945658	8.3083E-20	5.1998E-19
169	DAO	-9.8834935	2.9444E-41	4.2453E-40
170	F13A1	-9.8782026	1.0549E-35	1.2737E-34
171	ITGB2	-9.8660822	1.9882E-85	8.8101E-84
172	LCN2	-9.8582848	1.7914E-19	1.1072E-18
173	LINC01018	-9.8536701	8.66E-136	8.202E-134
174	P2RY13	-9.8310866	3.1807E-19	1.9398E-18
175	BAAT	-9.8296775	1.9225E-35	2.2999E-34
176	HLA-DRB5	-9.8279528	0.01587044	0.02543272
177	CDH19	-9.8260859	3.5342E-19	2.1499E-18

178	CYP2C8	-9.8043172	9.892E-248	2.896E-245
179	UGT2B15	-9.8038835	2.4346E-35	2.8982E-34
180	A1BG	-9.8031132	1.296E-253	4.039E-251
181	AKR1C6P	-9.7541928	1.5973E-18	9.3885E-18
182	SIGLEC10	-9.7339461	2.8248E-34	3.2369E-33
183	OLFM4	-9.7222498	3.1093E-18	1.8023E-17
184	ABCB4	-9.7217564	2.125E-228	5.401E-226
185	TPSB2	-9.711443	3.8929E-18	2.2417E-17
186	HLA-H	-9.7085943	3.1068E-21	2.0885E-20
187	CD53	-9.6810874	3.1819E-39	4.293E-38
188	CNDP1	-9.6695282	1.3937E-76	5.1386E-75
189	RPL7	-9.6229585	1.135E-231	3.004E-229
190	FGR	-9.6145027	6.8178E-24	5.1899E-23
191	MNDA	-9.6132623	2.9548E-17	1.6246E-16
192	PRF1	-9.5986747	3.9836E-17	2.1704E-16
193	SYCE1	-9.5927981	4.4922E-17	2.4393E-16
194	FGF21	-9.5839381	5.3833E-17	2.9109E-16
195	ADORA3	-9.569988	4.5883E-28	4.2032E-27
196	ASPG	-9.5460336	8.069E-191	1.499E-188
197	LECT2	-9.5360103	0.0010119	0.00193564
198	CRYAA	-9.5203461	1.9584E-16	1.0262E-15
199	GPIHBP1	-9.5203461	1.9584E-16	1.0262E-15
200	NFIX	-9.4950469	8.8363E-10	2.9977E-09
201	NCF4	-9.4922102	3.4528E-16	1.7889E-15
202	TFR2	-9.4858281	3.137E-274	1.289E-271
203	VNN2	-9.479045	2.3882E-19	1.4676E-18
204	EVI2B	-9.4676904	1.1494E-22	8.2518E-22
205	IGJ	-9.4675859	2.7709E-19	1.6968E-18
206	CCL5	-9.4407945	9.662E-16	4.8969E-15
207	APOF	-9.4224807	1.2597E-18	7.4472E-18
208	AGXT	-9.4189163	4.092E-162	5.568E-160
209	C14orf180	-9.4016763	1.0068E-18	5.9687E-18
210	ATOH8	-9.3887932	1.2162E-93	6.0715E-92
211	IFI44L	-9.3630954	7.2903E-21	4.8071E-20
212	LOC100233156	-9.3600629	4.7654E-15	2.3168E-14
213	ITIH3	-9.3329251	3.327E-162	4.558E-160
214	SLC1A2	-9.3302862	3.419E-119	2.632E-117
215	CSNK2B	-9.321495	1.0121E-14	4.8066E-14
216	LILRA3	-9.3143706	1.1624E-14	5.5109E-14
217	SAA4	-9.2902185	4.5538E-23	3.3262E-22
218	PARVG	-9.2863996	8.4618E-18	4.7942E-17
219	LOC101929337	-9.2811724	8.509E-18	4.8181E-17
220	CYP2C9	-9.2788907	8.698E-120	6.859E-118
221	NR1H4	-9.273005	4.225E-10	1.4694E-09
222	RXFP1	-9.2448734	4.4497E-14	2.0376E-13

223	ADH6	-9.2379013	3.969E-130	3.566E-128
224	HGFAC	-9.2152454	8.155E-249	2.462E-246
225	VAV1	-9.2114374	3.2806E-17	1.7965E-16
226	EMR1	-9.203053	9.8605E-14	4.4098E-13
227	LILRB4	-9.1976316	3.8225E-17	2.085E-16
228	ACSM2A	-9.1670818	1.458E-178	2.407E-176
229	LINC01127	-9.1599841	2.2186E-13	9.7062E-13
230	LAIR1	-9.1557092	4.4729E-19	2.7022E-18
231	MS4A4A	-9.155201	8.1393E-39	1.0875E-37
232	NAPSB	-9.15334	4.599E-20	2.9113E-19
233	TLR8	-9.139974	3.2238E-13	1.3949E-12
234	SYT9	-9.1337721	0.00169595	0.0031552
235	CYP8B1	-9.116459	5.5641E-84	2.3942E-82
236	AZGP1P1	-9.1148428	1.8028E-16	9.4647E-16
237	CYP2D6	-9.1026914	9.584E-210	2.08E-207
238	LOC101929829	-9.1026914	9.584E-210	2.08E-207
239	UBXN10	-9.0911429	2.7999E-16	1.4569E-15
240	MS4A7	-9.0888618	8.1239E-51	1.5509E-49
241	CES1P1	-9.0866105	8.6485E-13	3.63E-12
242	C10orf128	-9.0821541	2.9395E-16	1.5287E-15
243	DHX58	-9.0816577	1.4996E-05	3.5349E-05
244	PLGLA	-9.0761303	3.6437E-16	1.8858E-15
245	STAB2	-9.067695	2.273E-17	1.2554E-16
246	CXCL13	-9.0612992	1.374E-12	5.693E-12
247	CD180	-9.031197	2.3725E-12	9.67E-12
248	TPSAB1	-9.026845	2.5663E-12	1.0436E-11
249	AOX1	-9.0260628	1.632E-117	1.222E-115
250	HLA-B	-8.9851836	2.162E-102	1.258E-100
251	CD79B	-8.9825868	5.6716E-12	2.2593E-11
252	SLPI	-8.9796624	2.644E-76	9.6565E-75
253	HLA-DRB1	-8.9774175	8.25E-46	1.3451E-44
254	NGFR	-8.9627759	4.8983E-06	1.2083E-05
255	UGT2B4	-8.9594462	4.5189E-07	1.2255E-06
256	MT1DP	-8.9533404	2.8305E-15	1.394E-14
257	SLC27A5	-8.9477831	0	0
258	HRG	-8.9403769	2.948E-122	2.455E-120
259	FCN1	-8.9229434	1.6226E-11	6.271E-11
260	LHX2	-8.9133554	5.9707E-15	2.881E-14
261	RPS28	-8.900157	2E-119	1.552E-117
262	PTPRT	-8.8945614	2.6563E-11	1.0122E-10
263	CCL23	-8.8936696	5.4834E-21	3.6418E-20
264	CES5A	-8.8801583	3.4049E-11	1.2858E-10
265	FAIM3	-8.8791666	1.0703E-14	5.0795E-14
266	OASL	-8.859621	1.8091E-25	1.4747E-24
267	LRRC55	-8.8535377	1.2137E-20	7.9221E-20

268	PPP1R11	-8.8459809	6.1064E-11	2.2601E-10
269	FCGR3B	-8.8459809	6.1064E-11	2.2601E-10
270	ABLIM3	-8.8248394	3.027E-283	1.299E-280
271	HLA-F	-8.8160275	1.0128E-10	3.6946E-10
272	GIMAP6	-8.813334	1.2279E-51	2.4133E-50
273	PEG3-AS1	-8.8008143	1.3067E-10	4.7258E-10
274	CPA3	-8.7957073	1.4229E-10	5.1288E-10
275	BCL2L10	-8.7957073	1.4229E-10	5.1288E-10
276	LOC100129083	-8.7930736	2.8332E-17	1.5598E-16
277	C4A	-8.7905823	1.5496E-10	5.567E-10
278	C4B	-8.7905823	1.5496E-10	5.567E-10
279	C4B_2	-8.7905823	1.5496E-10	5.567E-10
280	HCLS1	-8.7795779	2.3683E-90	1.127E-88
281	CFP	-8.7778061	1.7915E-64	5.0234E-63
282	CYP2J2	-8.7712389	3.3456E-06	8.3857E-06
283	ALDH4A1	-8.7644594	0	0
284	CD1D	-8.7600378	3.6795E-31	3.7533E-30
285	SPRNP1	-8.7489127	3.0818E-10	1.0839E-09
286	LY86	-8.7383046	3.6645E-10	1.2805E-09
287	CLEC3B	-8.7373405	3.8838E-46	6.4078E-45
288	LOC389834	-8.7336833	1.1446E-13	5.0932E-13
289	RCSD1	-8.7314337	8.4236E-27	7.298E-26
290	BHMT2	-8.7088647	4.7488E-22	3.3146E-21
291	NR1I2	-8.7015988	3.269E-115	2.347E-113
292	LCP2	-8.7013193	6.43E-27	5.6212E-26
293	SIGLEC14	-8.671694	4.3914E-16	2.2637E-15
294	SERPINC1	-8.6701448	7.757E-121	6.244E-119
295	IL10RA	-8.6668592	3.3909E-44	5.2791E-43
296	CHST4	-8.6665443	5.4088E-20	3.4139E-19
297	GIMAP4	-8.642925	2.7541E-52	5.4912E-51
298	C5AR2	-8.6103507	2.7859E-09	9.0794E-09
299	IFITM2	-8.6030085	1.8651E-17	1.0349E-16
300	LRG1	-8.5964698	0	0
301	DOCK2	-8.5724519	9.8331E-27	8.5E-26
302	MPEG1	-8.53435	4.997E-104	2.998E-102
303	SAA3P	-8.5133163	2.1568E-26	1.8357E-25
304	LYZ	-8.5111313	7.2779E-96	3.8418E-94
305	ECHDC3	-8.5107538	1.141E-289	5.378E-287
306	GNA15	-8.495357	1.5717E-08	4.8145E-08
307	CNTNAP3B	-8.4827016	1.8886E-08	5.7478E-08
308	CNTNAP3P2	-8.4827016	1.8886E-08	5.7478E-08
309	TPRG1-AS1	-8.4810244	3.1493E-19	1.923E-18
310	OAS1	-8.4756057	3.709E-74	1.2935E-72
311	APOA5	-8.4755012	4.9113E-12	1.9637E-11
312	LYVE1	-8.4621747	3.6881E-10	1.2883E-09

313	HLA-DQA1	-8.455164	2.3352E-20	1.5024E-19
314	REG1A	-8.4445485	5.5743E-17	3.0099E-16
315	MPPED1	-8.4390995	2.6693E-06	6.7537E-06
316	LILRA5	-8.427927	1.3377E-11	5.1994E-11
317	DPEP2	-8.4243368	4.3361E-08	1.2823E-07
318	ITIH2	-8.423369	1.2477E-16	6.6135E-16
319	LILRA1	-8.4110398	5.2207E-08	1.5345E-07
320	GPR65	-8.4085218	1.6282E-14	7.6407E-14
321	CLEC1B	-8.407459	0.00604553	0.01036745
322	TNFRSF14	-8.4056121	1.7976E-70	5.8565E-69
323	THRSP	-8.4043308	1.8472E-87	8.4171E-86
324	C5orf27	-8.4042172	7.9344E-66	2.3403E-64
325	HLF	-8.3973391	6.32E-126	5.451E-124
326	AKR7A3	-8.392558	1.604E-119	1.255E-117
327	MOGAT2	-8.3862066	1.3092E-33	1.4587E-32
328	CXCR1	-8.3772511	8.3156E-08	2.3982E-07
329	SELE	-8.36725	9.0417E-28	8.1591E-27
330	TSHZ2	-8.3481822	1.1842E-49	2.1666E-48
331	GBP5	-8.3373847	4.938E-14	2.2527E-13
332	SIGLEC8	-8.3356315	1.4571E-07	4.1247E-07
333	LRCOL1	-8.3286372	3.7386E-20	2.3854E-19
334	SKAP1	-8.3211293	1.7193E-19	1.0629E-18
335	LOC100288866	-8.3143626	1.9303E-07	5.4034E-07
336	CXCL9	-8.3096875	7.1233E-11	2.6274E-10
337	CD33	-8.3079163	7.7687E-14	3.5011E-13
338	HPR	-8.3042492	4.3727E-77	1.6341E-75
339	PDE2A	-8.2908739	3.365E-114	2.355E-112
340	FGL2	-8.2588604	8.3704E-67	2.5308E-65
341	PRKCB	-8.2311421	2.3274E-27	2.0683E-26
342	CD74	-8.2231719	7.301E-60	1.8154E-58
343	ATF6B	-8.2183922	6.6001E-07	1.7598E-06
344	EVI2A	-8.2141798	2.3406E-10	8.3051E-10
345	PAQR9	-8.1946053	4.2222E-52	8.3751E-51
346	PGLYRP2	-8.1941139	4.9522E-13	2.1102E-12
347	ATF5	-8.1814497	0	0
348	HP	-8.1805441	5.7968E-26	4.8399E-25
349	A1BG-AS1	-8.1780292	0	0
350	CNRIP1	-8.1773559	5.0059E-15	2.4294E-14
351	NR0B2	-8.1713514	1.0196E-96	5.4417E-95
352	PTPRO	-8.1696139	5.0748E-10	1.7549E-09
353	LOC115110	-8.1439333	4.7463E-25	3.8208E-24
354	SAMSN1	-8.1421947	8.2153E-13	3.4579E-12
355	CGREF1	-8.1320665	5.91E-103	3.46E-101
356	LOC403323	-8.131882	1.8626E-06	4.7814E-06
357	LINC00924	-8.1155799	2.2489E-06	5.7343E-06

358	P2RX1	-8.1073593	2.4711E-06	6.2693E-06
359	SLAMF6	-8.1073593	2.4711E-06	6.2693E-06
360	PIPOX	-8.1017377	5.334E-55	1.1387E-53
361	FYB	-8.0938252	0.0054149	0.00935072
362	SERPINA10	-8.0906258	1.289E-159	1.694E-157
363	B3GNT3	-8.0881495	1.4673E-09	4.8918E-09
364	DMGDH	-8.0824157	1.32E-176	2.125E-174
365	TPSD1	-8.0824125	3.2774E-06	8.2243E-06
366	CYP2C19	-8.0809477	3.207E-142	3.331E-140
367	GPR182	-8.0745006	1.6918E-21	1.1497E-20
368	GFRA2	-8.0583145	9.1542E-15	4.3615E-14
369	FABP4	-8.0484646	4.7728E-06	1.1787E-05
370	CHRDL2	-8.0262469	6.0032E-28	5.4631E-27
371	CD38	-8.0207464	1.8813E-17	1.0436E-16
372	GOLGA8M	-8.0048746	7.6249E-06	1.8467E-05
373	ADAMTSL2	-8.0005008	4.8263E-56	1.0742E-54
374	SLC22A10	-7.9945872	1.0302E-49	1.8902E-48
375	ABCG5	-7.9879377	7.6288E-47	1.2816E-45
376	MSC	-7.9826145	5.6393E-09	1.7935E-08
377	FABP1	-7.9788554	6.558E-108	4.181E-106
378	ZIM2	-7.9780741	1.009E-05	2.4147E-05
379	RUNDC3B	-7.9700585	4.8309E-28	4.4213E-27
380	MGLL	-7.9614221	1.955E-242	5.555E-240
381	RNASE6	-7.9431343	2.7907E-14	1.2914E-13
382	MMRN1	-7.9374365	3.4449E-46	5.7178E-45
383	INHBB	-7.9369042	1.0307E-55	2.2602E-54
384	ETNPPL	-7.9276769	4.837E-122	3.994E-120
385	LOC389602	-7.9229316	1.7622E-05	4.1287E-05
386	FPR2	-7.9135327	1.933E-05	4.5092E-05
387	CSTA	-7.9099689	1.2112E-08	3.7458E-08
388	BIN2	-7.8986272	2.3142E-16	1.2068E-15
389	CRNN	-7.8945493	2.325E-05	5.3737E-05
390	CLEC2B	-7.892746	8.9481E-13	3.7541E-12
391	AGTR1	-7.8858419	1.873E-110	1.244E-108
392	UGT2B17	-7.8846116	6.1187E-77	2.2777E-75
393	ALDOB	-7.8808613	3.656E-103	2.154E-101
394	ABCG8	-7.8802336	6.4315E-17	3.4573E-16
395	IL13RA2	-7.8655974	3.0637E-05	6.9983E-05
396	LOC101927269	-7.8655974	3.0637E-05	6.9983E-05
397	CD300C	-7.8360525	4.0317E-05	9.0975E-05
398	IL1RL2	-7.8239696	3.8035E-09	1.2255E-08
399	CCL16	-7.809359	2.6755E-06	6.7676E-06
400	ALDH1L1-AS1	-7.7998066	3.7944E-85	1.6699E-83
401	SELP	-7.7828764	1.6133E-13	7.1339E-13
402	THEM5	-7.7825888	5.9661E-08	1.7443E-07

403	SLFN11	-7.7811116	1.9178E-13	8.4323E-13
404	TBXA2R	-7.7747464	2.35E-28	2.1796E-27
405	LYSMD2	-7.7622563	2.7484E-18	1.5971E-17
406	CCR2	-7.7541739	8.3182E-05	0.0001812
407	CD2	-7.7541739	8.3182E-05	0.0001812
408	CD48	-7.7519988	1.6623E-10	5.9572E-10
409	NLRP6	-7.7452083	6.5965E-10	2.2585E-09
410	MEOX2	-7.7329569	9.9493E-05	0.00021482
411	WDFY4	-7.7317495	1.0312E-13	4.6064E-13
412	F12	-7.7310385	9.099E-151	1.04E-148
413	APOC4	-7.7189782	3.0004E-56	6.7249E-55
414	KHK	-7.6952436	3.607E-119	2.765E-117
415	WISP2	-7.6939754	5.7136E-15	2.7617E-14
416	NKG7	-7.6886688	1.3604E-12	5.6399E-12
417	MPO	-7.6785728	4.6984E-10	1.6285E-09
418	F8A3	-7.6548616	2.0033E-09	6.6069E-09
419	F8A2	-7.6548616	2.0033E-09	6.6069E-09
420	CTSG	-7.6448241	0.00020175	0.00042016
421	OGN	-7.6442526	6.314E-10	2.1648E-09
422	AMBP	-7.6394136	6.593E-22	4.5803E-21
423	FAM107A	-7.6266774	6.1199E-19	3.6696E-18
424	GRAP	-7.6162885	7.2508E-22	5.0264E-21
425	BST2	-7.5998075	1.0567E-76	3.9034E-75
426	OMD	-7.5986529	0.00028553	0.00058455
427	AKR1CL1	-7.5930393	1.1123E-09	3.7432E-09
428	KCNA3	-7.5761366	5.3653E-07	1.4431E-06
429	DEFA1	-7.5750011	0.00033911	0.00068761
430	DEFA3	-7.5750011	0.00033911	0.00068761
431	DEFA1B	-7.5750011	0.00033911	0.00068761
432	LOC200772	-7.5750011	0.00033911	0.00068761
433	CD86	-7.5640879	4.3029E-16	2.2198E-15
434	FAM99A	-7.5633694	1.084E-52	2.1838E-51
435	APOC2	-7.5605815	4.3123E-16	2.2241E-15
436	ELANE	-7.5502965	6.8568E-07	1.8252E-06
437	APOC4-APOC2	-7.5393205	3.0091E-17	1.6511E-16
438	HBA2	-7.5355067	1.2637E-12	5.2573E-12
439	KIF19	-7.5308686	3.3251E-07	9.1201E-07
440	HS1BP3-IT1	-7.5307366	5.3318E-43	8.0476E-42
441	GIMAP8	-7.5278102	3.6556E-25	2.955E-24
442	ADH1C	-7.5276931	1.0425E-49	1.909E-48
443	IFI27	-7.520598	1.939E-107	1.22E-105
444	LINC00659	-7.5081471	1.2221E-13	5.4226E-13
445	IL1RN	-7.5054058	1.9379E-87	8.8096E-86
446	REG3A	-7.5016262	0.00056399	0.00111345
447	REG1B	-7.5016262	0.00056399	0.00111345

448	ALPL	-7.500311	3.3956E-18	1.9624E-17
449	C22orf34	-7.4971847	1.1513E-06	3.0116E-06
450	SLC22A25	-7.4943858	5.5438E-18	3.1613E-17
451	MYH11	-7.4852454	2.637E-107	1.654E-105
452	CCL19	-7.4634901	0.00072208	0.00140858
453	SLC28A1	-7.4587451	3.576E-99	1.9851E-97
454	CUX2	-7.4496453	7.0152E-41	9.9584E-40
455	PIK3AP1	-7.4457369	4.8317E-20	3.0546E-19
456	LOC400867	-7.4355561	6.3545E-89	2.9512E-87
457	C6	-7.4349863	1.2403E-39	1.6923E-38
458	IKZF3	-7.4321141	7.6585E-09	2.4043E-08
459	PRG4	-7.4224229	0	0
460	CMKLR1	-7.4222013	6.9953E-24	5.3188E-23
461	TEKT4P2	-7.4110214	0.00100347	0.00192046
462	ROBO4	-7.4047897	2.3505E-09	7.71E-09
463	ASPN	-7.4003609	1.2874E-36	1.5995E-35
464	PTPRN2	-7.3940621	2.8253E-17	1.556E-16
465	SEC16B	-7.3756357	2.566E-161	3.443E-159
466	SPARCL1	-7.3751321	2.4758E-97	1.3436E-95
467	CLEC14A	-7.3716624	0.00042023	0.00084238
468	GGT5	-7.365854	4.021E-12	1.6151E-11
469	FGL1	-7.3538512	1.0448E-07	2.9905E-07
470	S100A12	-7.3426327	0.00150213	0.00281268
471	LOC283177	-7.3351265	5.9386E-06	1.4529E-05
472	GZMH	-7.3285571	0.00162657	0.00303216
473	ACP5	-7.3256535	1.7201E-61	4.4726E-60
474	CASP1	-7.3175815	1.0903E-19	6.7948E-19
475	INHBE	-7.3163734	3.4942E-51	6.7575E-50
476	PADI4	-7.3143429	0.00176066	0.00326699
477	C11orf96	-7.288244	5.7179E-44	8.8376E-43
478	BTNL9	-7.2857545	2.5338E-36	3.126E-35
479	S100B	-7.2854872	0.00206058	0.00379038
480	LINC00844	-7.2854857	1.4926E-10	5.3691E-10
481	CPN2	-7.2851265	1.519E-224	3.715E-222
482	INHBC	-7.2819063	1.604E-285	7.206E-283
483	STAB1	-7.2743801	0	0
484	CD300A	-7.2743274	1.6901E-30	1.6831E-29
485	PON3	-7.2718968	4.7428E-68	1.4661E-66
486	IL2RA	-7.2708401	0.00222791	0.004078
487	BTBD16	-7.2618155	4.6781E-15	2.276E-14
488	AOAH	-7.2592533	1.4192E-24	1.1173E-23
489	NPAS2	-7.2592473	4.6949E-65	1.3458E-63
490	LOC729083	-7.2399985	3.0351E-17	1.6645E-16
491	CLEC4E	-7.2259846	0.0028032	0.00506101
492	FCER1G	-7.2191378	1.4095E-66	4.235E-65

493	MUC5B	-7.2081295	1.7907E-12	7.3515E-12
494	CDA	-7.2003125	6.8966E-46	1.1282E-44
495	AKR1C2	-7.1981179	1.0181E-52	2.0533E-51
496	SAA1	-7.1970068	7.3937E-13	3.1196E-12
497	MFSD2A	-7.1957569	1.9461E-74	6.7991E-73
498	KCNB1	-7.177529	0.0006072	0.00119497
499	RNF165	-7.1751596	4.18E-13	1.7899E-12
500	MAFIP	-7.1639219	0.00379962	0.00672548
501	ESR1	-7.1617247	1.2703E-33	1.417E-32
502	FAM83A	-7.1582645	2.1124E-08	6.4009E-08
503	TMEM26	-7.1549048	9.5609E-10	3.235E-09
504	UPP2	-7.1541435	3.6461E-11	1.3729E-10
505	MATK	-7.1510323	7.1998E-06	1.7493E-05
506	EMR4P	-7.1479799	0.00409568	0.00721647
507	CD300LG	-7.1479799	0.00409568	0.00721647
508	IGF2-AS	-7.1477237	6.2469E-10	2.1433E-09
509	GJA4	-7.1366847	2.0345E-44	3.188E-43
510	SIRPB2	-7.1360481	1.9269E-08	5.8579E-08
511	PVALB	-7.1318598	0.00441303	0.00773402
512	CPB2	-7.1150606	4.6452E-14	2.1242E-13
513	OLFM1	-7.1105438	6.2767E-35	7.3361E-34
514	LOC100130872	-7.1069879	7.7311E-25	6.1695E-24
515	XAF1	-7.1023983	5.533E-62	1.4624E-60
516	LOC101929427	-7.0990689	0.00511717	0.00887227
517	MBL2	-7.0877255	2.3291E-11	8.9105E-11
518	CSF2RB	-7.0730436	9.4868E-23	6.839E-22
519	GZMA	-7.0700715	3.4824E-05	7.9079E-05
520	CHCHD2	-7.0672199	3.3335E-06	8.3576E-06
521	DEFA5	-7.0655154	0.00592399	0.01016894
522	OSGIN1	-7.0654487	2.048E-254	6.486E-252
523	4-Sep	-7.0638684	5.9913E-57	1.3602E-55
524	NAGS	-7.0595065	5.1093E-91	2.4494E-89
525	COX6A2	-7.0563854	2.9583E-07	8.1418E-07
526	SERPING1	-7.0515972	2.2611E-92	1.1031E-90
527	AADAC	-7.05146	5.2937E-56	1.1742E-54
528	FAM26F	-7.0475887	9.9918E-10	3.3743E-09
529	RASAL3	-7.0431505	6.116E-10	2.1003E-09
530	BLK	-7.0385761	3.9009E-07	1.0645E-06
531	GSTM5	-7.038575	3.4811E-07	9.5343E-07
532	PLIN1	-7.0365244	2.1218E-54	4.4606E-53
533	SLC14A1	-7.0311629	0.00684679	0.01165359
534	AGMO	-7.0293485	3.8161E-64	1.0623E-62
535	FMO3	-7.0148022	7.6077E-14	3.4301E-13
536	APOD	-7.0118898	1.4144E-16	7.4805E-16
537	ACOT12	-7.0088946	7.8917E-43	1.1874E-41

538	SLCO1B7	-6.9959724	0.00790035	0.01332018
539	CD4	-6.9923134	7.2E-104	4.307E-102
540	C16orf45	-6.9827538	1.5421E-26	1.3218E-25
541	P2RX7	-6.9496972	3.0648E-11	1.1624E-10
542	LDHC	-6.9434363	0.00010501	0.00022618
543	MOGAT1	-6.941523	0.00976218	0.0162032
544	GPR97	-6.9301394	0.0001133	0.00024318
545	VAMP5	-6.927621	4.2952E-55	9.1999E-54
546	CDO1	-6.9257705	3.955E-119	3.02E-117
547	FHL5	-6.9229067	0.01046704	0.0172737
548	SLC5A1	-6.9180313	2.4007E-16	1.2515E-15
549	LOC100132111	-6.9138835	2.3918E-08	7.2214E-08
550	REREP3	-6.9040471	0.0112182	0.0184158
551	GBP1P1	-6.9040471	0.0112182	0.0184158
552	PRRC2A	-6.9031719	0.00014608	0.0003097
553	RPL21P28	-6.8955943	7.738E-216	1.78E-213
554	RPL21	-6.8955943	7.738E-216	1.78E-213
555	ICAM2	-6.8945366	5.6614E-41	8.0603E-40
556	PODN	-6.8938059	3.4637E-21	2.3243E-20
557	INS	-6.8775912	1.5959E-06	4.1238E-06
558	CCL14	-6.8711861	3.8694E-08	1.1487E-07
559	LOC100132891	-6.8648627	1.8902E-08	5.7511E-08
560	RTP3	-6.8509765	8.8371E-48	1.5299E-46
561	LINC00238	-6.8476768	0.00017155	0.00036049
562	ZNRD1	-6.8459424	0.01377709	0.02230566
563	PRTN3	-6.8426344	4.8138E-07	1.3014E-06
564	CYP4F3	-6.8283205	1.7351E-63	4.7483E-62
565	DUSP2	-6.8156365	2.6662E-09	8.7086E-09
566	LINC01146	-6.8085606	2.3277E-13	1.0161E-12
567	LINC00864	-6.8058637	0.01576764	0.02529106
568	CYP21A2	-6.8058637	0.01576764	0.02529106
569	ABCA9	-6.8027309	4.3963E-14	2.0146E-13
570	ANKRD35	-6.8017825	8.5322E-13	3.5851E-12
571	CALN1	-6.8009556	3.3683E-06	8.4362E-06
572	ATP2B2	-6.7998563	4.6157E-27	4.0553E-26
573	ITGAM	-6.7975817	1.8661E-36	2.3096E-35
574	SLC6A13	-6.7934834	1.9368E-29	1.8617E-28
575	LOC101928505	-6.7933634	2.4191E-28	2.2426E-27
576	CD69	-6.7920727	3.2088E-06	8.0638E-06
577	CARD16	-6.7913744	1.7514E-07	4.9168E-07
578	C3AR1	-6.7893652	1.2549E-20	8.1769E-20
579	PTGFR	-6.7863917	3.9428E-07	1.0752E-06
580	F13B	-6.7799491	4.7306E-10	1.6391E-09
581	TGM3	-6.7795726	3.9941E-06	9.9288E-06
582	TMEM204	-6.7778947	2.309E-16	1.2044E-15

583	CLEC4GP1	-6.7751633	0.00029166	0.00059654
584	CA1	-6.7602134	0.00026967	0.00055356
585	MAL	-6.7602131	0.00029235	0.00059788
586	SRL	-6.7602127	0.00033323	0.00067704
587	MYL2	-6.7602125	0.00034587	0.00070038
588	HRC	-6.7600238	3.8897E-06	9.6816E-06
589	RPS26	-6.7554975	1.111E-211	2.467E-209
590	GZMK	-6.7483952	0.00016435	0.00034593
591	IL15RA	-6.7461251	2.463E-30	2.4378E-29
592	NLRP11	-6.7435774	0.01924744	0.03044793
593	C4BPB	-6.7399646	1.2151E-93	6.0715E-92
594	CXCR2	-6.7345076	6.6341E-07	1.7679E-06
595	CFH	-6.7263581	1.0189E-07	2.9197E-07
596	HSD17B13	-6.725924	2.385E-138	2.351E-136
597	MT1F	-6.7236894	9.2159E-05	0.00019983
598	CTRB2	-6.722203	0.02055401	0.03232952
599	GLS2	-6.7218367	6.486E-125	5.545E-123
600	BMP10	-6.714158	4.6551E-23	3.3977E-22
601	PFKFB1	-6.7055507	2.2681E-22	1.6022E-21
602	CST7	-6.7040238	6.4777E-09	2.051E-08
603	ABCB11	-6.7016472	3.5549E-06	8.8895E-06
604	C2orf40	-6.7015459	1.6703E-05	3.921E-05
605	CLCA4	-6.7005073	0.02194064	0.03431499
606	EVPLL	-6.7005073	0.02194064	0.03431499
607	ARHGEF6	-6.7003157	5.6928E-35	6.6697E-34
608	CYP2B7P	-6.6969199	2.9262E-50	5.4727E-49
609	LOC100652824	-6.6963554	7.2067E-06	1.7505E-05
610	MMP8	-6.6907039	7.9359E-06	1.9182E-05
611	FXYD1	-6.689822	4.7796E-11	1.7837E-10
612	SRD5A2	-6.6833063	2.954E-14	1.3657E-13
613	TBX21	-6.6784802	0.02340494	0.03642821
614	SAMD9L	-6.653259	1.6949E-19	1.0482E-18
615	SPESP1	-6.6509825	0.0006107	0.00120163
616	DPT	-6.6425828	1.1277E-09	3.7938E-09
617	SLAMF7	-6.6421842	4.7804E-07	1.2939E-06
618	ARHGAP25	-6.6419415	6.3408E-16	3.2443E-15
619	APOL1	-6.6353615	2.5909E-65	7.4934E-64
620	CEACAM4	-6.6103065	0.02837204	0.04348653
621	SLC38A4	-6.6098618	5.2701E-06	1.2956E-05
622	MPV17L	-6.6097952	4.3202E-25	3.4821E-24
623	SPATA41	-6.5949939	4.7368E-11	1.7687E-10
624	ASPDH	-6.5941977	1.5465E-35	1.8604E-34
625	TIGIT	-6.5868467	0.03022857	0.04614538
626	MT1JP	-6.5868467	0.03022857	0.04614538
627	EFCC1	-6.5846393	0.00086832	0.00167776

628	CD40	-6.5838486	4.3986E-42	6.4871E-41
629	PLP1	-6.5821657	2.0808E-07	5.8095E-07
630	EVA1A	-6.579142	5.6984E-36	6.9504E-35
631	KCNMB1	-6.577751	5.3378E-08	1.5675E-07
632	LOC391322	-6.5675656	0.00094798	0.00182202
633	ZNF717	-6.5629992	0.03219438	0.04885676
634	IL21R	-6.5629186	7.0554E-06	1.7157E-05
635	GIMAP2	-6.5604936	4.6395E-15	2.2584E-14
636	PGM5	-6.5562342	0.0018037	0.003343
637	TXNRD2	-6.5504673	2.051E-109	1.348E-107
638	NPTX2	-6.5459143	1.3143E-08	4.0536E-08
639	PTPRB	-6.5458468	3.1235E-62	8.3351E-61
640	ZNF502	-6.5333328	7.5283E-09	2.3654E-08
641	CYP2A13	-6.5327997	0.00114392	0.0021731
642	SDPR	-6.5051689	1.3338E-56	3.0103E-55
643	LDHD	-6.4982584	1.1877E-57	2.7382E-56
644	STEAP4	-6.4982376	1.536E-162	2.12E-160
645	LINC00987	-6.4907827	1.3547E-24	1.0678E-23
646	HAL	-6.4870603	1.8005E-15	8.9724E-15
647	MAT1A	-6.4844472	2.136E-154	2.563E-152
648	MASP2	-6.475915	4.07E-139	4.054E-137
649	PTGDS	-6.4742198	4.3654E-22	3.0536E-21
650	CDH5	-6.47346	7.7708E-27	6.7536E-26
651	DOCK10	-6.4674745	3.1398E-19	1.9178E-18
652	SLC37A2	-6.4608206	2.2782E-16	1.1889E-15
653	GSTM1	-6.4593483	4.835E-197	9.731E-195
654	GDA	-6.4572009	2.9754E-67	9.0241E-66
655	RUNX3	-6.4545772	2.6053E-11	9.932E-11
656	CYBB	-6.448694	3.5321E-80	1.4012E-78
657	SERPINA6	-6.4440963	1.094E-278	4.596E-276
658	LOC100506388	-6.4420333	0.00174742	0.00324398
659	ARHGAP30	-6.4409813	1.3584E-39	1.8508E-38
660	SUCNR1	-6.4402256	1.2285E-09	4.119E-09
661	HLA-A	-6.4236956	5.505E-151	6.331E-149
662	IKZF1	-6.42188	2.6828E-20	1.7232E-19
663	MYOM1	-6.4182029	5.255E-117	3.86E-115
664	PRR29	-6.4172947	3.0184E-28	2.7903E-27
665	ALDH1L1	-6.4003801	4.4509E-59	1.0776E-57
666	TNFSF8	-6.3994589	5.9588E-05	0.00013193
667	EMR2	-6.3672257	2.9502E-16	1.5338E-15
668	HSPB2	-6.3620026	2.0202E-05	4.7002E-05
669	XDH	-6.3505472	1.356E-151	1.578E-149
670	FAM78B	-6.3497533	6.7541E-05	0.00014862
671	HSPB6	-6.3466294	1.5449E-05	3.6373E-05
672	CLDN5	-6.3457519	7.9698E-15	3.8141E-14

673	GGT6	-6.3451707	0.00264758	0.00479887
674	FLT4	-6.3448195	8.8756E-46	1.4458E-44
675	GALNT15	-6.3437149	1.415E-16	7.4816E-16
676	SLC22A7	-6.3409266	6.1798E-16	3.1653E-15
677	ITGAX	-6.3344318	2.5519E-12	1.0382E-11
678	GNLY	-6.3249931	0.00262659	0.00476351
679	OSCAR	-6.3220627	5.9559E-09	1.8916E-08
680	SMIM24	-6.3118245	2.6784E-24	2.0765E-23
681	DNASE1L3	-6.3115727	4.1508E-64	1.1522E-62
682	NCF1	-6.304528	0.00324155	0.00579931
683	ST8SIA6	-6.304528	0.00324155	0.00579931
684	USP32P2	-6.3025988	4.4371E-09	1.4223E-08
685	RSAD2	-6.3019842	3.3713E-16	1.7476E-15
686	IL12RB1	-6.3009742	8.2086E-08	2.3684E-07
687	LAPTM5	-6.2976628	1.703E-101	9.793E-100
688	CCL4	-6.2952884	5.0004E-05	0.00011161
689	DES	-6.2863722	1.5989E-11	6.1832E-11
690	TREM2	-6.2837692	0.00353243	0.0062871
691	FBP1	-6.2778252	0	0
692	NFAM1	-6.2635099	3.042E-30	2.9986E-29
693	GLT1D1	-6.2628101	1.8175E-52	3.6426E-51
694	FAM163B	-6.2627076	0.00369193	0.00655349
695	PCSK9	-6.2589138	1.008E-157	1.29E-155
696	WHAMMP2	-6.2572148	8.5165E-07	2.2524E-06
697	CCDC144B	-6.2410909	0.00272077	0.00492185
698	SEC14L4	-6.241005	4.1092E-20	2.6115E-19
699	C1S	-6.2401926	3.2995E-65	9.5002E-64
700	CD226	-6.2392093	1.2845E-09	4.3011E-09
701	CD93	-6.2391923	1.0323E-28	9.686E-28
702	PCAT19	-6.234168	1.9896E-05	4.6324E-05
703	ADAMTS4	-6.2338024	2.9466E-11	1.1195E-10
704	SNX29P2	-6.232014	6.8229E-13	2.8844E-12
705	CYP39A1	-6.2294796	8.2868E-63	2.2392E-61
706	C8B	-6.2267329	1.2874E-29	1.243E-28
707	SULT2A1	-6.2224597	1.161E-272	4.672E-270
708	EVPL	-6.2196381	0.00456706	0.00798321
709	TMC8	-6.2175136	9.408E-29	8.8406E-28
710	HPD	-6.2161381	4.7883E-64	1.3254E-62
711	CD52	-6.2032015	7.0049E-10	2.3915E-09
712	AR	-6.1999309	1.4905E-97	8.1578E-96
713	TXLNB	-6.1976119	0.00473017	0.00824939
714	GPR116	-6.196288	1.2838E-48	2.2796E-47
715	LINC00662	-6.1948185	3.7065E-09	1.1957E-08
716	PLIN4	-6.1932616	4.4242E-59	1.0725E-57
717	PREX2	-6.192986	2.4597E-10	8.7099E-10

718	KCNIP3	-6.1913859	4.9204E-09	1.5718E-08
719	PPP1R16B	-6.1748427	4.0785E-19	2.4701E-18
720	VWF	-6.1731665	5.361E-31	5.4285E-30
721	LOC101927151	-6.1723235	1.5363E-08	4.7105E-08
722	CPN1	-6.1577906	2.337E-188	4.181E-186
723	FIGF	-6.1525231	0.00582626	0.01000741
724	MEFV	-6.1487937	8.8065E-06	2.1199E-05
725	MEG3	-6.1445526	5.3736E-18	3.0679E-17
726	ODF3B	-6.1199013	3.6926E-31	3.7647E-30
727	NFIC	-6.113386	2.2206E-67	6.7668E-66
728	NEU4	-6.1115487	8.1796E-09	2.5625E-08
729	EPHX1	-6.1096909	0	0
730	HTRA1	-6.0974478	7.7293E-09	2.4262E-08
731	CASQ2	-6.0899977	0.00032224	0.00065595
732	NPL	-6.0862524	7.5904E-55	1.6097E-53
733	IDO2	-6.0852807	2.2481E-05	5.2054E-05
734	ADCYAP1R1	-6.0826956	0.00029945	0.00061188
735	FBXL22	-6.0821331	0.00729552	0.01236724
736	UGT2B10	-6.0794098	1.4192E-05	3.3532E-05
737	PRR26	-6.0750091	1.3796E-13	6.1133E-13
738	LGALS9	-6.0740213	1.5257E-22	1.0869E-21
739	KCNQ1	-6.0718053	1.2407E-16	6.5811E-16
740	CDHR5	-6.0684039	4.8276E-11	1.8013E-10
741	ERG	-6.0680758	5.3965E-31	5.4587E-30
742	LINC01488	-6.0600728	1.5694E-20	1.0182E-19
743	CRISP3	-6.0578853	0.00793866	0.01337806
744	CASS4	-6.0571934	4.4171E-07	1.1987E-06
745	DDO	-6.0533243	7.2402E-41	1.027E-39
746	SIGLEC5	-6.0332226	0.00899234	0.01500925
747	GBP4	-6.0330218	1.2935E-47	2.2253E-46
748	PLIN5	-6.0329218	1.125E-87	5.1506E-86
749	EXOC3L2	-6.0321213	7.3054E-27	6.3577E-26
750	CRHBP	-6.0274406	3.515E-109	2.302E-107
751	C1R	-6.0222187	2.2847E-81	9.2536E-80
752	C5AR1	-6.0183514	6.2247E-20	3.911E-19
753	IL4I1	-6.0178322	2.066E-264	7.129E-262
754	ARHGAP15	-6.0092277	3.308E-10	1.1599E-09
755	ITGB2-AS1	-6.0081323	0.00869223	0.01454707
756	CES1P2	-6.0081316	0.00939485	0.01563381
757	HID1-AS1	-6.0081312	0.00977967	0.01622805
758	IFITM3	-6.0020318	8.752E-116	6.309E-114
759	CCDC3	-5.9967088	1.0981E-13	4.8927E-13
760	GCKR	-5.9958462	4.461E-122	3.699E-120
761	LOC100422737	-5.9927484	2.7644E-10	9.7569E-10
762	CYP2D7P	-5.9870099	1.709E-38	2.2599E-37

763	P2RY12	-5.9859108	4.193E-12	1.6824E-11
764	ZNF300P1	-5.9825965	0.00986173	0.01634877
765	SLC9A9	-5.9815187	1.0969E-34	1.2766E-33
766	TPRG1	-5.9809329	0.0002812	0.00057643
767	CD8A	-5.9727296	2.0286E-15	1.0083E-14
768	GPX2	-5.9722065	1.1324E-38	1.5057E-37
769	NAT8	-5.9592672	2.0097E-10	7.1625E-10
770	ONECUT1	-5.9575135	0.00059327	0.00116851
771	LINC00092	-5.9566003	0.01156043	0.01895185
772	BCHE	-5.9481315	9.0138E-52	1.7788E-50
773	GPR34	-5.9480293	5.0835E-18	2.9066E-17
774	GRM8	-5.9462854	7.5876E-23	5.4842E-22
775	CAMK2N2	-5.9364637	0.00062254	0.00122342
776	TLR3	-5.9358804	9.708E-18	5.481E-17
777	PTPRH	-5.9356609	1.7195E-30	1.7107E-29
778	FCRL6	-5.9324273	3.7027E-05	8.3885E-05
779	MICA	-5.9293933	5.9436E-06	1.4539E-05
780	POU6F2-AS1	-5.9291512	3.7042E-05	8.3909E-05
781	EOMES	-5.9262249	0.00037611	0.00075771
782	PGR	-5.9170977	0.00066408	0.00130056
783	ECSCR	-5.9165075	6.4389E-08	1.8772E-07
784	CD97	-5.9068027	2.473E-106	1.531E-104
785	CLEC12A	-5.9048398	3.396E-08	1.0136E-07
786	CTSW	-5.9005217	7.6405E-09	2.3991E-08
787	NCF1C	-5.8974672	0.00072778	0.00141883
788	APOL4	-5.8968964	1.2346E-07	3.5171E-07
789	COLEC10	-5.8942036	9.2076E-08	2.6456E-07
790	DNAJC12	-5.8929628	8.917E-45	1.4121E-43
791	NFIB	-5.8897948	7.6399E-41	1.0821E-39
792	TLR4	-5.887938	5.629E-14	2.5613E-13
793	GYS2	-5.8832297	8.709E-104	5.193E-102
794	LOC101928398	-5.8756803	0.01322398	0.02148396
795	TRIM55	-5.8616815	0.00190807	0.0035236
796	AQP7P3	-5.8603315	0.00108094	0.00205954
797	MZB1	-5.8556113	5.204E-05	0.00011591
798	PDE6G	-5.8476644	0.01611514	0.02579276
799	IL7	-5.8462475	1.1292E-05	2.6894E-05
800	COLEC11	-5.8287221	6.3553E-26	5.2947E-25
801	CD96	-5.8245553	0.00493194	0.00857266
802	TYMP	-5.8218475	5.8502E-55	1.2462E-53
803	GK	-5.8165717	2.431E-131	2.205E-129
804	RERGL	-5.8161533	0.00119911	0.00227104
805	MX2	-5.811851	6.3511E-12	2.5175E-11
806	LINC00313	-5.8057211	1.773E-09	5.8695E-09
807	BGN	-5.8032749	2.0047E-40	2.7985E-39

808	NECAB2	-5.8017508	4.7669E-16	2.4533E-15
809	SLC17A2	-5.7986946	7.2065E-30	7.0318E-29
810	RHCG	-5.7919605	0.00054647	0.00108042
811	GIPC3	-5.7840147	3.0822E-17	1.6898E-16
812	SNX20	-5.780979	2.9896E-05	6.8369E-05
813	MYEOV	-5.7729392	0.00035751	0.00072243
814	FITM1	-5.7672144	7.624E-27	6.632E-26
815	CD300E	-5.7542073	0.00171504	0.00318694
816	SCIMP	-5.7518609	7.7602E-17	4.1554E-16
817	GREM2	-5.7512181	1.5211E-58	3.6191E-57
818	S1PR4	-5.7469419	9.4145E-06	2.26E-05
819	PZP	-5.7443749	9.6944E-48	1.6753E-46
820	CLEC1A	-5.738709	4.2269E-06	1.0483E-05
821	IL21R-AS1	-5.7299919	0.00156758	0.00292785
822	ANXA10	-5.7228219	1.4291E-33	1.5886E-32
823	MYLK	-5.7180145	4.776E-269	1.845E-266
824	TRPA1	-5.7168026	0.01513723	0.02434457
825	ADAMTS17	-5.7146835	7.4559E-26	6.1876E-25
826	SLC2A2	-5.7087945	1.82E-28	1.6938E-27
827	CRIP2	-5.7083604	1.932E-23	1.4373E-22
828	LRRC25	-5.7073216	2.1596E-09	7.0983E-09
829	CDHR2	-5.6897136	3.7664E-57	8.5809E-56
830	F8	-5.6722165	8.1074E-93	4.006E-91
831	CXCL6	-5.6696299	1.7088E-07	4.8034E-07
832	NT5E	-5.6691871	3.6746E-18	2.1205E-17
833	LINC01124	-5.6672587	5.2991E-06	1.3024E-05
834	APOL3	-5.6671122	4.0668E-58	9.5586E-57
835	PTCRA	-5.6670897	0.02635854	0.04064675
836	CBFA2T3	-5.6666343	1.2688E-15	6.3836E-15
837	ZAN	-5.6627724	0.00151391	0.00283363
838	PTPN22	-5.6557724	0.02060297	0.03239335
839	ZNF812	-5.6556722	1.9258E-05	4.494E-05
840	DHODH	-5.6502732	1.972E-169	2.977E-167
841	C10orf11	-5.6488641	2.3327E-19	1.4344E-18
842	HOGA1	-5.645069	1.407E-110	9.371E-109
843	RPS27	-5.6443169	1.683E-120	1.343E-118
844	LINC01197	-5.6421231	0.00019601	0.00040892
845	PTH1R	-5.6348945	4.8917E-14	2.2332E-13
846	STARD8	-5.6194104	3.1613E-70	1.0196E-68
847	USHBP1	-5.6122658	2.2296E-10	7.9243E-10
848	MGC32805	-5.6012523	1.0273E-09	3.4669E-09
849	ITIH1	-5.6002193	9.736E-163	1.353E-160
850	HSPA7	-5.5997921	0.02098707	0.0329597
851	LPL	-5.5994722	3.2554E-09	1.0562E-08
852	LINC00870	-5.5947254	3.3106E-07	9.084E-07

853	CELA3A	-5.5920452	6.3357E-05	0.0001398
854	MYCT1	-5.5879581	2.2201E-29	2.1297E-28
855	CEACAM1	-5.585352	1.7517E-42	2.6134E-41
856	ANGPTL1	-5.5738569	1.1806E-13	5.2471E-13
857	CDH23	-5.5701025	9.8453E-20	6.1498E-19
858	EMILIN1	-5.5682455	9.1524E-18	5.1749E-17
859	GNAO1	-5.5567397	1.5269E-27	1.367E-26
860	CYP4F22	-5.5530988	1.4058E-09	4.694E-09
861	SHE	-5.5524072	2.3444E-45	3.7744E-44
862	CDH15	-5.5327892	0.03058382	0.04662875
863	HCST	-5.532167	2.7795E-09	9.0602E-09
864	C1orf162	-5.5267764	1.6266E-12	6.7133E-12
865	CYTIP	-5.5234761	2.284E-07	6.3547E-07
866	LMO2	-5.5208557	3.8907E-29	3.6956E-28
867	LINC00961	-5.5207386	0.00123732	0.00234018
868	FGD5	-5.5204017	1.5914E-44	2.5037E-43
869	MILR1	-5.5193529	0.00364303	0.00647203
870	PSTPIP1	-5.5155259	2.1322E-07	5.9478E-07
871	CHRNA4	-5.5110399	6.1283E-57	1.3897E-55
872	SLC11A1	-5.50984	7.0496E-13	2.979E-12
873	IRAK3	-5.5080883	1.7423E-11	6.7119E-11
874	AMDHD1	-5.5078658	4.7236E-96	2.5003E-94
875	SV2B	-5.5059063	0.00719388	0.0122078
876	MUC2	-5.4994727	0.00356412	0.00633942
877	SLC12A1	-5.4988009	0.02718467	0.04181924
878	HOXA5	-5.4983174	0.00268339	0.00485969
879	RDH12	-5.4875209	3.9107E-14	1.7968E-13
880	PSMC1	-5.4850528	1.8419E-85	8.1808E-84
881	SCN4A	-5.4821086	0.00297985	0.00536141
882	HLA-C	-5.4817869	9.1745E-95	4.7141E-93
883	CYP2C18	-5.4755216	9.2425E-48	1.5986E-46
884	TGM2	-5.4737511	5.0553E-36	6.1855E-35
885	TOX2	-5.4705105	1.9871E-09	6.557E-09
886	SLC22A31	-5.4680263	0.00235166	0.0042911
887	GZMM	-5.4632665	0.00018976	0.00039659
888	ELN	-5.4628413	2.9633E-66	8.8214E-65
889	SLC4A1	-5.461908	0.00522763	0.00904593
890	NAT2	-5.4598929	9.2829E-19	5.5081E-18
891	TTC36	-5.4550317	1.4694E-69	4.6768E-68
892	GMFG	-5.4526303	1.1048E-25	9.1061E-25
893	LOC101927571	-5.4515529	0.00397782	0.00701996
894	CABP4	-5.4511203	5.5462E-06	1.3602E-05
895	RTN4RL1	-5.4461353	3.9445E-39	5.3069E-38
896	ACSL1	-5.4430182	4.7126E-71	1.5564E-69
897	ENPP3	-5.4426182	0.00022641	0.00046849

898	CNTD2	-5.4417323	1.3368E-19	8.2936E-19
899	GPR4	-5.4404734	1.663E-17	9.2483E-17
900	RAB40B	-5.4290881	2.7429E-45	4.4124E-44
901	LYL1	-5.4234223	2.5722E-11	9.8133E-11
902	CYP4V2	-5.4194228	2.722E-105	1.67E-103
903	NRROS	-5.4102228	2.087E-14	9.7275E-14
904	KCNK17	-5.4056347	0.02855925	0.04374614
905	CLIC2	-5.4040593	4.2489E-23	3.1094E-22
906	CXCL10	-5.4016914	0.00033575	0.00068137
907	PLG	-5.3946821	1.9828E-11	7.6189E-11
908	LOC101927688	-5.3912054	1.0678E-09	3.5992E-09
909	RAC2	-5.3909633	3.057E-16	1.5881E-15
910	CD200R1	-5.3869669	8.3187E-09	2.6044E-08
911	ARHGEF15	-5.3805653	1.0472E-12	4.3811E-12
912	TDRD10	-5.36741	1.1689E-05	2.7788E-05
913	PDE7B	-5.3647957	9.0004E-28	8.1256E-27
914	CCBE1	-5.364092	0.00546067	0.00942471
915	CYSLTR1	-5.3626947	0.0003407	0.00069055
916	NNMT	-5.3604119	3.4696E-44	5.3929E-43
917	SGCA	-5.3589462	2.7139E-07	7.4893E-07
918	TLR7	-5.3578357	0.00648819	0.01108033
919	PDK4	-5.3544588	1.9353E-19	1.1946E-18
920	TIE1	-5.3532796	6.6276E-21	4.3836E-20
921	IFI6	-5.3391488	3.654E-108	2.345E-106
922	ZNF366	-5.3318909	3.0995E-10	1.09E-09
923	ADAMTS2	-5.3316222	2.5682E-05	5.9103E-05
924	KIAA1755	-5.3311392	8.9182E-05	0.00019373
925	FAM20A	-5.3296087	1.1098E-41	1.6206E-40
926	PDE4B	-5.327677	4.3803E-12	1.7569E-11
927	LPAR5	-5.3254404	2.071E-06	5.2947E-06
928	PPARG	-5.3202406	3.6981E-13	1.5902E-12
929	ENPEP	-5.315675	2.4458E-47	4.1706E-46
930	GLYATL1	-5.3140698	4.2281E-48	7.3992E-47
931	SDSL	-5.3105101	5.0154E-71	1.6535E-69
932	RARRES2	-5.3080908	3.0391E-95	1.5826E-93
933	PLGLB1	-5.297171	9.9779E-20	6.2225E-19
934	PLGLB2	-5.297171	9.9779E-20	6.2225E-19
935	RPL9	-5.2949639	6.394E-244	1.844E-241
936	TACR2	-5.2819844	2.9272E-05	6.7006E-05
937	LPA	-5.2762327	1.0497E-39	1.4363E-38
938	RPS17	-5.2728185	1.0384E-05	2.4813E-05
939	WHAMMP3	-5.2715624	9.7835E-09	3.0511E-08
940	ZEB2-AS1	-5.2621438	0.00843258	0.01413846
941	WDR72	-5.2553431	2.476E-55	5.3628E-54
942	ITGB7	-5.2505302	6.4737E-16	3.3088E-15

943	ASCL2	-5.2503168	0.0052954	0.00915336
944	CEACAM21	-5.2502588	4.1467E-07	1.1273E-06
945	ADRB2	-5.2483554	9.1663E-13	3.844E-12
946	PTPN7	-5.2442573	1.3676E-06	3.5527E-06
947	LOC100286922	-5.2441052	0.00680308	0.01158431
948	CTSF	-5.2416902	4.8068E-82	1.9717E-80
949	SMOC1	-5.2307486	2.986E-31	3.0605E-30
950	PECAM1	-5.2284698	4.6255E-20	2.9271E-19
951	PNPO	-5.2282516	6.5397E-70	2.0884E-68
952	C10orf105	-5.2261956	1.0552E-05	2.5206E-05
953	SERPINA11	-5.2255707	3.2108E-35	3.7987E-34
954	TRIM47	-5.2247868	3.0368E-38	3.9912E-37
955	UGT2A2	-5.2142493	3.3884E-19	2.0632E-18
956	PEMT	-5.2139066	4.4188E-79	1.7247E-77
957	TMEM74	-5.2122345	1.1554E-06	3.0219E-06
958	ACACB	-5.2119081	6.962E-135	6.561E-133
959	LY96	-5.2113669	1.3305E-11	5.175E-11
960	NOV	-5.2090877	3.1037E-07	8.5297E-07
961	CYP17A1	-5.2079675	0.01060353	0.01748103
962	HNF4A-AS1	-5.2008921	1.1722E-19	7.289E-19
963	MSR1	-5.1994178	1.9495E-13	8.5677E-13
964	CX3CR1	-5.1972433	4.8149E-07	1.3014E-06
965	MYO1G	-5.1964517	1.7727E-11	6.8265E-11
966	PAQR9-AS1	-5.190397	0.00079832	0.00154855
967	ASS1	-5.1888937	3.544E-105	2.167E-103
968	SYTL4	-5.1845433	3.3543E-87	1.5212E-85
969	DSG1	-5.1842975	0.00824028	0.01384849
970	SLAMF8	-5.1839964	2.637E-07	7.2855E-07
971	LOC730102	-5.1795188	3.9426E-33	4.3403E-32
972	MLPH	-5.1794382	2.6515E-18	1.5425E-17
973	ENAM	-5.1793486	0.00056044	0.00110679
974	TMEM156	-5.1773746	0.00828091	0.01391194
975	GFRA1	-5.1755404	3.1033E-27	2.7452E-26
976	IFIT2	-5.1710223	4.677E-43	7.0704E-42
977	UBE2QL1	-5.1698173	1.2754E-27	1.1471E-26
978	GPD1	-5.1586244	3.1357E-20	2.0067E-19
979	SLC38A3	-5.1585296	1.0869E-10	3.9524E-10
980	PCDH1	-5.1486744	2.846E-111	1.916E-109
981	SOX18	-5.1391457	2.1373E-23	1.5888E-22
982	EPHA3	-5.1382151	1.2189E-07	3.4749E-07
983	PDE1B	-5.1371913	3.0058E-15	1.4792E-14
984	CCR5	-5.1351553	9.9353E-07	2.6141E-06
985	SLCO3A1	-5.1340622	2.3277E-21	1.5724E-20
986	CALCA	-5.1337959	0.01229848	0.02008001
987	PRG2	-5.1321418	1.3549E-10	4.892E-10

988	LINC01484	-5.1263937	0.00118458	0.00224547
989	ABI3	-5.1102399	5.4853E-17	2.9635E-16
990	TDRD9	-5.1101828	0.01369764	0.02218818
991	HSD17B6	-5.1083037	6.5058E-31	6.5533E-30
992	LINC00907	-5.1064726	0.00043598	0.00087242
993	EMCN	-5.10578	8.882E-14	3.9879E-13
994	ITIH4-AS1	-5.1034451	8.1889E-30	7.9783E-29
995	PRODH2	-5.1001101	5.7411E-22	3.9956E-21
996	CTRB1	-5.0975797	0.0020625	0.00379212
997	STX1B	-5.094316	1.5211E-10	5.4696E-10
998	SYK	-5.0828211	8.942E-29	8.4068E-28
999	ARHGAP9	-5.0783564	2.0425E-17	1.1323E-16
1000	LOC606724	-5.0782949	8.1978E-05	0.00017878
1001	CXCL12	-5.077709	1.3272E-06	3.4521E-06
1002	LOC101929384	-5.0756607	2.3822E-05	5.4994E-05
1003	PTGIR	-5.0672354	1.3372E-07	3.7982E-07
1004	SP5	-5.0671623	6.9235E-06	1.6859E-05
1005	NRXN2	-5.0642523	2.4033E-20	1.5452E-19
1006	S1PR1	-5.0617185	1.5826E-17	8.8215E-17
1007	PYHIN1	-5.0587673	0.0176858	0.02812723
1008	SERPINA1	-5.0580481	1.0751E-30	1.0756E-29
1009	MMRN2	-5.05689	1.0003E-59	2.4777E-58
1010	TUBA4A	-5.0492592	3.3419E-78	1.276E-76
1011	ADRA2B	-5.0473176	4.9837E-60	1.2488E-58
1012	SPN	-5.0428358	1.0234E-10	3.7324E-10
1013	RPS3A	-5.0379437	2.177E-193	4.165E-191
1014	ZNF572	-5.0345383	0.00225026	0.00411578
1015	SHMT1	-5.0333727	0	0
1016	MYOM2	-5.0211722	3.6759E-09	1.1866E-08
1017	LOC100507053	-5.0196213	1.6878E-46	2.811E-45
1018	MIP	-5.0193824	0.00093021	0.00179
1019	ART4	-5.0182641	1.3176E-50	2.4933E-49
1020	ALDH8A1	-5.0161858	3.4719E-35	4.1E-34
1021	CCAT1	-5.0143692	4.9024E-06	1.2092E-05
1022	GPR88	-5.0108964	3.0943E-26	2.614E-25
1023	PRAP1	-5.0062401	1.4136E-59	3.4834E-58
1024	LSP1	-5.0057963	1.2009E-13	5.331E-13
1025	AGMAT	-5.0039226	5.3458E-62	1.4148E-60
1026	AKR7L	-5.0031563	2.5286E-47	4.3041E-46
1027	CLEC11A	-5.0012988	3.3367E-25	2.7029E-24
1028	SLC8A1	-4.9978848	9.5782E-10	3.2403E-09
1029	ELTD1	-4.9960524	1.951E-14	9.1092E-14
1030	DOC2B	-4.9929052	0.01796115	0.02853227
1031	CADM2	-4.9927685	0.00138893	0.00261033
1032	DBH	-4.9918283	1.9419E-37	2.4863E-36

1033	HULC	-4.9909933	0.00019372	0.00040427
1034	TCF21	-4.9908049	0.00376456	0.00667138
1035	RASIP1	-4.9866809	2.5329E-31	2.6071E-30
1036	SH2D3C	-4.9847845	1.3467E-21	9.2165E-21
1037	C1QTNF7	-4.9774079	7.8598E-05	0.00017172
1038	DOK7	-4.9710959	3.1822E-05	7.2577E-05
1039	GPT	-4.9584104	1.7139E-59	4.202E-58
1040	SPRYD4	-4.9561842	2.951E-174	4.599E-172
1041	BMX	-4.9510335	0.00737753	0.0124953
1042	ITIH4	-4.950067	3.8453E-30	3.7788E-29
1043	GCSH	-4.9468623	1.5818E-35	1.9005E-34
1044	APOH	-4.9455809	3.2678E-11	1.2365E-10
1045	DHRS4L2	-4.9433802	2.591E-39	3.5129E-38
1046	FGFBP2	-4.9427055	0.00319854	0.00572926
1047	AKR1B15	-4.938279	0.00185153	0.00342344
1048	ACAT1	-4.9365755	7.8382E-89	3.6315E-87
1049	SPDYE8P	-4.9360237	0.00358837	0.00637844
1050	RASGRP4	-4.9339075	1.1531E-12	4.8084E-12
1051	TNFAIP8L2	-4.9327418	7.164E-10	2.4441E-09
1052	CHRM2	-4.9324496	0.00074819	0.00145642
1053	RTP4	-4.930197	1.0293E-06	2.7059E-06
1054	NRN1	-4.926661	4.4614E-16	2.2991E-15
1055	MYO1F	-4.9249367	6.7935E-45	1.0826E-43
1056	SIGLEC7	-4.9208923	1.5935E-11	6.1634E-11
1057	SPDYC	-4.9160334	0.00247154	0.00449793
1058	ACSL6	-4.907338	2.893E-10	1.0196E-09
1059	HTR2B	-4.9027436	1.6648E-06	4.2932E-06
1060	RNASE2	-4.8999541	4.6557E-05	0.00010437
1061	AKR1B10	-4.8974974	4.0525E-38	5.2972E-37
1062	PCBP3	-4.8900996	0.00091137	0.00175586
1063	ARHGAP20	-4.8813166	2.1205E-13	9.298E-13
1064	SHISA3	-4.8806981	0.00016511	0.00034749
1065	PDE1A	-4.8806068	9.0578E-16	4.5955E-15
1066	IGFBP7-AS1	-4.8714768	9.4398E-18	5.3311E-17
1067	PLA2G5	-4.8700316	1.2646E-08	3.9041E-08
1068	MMP7	-4.8644687	0.00017633	0.00036993
1069	METTL7B	-4.8613468	4.3047E-69	1.3567E-67
1070	BCL6B	-4.8613273	4.03E-11	1.5118E-10
1071	GNGT2	-4.8578821	0.00076936	0.00149463
1072	CFD	-4.8560792	7.029E-27	6.1226E-26
1073	FAM198A	-4.8517394	1.3621E-36	1.6891E-35
1074	FLI1	-4.8479623	3.4488E-22	2.423E-21
1075	LINC00525	-4.845368	0.0179255	0.02848266
1076	POR	-4.8444054	2.779E-268	1.032E-265
1077	CLMN	-4.8404529	1.872E-42	2.7907E-41

1078	ABLIM2	-4.8390533	7.6665E-07	2.0354E-06
1079	MASP1	-4.8376245	3.9227E-30	3.8529E-29
1080	SLC9A3R2	-4.8359842	2.0743E-56	4.6654E-55
1081	CD27-AS1	-4.8359173	2.3919E-09	7.8433E-09
1082	LOC100128531	-4.8282107	4.5105E-27	3.9664E-26
1083	SRD5A1	-4.8255665	5.601E-148	6.219E-146
1084	SYPL2	-4.8201197	4.0036E-17	2.1807E-16
1085	HLA-DQB1	-4.8174495	2.2426E-13	9.8093E-13
1086	MCHR1	-4.8123277	9.3318E-13	3.9117E-12
1087	MPZ	-4.809459	1.1144E-16	5.9215E-16
1088	CCL28	-4.8088867	0.02420526	0.03756793
1089	SLITRK3	-4.8070173	1.6068E-40	2.2512E-39
1090	HPS3	-4.7982965	0	0
1091	LRRC73	-4.7955	2.0721E-06	5.2966E-06
1092	HAND2-AS1	-4.7842913	0.03116559	0.04744459
1093	SELPLG	-4.779187	2.0142E-35	2.4081E-34
1094	MOCOS	-4.7763297	1.0408E-58	2.4918E-57
1095	THBS2	-4.7754774	7.6979E-10	2.6207E-09
1096	CCR1	-4.7742696	1.9855E-12	8.1307E-12
1097	HLA-DRB3	-4.7705512	3.2328E-17	1.7714E-16
1098	CYP27A1	-4.7705494	1.7688E-75	6.3283E-74
1099	SLA	-4.7678935	1.5574E-39	2.119E-38
1100	HRSP12	-4.7670443	1.888E-119	1.471E-117
1101	TAGAP	-4.7642642	6.3294E-09	2.0073E-08
1102	DUSP6	-4.7606199	1.9032E-68	5.9306E-67
1103	IL2RB	-4.7603443	3.5885E-09	1.1596E-08
1104	GFOD2	-4.7601192	2.2579E-31	2.3278E-30
1105	SELL	-4.7587839	1.1383E-20	7.4473E-20
1106	CD247	-4.7578978	5.8362E-05	0.00012937
1107	CD27	-4.7501628	2.2774E-07	6.3371E-07
1108	EPO	-4.744573	3.4387E-77	1.2875E-75
1109	CYBRD1	-4.7439608	1.9718E-10	7.034E-10
1110	LGALS1	-4.7421133	7.0313E-06	1.71E-05
1111	EBI3	-4.7420391	2.169E-15	1.0761E-14
1112	ACSS1	-4.7389098	2.6695E-16	1.3898E-15
1113	FCER1A	-4.7372822	0.02432121	0.03773577
1114	DPYD-AS1	-4.7372312	6.1684E-11	2.2826E-10
1115	B3GAT1	-4.7359073	4.1561E-18	2.3876E-17
1116	MFNG	-4.7350331	3.8676E-47	6.5259E-46
1117	OLFML1	-4.7338394	9.7129E-08	2.7862E-07
1118	DMRTA1	-4.731104	2.6908E-52	5.3706E-51
1119	PIK3CG	-4.730887	3.495E-05	7.9356E-05
1120	EP300-AS1	-4.728895	0.00274663	0.0049646
1121	ALDH3A1	-4.7273244	5.5949E-12	2.2297E-11
1122	AFM	-4.7240596	3.7354E-05	8.4576E-05

1123	PRELP	-4.7239674	1.3343E-14	6.3059E-14
1124	CYP4F12	-4.7230421	5.0559E-09	1.6135E-08
1125	SLC27A2	-4.722612	2.482E-202	5.213E-200
1126	LTBR	-4.7181183	2.1372E-71	7.0946E-70
1127	ESPN	-4.7176847	6.4323E-40	8.845E-39
1128	QTRT1	-4.7115726	1.8453E-29	1.7755E-28
1129	ARID5A	-4.7108813	3.0319E-61	7.8206E-60
1130	TMEM173	-4.7081129	3.9927E-27	3.5223E-26
1131	RHOJ	-4.7030543	9.1723E-11	3.3575E-10
1132	CD163	-4.7012266	1.7642E-89	8.2931E-88
1133	SHANK3	-4.6957996	7.959E-20	4.986E-19
1134	LINC01268	-4.6941255	0.00124501	0.00235405
1135	KDR	-4.6875962	7.325E-21	4.8284E-20
1136	LOC100506281	-4.6857033	2.3079E-05	5.3361E-05
1137	ETV2	-4.6854318	2.4573E-06	6.2359E-06
1138	LINC00675	-4.6842379	0.00069104	0.00135116
1139	ACKR1	-4.6814241	9.6416E-05	0.00020853
1140	MDGA1	-4.6813119	8.7099E-07	2.3013E-06
1141	KCNJ18	-4.6801755	0.00228654	0.00417859
1142	BLNK	-4.6799376	8.2281E-11	3.0222E-10
1143	LOC145837	-4.6762632	4.0534E-07	1.1028E-06
1144	ASB13	-4.6716938	4.978E-181	8.363E-179
1145	BAALC	-4.6714747	5.0945E-16	2.617E-15
1146	CD68	-4.6693765	2.362E-134	2.215E-132
1147	ZNF662	-4.667915	4.5736E-07	1.2396E-06
1148	UGT2A1	-4.6587783	2.1243E-18	1.2422E-17
1149	FAM86B1	-4.6585488	0.00456988	0.00798645
1150	CPLX1	-4.6566145	3.5601E-21	2.3874E-20
1151	CAMK2B	-4.6556421	1.2861E-10	4.6522E-10
1152	TMEM100	-4.655567	5.2746E-10	1.822E-09
1153	BTN3A3	-4.653444	1.7037E-27	1.5217E-26
1154	GLYAT	-4.6512035	6.9162E-06	1.6844E-05
1155	ADRA1B	-4.6482237	1.0124E-09	3.4172E-09
1156	PMS2L2	-4.6471395	0.00465353	0.00812087
1157	C2CD4D	-4.6452596	0.00504428	0.00875611
1158	IL22RA1	-4.6435114	2.0539E-13	9.0124E-13
1159	WASH7P	-4.6393504	0.0017504	0.00324921
1160	KNDC1	-4.6374249	1.2893E-11	5.0211E-11
1161	MAPT	-4.635226	0.0066188	0.01128543
1162	ASTN1	-4.6339306	0.02676453	0.04122216
1163	ANO3	-4.6273707	4.4154E-09	1.4156E-08
1164	PLCH2	-4.62364	1.0143E-11	3.9726E-11
1165	HSD3B7	-4.6223303	2.147E-146	2.331E-144
1166	TBX15	-4.6217228	1.399E-23	1.0468E-22
1167	RGPD1	-4.6190535	1.9602E-34	2.2583E-33

1168	TENC1	-4.6131033	6.0234E-51	1.1545E-49
1169	ASGR1	-4.6100837	1.8604E-40	2.6009E-39
1170	TSPO	-4.604765	5.1764E-38	6.7482E-37
1171	LOC643355	-4.6038646	0.00098897	0.00189589
1172	ACSM3	-4.6018365	3.84E-113	2.64E-111
1173	SNCG	-4.6009587	1.3483E-06	3.5051E-06
1174	LOC101927755	-4.5994414	0.00011883	0.00025456
1175	LINC00861	-4.5967798	0.01732941	0.02759678
1176	CIITA	-4.5926041	1.8201E-11	7.0062E-11
1177	AKR1C1	-4.5919788	2.8346E-31	2.9084E-30
1178	BOK	-4.5915211	1.756E-126	1.528E-124
1179	PLIN2	-4.5913745	5.8691E-76	2.1274E-74
1180	NOVA2	-4.5895258	0.00101243	0.00193639
1181	ZEB1	-4.5808236	2.4735E-42	3.6703E-41
1182	BEAN1	-4.5797379	0.02372806	0.0368925
1183	HLA-DPA1	-4.579724	0.00016615	0.00034953
1184	CES2	-4.5792011	0	0
1185	NLRP1	-4.5786524	1.1465E-20	7.4983E-20
1186	UNQ6494	-4.5747989	3.0979E-09	1.0067E-08
1187	CRIP3	-4.572114	2.3387E-15	1.1577E-14
1188	NAAA	-4.5696557	8.112E-34	9.1013E-33
1189	IFITM1	-4.558987	7.5769E-19	4.5237E-18
1190	RGS7BP	-4.557598	8.2193E-07	2.1765E-06
1191	ASB9	-4.5552056	2.842E-10	1.0023E-09
1192	CMAHP	-4.5523641	1.2517E-10	4.5339E-10
1193	IL1RL1	-4.5511222	3.6082E-12	1.4523E-11
1194	TMEM220-AS1	-4.54993	1.5617E-32	1.6753E-31
1195	ALDH2	-4.5419235	0	0
1196	TPST2	-4.5387698	5.8324E-79	2.2673E-77
1197	SLC13A3	-4.5364357	6.1111E-31	6.1621E-30
1198	UBA7	-4.5342412	2.7151E-70	8.7866E-69
1199	MKX	-4.5324103	5.5968E-05	0.00012425
1200	CXorf36	-4.5312719	8.4725E-09	2.6508E-08
1201	PAPPA2	-4.527792	5.9045E-20	3.7207E-19
1202	SLC17A3	-4.524663	4.5615E-19	2.7531E-18
1203	TNFAIP8L3	-4.5241673	1.0978E-09	3.6969E-09
1204	HAVCR2	-4.5240769	8.3911E-19	4.9897E-18
1205	SOX7	-4.519085	3.7147E-05	8.4136E-05
1206	SPRN	-4.5189726	8.012E-126	6.879E-124
1207	GPR82	-4.5187864	2.262E-06	5.7662E-06
1208	SLC6A12	-4.5141155	1.7574E-16	9.2336E-16
1209	FRMPD1	-4.5116855	0.00101573	0.00194161
1210	SLC25A34	-4.5116548	8.7061E-28	7.8672E-27
1211	PHYH	-4.5111435	3.184E-193	6.031E-191
1212	HRCT1	-4.5105011	3.2174E-08	9.6298E-08

1213	NPY6R	-4.5074921	0.00152267	0.00284893
1214	DGKG	-4.494891	3.4733E-15	1.6997E-14
1215	LOC283856	-4.4935776	0.00609889	0.01045338
1216	MTHFD1	-4.4878962	2.755E-120	2.19E-118
1217	APOC3	-4.4859844	1.0763E-33	1.2026E-32
1218	SEPHS2	-4.4834875	4.82E-196	9.503E-194
1219	LDB2	-4.483426	9.3526E-15	4.4516E-14
1220	C19orf38	-4.482175	0.00753241	0.01273974
1221	CECR1	-4.4777807	2.1612E-53	4.4371E-52
1222	LAMA4	-4.4762462	8.5644E-11	3.1397E-10
1223	CYP26B1	-4.4756405	5.7228E-10	1.9712E-09
1224	WFDC1	-4.4713522	2.8769E-13	1.2484E-12
1225	RELN	-4.4693176	1.131E-24	8.9483E-24
1226	GMNC	-4.4670317	1.0838E-16	5.7637E-16
1227	C1QTNF5	-4.4661565	5.8497E-19	3.5131E-18
1228	MFRP	-4.4661565	5.8497E-19	3.5131E-18
1229	ABCC11	-4.4608597	5.7689E-14	2.6231E-13
1230	CLEC7A	-4.4604668	0.00045026	0.00089876
1231	PPBP	-4.4594786	0.01673601	0.02670905
1232	LOC100505942	-4.4547556	3.5285E-07	9.6613E-07
1233	TSLP	-4.4511425	2.6031E-10	9.1974E-10
1234	WAS	-4.449269	2.7972E-12	1.1351E-11
1235	NR1D1	-4.4490931	1.6831E-76	6.194E-75
1236	FKBP5	-4.4446321	5.0986E-27	4.4714E-26
1237	LOC101929372	-4.4445579	4.9224E-39	6.595E-38
1238	TMEM220	-4.4386602	1.5E-35	1.8056E-34
1239	LEAP2	-4.4382414	3.9558E-16	2.0451E-15
1240	MGP	-4.4364809	0.01711513	0.02727136
1241	CD3E	-4.4352567	3.0267E-09	9.8444E-09
1242	TMEM255B	-4.4332982	3.2408E-13	1.4013E-12
1243	CCL15-CCL14	-4.4259684	7.5573E-05	0.0001653
1244	SLC43A1	-4.423941	1.2015E-17	6.7402E-17
1245	HIC1	-4.4236686	4.9866E-12	1.993E-11
1246	GPX3	-4.4223627	1.319E-184	2.317E-182
1247	RRN3P1	-4.4145795	0.00099889	0.00191284
1248	IFI30	-4.4117873	2.077E-121	1.693E-119
1249	LPAL2	-4.4103493	1.8566E-24	1.4505E-23
1250	FUT6	-4.4050204	2.943E-22	2.0729E-21
1251	MANEAL	-4.402412	9.955E-22	6.8542E-21
1252	FAM124B	-4.4019763	0.00401214	0.00707637
1253	ARHGEF37	-4.3929698	6.651E-12	2.6348E-11
1254	TESC	-4.3922567	2.6749E-08	8.0484E-08
1255	DDT	-4.3867933	1.636E-67	4.9934E-66
1256	GGTA1P	-4.3794144	1.162E-06	3.0366E-06
1257	PRAM1	-4.3786352	5.5525E-08	1.6281E-07

1258	VIPR1-AS1	-4.3683447	0.0044916	0.00786101
1259	CTSS	-4.3663098	1.1322E-33	1.2637E-32
1260	TBX2	-4.3644505	1.7363E-05	4.0706E-05
1261	HS3ST3B1	-4.3639557	3.9756E-20	2.5291E-19
1262	RBFOX3	-4.3638556	6.361E-07	1.6984E-06
1263	CCND1	-4.3604433	6.2154E-21	4.1208E-20
1264	FAM110D	-4.3592688	4.8642E-05	0.00010871
1265	LONRF2	-4.3584885	3.4058E-07	9.3374E-07
1266	PPP1R3C	-4.3557909	1.3769E-37	1.7676E-36
1267	OR7D2	-4.3532071	0.01246596	0.02032489
1268	CALCRL	-4.3531146	4.2441E-26	3.5728E-25
1269	SYNPO2	-4.3529528	3.6696E-13	1.579E-12
1270	ADCY1	-4.3485758	3.1149E-40	4.3358E-39
1271	TMEM82	-4.3468125	5.8757E-63	1.5944E-61
1272	NPIPA1	-4.3450316	0.01104621	0.01815662
1273	PCK2	-4.343168	2.909E-112	1.979E-110
1274	TDO2	-4.3383297	6.6588E-46	1.0902E-44
1275	IL7R	-4.3312646	5.8855E-08	1.7221E-07
1276	GHRHR	-4.3312162	0.02293262	0.03575934
1277	MDH1B	-4.3312162	0.02293262	0.03575934
1278	HNMT	-4.3305596	7.1147E-38	9.219E-37
1279	KCNAB2	-4.3276988	6.479E-27	5.6614E-26
1280	IGFBP2	-4.3236854	4.2058E-41	6.0189E-40
1281	DENND2D	-4.3197767	4.2388E-32	4.4873E-31
1282	LMO3	-4.3167017	4.0125E-05	9.0573E-05
1283	ITGBL1	-4.3129136	5.3375E-14	2.4315E-13
1284	DPYD	-4.3033833	1.0022E-51	1.9717E-50
1285	DDTL	-4.3022965	9.3582E-65	2.6394E-63
1286	RPL18A	-4.3020681	8.2006E-64	2.2569E-62
1287	CYP3A7	-4.297566	0.00084387	0.00163232
1288	C14orf105	-4.2908376	6.3339E-21	4.198E-20
1289	TAPBPL	-4.2895201	3.5759E-46	5.925E-45
1290	PRSS54	-4.2880003	2.081E-05	4.8351E-05
1291	KLRB1	-4.2874212	0.00305899	0.00549867
1292	ST8SIA3	-4.2856191	0.0060381	0.01035654
1293	FOLH1B	-4.2769572	5.4239E-05	0.00012053
1294	CD99L2	-4.2720513	1.221E-214	2.775E-212
1295	CYP3A7-CYP3AP1	-4.2670599	0.00082997	0.00160687
1296	CMA1	-4.2646516	0.01548265	0.02486905
1297	TMEM132E	-4.25769	0.00443072	0.0077608
1298	CA9	-4.2556139	0.00012617	0.00026953
1299	EPHB1	-4.2553446	5.0005E-09	1.5966E-08
1300	SP110	-4.2519439	4.1185E-23	3.0162E-22
1301	ANKRD44	-4.2475454	3.6588E-39	4.926E-38

1302	SLC43A3	-4.2455218	2.6319E-24	2.0421E-23
1303	LRRC6	-4.243262	0.00091614	0.00176415
1304	SIRPG	-4.2422587	0.02026278	0.03191821
1305	GATM	-4.240908	8.4797E-55	1.7964E-53
1306	SGK2	-4.2344101	2.1283E-13	9.3302E-13
1307	FCAMR	-4.2306354	0.01406822	0.02275032
1308	NHLRC1	-4.22932	3.0135E-17	1.653E-16
1309	GLIDR	-4.2273841	2.2049E-06	5.6274E-06
1310	GOT1	-4.2247646	3.8551E-64	1.0717E-62
1311	FETUB	-4.2243126	9.7486E-08	2.7961E-07
1312	LEPR	-4.223415	3.976E-107	2.486E-105
1313	IGSF21	-4.2224717	0.00564171	0.00970956
1314	NLRP12	-4.2199813	0.00443336	0.0077633
1315	KCNJ12	-4.2141243	6.4919E-05	0.00014306
1316	HMCN2	-4.2055208	2.6843E-05	6.1644E-05
1317	DCPS	-4.2035198	1.109E-119	8.711E-118
1318	PDCD1LG2	-4.2025686	0.00029439	0.00060187
1319	TCTEX1D1	-4.2018522	0.00036986	0.00074566
1320	BACE2	-4.2007598	4.7716E-13	2.0373E-12
1321	GNA14	-4.1939986	1.9033E-06	4.8819E-06
1322	DDX43	-4.1916542	0.02915132	0.04461018
1323	RGS18	-4.1905199	1.9812E-07	5.5386E-07
1324	IGSF6	-4.1860435	1.2046E-08	3.7262E-08
1325	NINJ2	-4.1853302	1.7771E-25	1.4493E-24
1326	APOC1	-4.1807591	7.4919E-56	1.6486E-54
1327	CELA3B	-4.1776408	0.01357351	0.0220037
1328	NDUFA4L2	-4.1727751	1.6602E-09	5.5166E-09
1329	CLU	-4.1726205	2.1758E-40	3.0329E-39
1330	C9orf9	-4.1719429	0.00143545	0.0026933
1331	KIAA0408	-4.1562978	0.018302	0.02904268
1332	LOC100996255	-4.1537329	9.7442E-06	2.3369E-05
1333	PAH	-4.1536044	1.6452E-95	8.6141E-94
1334	GPLD1	-4.1525805	1.231E-124	1.048E-122
1335	ANGPTL6	-4.151999	1.374E-57	3.1639E-56
1336	C19orf35	-4.1485907	0.0166897	0.02663733
1337	FAH	-4.1423677	2.918E-129	2.598E-127
1338	GLOD5	-4.1386014	0.00020362	0.00042373
1339	SCNN1B	-4.1385639	0.02825194	0.04332307
1340	UPB1	-4.137072	2.0316E-37	2.596E-36
1341	NAALADL1	-4.1296816	1.1702E-05	2.7816E-05
1342	THNSL1	-4.1282518	2.2731E-31	2.3422E-30
1343	SLC25A18	-4.1277272	8.5358E-40	1.1704E-38
1344	LGALS4	-4.1204349	4.7878E-28	4.3839E-27
1345	KDM8	-4.1189566	1.5477E-21	1.0544E-20
1346	CEACAM5	-4.1134576	0.00377315	0.00668415

1347	EMP1	-4.111314	1.0982E-09	3.6976E-09
1348	NMNAT3	-4.1059443	1.3828E-06	3.5907E-06
1349	ECM1	-4.1023982	9.8422E-35	1.1469E-33
1350	B2M	-4.1009063	1.771E-167	2.612E-165
1351	PPP1R3G	-4.0985506	1.5111E-07	4.2701E-07
1352	CCM2L	-4.0955364	2.5474E-06	6.4529E-06
1353	CKM	-4.0940168	0.01504824	0.02421556
1354	LINC00899	-4.0882825	0.00255343	0.00463867
1355	SOX10	-4.0871055	0.00374065	0.00663326
1356	SNED1	-4.0864537	5.5775E-33	6.0949E-32
1357	C19orf66	-4.0852771	1.5777E-74	5.5321E-73
1358	MST1P2	-4.0808496	7.7102E-37	9.6917E-36
1359	CPT1A	-4.0790641	6.4506E-06	1.5739E-05
1360	FAM65C	-4.0782377	2.4466E-29	2.3435E-28
1361	C1orf204	-4.0723494	0.00034626	0.00070108
1362	FAM49A	-4.0674855	4.5063E-08	1.331E-07
1363	DPYS	-4.0654882	5.4485E-20	3.4378E-19
1364	ARID3C	-4.0626655	1.0952E-08	3.4013E-08
1365	FAM99B	-4.0537367	0.00112175	0.00213308
1366	LOC100130587	-4.0523454	8.3083E-05	0.00018105
1367	PALM2	-4.0502303	4.9089E-13	2.0936E-12
1368	PIR-FIGF	-4.0472573	3.9789E-46	6.559E-45
1369	LMLN-AS1	-4.045695	0.00510681	0.0088559
1370	TBX2-AS1	-4.0451226	0.01291299	0.02101406
1371	HTR7	-4.0405898	0.01019676	0.01685934
1372	RNA45S5	-4.04025	1.6108E-19	9.9712E-19
1373	VASN	-4.0399901	8.4718E-47	1.4196E-45
1374	APBB1IP	-4.0379343	6.5293E-32	6.8707E-31
1375	ACVRL1	-4.0369445	1.9934E-28	1.8524E-27
1376	RHOH	-4.0368159	0.01363624	0.02209475
1377	LRRTM2	-4.0359262	0.00011536	0.00024741
1378	TNFRSF1B	-4.0316732	1.0191E-39	1.3954E-38
1379	STEAP3	-4.0286737	2.0319E-72	6.9115E-71
1380	TNFSF13	-4.0285121	5.4908E-34	6.1927E-33
1381	MFAP3L	-4.0264567	2.5542E-29	2.4441E-28
1382	ZEB2	-4.0259353	9.0735E-08	2.6082E-07
1383	EBF1	-4.0252348	4.5177E-05	0.00010142
1384	LBP	-4.023795	8.6366E-08	2.4875E-07
1385	SCARA5	-4.0214355	1.1405E-16	6.0586E-16
1386	AKR1C3	-4.0199236	1.4359E-70	4.6939E-69
1387	HDC	-4.0150691	0.00013762	0.0002927
1388	GUCY1B3	-4.0113024	7.4395E-10	2.535E-09
1389	HIST1H2BC	-4.0065364	5.1196E-14	2.3344E-13
1390	LMOD1	-4.0048102	2.784E-19	1.7043E-18
1391	GUCA2B	-4.0045129	0.00644544	0.01101123

1392	TYW1B	-4.0044434	3.8458E-18	2.216E-17
1393	TLR1	-4.0027048	2.719E-15	1.3404E-14
1394	LRP3	-3.9922379	4.4013E-21	2.9342E-20
1395	PITPNM3	-3.991669	6.1523E-26	5.1278E-25
1396	NOTCH1	-3.9909413	1.3422E-25	1.1011E-24
1397	LY9	-3.9897488	0.01321397	0.02147132
1398	LOC101928517	-3.9881444	9.5352E-08	2.7377E-07
1399	LILRA6	-3.9846349	2.6857E-21	1.8104E-20
1400	MSRB1	-3.9788347	8.369E-103	4.885E-101
1401	LCAT	-3.978582	1.6733E-59	4.1182E-58
1402	MB21D1	-3.9748371	0.00388511	0.00686736
1403	HSPB9	-3.9718603	0.00045446	0.00090677
1404	ZIC1	-3.9651716	1.5657E-08	4.7976E-08
1405	KIF6	-3.9629165	0.03158351	0.04800945
1406	CALHM2	-3.9625422	2.0073E-11	7.7114E-11
1407	LOC283070	-3.9595362	6.6611E-11	2.4611E-10
1408	SP140L	-3.9584093	6.5233E-27	5.6976E-26
1409	CLDN10	-3.9579136	9.3933E-07	2.4775E-06
1410	SPRY4	-3.9577441	1.3031E-20	8.4767E-20
1411	VIPR1	-3.9575403	6.9708E-33	7.5831E-32
1412	FAM167B	-3.9499657	1.4489E-08	4.4505E-08
1413	CPT2	-3.9468658	6.776E-224	1.616E-221
1414	GRAMD1C	-3.9438044	9.6839E-15	4.6059E-14
1415	ABCC2	-3.9414329	5.288E-31	5.3573E-30
1416	TRIM16L	-3.9412263	3.3452E-19	2.0381E-18
1417	RGS1	-3.9399332	0.00039641	0.00079695
1418	SLC15A3	-3.9395695	7.331E-24	5.5652E-23
1419	TMLHE-AS1	-3.9384603	0.0148343	0.02389119
1420	LOC101927830	-3.9384603	0.0148343	0.02389119
1421	ANKRD24	-3.9367918	1.4103E-25	1.1546E-24
1422	PIR	-3.9327187	1.8125E-49	3.3098E-48
1423	EFCAB6	-3.9272824	8.4178E-05	0.00018327
1424	FTL	-3.9229442	3.009E-283	1.299E-280
1425	TMEM53	-3.9205353	1.1407E-32	1.2317E-31
1426	PRKAR2B	-3.9192998	1.7078E-07	4.8012E-07
1427	PROC	-3.9188709	4.0348E-30	3.961E-29
1428	LEFTY1	-3.9176617	0.00208312	0.00382784
1429	SEC11C	-3.9174259	3.2047E-41	4.6136E-40
1430	CCL3	-3.9173151	0.00028805	0.0005896
1431	FEZ1	-3.9155249	1.8838E-06	4.8346E-06
1432	CA5A	-3.9154303	9.091E-26	7.5187E-25
1433	NPR1	-3.915339	5.5036E-60	1.3773E-58
1434	DCXR	-3.913504	2.8372E-95	1.4815E-93
1435	AVPR1A	-3.9118549	1.0964E-33	1.2245E-32
1436	NR1I3	-3.9066775	7.1777E-27	6.2493E-26

1437	BPI	-3.9018812	0.00790506	0.01332686
1438	NLRC3	-3.9018597	1.6824E-10	6.0258E-10
1439	CNTFR	-3.9002885	0.02511787	0.0388626
1440	SARDH	-3.8959007	1.4567E-67	4.4602E-66
1441	PPM1E	-3.8954645	6.279E-10	2.1536E-09
1442	LOC100133286	-3.8943851	1.2636E-75	4.5291E-74
1443	TBXAS1	-3.8914213	6.0261E-29	5.6959E-28
1444	DYRK3	-3.8869313	2.6243E-07	7.2523E-07
1445	STX11	-3.8860604	2.4633E-15	1.2168E-14
1446	CD274	-3.8854555	2.9728E-06	7.4824E-06
1447	APOC1P1	-3.8823051	6.9221E-24	5.2651E-23
1448	FGA	-3.8814493	7.9357E-28	7.1812E-27
1449	PXMP2	-3.8750751	8.1072E-96	4.2679E-94
1450	SELENBP1	-3.8711409	2.2792E-65	6.6017E-64
1451	RNF125	-3.8679208	1.8277E-29	1.7594E-28
1452	C1orf226	-3.8674338	2.4353E-78	9.3167E-77
1453	ADSSL1	-3.8651817	1.2344E-23	9.2614E-23
1454	LIME1	-3.8630021	1.5873E-65	4.6253E-64
1455	PIK3IP1	-3.8622538	1.8809E-21	1.275E-20
1456	F8A1	-3.8596741	7.415E-14	3.3458E-13
1457	ATP8B4	-3.8589441	5.6357E-16	2.8896E-15
1458	WDR17	-3.8589308	0.01235997	0.02017187
1459	NLRC5	-3.8561069	6.325E-17	3.4039E-16
1460	OAF	-3.8541787	1.6194E-33	1.7991E-32
1461	EVA1C	-3.8528867	1.6051E-10	5.7597E-10
1462	ITLN1	-3.8472394	6.5384E-07	1.7441E-06
1463	SLC25A15	-3.8470918	9.893E-142	1.022E-139
1464	SPRY1	-3.8374165	8.9801E-30	8.736E-29
1465	LAX1	-3.8366393	0.01777002	0.02824955
1466	PC	-3.8340234	7.8187E-65	2.2182E-63
1467	MLXIPL	-3.8305536	1.2843E-22	9.1998E-22
1468	P2RX6	-3.8278083	9.0558E-05	0.00019652
1469	FAM212A	-3.8278003	0.00521132	0.00901932
1470	TM4SF5	-3.8253794	1.2648E-41	1.8414E-40
1471	KCNK3	-3.8225949	1.2942E-11	5.0392E-11
1472	DAAM2	-3.8182012	2.4453E-27	2.1721E-26
1473	ZFHX4	-3.8156094	1.038E-08	3.2319E-08
1474	SP140	-3.8136498	0.02537078	0.03920994
1475	C9orf152	-3.8125119	0.00723672	0.01227619
1476	PARP10	-3.8105677	1.3269E-62	3.5654E-61
1477	SMPD3	-3.80033	9.7212E-23	7.0053E-22
1478	FAM78A	-3.798697	3.5331E-09	1.1432E-08
1479	DDX60	-3.7986194	8.4551E-15	4.0394E-14
1480	LINC00865	-3.7949916	1.8037E-06	4.6387E-06
1481	ST6GAL2	-3.7941356	2.4473E-15	1.2099E-14

1482	ARRDC4	-3.7941225	6.2943E-07	1.6813E-06
1483	SP100	-3.7929536	2.8997E-54	6.0696E-53
1484	ETV5	-3.7922295	1.4555E-09	4.8558E-09
1485	GNMT	-3.7916072	4.874E-18	2.7926E-17
1486	C15orf65	-3.7887504	0.0095666	0.0158977
1487	PPARGC1B	-3.7836467	1.684E-23	1.2552E-22
1488	TST	-3.7770419	7.687E-213	1.727E-210
1489	TPTE2P5	-3.7739685	4.7776E-88	2.203E-86
1490	C11orf21	-3.7739221	0.01815934	0.02882814
1491	HIST1H2AC	-3.7731665	9.813E-14	4.3906E-13
1492	ST3GAL3	-3.7719601	1.2406E-29	1.199E-28
1493	PTRH1	-3.771881	3.6683E-26	3.0948E-25
1494	IVD	-3.7708908	1.641E-259	5.373E-257
1495	SOD1	-3.7707308	1.045E-188	1.887E-186
1496	FERMT3	-3.7706141	1.6687E-24	1.3068E-23
1497	ETFDH	-3.7700307	1.7306E-34	1.9985E-33
1498	ST3GAL4	-3.7694943	1.7531E-31	1.8132E-30
1499	SLC46A3	-3.7685987	3.151E-28	2.9058E-27
1500	SOBP	-3.7685597	1.0987E-09	3.6989E-09
1501	HADHB	-3.7645694	3.673E-104	2.211E-102
1502	OAS3	-3.7614751	4.4909E-70	1.4413E-68
1503	FAM149A	-3.7562542	3.1733E-38	4.165E-37
1504	IGFBP7	-3.7556655	1.7889E-05	4.1884E-05
1505	CORO1A	-3.755366	2.4631E-26	2.0899E-25
1506	OSBPL5	-3.755047	4.121E-06	1.0231E-05
1507	HIST1H3H	-3.7550309	0.01510228	0.02429442
1508	OIT3	-3.7517468	6.4911E-47	1.0915E-45
1509	APLNR	-3.7513049	3.3697E-15	1.6503E-14
1510	FUT7	-3.7464625	0.02781504	0.04270734
1511	RPS10P7	-3.7455803	0.00287227	0.00517894
1512	SUSD5	-3.7440611	0.00114137	0.00216848
1513	MLKL	-3.7408928	5.0106E-16	2.5746E-15
1514	NAP1L2	-3.7406018	4.1354E-06	1.0263E-05
1515	SLC5A6	-3.7396055	5.316E-176	8.488E-174
1516	ANGPTL4	-3.7383716	7.8282E-25	6.2444E-24
1517	SLC35C1	-3.7332556	2.729E-170	4.184E-168
1518	PCOLCE2	-3.7264122	4.5649E-15	2.2232E-14
1519	RFPL1	-3.7250436	7.3346E-07	1.9486E-06
1520	BOP1	-3.7241571	2.809E-54	5.8989E-53
1521	IL27	-3.7234965	2.4304E-10	8.614E-10
1522	NXF3	-3.723449	2.2803E-05	5.2761E-05
1523	PACRG	-3.7203033	2.7852E-06	7.0293E-06
1524	NGEF	-3.7197161	3.1275E-10	1.0994E-09
1525	PPIA	-3.7190863	6.644E-227	1.667E-224
1526	SIRPA	-3.7158821	2.501E-55	5.4108E-54

1527	MAFB	-3.7093234	1.1358E-59	2.806E-58
1528	FAM53A	-3.7023034	3.0577E-08	9.1604E-08
1529	RASSF9	-3.701827	0.0013688	0.0025735
1530	TLR2	-3.6983551	7.243E-14	3.2711E-13
1531	CCDC113	-3.6970624	4.6301E-15	2.2544E-14
1532	ST6GAL1	-3.6963104	9.032E-25	7.181E-24
1533	DKFZp779M0652	-3.695788	1.3503E-08	4.1598E-08
1534	TIAM1	-3.695473	1.0869E-05	2.5922E-05
1535	CCR7	-3.6949975	0.00048799	0.00097006
1536	MRPL24	-3.6933363	1.585E-139	1.595E-137
1537	APMAP	-3.692145	4.929E-293	2.381E-290
1538	CPED1	-3.691265	2.0832E-26	1.7753E-25
1539	HEYL	-3.6860038	2.4483E-11	9.3479E-11
1540	AGT	-3.6857763	1.1104E-23	8.3605E-23
1541	RPPH1	-3.6848116	2.6822E-06	6.7836E-06
1542	FAM115D	-3.6791814	0.00355414	0.00632225
1543	NMT2	-3.6765227	2.6585E-36	3.2757E-35
1544	STAT5A	-3.6752917	6.4788E-20	4.0679E-19
1545	KLRK1	-3.6700307	0.00038461	0.00077411
1546	ABHD14B	-3.6695454	1.294E-101	7.463E-100
1547	MAPK11	-3.6674331	3.3983E-15	1.6638E-14
1548	PM20D1	-3.6664012	0.00562601	0.00968671
1549	KLRC4-KLRK1	-3.6640299	0.00054523	0.00107818
1550	CRLF1	-3.6638987	0.00206781	0.00380115
1551	XIRP2	-3.6622675	0.01551645	0.02491908
1552	PNRC2	-3.6618871	2.667E-23	1.9742E-22
1553	TMEM139	-3.6598695	3.8858E-21	2.5986E-20
1554	HECW2	-3.6583288	6.8868E-22	4.7792E-21
1555	ACSL5	-3.6545071	0.00022941	0.0004744
1556	C15orf52	-3.6516231	3.9584E-12	1.5909E-11
1557	IGFBP4	-3.6511528	2.0013E-27	1.7827E-26
1558	TSKU	-3.6499469	2.648E-36	3.2648E-35
1559	ARSF	-3.6430239	0.00103389	0.00197497
1560	SLC4A4	-3.6410806	3.1301E-37	3.9785E-36
1561	FTCD	-3.6404005	1.4249E-43	2.1763E-42
1562	FAHD2A	-3.6389123	1.8522E-70	6.0244E-69
1563	KCNC3	-3.6368772	1.1424E-08	3.545E-08
1564	C16orf95	-3.6354824	1.2895E-18	7.6185E-18
1565	PILRA	-3.6343648	0.00028863	0.00059071
1566	MTSS1L	-3.6326951	2.282E-90	1.0886E-88
1567	SORL1	-3.6306515	4.8141E-41	6.8742E-40
1568	TMEM45B	-3.6264917	6.9761E-09	2.2005E-08
1569	AGAP2	-3.6213398	0.00014881	0.00031504
1570	RASGRP2	-3.6132398	1.1158E-24	8.8348E-24
1571	SHBG	-3.612543	1.4695E-25	1.2015E-24

1572	ALDH1A2	-3.6112548	0.00486453	0.0084669
1573	G0S2	-3.610391	3.9484E-21	2.6396E-20
1574	ADRB1	-3.6072801	0.00539797	0.00932399
1575	FGF23	-3.6032236	0.00075473	0.00146812
1576	C1orf21	-3.6031684	7.6016E-18	4.3106E-17
1577	ZAP70	-3.6010373	2.0879E-06	5.3358E-06
1578	KLF2	-3.6010296	1.7814E-12	7.3166E-12
1579	RPS27A	-3.6001463	3.192E-119	2.467E-117
1580	ADRA2A	-3.5997698	5.9858E-06	1.4639E-05
1581	CLDN14	-3.5994159	3.6492E-09	1.1784E-08
1582	FFAR2	-3.5981875	0.00528706	0.00914139
1583	MALL	-3.596881	0.0221236	0.03458156
1584	ACTA2-AS1	-3.5938653	2.3242E-26	1.9738E-25
1585	PRKG1	-3.5929006	7.0286E-09	2.2163E-08
1586	AZU1	-3.5924831	0.00038321	0.00077146
1587	SCARF1	-3.5919534	2.8045E-23	2.072E-22
1588	SEC14L2	-3.5860275	2.913E-79	1.1416E-77
1589	PCOLCE-AS1	-3.5841157	3.9949E-07	1.0884E-06
1590	SLC16A11	-3.5821475	3.59E-19	2.1824E-18
1591	NLRP3	-3.5820482	0.00012309	0.00026316
1592	VEGFC	-3.5809702	0.00111028	0.00211294
1593	CPB2-AS1	-3.5767125	5.0615E-07	1.3656E-06
1594	LMLN	-3.5742297	4.4013E-14	2.0164E-13
1595	TNFAIP2	-3.5737336	8.4312E-07	2.2305E-06
1596	DCN	-3.5719906	0.01133219	0.01859346
1597	LILRB3	-3.5714123	5.9869E-22	4.1637E-21
1598	SLC13A5	-3.5680527	2.6852E-25	2.1816E-24
1599	F5	-3.5640591	0.00023094	0.0004774
1600	ALDH1B1	-3.5636437	3.167E-111	2.124E-109
1601	JPH4	-3.5621343	0.0084152	0.01411422
1602	GCGR	-3.5621242	3.1345E-22	2.2053E-21
1603	PLA2G4C	-3.5582905	3.4287E-10	1.2009E-09
1604	RPS10	-3.5559005	1.7991E-62	4.8209E-61
1605	SOD2	-3.5521966	9.35E-41	1.3195E-39
1606	LOC101927905	-3.5499459	0.01170659	0.01917356
1607	GAS2	-3.549512	2.4731E-33	2.7382E-32
1608	GBP1	-3.5494067	7.9691E-32	8.3675E-31
1609	C9orf163	-3.5490761	2.6396E-13	1.1481E-12
1610	ARL11	-3.5490443	3.6321E-06	9.0685E-06
1611	RPS7	-3.5471604	3.5695E-83	1.4959E-81
1612	ACOT4	-3.5437373	1.1236E-11	4.3882E-11
1613	DHRS4L1	-3.5351346	4.8315E-05	0.00010805
1614	PKD1L3	-3.5263541	1.9761E-08	6.0019E-08
1615	WHAMMP1	-3.5226821	9.7946E-07	2.5791E-06
1616	CSRP1	-3.5205345	2.9504E-14	1.3643E-13

1617	BMP6	-3.5204814	1.3141E-54	2.7746E-53
1618	C1QL1	-3.5196454	0.00244768	0.00445829
1619	TMEM105	-3.5173505	5.6127E-06	1.3756E-05
1620	ANGPT2	-3.517196	1.7974E-15	8.9592E-15
1621	HIST2H2AC	-3.5163355	0.03107421	0.04731666
1622	GCK	-3.51615	0.01585042	0.02540697
1623	MYC	-3.5152906	1.1197E-11	4.3739E-11
1624	CNTN1	-3.5142272	8.4377E-07	2.2319E-06
1625	KLRD1	-3.5131409	0.0001967	0.00041022
1626	ARHGDIB	-3.5100736	4.21E-30	4.1309E-29
1627	PCDH12	-3.5096853	7.2541E-45	1.1535E-43
1628	CYB5A	-3.5042632	1.6377E-90	7.8318E-89
1629	TPPP	-3.5031345	1.0235E-10	3.7324E-10
1630	IQSEC3	-3.4996717	2.9138E-05	6.6717E-05
1631	LOC100507642	-3.4956644	5.5933E-09	1.7797E-08
1632	SEMA3F	-3.4902656	5.1499E-06	1.2673E-05
1633	PEX11A	-3.490205	2.6951E-35	3.2023E-34
1634	DHTKD1	-3.4896735	6.147E-159	7.971E-157
1635	CAMK4	-3.4882025	0.00307985	0.00553358
1636	CBR1	-3.4843261	4.4361E-37	5.6163E-36
1637	NPR3	-3.4812353	8.7589E-08	2.5212E-07
1638	ADAP2	-3.4776641	1.3504E-08	4.1598E-08
1639	EEF1A1	-3.4717436	4.2468E-96	2.2541E-94
1640	JAZF1-AS1	-3.4704757	0.02375918	0.03693494
1641	GYPC	-3.4704132	2.31E-13	1.0086E-12
1642	ISLR	-3.4696603	3.3624E-06	8.4223E-06
1643	FCHO1	-3.4685468	2.9904E-05	6.8379E-05
1644	NUP62CL	-3.4638297	0.00549543	0.00947877
1645	F7	-3.4620791	1.2772E-50	2.4239E-49
1646	KNG1	-3.4597342	5.8747E-28	5.3487E-27
1647	NCALD	-3.4548857	3.9484E-08	1.1716E-07
1648	NKX3-1	-3.4534804	6.7549E-12	2.6748E-11
1649	RASGEF1B	-3.4493108	1.3282E-33	1.479E-32
1650	SLC26A1	-3.4481596	3.0238E-57	6.9136E-56
1651	IRAK2	-3.4465166	2.9913E-36	3.681E-35
1652	RPL36A	-3.446189	3.0072E-23	2.2176E-22
1653	PGLYRP1	-3.4446119	0.006224	0.01065459
1654	ETFB	-3.4430899	4.588E-153	5.438E-151
1655	SIDT1	-3.4420568	0.0046318	0.00808587
1656	PI16	-3.4416645	2.1608E-05	5.0121E-05
1657	LOC728040	-3.4408967	3.4026E-05	7.7358E-05
1658	AKR1E2	-3.4388856	0.00321683	0.00575882
1659	IL1R1	-3.4359211	7.6264E-05	0.00016673
1660	HYAL1	-3.4322405	7.6521E-48	1.3295E-46
1661	MOCS1	-3.4265404	3.28E-115	2.347E-113

1662	IRF4	-3.4255999	0.0214751	0.03365501
1663	CISH	-3.4245108	5.7009E-56	1.2631E-54
1664	LOC101927100	-3.4244596	0.01652864	0.02639774
1665	AHRR	-3.4224599	3.1742E-33	3.5063E-32
1666	BMPER	-3.4202715	1.9884E-09	6.5599E-09
1667	HSPD1	-3.4178906	2.827E-182	4.833E-180
1668	MST1	-3.4156468	2.6977E-39	3.6524E-38
1669	MGST1	-3.4124424	1.8886E-62	5.0536E-61
1670	PDXP	-3.4121505	7.9877E-56	1.7537E-54
1671	DAPP1	-3.4108844	8.1043E-06	1.9574E-05
1672	ALOX5	-3.4102016	3.7289E-24	2.8782E-23
1673	PCCA	-3.4090264	6.3028E-44	9.7338E-43
1674	VKORC1	-3.4067697	1.253E-101	7.25E-100
1675	POM121L9P	-3.4066301	0.01607572	0.02573181
1676	DHRS4	-3.4053082	2.4122E-59	5.8768E-58
1677	METAP1D	-3.4039567	1.0489E-13	4.6802E-13
1678	ABHD17A	-3.4030004	1.6968E-45	2.7501E-44
1679	RPS2	-3.3995977	2.7195E-81	1.0946E-79
1680	VSTM4	-3.3986776	3.3981E-14	1.5654E-13
1681	HIST1H2BN	-3.3984254	0.02632061	0.04059346
1682	SCN1B	-3.397294	1.0212E-15	5.1646E-15
1683	SFRP5	-3.3939266	1.2668E-09	4.2432E-09
1684	MAOB	-3.3932641	3.7618E-31	3.8312E-30
1685	JAZF1	-3.3919074	1.1922E-32	1.2854E-31
1686	ACADL	-3.3907478	2.4588E-11	9.3862E-11
1687	MB	-3.389219	4.3955E-05	9.8779E-05
1688	NUDT7	-3.3888082	2.7361E-39	3.6993E-38
1689	FAM86B3P	-3.387534	2.745E-06	6.9361E-06
1690	BRINP1	-3.3844174	0.00570294	0.00980954
1691	ARSG	-3.3840019	2.6014E-12	1.0572E-11
1692	HTATSF1P2	-3.3839449	8.101E-24	6.1377E-23
1693	NEURL1B	-3.3835421	1.348E-84	5.8656E-83
1694	TRPV2	-3.3830214	4.5328E-11	1.6959E-10
1695	SERPINA2	-3.3826672	0.00977841	0.01622736
1696	LAT2	-3.3787104	8.4796E-18	4.8029E-17
1697	HIST1H2BK	-3.3765576	6.3126E-22	4.387E-21
1698	FTH1	-3.3747518	1.209E-82	5.0017E-81
1699	SREBF1	-3.3746618	4.1092E-24	3.1667E-23
1700	KCNJ15	-3.3740603	1.1225E-05	2.6737E-05
1701	LOC154761	-3.3711962	0.00193105	0.00356536
1702	BATF2	-3.3687904	8.0732E-16	4.1056E-15
1703	RTN4RL2	-3.3687553	1.3328E-08	4.1087E-08
1704	CES3	-3.3685142	2.1187E-16	1.109E-15
1705	TPSG1	-3.3669429	1.7073E-05	4.006E-05
1706	PGBD5	-3.3654541	1.1742E-11	4.5793E-11

1707	TMPRSS6	-3.3635501	7.2821E-31	7.3238E-30
1708	ICAM3	-3.3625441	2.8597E-61	7.3862E-60
1709	SIRPB1	-3.3620962	0.00015246	0.00032219
1710	GALNT2	-3.3610189	2.081E-144	2.186E-142
1711	FGF14-AS2	-3.3570502	0.00529525	0.00915336
1712	LDHA	-3.3545453	5.5726E-71	1.8341E-69
1713	TCP10L	-3.3545435	1.3337E-05	3.1607E-05
1714	CLEC10A	-3.3535722	1.2059E-06	3.1471E-06
1715	BTBD11	-3.3526445	0.00822008	0.01381574
1716	TMEM231	-3.3499862	8.4495E-13	3.5511E-12
1717	ACY1	-3.348827	9.56E-218	2.252E-215
1718	IRF8	-3.3487667	1.0789E-07	3.0839E-07
1719	RPL10	-3.3449434	3.9265E-88	1.8148E-86
1720	KCNE1	-3.3436394	5.2853E-07	1.4228E-06
1721	LDLRAD4	-3.3410127	1.9557E-20	1.2629E-19
1722	GVINP1	-3.3387365	2.7083E-11	1.0316E-10
1723	GRB14	-3.3363976	1.4758E-31	1.5346E-30
1724	SPRR3	-3.3361028	1.0057E-05	2.4073E-05
1725	LINC01370	-3.3360518	4.864E-05	0.00010871
1726	ABAT	-3.3343021	5.7684E-16	2.9569E-15
1727	FCGR1A	-3.3330801	0.00045615	0.00091003
1728	ZFP3	-3.3323086	3.4544E-06	8.6438E-06
1729	ACAA2	-3.3292249	3.2763E-90	1.5552E-88
1730	PEAR1	-3.3261685	4.0481E-07	1.1017E-06
1731	C1orf228	-3.3224119	2.6046E-07	7.2011E-07
1732	FGG	-3.3220997	3.4931E-47	5.9227E-46
1733	ANG	-3.3218432	7.0174E-44	1.082E-42
1734	KCNK13	-3.319201	0.00584587	0.01004019
1735	ITK	-3.3165038	3.3575E-05	7.6395E-05
1736	FAM229B	-3.3100127	1.3593E-16	7.193E-16
1737	FRK	-3.3054815	1.8893E-15	9.4005E-15
1738	VMO1	-3.3003449	3.5855E-14	1.6501E-13
1739	C2orf82	-3.2990155	4.4521E-06	1.1023E-05
1740	ABHD14A-ACY1	-3.2986792	6.874E-238	1.897E-235
1741	C9orf117	-3.2948799	3.5952E-09	1.1611E-08
1742	CLUH	-3.2940027	1.432E-154	1.729E-152
1743	SIGLEC9	-3.2922722	6.8896E-09	2.176E-08
1744	TRPM4	-3.2920352	4.3934E-35	5.1662E-34
1745	LINC00598	-3.2917384	0.0049724	0.00863528
1746	RBKS	-3.2916639	7.9422E-22	5.488E-21
1747	ALOX12P2	-3.2911941	1.1195E-12	4.6743E-12
1748	EXOC3L1	-3.2905427	6.2772E-15	3.0236E-14
1749	ACADS	-3.290183	6.498E-109	4.199E-107
1750	KLF11	-3.2861713	1.654E-83	6.962E-82
1751	FBXW10	-3.2852154	0.0252377	0.039023

1752	NUGGC	-3.284517	1.4332E-19	8.8808E-19
1753	PKLR	-3.283206	9.947E-42	1.4559E-40
1754	TEK	-3.2830796	5.035E-07	1.3588E-06
1755	SDHD	-3.282752	7.6475E-84	3.2688E-82
1756	RPGRIP1	-3.2786273	0.00613212	0.01050661
1757	C22orf15	-3.277924	8.3287E-19	4.9552E-18
1758	NFIA	-3.2727786	6.9453E-10	2.3723E-09
1759	RASSF1-AS1	-3.2719694	0.00014807	0.00031353
1760	DMBT1	-3.2715973	3.2545E-05	7.4165E-05
1761	AFAP1L1	-3.2667673	1.4065E-22	1.0046E-21
1762	COMT	-3.2636556	6.7648E-10	2.3136E-09
1763	LOC389641	-3.2601739	3.785E-09	1.2198E-08
1764	FNDC4	-3.2600251	1.1392E-67	3.4991E-66
1765	TIMD4	-3.2594132	3.4219E-12	1.3793E-11
1766	PER3	-3.258389	1.0427E-07	2.985E-07
1767	ACTN2	-3.2564996	0.00571486	0.00982829
1768	C10orf10	-3.2521248	2.8641E-57	6.5563E-56
1769	MRGPRF	-3.2511069	1.5356E-09	5.1126E-09
1770	CD82	-3.2498953	1.8563E-09	6.1378E-09
1771	ACOT2	-3.2487389	4.831E-25	3.8873E-24
1772	HLA-DPB1	-3.2473489	6.1084E-12	2.4263E-11
1773	C6orf123	-3.2456396	9.8935E-05	0.00021369
1774	PTX3	-3.2449761	3.04E-05	6.9473E-05
1775	ST3GAL1	-3.2430869	5.4807E-39	7.338E-38
1776	SLC51A	-3.2429603	4.4567E-26	3.7453E-25
1777	LOC101928303	-3.2419808	1.615E-10	5.7942E-10
1778	HHIPL1	-3.2416365	2.5651E-12	1.0433E-11
1779	ZMYND12	-3.241627	4.4709E-07	1.2127E-06
1780	SEMA6B	-3.2358877	4.099E-05	9.2406E-05
1781	SEMA3G	-3.2358375	0.00095962	0.00184312
1782	SGCD	-3.2319382	0.00018664	0.00039042
1783	MGC27382	-3.2293366	0.02775779	0.04262284
1784	TMEM205	-3.2236305	7.795E-138	7.568E-136
1785	JAM2	-3.2227105	4.2441E-05	9.5544E-05
1786	HMGCL	-3.2221614	2.196E-104	1.326E-102
1787	LRIG1	-3.2215909	7.862E-14	3.539E-13
1788	CD36	-3.2207132	0.02747344	0.04221976
1789	LAMC3	-3.2195311	7.1235E-05	0.00015629
1790	CMSS1	-3.2160867	1.9665E-16	1.0302E-15
1791	PLEKHF1	-3.2157065	3.8491E-20	2.451E-19
1792	RETSAT	-3.2155908	9.4573E-30	9.1863E-29
1793	RPP40	-3.2153692	5.6161E-16	2.8803E-15
1794	CHST13	-3.2145971	9.7389E-26	8.0408E-25
1795	CREB3L3	-3.2129521	3.9649E-33	4.3623E-32
1796	GPR85	-3.2104724	0.0213417	0.03346766

1797	PANX2	-3.2069819	1.5659E-30	1.5611E-29
1798	FLT3	-3.2051109	0.00628747	0.01075753
1799	IKBKG	-3.2049707	9.6294E-61	2.4543E-59
1800	F2	-3.2043176	1.7417E-26	1.4883E-25
1801	GLP2R	-3.2035556	0.00054296	0.00107403
1802	HSD17B10	-3.1984248	2.665E-117	1.979E-115
1803	AOC3	-3.1959836	3.9884E-23	2.9221E-22
1804	PYCARDOS	-3.1948809	6.9461E-10	2.3723E-09
1805	JAK3	-3.1943736	1.7341E-09	5.7435E-09
1806	WDR65	-3.1940718	1.5557E-11	6.0221E-11
1807	FAM43A	-3.1932661	2.4604E-22	1.7336E-21
1808	GAB3	-3.1914922	8.7279E-09	2.729E-08
1809	FENDRR	-3.1894616	2.323E-05	5.3697E-05
1810	C10orf54	-3.1892868	6.9324E-42	1.0177E-40
1811	SLC25A22	-3.1842743	2.5362E-19	1.5551E-18
1812	FAM115C	-3.1839452	3.2091E-12	1.2955E-11
1813	CARD9	-3.1838228	1.0451E-08	3.2519E-08
1814	ABI3BP	-3.1824911	6.2233E-07	1.6639E-06
1815	CYP7A1	-3.176055	0.00530447	0.00916821
1816	SH2D2A	-3.1746993	0.00647689	0.01106202
1817	NTHL1	-3.1746263	4.2669E-23	3.1214E-22
1818	SQRDL	-3.174158	2.1612E-13	9.4683E-13
1819	MDFIC	-3.1724015	8.5569E-24	6.4729E-23
1820	ACMSD	-3.1722523	6.9669E-25	5.5712E-24
1821	MAP3K5	-3.1717162	4.9469E-14	2.2563E-13
1822	HIST1H1C	-3.1683996	2.1365E-17	1.1817E-16
1823	CLYBL	-3.1677136	9.1064E-51	1.7334E-49
1824	GBP3	-3.1672452	3.8749E-19	2.3498E-18
1825	C3orf67	-3.163534	1.8331E-06	4.7108E-06
1826	CRYL1	-3.1633003	4.7652E-86	2.141E-84
1827	EXOSC5	-3.1628775	8.3983E-58	1.9549E-56
1828	C1orf111	-3.1623606	0.0031345	0.00562393
1829	MUC6	-3.1621826	0.00033386	0.0006781
1830	SCUBE1	-3.1607248	6.3507E-09	2.0134E-08
1831	ACTA2	-3.1597918	8.9514E-23	6.4555E-22
1832	LOC283335	-3.159238	1.1493E-26	9.9259E-26
1833	DUSP23	-3.1550744	2.6851E-35	3.1924E-34
1834	HPN-AS1	-3.1547777	5.303E-77	1.9779E-75
1835	OGDHL	-3.153407	2.9474E-33	3.2576E-32
1836	TMEM171	-3.1490558	0.00049597	0.00098551
1837	IPCEF1	-3.1473736	0.00422361	0.00742629
1838	HEY2	-3.1464221	1.8038E-08	5.4992E-08
1839	RNF144B	-3.1445363	1.1504E-31	1.2021E-30
1840	CECR5	-3.1426057	1.8625E-24	1.4545E-23
1841	PCTP	-3.1416533	1.571E-113	1.092E-111

1842	ENG	-3.1416026	9.5108E-05	0.00020588
1843	NDST3	-3.1409163	0.01934629	0.03058426
1844	BEND7	-3.1391514	4.4763E-11	1.6754E-10
1845	GGACT	-3.1385658	3.4476E-42	5.1002E-41
1846	DHRS3	-3.1377513	1.537E-198	3.194E-196
1847	CD300LF	-3.1362103	0.00224593	0.00410864
1848	TK2	-3.1281521	2.7389E-92	1.3296E-90
1849	SLC17A8	-3.1265196	0.02883082	0.04414419
1850	FAM86JP	-3.1262461	0.00101467	0.00193997
1851	LIPC	-3.1262029	7.1704E-15	3.4426E-14
1852	SLC41A2	-3.1243657	1.6444E-30	1.6385E-29
1853	OTUD7A	-3.1145115	0.00041088	0.00082441
1854	BAI1	-3.1115283	0.0212491	0.03333869
1855	ARPP21	-3.1112983	6.0474E-07	1.6187E-06
1856	GPR55	-3.1058443	0.02677031	0.04122777
1857	CD81	-3.1051177	4.114E-114	2.87E-112
1858	GBP2	-3.1038795	3.7988E-44	5.8998E-43
1859	AGPAT9	-3.1008802	1.509E-07	4.2648E-07
1860	ITGAL	-3.1002916	1.9566E-21	1.3254E-20
1861	DGCR6L	-3.0949306	1.6875E-97	9.2099E-96
1862	CECR5-AS1	-3.0911651	0.01463011	0.02358987
1863	LAP3	-3.09	1.011E-175	1.601E-173
1864	SDHA	-3.089039	4.0544E-89	1.8875E-87
1865	ECHS1	-3.0860772	2.82E-116	2.056E-114
1866	RRM2	-3.0860131	1.664E-07	4.6822E-07
1867	FAM86C1	-3.0854759	7.8488E-14	3.5339E-13
1868	CYP2U1	-3.0833841	4.4363E-11	1.6607E-10
1869	KLF4	-3.0822789	8.5331E-22	5.892E-21
1870	PYCARD	-3.0819876	6.9505E-16	3.5487E-15
1871	PDK2	-3.0812161	3.3024E-40	4.5901E-39
1872	ADAMTS13	-3.0792672	7.2682E-14	3.2817E-13
1873	PYCRL	-3.0787311	1.186E-37	1.5256E-36
1874	KANK2	-3.0781762	2.3391E-37	2.9868E-36
1875	CD34	-3.0773885	1.726E-07	4.8489E-07
1876	PTGR1	-3.0733175	5.4896E-18	3.1323E-17
1877	TMEM52	-3.0720313	7.8942E-08	2.2801E-07
1878	ABCC9	-3.0707612	6.1191E-08	1.7874E-07
1879	CPNE5	-3.06944	0.00900405	0.01502749
1880	GADD45G	-3.0686892	4.3418E-18	2.4921E-17
1881	AS3MT	-3.0665838	9.4375E-30	9.1716E-29
1882	LOC101929679	-3.063202	0.00017883	0.0003749
1883	LOC100130950	-3.0622823	0.00170471	0.00316957
1884	SMIM2-AS1	-3.0611699	0.00107995	0.00205786
1885	SRPX	-3.0610175	1.6452E-20	1.0663E-19
1886	ABTB1	-3.060429	1.4154E-27	1.2678E-26

1887	LCK	-3.0585494	0.00108362	0.00206424
1888	MX1	-3.0583167	1.0118E-71	3.4056E-70
1889	DEXI	-3.0574054	1.1938E-65	3.4894E-64
1890	CYB561	-3.056459	1.7811E-56	4.0106E-55
1891	LOC101928909	-3.0553115	0.00287141	0.00517786
1892	LRP5	-3.0538285	5.8407E-36	7.1194E-35
1893	RPS15	-3.0533674	3.4672E-69	1.0945E-67
1894	MAGIX	-3.0518231	1.1713E-22	8.4059E-22
1895	PRSS53	-3.0515033	3.5986E-13	1.5498E-12
1896	ZNF503	-3.045611	1.4136E-06	3.6673E-06
1897	SCO2	-3.0424779	3.432E-123	2.87E-121
1898	SCCPDH	-3.0413497	5.875E-141	6.038E-139
1899	KCNJ10	-3.0412756	0.00024851	0.00051192
1900	RPL13AP5	-3.0407389	6.4281E-77	2.3883E-75
1901	SAMD5	-3.0407339	2.0921E-05	4.8586E-05
1902	ONECUT2	-3.0328764	7.4779E-09	2.3515E-08
1903	PTAFR	-3.0305013	5.2611E-16	2.7004E-15
1904	PCOLCE	-3.0304934	1.5069E-08	4.6241E-08
1905	PMS2P5	-3.0261702	4.2059E-05	9.474E-05
1906	HSPE1	-3.0243069	1.4785E-92	7.2682E-91
1907	MOK	-3.0232544	1.7416E-05	4.0819E-05
1908	UGT2B7	-3.0210356	0.00487622	0.00848574
1909	NLRC4	-3.0200533	9.6637E-08	2.7725E-07
1910	RMRP	-3.0194229	0.00019337	0.00040366
1911	C8orf4	-3.0188468	1.1889E-10	4.3118E-10
1912	NT5C1B	-3.0140852	0.0009984	0.00191208
1913	CATIP	-3.0140232	0.02928757	0.04480803
1914	PLSCR4	-3.0120734	3.7736E-41	5.4165E-40
1915	VIT	-3.0095183	0.03052403	0.04654914
1916	SPRYD3	-3.0072654	9.8266E-32	1.0284E-30
1917	BAIAP2	-3.0070407	1.591E-71	5.3181E-70
1918	SSTR2	-3.0043166	0.00924299	0.01539834
1919	NOS3	-3.0042031	4.1354E-31	4.2072E-30
1920	CPA1	-3.0002204	0.00019835	0.00041351
1921	CIDEB	-2.9998636	3.4252E-54	7.1618E-53
1922	C2orf81	-2.9997347	0.00682139	0.01161242
1923	GSTO2	-2.9956329	1.4345E-08	4.4089E-08
1924	RASD1	-2.9944395	4.3897E-09	1.4078E-08
1925	XKR9	-2.9923659	0.00011908	0.00025503
1926	LRRC70	-2.9917657	0.00245167	0.00446472
1927	HFE	-2.9902065	8.7185E-20	5.4529E-19
1928	TPMT	-2.9851302	3.1949E-57	7.2876E-56
1929	GLUD1	-2.9842308	2.0714E-22	1.468E-21
1930	DDIT4	-2.9840637	8.4329E-16	4.2863E-15
1931	THEM6	-2.9839311	4.4485E-64	1.2331E-62

1932	RPS10-NUDT3	-2.9801698	1.9283E-92	9.4554E-91
1933	C1QTNF1	-2.9766722	4.8952E-07	1.3224E-06
1934	ACCS	-2.9759291	7.6186E-19	4.5454E-18
1935	SLC2A9	-2.9742588	1.8398E-15	9.1565E-15
1936	NSUN6	-2.9715041	2.3748E-67	7.2255E-66
1937	REPS2	-2.9696274	1.1601E-31	1.2116E-30
1938	CCDC28A	-2.9684011	2.7448E-21	1.8496E-20
1939	KIF17	-2.9676889	2.4626E-07	6.8221E-07
1940	FAM228A	-2.9659926	0.02629941	0.04056399
1941	TMEM121	-2.9659491	0.00062844	0.00123414
1942	GRAMD4	-2.9646048	3.678E-170	5.595E-168
1943	LOC645752	-2.9633666	0.01252587	0.02041676
1944	HSPA6	-2.9613612	0.00034956	0.00070747
1945	CHST7	-2.9605188	1.7219E-24	1.3469E-23
1946	CCDC13	-2.9588497	1.7389E-05	4.0762E-05
1947	HLX-AS1	-2.9580062	1.5692E-06	4.0575E-06
1948	FGGY	-2.9572222	7.1636E-08	2.0781E-07
1949	PDZRN4	-2.9564305	0.00272513	0.00492788
1950	HHEX	-2.9563243	3.5601E-23	2.6143E-22
1951	TCHH	-2.9520775	0.00221986	0.00406365
1952	IFIT3	-2.9508344	1.775E-31	1.8349E-30
1953	SCARNA2	-2.9506357	0.01931962	0.03054961
1954	CD44	-2.9489804	1.2277E-09	4.1173E-09
1955	IGFLR1	-2.9480016	1.9206E-20	1.2414E-19
1956	PPAPDC3	-2.9473267	0.01690946	0.02697025
1957	ALAS1	-2.9446192	6.0134E-25	4.8187E-24
1958	ACRBP	-2.9435461	0.00092968	0.00178927
1959	RAMP2	-2.9429117	4.023E-07	1.0953E-06
1960	SDR42E1	-2.941605	1.8664E-14	8.7269E-14
1961	LINC00939	-2.9407679	0.02160817	0.03384707
1962	BDH1	-2.9375714	6.3082E-50	1.1663E-48
1963	CRTAC1	-2.9371949	0.00617325	0.0105724
1964	QDPR	-2.9359954	2.478E-81	1.0016E-79
1965	SLC22A18AS	-2.9356296	4.8457E-34	5.4844E-33
1966	ETV1	-2.935516	0.00041158	0.00082564
1967	WDR31	-2.9352182	5.2092E-09	1.661E-08
1968	LOC101929089	-2.9349864	5.867E-30	5.7277E-29
1969	TXNIP	-2.9345217	2.7366E-08	8.2265E-08
1970	SLC2A10	-2.9342401	2.7676E-38	3.6424E-37
1971	HS3ST3A1	-2.927934	7.1598E-06	1.74E-05
1972	DAK	-2.92044	3.4978E-56	7.8305E-55
1973	RRN3P2	-2.9167467	0.00908322	0.01515438
1974	NOS1AP	-2.9155809	1.816E-14	8.5011E-14
1975	IPO4	-2.9138503	7.8068E-27	6.7818E-26
1976	SELO	-2.9134753	1.799E-157	2.271E-155

1977	ASB2	-2.9118321	0.00065743	0.00128845
1978	SERPINF2	-2.9097847	3.002E-15	1.4777E-14
1979	NT5M	-2.9097644	0.0010507	0.00200528
1980	CLDN11	-2.9091482	7.8773E-08	2.2756E-07
1981	ACOT1	-2.9078254	6.9075E-27	6.0195E-26
1982	LTBP4	-2.9065366	6.5907E-25	5.2725E-24
1983	TGFA	-2.9044095	6.3039E-14	2.8543E-13
1984	PROX1	-2.9039899	1.5667E-15	7.8373E-15
1985	CATIP-AS1	-2.9016616	0.01280163	0.02083986
1986	UQCRFS1	-2.8996948	5.2762E-34	5.9682E-33
1987	SDC1	-2.8954593	1.6769E-27	1.4985E-26
1988	PNKD	-2.8948369	8.2548E-33	8.9597E-32
1989	SULT1A1	-2.8940752	4.0412E-52	8.0242E-51
1990	ACADVL	-2.8898098	1.997E-37	2.5549E-36
1991	FAM86FP	-2.8889908	0.00018243	0.00038224
1992	LINC00959	-2.88722	0.00266194	0.00482399
1993	PALD1	-2.8871314	0.00285748	0.00515467
1994	IFITM10	-2.8861315	1.1406E-31	1.1931E-30
1995	BHMT	-2.8858122	3.3174E-11	1.2542E-10
1996	SYNE4	-2.8854688	2.4206E-07	6.7145E-07
1997	SRXN1	-2.8852527	9.8097E-92	4.7381E-90
1998	NUP62	-2.8846426	1.532E-161	2.069E-159
1999	LOC100133985	-2.8780488	0.00250942	0.00456277
2000	PQLC1	-2.8761739	3.9207E-52	7.793E-51
2001	GCDH	-2.8761622	5.955E-56	1.3179E-54
2002	CD5	-2.8733801	0.0048908	0.00850727
2003	MST1L	-2.8719392	9.8607E-18	5.5639E-17
2004	TNFAIP8L1	-2.8711689	7.6838E-28	6.963E-27
2005	CAMK1D	-2.8703105	5.8408E-06	1.43E-05
2006	TTC38	-2.8690884	1.505E-114	1.061E-112
2007	ADCK3	-2.8653617	3.5635E-49	6.4282E-48
2008	EIF5A	-2.8653577	1.84E-116	1.347E-114
2009	SLC17A9	-2.8647306	3.3876E-40	4.7017E-39
2010	KCTD14	-2.8637915	1.6504E-09	5.4853E-09
2011	TGFB3	-2.8627496	3.6728E-06	9.1642E-06
2012	PTMA	-2.8610999	1.6027E-44	2.5194E-43
2013	CLRN3	-2.8605692	1.0833E-06	2.8439E-06
2014	ITPRIP	-2.8601902	2.2754E-15	1.1272E-14
2015	ABCA8	-2.8592171	6.7207E-14	3.0394E-13
2016	IL16	-2.8558016	7.4526E-15	3.5755E-14
2017	UBXN8	-2.8557165	5.5573E-47	9.3526E-46
2018	CCNB3	-2.8502899	0.01415112	0.02287864
2019	LPAR6	-2.8489367	2.2851E-22	1.613E-21
2020	H6PD	-2.8486639	7.1255E-79	2.7588E-77
2021	RPL6	-2.8478543	6.8704E-83	2.8731E-81

2022	GOLGA8N	-2.8476569	0.00511916	0.00887412
2023	PEX11G	-2.8476462	1.2266E-18	7.256E-18
2024	LRRC29	-2.8468102	4.5676E-09	1.4625E-08
2025	RASGRP3	-2.8463391	1.4663E-06	3.7979E-06
2026	ENPP7	-2.8462201	9.0265E-09	2.8191E-08
2027	SH3TC1	-2.8454073	1.8573E-19	1.1471E-18
2028	GAPDH	-2.8449811	5.364E-158	6.909E-156
2029	SLC34A1	-2.8447146	0.01072885	0.01767255
2030	EHF	-2.8441193	0.00061724	0.00121374
2031	LOC285847	-2.8440014	0.00036475	0.0007362
2032	MCCC2	-2.8416232	4.0395E-84	1.742E-82
2033	DNAH5	-2.8410764	7.9119E-07	2.098E-06
2034	RHEBL1	-2.8409173	0.01157336	0.01897144
2035	ERLIN1	-2.8397476	9.752E-104	5.797E-102
2036	KCND3	-2.8388312	2.1413E-09	7.0406E-09
2037	ADAM15	-2.8380355	6.0053E-24	4.5877E-23
2038	GPR146	-2.837348	2.0106E-38	2.6551E-37
2039	PCBD1	-2.8372	1.3381E-87	6.1117E-86
2040	MYRIP	-2.8362383	2.0407E-15	1.0141E-14
2041	JHDM1D-AS1	-2.8352938	1.5033E-07	4.2499E-07
2042	RAB20	-2.8352358	3.9694E-21	2.6527E-20
2043	NAMPT	-2.8341977	1.628E-24	1.2755E-23
2044	C1QTNF2	-2.8337612	0.02693381	0.04147238
2045	CPEB3	-2.8326631	4.1848E-28	3.8427E-27
2046	C10orf32-ASMT	-2.8299882	2.0427E-50	3.8352E-49
2047	FTCDNL1	-2.8297893	0.00063976	0.00125509
2048	FAM86A	-2.8289566	4.0098E-51	7.7238E-50
2049	GSDMD	-2.8275297	6.4102E-48	1.1177E-46
2050	FAM124A	-2.8271747	0.00076014	0.00147805
2051	SPTBN4	-2.826738	0.00016765	0.00035256
2052	MRM1	-2.8251197	3.4057E-14	1.5685E-13
2053	SYBU	-2.8210579	1.1747E-09	3.9457E-09
2054	BIRC3	-2.8180567	3.2369E-15	1.5897E-14
2055	MSRA	-2.8179064	6.7929E-36	8.2644E-35
2056	HHIP	-2.8169618	5.1976E-05	0.00011578
2057	HRH1	-2.8132047	0.0176795	0.02811953
2058	ACTR3C	-2.8129595	5.7671E-23	4.1919E-22
2059	EIF5AL1	-2.8120534	9.6203E-11	3.5128E-10
2060	CCRL2	-2.8104316	0.00656852	0.01120465
2061	CACNA1H	-2.8103355	1.8262E-14	8.5472E-14
2062	RPL26L1	-2.8086795	3.8575E-22	2.7052E-21
2063	VAV3	-2.8068212	3.1992E-10	1.1234E-09
2064	LINC01184	-2.8065035	1.1618E-13	5.167E-13
2065	FGF7	-2.8047687	0.02562894	0.03958043
2066	LINC00671	-2.8012716	1.6639E-07	4.6822E-07

2067	LOC100507472	-2.8002054	1.4596E-10	5.2532E-10
2068	ECH1	-2.8001449	4.951E-101	2.813E-99
2069	SRPR	-2.7984884	2.094E-127	1.839E-125
2070	FGB	-2.7969146	8.5332E-41	1.2069E-39
2071	FOXP3	-2.7953738	0.00013583	0.00028915
2072	DDR2	-2.7942404	0.00194255	0.00358521
2073	FAM135B	-2.7929881	0.02718135	0.04181746
2074	LOC101928525	-2.7900836	1.3246E-48	2.35E-47
2075	PKD1L2	-2.7859402	0.00033071	0.0006722
2076	HSPA12B	-2.785321	1.6661E-06	4.2959E-06
2077	FNDC5	-2.7817494	0.00031482	0.00064153
2078	S100P	-2.7801086	4.4547E-05	0.00010005
2079	UBE2L6	-2.7790944	2.8021E-27	2.4845E-26
2080	TNFSF10	-2.772177	4.5135E-09	1.4454E-08
2081	LOC101929574	-2.7698997	0.02281601	0.03558613
2082	LINC01119	-2.7696808	0.00065988	0.00129272
2083	PREX1	-2.7690741	3.8628E-24	2.9804E-23
2084	GSTT2	-2.7687309	2.4893E-06	6.3131E-06
2085	GSTT2B	-2.7687309	2.4893E-06	6.3131E-06
2086	NPIPB3	-2.7687193	9.6425E-08	2.7673E-07
2087	AOC4P	-2.7682633	2.603E-08	7.837E-08
2088	UNC13D	-2.7673584	3.9433E-26	3.3239E-25
2089	PGAM1	-2.7661438	5.5842E-25	4.4841E-24
2090	SLC27A4	-2.7643695	8.2318E-90	3.898E-88
2091	LOC100288162	-2.7627399	8.2548E-13	3.4723E-12
2092	CDKN2C	-2.7622816	1.4586E-10	5.2505E-10
2093	P2RX5	-2.7618609	0.01628291	0.02603972
2094	CFAP69	-2.7599643	6.8162E-07	1.8149E-06
2095	OLFM2	-2.7587358	2.3937E-12	9.7525E-12
2096	TMX4	-2.7560311	6.2124E-59	1.4965E-57
2097	KLRG1	-2.7557476	0.0010021	0.00191821
2098	DIRAS2	-2.7555012	0.00267512	0.00484562
2099	C1orf54	-2.7523229	1.3384E-07	3.8011E-07
2100	NKD1	-2.7517911	0.00218774	0.00400827
2101	RBP5	-2.750175	6.3098E-11	2.334E-10
2102	TMOD1	-2.7459638	8.0331E-47	1.3472E-45
2103	MCAT	-2.744738	4.6894E-43	7.0837E-42
2104	CD302	-2.7434079	3.3246E-11	1.2565E-10
2105	TBC1D10C	-2.7427214	7.4354E-09	2.3396E-08
2106	PARP3	-2.7419635	2.0486E-14	9.5555E-14
2107	MECR	-2.7391147	4.1996E-49	7.5616E-48
2108	SLC22A9	-2.7357705	0.00013333	0.00028417
2109	MAP3K14	-2.7356873	1.5974E-40	2.2396E-39
2110	ABCC6	-2.7335499	4.3441E-62	1.1529E-60
2111	CCDC85B	-2.7280397	1.9772E-10	7.0517E-10

2112	ALDH9A1	-2.7262768	4.1661E-40	5.7615E-39
2113	CABLES1	-2.7258116	1.3716E-22	9.7998E-22
2114	MRPL46	-2.7229529	4.3173E-42	6.372E-41
2115	PKD1P6	-2.7229098	5.5099E-09	1.7543E-08
2116	HIST1H2BD	-2.7228871	2.6061E-13	1.1345E-12
2117	ADAMTS8	-2.7200216	0.02009876	0.03167793
2118	RYR1	-2.7191762	0.00676806	0.01152771
2119	ZDHHC19	-2.718083	0.00456714	0.00798321
2120	SLC16A13	-2.7164068	1.1275E-66	3.393E-65
2121	NAPSA	-2.7103359	0.02249767	0.03514635
2122	PARP15	-2.7091925	0.00655316	0.01118141
2123	OXSM	-2.7088984	1.7322E-47	2.9694E-46
2124	BCKDHB	-2.7048265	7.8931E-66	2.3317E-64
2125	FGFRL1	-2.7043589	1.2151E-28	1.1357E-27
2126	SMARCA2	-2.7036085	2.2057E-16	1.1526E-15
2127	EPDR1	-2.7008421	4.2087E-23	3.0812E-22
2128	CCNT2-AS1	-2.7002568	3.7712E-17	2.0588E-16
2129	MET	-2.6983655	8.625E-21	5.6698E-20
2130	SLCO4A1-AS1	-2.6974669	0.0084266	0.01412966
2131	MRPL36	-2.697423	1.1477E-42	1.7176E-41
2132	LINC01314	-2.6951741	2.5959E-11	9.898E-11
2133	RILP	-2.6935871	1.3094E-17	7.326E-17
2134	AGBL4	-2.6929489	0.00863499	0.01446152
2135	TRPM2	-2.6897968	0.00013838	0.00029418
2136	LINGO1	-2.6891348	0.02438142	0.03782311
2137	LHFP	-2.6875398	1.0106E-09	3.4121E-09
2138	LSMEM2	-2.6859172	2.1512E-18	1.2571E-17
2139	MLYCD	-2.6858909	2.1519E-64	6.0253E-63
2140	FH	-2.6808832	4.9339E-98	2.708E-96
2141	RDH5	-2.6800322	1.2963E-45	2.1081E-44
2142	CAMK1	-2.6793502	8.1209E-35	9.4744E-34
2143	RNF180	-2.6793377	4.7631E-11	1.7779E-10
2144	AGXT2	-2.6782773	2.0909E-22	1.4814E-21
2145	INSIG1	-2.676072	1.1441E-21	7.8635E-21
2146	TUBA8	-2.6759833	8.6612E-15	4.1327E-14
2147	SCP2	-2.6740446	1.3522E-45	2.1972E-44
2148	ASGR2	-2.6734305	1.3501E-27	1.211E-26
2149	DDX11L2	-2.6724122	0.00728569	0.01235167
2150	BLVRB	-2.6722301	7.8167E-64	2.1543E-62
2151	PROZ	-2.6717749	1.6449E-41	2.3876E-40
2152	AIDA	-2.6716333	2.2093E-22	1.5623E-21
2153	TLE6	-2.6698316	1.8463E-07	5.1765E-07
2154	EFNA1	-2.6658671	1.5534E-15	7.773E-15
2155	LINC00467	-2.6639718	3.9718E-08	1.178E-07
2156	SERPINB9P1	-2.6636996	0.00440565	0.00772317

2157	CFI	-2.6623678	2.6023E-08	7.836E-08
2158	HAAO	-2.6610452	5.1615E-37	6.5219E-36
2159	GOT2	-2.6609968	3.6618E-40	5.075E-39
2160	PHB	-2.6597247	9.266E-108	5.869E-106
2161	POU2F2	-2.659434	0.00042797	0.00085735
2162	YBX1	-2.6589147	1.8131E-77	6.8018E-76
2163	ENKD1	-2.658831	1.3689E-14	6.4631E-14
2164	CLPB	-2.6562527	5.6917E-38	7.4049E-37
2165	AGAP2-AS1	-2.6543362	0.000591	0.00116427
2166	SLC17A1	-2.6517999	9.6119E-10	3.2494E-09
2167	ROM1	-2.6517189	7.3149E-08	2.1201E-07
2168	AHSG	-2.6515507	4.7672E-08	1.4055E-07
2169	CRACR2A	-2.6501747	0.00073851	0.00143831
2170	C12orf66	-2.6489142	5.4594E-13	2.3217E-12
2171	ACBD4	-2.6481721	5.8776E-54	1.2223E-52
2172	CD177	-2.6468398	0.02145872	0.0336348
2173	C9orf139	-2.6459568	0.02352135	0.03659761
2174	RIPPLY1	-2.6450906	0.0012732	0.00240498
2175	NPW	-2.642984	1.4008E-08	4.3087E-08
2176	DBH-AS1	-2.6396885	9.6991E-15	4.612E-14
2177	DUSP1	-2.6386246	3.6964E-34	4.2058E-33
2178	METTL1	-2.6385616	9.7011E-13	4.063E-12
2179	HSD17B2	-2.6375479	6.5653E-21	4.3439E-20
2180	ZNF584	-2.6353814	3.3489E-22	2.3545E-21
2181	CHPF	-2.6353759	2.3722E-49	4.3196E-48
2182	KLHL32	-2.6348282	0.00467188	0.00815143
2183	C16orf46	-2.6337806	3.1557E-05	7.1998E-05
2184	CATSPER2P1	-2.6305778	0.00042494	0.00085155
2185	UBB	-2.6280375	6.477E-189	1.192E-186
2186	MAP4K1	-2.6279578	0.00163444	0.00304565
2187	LOC399491	-2.624428	3.0449E-12	1.2312E-11
2188	PLEKHO2	-2.6234477	6.4362E-34	7.2379E-33
2189	GPHN	-2.6221138	4.5317E-86	2.0409E-84
2190	MYLK-AS1	-2.6219177	1.2132E-17	6.7979E-17
2191	JAKMIP3	-2.6203825	0.00440804	0.00772597
2192	PNPLA2	-2.620054	1.8052E-68	5.6434E-67
2193	BLOC1S5-TXNDC5	-2.6198071	1.0105E-80	4.0422E-79
2194	LOC100507316	-2.6188704	0.00950522	0.0158025
2195	HPS5	-2.6186838	7.5436E-84	3.2315E-82
2196	PRPS1	-2.6180244	4.3359E-82	1.7861E-80
2197	PCCB	-2.6168751	6.3936E-85	2.7947E-83
2198	ALG1L9P	-2.6160309	4.3946E-05	9.877E-05
2199	PKD1P1	-2.6152207	2.8736E-12	1.1646E-11
2200	TRAP1	-2.6142854	1.3471E-40	1.8942E-39

2201	SERPINA3	-2.6123117	0.00014283	0.000303
2202	C14orf79	-2.6117315	5.777E-15	2.791E-14
2203	VWA1	-2.6112522	1.2561E-34	1.4593E-33
2204	CASC10	-2.6074553	1.0327E-05	2.4685E-05
2205	CFAP46	-2.605617	0.00063854	0.00125296
2206	FOLH1	-2.604488	0.00036094	0.00072882
2207	KLHL25	-2.5990619	1.2318E-48	2.1894E-47
2208	CCDC158	-2.5989975	3.0483E-05	6.9646E-05
2209	BACH2	-2.5965417	2.8554E-18	1.6576E-17
2210	SLC16A2	-2.5951325	1.1509E-20	7.5223E-20
2211	ESAM	-2.5936635	2.0831E-20	1.3437E-19
2212	LRRC2	-2.5928173	1.4049E-14	6.6266E-14
2213	GNE	-2.5928059	4.8615E-11	1.8125E-10
2214	NFS1	-2.5927925	1.1725E-29	1.1343E-28
2215	HOXB-AS3	-2.5907728	0.02790724	0.04281829
2216	LOC100505812	-2.5900911	0.001696	0.0031552
2217	SPDYE3	-2.5871639	8.586E-05	0.00018685
2218	A2M-AS1	-2.5869896	1.7306E-07	4.8604E-07
2219	C5orf63	-2.5864569	1.1671E-05	2.775E-05
2220	SRCIN1	-2.585036	9.052E-05	0.00019646
2221	BOLA2	-2.5850319	0.02517078	0.03893198
2222	BOLA2B	-2.5850319	0.02517078	0.03893198
2223	SPNS2	-2.5834007	3.2711E-15	1.6056E-14
2224	TSPAN8	-2.5831037	2.7441E-05	6.297E-05
2225	TXNDC5	-2.5817126	5.052E-75	1.7909E-73
2226	ZBED5-AS1	-2.5753723	1.6603E-13	7.3354E-13
2227	ISOC2	-2.5735492	3.07E-37	3.9072E-36
2228	TCAP	-2.5733266	0.00272429	0.00492682
2229	ACADSB	-2.5731042	4.0885E-10	1.4232E-09
2230	ST20-MTHFS	-2.5705316	2.4109E-37	3.0766E-36
2231	CLEC4A	-2.5678634	0.00043786	0.00087581
2232	GUCY1A2	-2.5630187	1.0913E-06	2.8634E-06
2233	PXMP4	-2.5606604	2.2263E-14	1.0357E-13
2234	PSD4	-2.5584481	3.1535E-43	4.7822E-42
2235	TPI1	-2.5562438	1.998E-101	1.145E-99
2236	APOBEC3G	-2.5559468	6.8883E-05	0.00015133
2237	SMAP2	-2.5552069	1.5312E-24	1.2025E-23
2238	LINC01503	-2.5548262	0.00015065	0.00031858
2239	COQ4	-2.5538207	3.7757E-80	1.4948E-78
2240	PLCB2	-2.5532358	4.1445E-15	2.0215E-14
2241	HSD17B4	-2.5500424	4.608E-109	2.998E-107
2242	RPL13A	-2.5497325	5.0512E-76	1.8378E-74
2243	RPP25	-2.5496295	3.6815E-08	1.0958E-07
2244	GPNMB	-2.5492693	0.00016903	0.00035538
2245	CXCR4	-2.5492183	4.0805E-06	1.0138E-05

2246	ALDH1A3	-2.5489396	3.3121E-09	1.0742E-08
2247	APOLD1	-2.5470609	1.4493E-11	5.6171E-11
2248	RPL23A	-2.5456339	6.9835E-58	1.6275E-56
2249	MYH14	-2.5455352	3.8166E-25	3.0826E-24
2250	FECH	-2.5444119	1.0808E-46	1.8094E-45
2251	DLST	-2.5442646	2.8816E-70	9.3099E-69
2252	KCNN3	-2.5437317	0.00974044	0.01617128
2253	LOC100126784	-2.5431996	0.00120726	0.00228601
2254	LRRC20	-2.5409152	1.6536E-21	1.1241E-20
2255	FAM86C2P	-2.5403691	1.3679E-05	3.2364E-05
2256	MAN1C1	-2.5346733	1.4188E-08	4.3627E-08
2257	EEF1B2	-2.5329565	1.162E-100	6.562E-99
2258	ZC2HC1C	-2.5319881	1.2406E-08	3.833E-08
2259	STARD9	-2.5317854	1.8399E-06	4.727E-06
2260	MON1A	-2.5314429	3.1526E-32	3.3484E-31
2261	PSMG4	-2.5296725	1.2883E-26	1.1107E-25
2262	COPZ2	-2.529183	3.0439E-12	1.2311E-11
2263	EGR1	-2.5284565	7.7793E-09	2.4411E-08
2264	LINC00319	-2.5279541	3.5664E-06	8.9172E-06
2265	IFRD2	-2.5269426	3.3438E-28	3.0822E-27
2266	ATP2A3	-2.5252003	3.6653E-05	8.3066E-05
2267	NDUFS2	-2.5237298	1.9769E-55	4.2914E-54
2268	FCRLB	-2.5230275	0.00506241	0.0087868
2269	GPX1	-2.5226088	1.5132E-69	4.8084E-68
2270	MRPS2	-2.52169	2.0468E-43	3.1161E-42
2271	RFPL1S	-2.5201011	4.821E-06	1.1897E-05
2272	C21orf33	-2.5199578	4.7381E-53	9.6358E-52
2273	TIMM23B	-2.5169932	4.8545E-08	1.4304E-07
2274	SYNM	-2.5157861	5.1323E-29	4.863E-28
2275	CTH	-2.5124343	6.3E-50	1.1659E-48
2276	MPST	-2.5123608	5.545E-182	9.397E-180
2277	GNG2	-2.5122395	0.0046201	0.00806691
2278	PCDHB15	-2.5120492	0.00058573	0.00115484
2279	SIGMAR1	-2.5087729	4.2584E-49	7.6603E-48
2280	POLD2	-2.5027568	1.5374E-32	1.6511E-31
2281	DUSP10	-2.502617	7.2596E-26	6.0299E-25
2282	HLX	-2.5022185	1.1089E-05	2.6423E-05
2283	RCBTB2	-2.497971	6.9167E-10	2.3631E-09
2284	SLC47A1	-2.4974598	6.3396E-07	1.6929E-06
2285	ITPKB	-2.4957565	1.7514E-12	7.2041E-12
2286	NEGR1	-2.4956335	0.00047427	0.00094413
2287	PCSK6	-2.4940663	1.8621E-10	6.6547E-10
2288	NDUFAF2	-2.4938848	4.471E-13	1.9106E-12
2289	APOL2	-2.4893314	7.7994E-22	5.3931E-21
2290	LY75-CD302	-2.4887289	2.7708E-10	9.7757E-10

2291	LOC283038	-2.4886418	0.01220575	0.01994039
2292	CD244	-2.4886314	5.9817E-06	1.463E-05
2293	XPNPEP2	-2.4882788	4.7369E-05	0.00010607
2294	SUN2	-2.487057	5.821E-120	4.609E-118
2295	CTSZ	-2.4858706	8.774E-112	5.948E-110
2296	GOLT1A	-2.4851905	7.5235E-56	1.6536E-54
2297	NME1	-2.484782	5.2545E-76	1.9082E-74
2298	SVEP1	-2.4838839	4.8032E-05	0.00010746
2299	ISM1	-2.4837584	0.02074304	0.03260296
2300	MEF2C	-2.483612	8.3972E-14	3.7738E-13
2301	ZNF214	-2.4808068	5.0173E-06	1.2359E-05
2302	HS6ST1	-2.4808059	1.8611E-44	2.921E-43
2303	SERPINB8	-2.4799762	2.1882E-12	8.9416E-12
2304	TSFM	-2.4799625	9.9086E-52	1.9534E-50
2305	TUSC8	-2.4798773	0.00014711	0.00031181
2306	VARS2	-2.4774981	1.2755E-12	5.3051E-12
2307	NEIL2	-2.4773862	3.6377E-29	3.4587E-28
2308	PUS1	-2.4767214	4.5219E-40	6.2492E-39
2309	SLC25A10	-2.4740357	9.5255E-97	5.0979E-95
2310	RPL29	-2.473767	1.3174E-81	5.3698E-80
2311	DPP9-AS1	-2.4726693	7.639E-40	1.0489E-38
2312	ACAD8	-2.4721321	1.6852E-58	4.0047E-57
2313	MORN2	-2.4715757	6.6048E-09	2.0895E-08
2314	SLC26A5	-2.4702958	0.03147878	0.0478761
2315	CSAD	-2.4700261	1.1467E-19	7.1373E-19
2316	TRIM14	-2.4696903	6.2555E-72	2.1129E-70
2317	DYNLL1-AS1	-2.468835	3.6605E-10	1.2793E-09
2318	SPOCK2	-2.4688209	2.3077E-12	9.412E-12
2319	MTUS2	-2.4672088	0.00454678	0.00795183
2320	COL16A1	-2.466272	0.00433277	0.00760439
2321	FRY	-2.4649773	5.403E-30	5.2854E-29
2322	LINC00847	-2.4645067	1.6699E-10	5.9822E-10
2323	DECR1	-2.464246	7.6073E-71	2.4953E-69
2324	TNS1	-2.4602159	1.6199E-07	4.5634E-07
2325	TMOD2	-2.459606	2.3592E-06	6.0004E-06
2326	PARD6A	-2.4582201	9.7598E-06	2.3403E-05
2327	NAIP	-2.4549178	0.00011113	0.0002389
2328	DNAJA3	-2.4541792	6.8479E-72	2.3089E-70
2329	NRG3	-2.4520393	0.01059041	0.01746089
2330	ITGB3	-2.4498675	1.5852E-11	6.1327E-11
2331	CCDC88B	-2.4485789	1.6851E-05	3.9548E-05
2332	PTPLAD2	-2.4483302	0.00550535	0.0094925
2333	LINC00963	-2.4429031	1.8833E-28	1.751E-27
2334	NDRG2	-2.4422016	2.7926E-12	1.1335E-11
2335	CYP7B1	-2.4416376	1.7073E-15	8.5232E-15

2336	MNS1	-2.4411544	0.03025905	0.04618096
2337	N4BP2L1	-2.4402349	7.0671E-09	2.2281E-08
2338	PRSS36	-2.4367702	0.03203848	0.04863164
2339	LRRC3	-2.4364158	3.4046E-20	2.1759E-19
2340	C1RL	-2.4363502	1.1022E-65	3.2313E-64
2341	METTL7A	-2.4353504	2.3611E-07	6.5569E-07
2342	TUBB	-2.4347967	4.7756E-20	3.0201E-19
2343	PLP2	-2.4328979	4.5143E-14	2.0662E-13
2344	RPL39	-2.4317514	3.458E-37	4.3866E-36
2345	TCTN1	-2.4284722	3.2868E-07	9.02E-07
2346	CGNL1	-2.4276787	2.2728E-94	1.1525E-92
2347	F2RL2	-2.4274478	2.9227E-05	6.6912E-05
2348	NQO2	-2.4258602	3.4149E-42	5.0556E-41
2349	DSEL	-2.4256398	2.8294E-12	1.1477E-11
2350	HMGN2	-2.4250157	9.9736E-23	7.1792E-22
2351	CD163L1	-2.4240055	0.0004669	0.00093023
2352	NAPRT	-2.4227129	5.1677E-46	8.4826E-45
2353	TMCO6	-2.4222245	1.1356E-25	9.3561E-25
2354	ID1	-2.4215522	1.7342E-07	4.87E-07
2355	KLF15	-2.4207688	1.7505E-37	2.2441E-36
2356	PPFIA3	-2.4204498	8.0013E-11	2.9422E-10
2357	DYSF	-2.4197095	5.8442E-22	4.0659E-21
2358	GPR183	-2.4192003	0.00016195	0.00034113
2359	SLC27A1	-2.417647	1.6428E-10	5.8895E-10
2360	DPM3	-2.4166544	3.2302E-30	3.1824E-29
2361	EFHD1	-2.4155915	3.2911E-08	9.8365E-08
2362	SLC25A42	-2.4151262	7.9336E-08	2.2908E-07
2363	FAM207A	-2.4149621	3.9101E-13	1.678E-12
2364	TSEN2	-2.412497	2.3756E-11	9.0832E-11
2365	NRP1	-2.4121713	7.445E-37	9.3644E-36
2366	RPL7A	-2.4106615	4.5386E-45	7.2648E-44
2367	PALM3	-2.4101785	7.9594E-08	2.2979E-07
2368	CAT	-2.4097156	2.5906E-28	2.3993E-27
2369	ALDH1A1	-2.4069049	3.1515E-10	1.1072E-09
2370	1-Sep	-2.4067337	7.0186E-07	1.8667E-06
2371	MFSD1	-2.4061831	3.4165E-34	3.8919E-33
2372	TANGO6	-2.4060638	2.3299E-20	1.4995E-19
2373	HPN	-2.4048375	3.3365E-94	1.6875E-92
2374	AIG1	-2.4039528	2.8887E-26	2.4446E-25
2375	IGFBP3	-2.4029155	1.4367E-07	4.0689E-07
2376	DPH2	-2.4019441	2.9146E-46	4.8418E-45
2377	SH2B3	-2.3978632	2.1051E-19	1.2977E-18
2378	ABCB8	-2.3978268	1.3319E-25	1.0931E-24
2379	UBAC2-AS1	-2.395052	0.00198057	0.00364912
2380	DNAJA4	-2.3921526	0.00296929	0.00534339

2381	ZNF747	-2.3913276	3.1173E-18	1.8064E-17
2382	RPL12	-2.3912596	3.6784E-51	7.0995E-50
2383	THSD1	-2.3899959	3.1263E-11	1.185E-10
2384	FICD	-2.3895182	1.0541E-13	4.7001E-13
2385	ACSF3	-2.3894446	4.7443E-70	1.5201E-68
2386	KRT222	-2.3878822	4.7993E-06	1.1849E-05
2387	SHROOM1	-2.387477	2.633E-28	2.4374E-27
2388	MTG2	-2.385756	1.2136E-23	9.1094E-23
2389	IMMP2L	-2.3847049	1.4156E-09	4.725E-09
2390	SORBS1	-2.3839298	4.6094E-60	1.1596E-58
2391	SEPHS1	-2.3793445	4.7263E-61	1.2143E-59
2392	ATG16L2	-2.3786669	7.9788E-05	0.00017422
2393	COLCA1	-2.3769818	0.00538851	0.00930932
2394	PRKCQ-AS1	-2.3764181	0.00737567	0.01249325
2395	HOXB2	-2.376171	0.02558438	0.03951793
2396	TUBE1	-2.3759071	2.6933E-12	1.0936E-11
2397	PTCD2	-2.3736452	4.3529E-09	1.3965E-08
2398	PLS1	-2.3735822	7.0295E-23	5.0936E-22
2399	LYRM1	-2.3718863	6.5852E-24	5.0188E-23
2400	NOP9	-2.3697026	1.4658E-74	5.1488E-73
2401	ALKBH2	-2.3695171	2.0735E-09	6.8304E-09
2402	PLVAP	-2.3677196	0.00044864	0.00089608
2403	HSPA9	-2.3645828	5.1866E-68	1.6007E-66
2404	CD6	-2.3633653	0.00062207	0.00122263
2405	DIEXF	-2.363089	1.7108E-21	1.1622E-20
2406	RNF5P1	-2.3620191	0.00091047	0.0017543
2407	GALNT14	-2.3614185	1.1022E-06	2.8896E-06
2408	DGCR5	-2.3604427	1.3501E-10	4.8774E-10
2409	ANKRD22	-2.3601611	0.02734797	0.04205036
2410	ENHO	-2.3581026	0.00029039	0.00059413
2411	ALB	-2.3580572	0.0013511	0.00254194
2412	ABHD2	-2.3566518	1.3756E-21	9.4043E-21
2413	KIAA0226L	-2.3533429	8.9546E-05	0.00019445
2414	IMP4	-2.3513249	2.5482E-66	7.5974E-65
2415	SDC3	-2.350126	1.8488E-26	1.5783E-25
2416	FAM185A	-2.3490205	1.9048E-13	8.3772E-13
2417	ZNF25	-2.3484316	1.6809E-06	4.333E-06
2418	NRAP	-2.3484223	2.4047E-22	1.6956E-21
2419	MTHFS	-2.3484197	1.6711E-31	1.7311E-30
2420	WASH3P	-2.3480299	1.0333E-07	2.9599E-07
2421	MYO7A	-2.3477261	4.5859E-11	1.7151E-10
2422	FAM50B	-2.3462519	2.5936E-32	2.7624E-31
2423	HADHA	-2.345649	7.7852E-47	1.3068E-45
2424	TRIB3	-2.3456141	8.8972E-17	4.7537E-16
2425	IDNK	-2.3454713	1.2524E-17	7.0112E-17

2426	RNMTL1	-2.3454198	2.1544E-43	3.2774E-42
2427	UQCRQ	-2.3449743	4.0071E-29	3.8043E-28
2428	CHADL	-2.3438091	6.0587E-07	1.6215E-06
2429	STOX2	-2.3437383	1.7468E-10	6.2497E-10
2430	FAM210B	-2.3408096	3.3065E-51	6.4073E-50
2431	ATRNL1	-2.3399316	6.5566E-05	0.00014441
2432	FUOM	-2.3389747	1.186E-38	1.5759E-37
2433	ZCWPW1	-2.337782	8.9696E-08	2.5795E-07
2434	TIMM8A	-2.3357986	7.0819E-26	5.8874E-25
2435	LINC00116	-2.3355459	4.3389E-21	2.8936E-20
2436	ZNF503-AS2	-2.3352649	0.00250308	0.00455277
2437	LPXN	-2.3340841	1.7614E-10	6.3008E-10
2438	HIBADH	-2.3339301	7.3445E-40	1.0092E-38
2439	ZNF485	-2.3330527	0.00939	0.01562709
2440	ITGA4	-2.3327835	1.3159E-07	3.7408E-07
2441	ISG20	-2.3325973	4.9644E-18	2.8419E-17
2442	CSF1	-2.3319184	6.2454E-14	2.8297E-13
2443	CTSO	-2.3307725	3.8605E-41	5.5329E-40
2444	METTL20	-2.3295091	3.9028E-10	1.3603E-09
2445	TSNAXIP1	-2.3294267	0.0009895	0.00189674
2446	RBFA	-2.3290324	3.7785E-54	7.8919E-53
2447	C1orf53	-2.3267762	3.2399E-09	1.0513E-08
2448	IDH2	-2.3260094	1.388E-52	2.7875E-51
2449	PINK1	-2.325468	3.2465E-64	9.0508E-63
2450	GRWD1	-2.3217002	3.7044E-46	6.1274E-45
2451	GATA2	-2.32115	0.02399497	0.03728348
2452	TTC22	-2.3196454	1.3726E-09	4.5864E-09
2453	GTF2IRD2B	-2.3184234	1.9618E-05	4.5703E-05
2454	BCL3	-2.3181159	8.6141E-24	6.5137E-23
2455	CPTP	-2.3179914	1.3369E-28	1.2484E-27
2456	CMPK2	-2.3168343	1.7091E-07	4.8037E-07
2457	VPREB3	-2.3168278	0.01763825	0.02805623
2458	ELOVL5	-2.3164638	7.7197E-26	6.3983E-25
2459	LPPR1	-2.316192	2.6737E-07	7.3826E-07
2460	TMEM56	-2.3159829	1.7135E-59	4.202E-58
2461	CNDP2	-2.3155587	1.987E-159	2.594E-157
2462	UAP1	-2.3151101	4.9813E-60	1.2488E-58
2463	PINK1-AS	-2.3139618	1.7459E-72	5.9595E-71
2464	L3MBTL4	-2.3122151	4.2002E-10	1.4611E-09
2465	ADCY5	-2.3118618	3.8993E-11	1.4645E-10
2466	ASL	-2.3116827	1.433E-21	9.7827E-21
2467	PPIP5K1	-2.3105106	2.6569E-18	1.5452E-17
2468	CRADD	-2.3100502	8.5953E-48	1.4893E-46
2469	UBXN11	-2.3082539	4.4067E-11	1.6503E-10
2470	SECTM1	-2.3073546	7.0754E-06	1.7203E-05

2471	CYB561A3	-2.3070412	1.0033E-65	2.9458E-64
2472	STARD5	-2.3069007	1.3989E-25	1.1466E-24
2473	RIN3	-2.3053525	4.547E-31	4.6187E-30
2474	FAM195A	-2.3025067	5.8599E-26	4.8904E-25
2475	HERC5	-2.3020691	4.6799E-06	1.157E-05
2476	HABP2	-2.3018951	8.8842E-28	8.0244E-27
2477	ARHGAP4	-2.3013592	2.4042E-06	6.1085E-06
2478	SLCO4A1	-2.3006782	1.2995E-07	3.6955E-07
2479	WASH1	-2.3002566	6.0031E-11	2.2231E-10
2480	EPB41L4B	-2.2981451	4.0018E-08	1.1867E-07
2481	HAGH	-2.2941436	3.0002E-27	2.6576E-26
2482	CRYBB3	-2.2932274	0.03201609	0.04860528
2483	NALCN	-2.2921365	0.0005332	0.00105578
2484	SUOX	-2.2893899	5.5655E-61	1.4261E-59
2485	SMAD1	-2.2886772	1.481E-21	1.0104E-20
2486	ABCG1	-2.287705	0.00080955	0.00156954
2487	GFOD1	-2.2863649	4.767E-07	1.2904E-06
2488	HPCAL1	-2.2860677	1.7423E-23	1.2972E-22
2489	SLC1A7	-2.2854527	0.00018897	0.00039504
2490	SYN1	-2.2846024	1.1321E-09	3.8072E-09
2491	APLN	-2.2833656	0.00661282	0.01127722
2492	HIST1H2BJ	-2.2830885	0.02577722	0.03979989
2493	CIART	-2.2826444	1.6204E-05	3.8095E-05
2494	DHCR24	-2.2819271	5.659E-15	2.7367E-14
2495	NUCB1-AS1	-2.281629	6.0884E-36	7.4167E-35
2496	ARMC6	-2.2796567	1.1063E-53	2.2835E-52
2497	ORMDL3	-2.2795954	7.1735E-46	1.1705E-44
2498	ATP5G2	-2.2765858	5.9943E-25	4.8054E-24
2499	FXYD6	-2.2757675	3.0306E-05	6.9276E-05
2500	PECR	-2.2731888	3.8627E-62	1.0279E-60
2501	RPLP0	-2.2730025	1.9982E-37	2.5549E-36
2502	IRF7	-2.2721327	5.1705E-36	6.3225E-35
2503	ESPNL	-2.2710335	0.00184802	0.00341761
2504	GALK2	-2.269883	1.0511E-39	1.4371E-38
2505	ANKRD37	-2.2698822	2.166E-10	7.7081E-10
2506	ZC3H12D	-2.2696919	0.01062931	0.01751905
2507	RRS1	-2.2663434	6.5343E-13	2.7661E-12
2508	TM6SF2	-2.2661851	3.1834E-11	1.2057E-10
2509	LRRC37A4P	-2.265522	0.01984033	0.03130125
2510	SLC40A1	-2.2624274	1.5929E-15	7.9645E-15
2511	CDC42EP1	-2.2616853	8.9488E-14	4.016E-13
2512	TMEM218	-2.2600526	5.0057E-26	4.1957E-25
2513	TTC16	-2.2588618	0.02791604	0.04282839
2514	ENTPD1-AS1	-2.2587262	0.00609592	0.01044923
2515	CADPS2	-2.2571058	6.8803E-12	2.7223E-11

2516	PRELID1	-2.2538466	3.7274E-56	8.3253E-55
2517	MTSS1	-2.2531135	1.0664E-66	3.2191E-65
2518	ATP5G1	-2.2524624	6.1235E-37	7.7172E-36
2519	LINC00999	-2.2523525	4.4842E-05	0.00010069
2520	ACADM	-2.2515	3.8167E-84	1.6496E-82
2521	ADAM8	-2.2510106	0.00011961	0.00025609
2522	ATP6V1E2	-2.2484383	0.00020506	0.00042654
2523	PPAP2A	-2.2456151	3.4843E-27	3.0766E-26
2524	MYO16	-2.2415234	3.5804E-06	8.9476E-06
2525	MGST2	-2.2412841	1.1343E-11	4.428E-11
2526	SERPIND1	-2.2400389	1.8577E-10	6.6415E-10
2527	KGFLP2	-2.2387384	0.005779	0.00993154
2528	STEAP1	-2.2382487	6.8408E-11	2.5261E-10
2529	ADCY4	-2.2378203	1.3657E-06	3.5489E-06
2530	DUT	-2.2370961	4.1147E-20	2.6142E-19
2531	DHRS4-AS1	-2.2357788	1.0959E-36	1.3668E-35
2532	CHRD	-2.2331617	7.1092E-14	3.2129E-13
2533	TFB2M	-2.2330287	1.427E-53	2.936E-52
2534	NSMCE1	-2.2323277	2.3892E-30	2.3671E-29
2535	PSAT1	-2.2315142	3.019E-20	1.9333E-19
2536	JOSD2	-2.2298144	3.6057E-17	1.9707E-16
2537	GRHPR	-2.2256606	5.352E-57	1.2165E-55
2538	NHP2	-2.2218446	3.7503E-46	6.1981E-45
2539	NLE1	-2.2210768	4.541E-06	1.1235E-05
2540	GAA	-2.2208865	1.7337E-21	1.1773E-20
2541	SNORA24	-2.2206651	0.00374023	0.00663313
2542	PLEKHH2	-2.2205643	1.0378E-05	2.4804E-05
2543	RAB43	-2.2202903	2.8055E-20	1.7996E-19
2544	FAM110C	-2.2197898	3.6097E-08	1.0754E-07
2545	PHKG1	-2.2180988	2.2369E-35	2.6661E-34
2546	JUNB	-2.2173017	6.2391E-13	2.644E-12
2547	HOXB5	-2.2156097	0.01377092	0.02229754
2548	MAGI2-AS3	-2.2145052	1.6629E-10	5.9581E-10
2549	ALDH6A1	-2.2144339	3.7471E-59	9.0946E-58
2550	VDR	-2.2140464	0.00027973	0.00057353
2551	TRUB2	-2.212265	9.8682E-61	2.5119E-59
2552	RHOB	-2.2095492	1.621E-10	5.8147E-10
2553	HVCN1	-2.2078919	1.0802E-06	2.8364E-06
2554	GADD45B	-2.2078789	1.874E-10	6.6961E-10
2555	C16orf86	-2.2072329	8.181E-07	2.1669E-06
2556	HMGB1	-2.2063966	1.3658E-22	9.762E-22
2557	HADH	-2.2059131	3.1369E-49	5.6692E-48
2558	HOXB6	-2.2038821	0.00280353	0.00506112
2559	TSPAN33	-2.203621	1.2318E-06	3.2126E-06
2560	LOC642236	-2.2035119	0.01742094	0.02773341

2561	C2CD2L	-2.2014146	1.8297E-33	2.0316E-32
2562	FOS	-2.2011603	7.2129E-10	2.4603E-09
2563	ACYP2	-2.1999155	2.6489E-16	1.3794E-15
2564	MMACHC	-2.1991784	2.317E-45	3.7335E-44
2565	PLXNA2	-2.1988822	8.3813E-06	2.0221E-05
2566	SIGLEC11	-2.1987875	0.00908946	0.01516349
2567	USP18	-2.1973291	5.362E-07	1.4424E-06
2568	TPI1P2	-2.1960795	0.00448747	0.00785591
2569	RARRES3	-2.1958047	9.1469E-09	2.8558E-08
2570	SPON2	-2.1946808	2.7333E-05	6.2753E-05
2571	NIPSNAP3A	-2.194096	3.9107E-35	4.607E-34
2572	CCPG1	-2.1934018	3.1901E-10	1.1204E-09
2573	GLB1L2	-2.1933717	0.03183593	0.04835837
2574	GPR137B	-2.1908532	2.6844E-06	6.7883E-06
2575	C19orf73	-2.1908312	0.00497244	0.00863528
2576	GLT8D1	-2.1907791	7.868E-65	2.2289E-63
2577	EEFSEC	-2.1896146	4.3008E-34	4.8792E-33
2578	IFI16	-2.1883843	1.0104E-05	2.4175E-05
2579	NR2F1-AS1	-2.1867044	0.0214459	0.03361742
2580	PPP4R4	-2.1864835	0.00014223	0.00030179
2581	FAU	-2.1851985	3.6188E-59	8.7944E-58
2582	ATP6V0E2	-2.1851485	2.8861E-13	1.2522E-12
2583	POLD4	-2.1845517	1.855E-48	3.279E-47
2584	CYP11A1	-2.1818493	1.9092E-07	5.3489E-07
2585	ACN9	-2.1813525	8.1038E-11	2.9782E-10
2586	ALG3	-2.1795167	7.3031E-61	1.8664E-59
2587	ABCC6P2	-2.1790221	0.00767547	0.01296735
2588	CSF1R	-2.1776073	2.417E-09	7.9201E-09
2589	TRIM22	-2.1775607	3.6655E-08	1.0913E-07
2590	PAN2	-2.1774655	6.116E-19	3.6685E-18
2591	RAPGEF4	-2.1763085	1.5036E-10	5.4075E-10
2592	SFXN2	-2.1758122	1.082E-30	1.082E-29
2593	TMPRSS9	-2.1749085	8.5902E-06	2.0699E-05
2594	EIF2B3	-2.1732204	9.7991E-23	7.0588E-22
2595	DESI1	-2.1730468	7.2055E-49	1.289E-47
2596	NFE2	-2.1717731	0.01495707	0.02407601
2597	PLGRKT	-2.170602	8.4604E-11	3.1046E-10
2598	MUT	-2.1704547	6.0699E-86	2.7209E-84
2599	SERINC2	-2.1698153	3.8445E-17	2.0964E-16
2600	B9D1	-2.1687966	3.2978E-12	1.3301E-11
2601	PGM1	-2.1679266	5.2108E-95	2.6918E-93
2602	CYCS	-2.1678347	2.3053E-13	1.0067E-12
2603	BCS1L	-2.1662263	1.3225E-17	7.3951E-17
2604	TSPAN7	-2.1660708	1.4202E-16	7.507E-16
2605	TMEM232	-2.1656552	0.02789168	0.04280121

2606	TMEM163	-2.1655026	0.01475525	0.0237718
2607	CTSL	-2.1653774	8.0354E-15	3.8446E-14
2608	SYDE1	-2.1653422	1.5688E-12	6.4789E-12
2609	LRP5L	-2.1649988	7.2153E-16	3.68E-15
2610	RPS6KL1	-2.1645853	0.00046512	0.00092688
2611	ABCB6	-2.1622583	1.5541E-35	1.8684E-34
2612	ARAP3	-2.1618338	4.5027E-09	1.4422E-08
2613	KL	-2.1617213	0.02298569	0.03583342
2614	FUCA1	-2.1609726	3.7576E-25	3.0363E-24
2615	SDHC	-2.1600849	2.1833E-39	2.9622E-38
2616	FOXF1	-2.1598815	0.00512023	0.00887519
2617	EI24	-2.1590116	8.037E-106	4.961E-104
2618	PITRM1-AS1	-2.1579433	4.3879E-20	2.7804E-19
2619	SRPRB	-2.1565923	3.0768E-89	1.4358E-87
2620	PDGFRB	-2.1545189	3.2301E-06	8.1151E-06
2621	NUDT16P1	-2.1539514	1.6917E-14	7.9331E-14
2622	COQ3	-2.1533611	6.9841E-18	3.9686E-17
2623	PLEKHG1	-2.1501398	4.7249E-05	0.00010584
2624	ZSCAN31	-2.1489277	2.5687E-07	7.1063E-07
2625	MPC1	-2.148872	2.9131E-17	1.6021E-16
2626	C5orf49	-2.1476947	2.9364E-06	7.3955E-06
2627	FLT3LG	-2.1474919	1.5973E-05	3.7569E-05
2628	MXD3	-2.1442363	9.1401E-51	1.7381E-49
2629	PRKCH	-2.1435896	0.0032915	0.00588159
2630	MEI4	-2.1430141	0.00183419	0.00339431
2631	TSPAN1	-2.1379135	2.4122E-12	9.8257E-12
2632	CDCA7L	-2.1371227	1.5917E-07	4.4873E-07
2633	ENO3	-2.1360223	1.3944E-16	7.377E-16
2634	RPL13	-2.1358039	1.4259E-58	3.4011E-57
2635	LINC00526	-2.1355383	1.6628E-11	6.42E-11
2636	SLAMF9	-2.1341663	0.01723265	0.02745175
2637	NPY1R	-2.1338052	1.9416E-07	5.4326E-07
2638	CROCCP2	-2.1311648	6.3061E-41	8.9584E-40
2639	ISCA1	-2.1309924	4.2508E-44	6.5965E-43
2640	DNAH6	-2.130681	0.02163579	0.03388484
2641	PCBD2	-2.1304553	6.1488E-20	3.8645E-19
2642	GUSBP5	-2.1276614	0.01890757	0.02993479
2643	TRIM34	-2.1272097	1.1712E-08	3.6304E-08
2644	TTLL11	-2.1267975	2.2657E-12	9.2487E-12
2645	FLJ37035	-2.1257302	0.02090723	0.03284231
2646	RABEPK	-2.1256054	1.3341E-29	1.2875E-28
2647	DISP2	-2.1253915	0.0005071	0.00100649
2648	RFFL	-2.1253599	8.5663E-53	1.7312E-51
2649	GNN	-2.1225095	0.01571068	0.02520388
2650	DLL4	-2.122418	6.78E-12	2.6842E-11

2651	KLHL29	-2.1224062	0.00110023	0.00209402
2652	ACOT6	-2.1218892	0.00148177	0.00277644
2653	C10orf25	-2.1215951	1.7081E-08	5.2201E-08
2654	EHHADH-AS1	-2.121496	8.7734E-19	5.209E-18
2655	FLJ32255	-2.1214209	5.3291E-06	1.3096E-05
2656	POLDIP2	-2.1213918	7.6986E-43	1.1593E-41
2657	GRINA	-2.1207998	9.7378E-84	4.1257E-82
2658	MRPL4	-2.1191003	4.9019E-76	1.7869E-74
2659	DYX1C1-CCPG1	-2.1189411	4.0048E-10	1.3944E-09
2660	MARS2	-2.1174183	1.2979E-22	9.2905E-22
2661	L3HYPDH	-2.1154344	1.2882E-25	1.0577E-24
2662	SLC25A13	-2.1121974	3.316E-37	4.2092E-36
2663	IQCG	-2.1115269	0.00015706	0.00033138
2664	GPI	-2.1093552	2.674E-34	3.0697E-33
2665	CRISPLD2	-2.1085926	0.0009828	0.00188501
2666	RPL17	-2.1083982	2.2649E-53	4.6451E-52
2667	CILP	-2.1080171	0.01115042	0.01831581
2668	C3orf70	-2.1067344	0.00092973	0.00178927
2669	RWDD2B	-2.1064225	7.1206E-21	4.6969E-20
2670	NR2F1	-2.1057108	0.00441481	0.00773574
2671	OGFR-AS1	-2.1050151	0.00015019	0.00031772
2672	GTF2IRD2	-2.102533	2.376E-06	6.0401E-06
2673	BCKDHA	-2.1023804	4.444E-20	2.8141E-19
2674	PYGM	-2.0989903	0.00257446	0.00467599
2675	MRPS28	-2.0982167	4.9606E-32	5.24E-31
2676	EIF3CL	-2.0981443	3.9535E-09	1.2719E-08
2677	BNIP3	-2.0979209	8.6723E-41	1.2257E-39
2678	NUDT5	-2.0977177	5.4259E-58	1.2706E-56
2679	SOD3	-2.0964994	0.00013672	0.00029091
2680	LOC257396	-2.0951444	0.00372137	0.0066021
2681	SLC39A14	-2.0943682	3.2117E-18	1.8605E-17
2682	HACL1	-2.0936759	1.7615E-48	3.1223E-47
2683	LGMN	-2.0934045	1.4555E-39	1.9817E-38
2684	ZADH2	-2.0921509	2.6629E-06	6.7392E-06
2685	EHHADH	-2.0920415	8.2978E-74	2.873E-72
2686	EIF3C	-2.0913077	4.3116E-09	1.3837E-08
2687	FKBP11	-2.0907674	6.3174E-20	3.9679E-19
2688	RNF13	-2.0895141	2.2355E-22	1.5797E-21
2689	LINC00346	-2.0889361	0.01934561	0.03058426
2690	A1CF	-2.0887265	1.9093E-12	7.8257E-12
2691	BOK-AS1	-2.0868102	2.6169E-05	6.0181E-05
2692	POLR3GL	-2.0864139	4.8171E-16	2.4785E-15
2693	RANBP10	-2.0854539	2.6097E-30	2.579E-29
2694	SPR	-2.0853711	4.9228E-35	5.7817E-34
2695	CSDC2	-2.0844974	1.7562E-08	5.3594E-08

2696	FAM120A	-2.0844092	5.4426E-63	1.481E-61
2697	LINC01128	-2.0830493	5.2395E-17	2.8347E-16
2698	DCAF11	-2.0820293	5.5516E-60	1.3876E-58
2699	COL25A1	-2.0811727	7.9872E-05	0.00017436
2700	ITGA8	-2.0809339	0.00074716	0.00145456
2701	PITRM1	-2.0807732	2.2923E-47	3.9123E-46
2702	RPRD1B	-2.0805577	2.8914E-75	1.0288E-73
2703	SQSTM1	-2.0798455	1.0962E-82	4.5447E-81
2704	PEX16	-2.0794346	5.4501E-34	6.1518E-33
2705	SEC61A2	-2.0790551	9.558E-36	1.1556E-34
2706	FARS2	-2.0788675	4.3131E-22	3.018E-21
2707	FAM129A	-2.07882	0.00017708	0.00037129
2708	DPP9	-2.078441	2.8174E-47	4.7916E-46
2709	CHST15	-2.076633	2.3077E-15	1.1429E-14
2710	SF3B5	-2.0762714	3.0522E-57	6.9704E-56
2711	C3orf14	-2.07614	0.00100257	0.00191893
2712	PROS1	-2.0748431	3.3384E-35	3.9448E-34
2713	PKHD1L1	-2.0745039	1.4265E-07	4.0428E-07
2714	RPSA	-2.0741098	1.2205E-29	1.1802E-28
2715	TNFSF13B	-2.07335	7.1355E-07	1.8968E-06
2716	C11orf71	-2.0727849	3.3577E-17	1.8382E-16
2717	KEAP1	-2.0716776	8.5259E-83	3.5577E-81
2718	ECE2	-2.071632	9.3893E-27	8.12E-26
2719	ADAMTSL1	-2.0708645	0.01102808	0.01813915
2720	CHST2	-2.0705108	0.03165293	0.04810688
2721	NUDT8	-2.0703633	6.1248E-20	3.8507E-19
2722	TARS2	-2.0703253	1.077E-29	1.043E-28
2723	CX3CL1	-2.069863	1.3261E-06	3.45E-06
2724	HHIP-AS1	-2.0679671	0.00400095	0.00705792
2725	RPH3AL	-2.0672895	1.7177E-20	1.111E-19
2726	NADK2	-2.0671299	1.7284E-58	4.1022E-57
2727	TUBA1B	-2.0656644	8.3171E-14	3.7386E-13
2728	SNORD60	-2.0654575	0.02474178	0.03832675
2729	TUT1	-2.0650501	4.5714E-40	6.3086E-39
2730	SRM	-2.0630297	1.2109E-43	1.8509E-42
2731	POLR2L	-2.0629988	7.7405E-84	3.3012E-82
2732	SLC25A25	-2.062837	8.4684E-08	2.4416E-07
2733	RPL10A	-2.0625146	5.8218E-33	6.3546E-32
2734	CISD3	-2.061086	1.5098E-51	2.9554E-50
2735	LOC101927559	-2.060716	2.1238E-11	8.1427E-11
2736	RBP7	-2.0592169	1.0101E-05	2.4169E-05
2737	BLOC1S1-RDH5	-2.0577161	1.362E-63	3.7325E-62
2738	SLC39A3	-2.0576215	1.4581E-41	2.118E-40
2739	TMEM143	-2.0563556	7.6203E-19	4.5454E-18
2740	HMCN1	-2.0556864	0.03115761	0.04743618

2741	SORD	-2.0552638	6.3903E-10	2.1902E-09
2742	PDLIM3	-2.0540285	4.7874E-06	1.1822E-05
2743	NCF2	-2.052674	4.0062E-05	9.0442E-05
2744	NMI	-2.0513772	7.9417E-07	2.1056E-06
2745	ESRRA	-2.050552	2.7714E-30	2.7374E-29
2746	SLC35D1	-2.0497366	1.2289E-22	8.8098E-22
2747	THADA	-2.0494694	2.1946E-51	4.2741E-50
2748	GBE1	-2.0485878	3.3605E-18	1.9439E-17
2749	CLPTM1L	-2.0450518	5.2603E-42	7.7402E-41
2750	SEC16A	-2.0444445	1.7894E-86	8.0965E-85
2751	RBM43	-2.0442421	2.1113E-07	5.8919E-07
2752	KMO	-2.0423257	2.5352E-07	7.0182E-07
2753	FAM101B	-2.0400473	2.8153E-09	9.1722E-09
2754	PRKAG2-AS1	-2.0395651	2.8832E-07	7.9439E-07
2755	RSU1	-2.0388498	4.8947E-28	4.4775E-27
2756	DDX28	-2.0386226	4.4129E-24	3.3953E-23
2757	PAQR4	-2.0380602	6.743E-09	2.1308E-08
2758	CBLN1	-2.0363657	0.01335746	0.02168623
2759	NID1	-2.0358794	2.0351E-14	9.4948E-14
2760	PREB	-2.035219	9.6183E-58	2.2335E-56
2761	LINC00997	-2.0347254	1.4214E-05	3.3575E-05
2762	RNF152	-2.034671	2.6031E-09	8.5081E-09
2763	MCC	-2.0335919	2.486E-14	1.1535E-13
2764	CD72	-2.0329253	1.9167E-07	5.3674E-07
2765	LRP1B	-2.0325262	0.00015815	0.00033354
2766	DECR2	-2.0324706	8.3554E-65	2.36E-63
2767	PSME2	-2.032141	1.3199E-63	3.6224E-62
2768	PDK1	-2.0318164	8.783E-17	4.694E-16
2769	TPPP2	-2.029972	0.01053393	0.01737963
2770	SYVN1	-2.0298162	3.9384E-42	5.8218E-41
2771	WDR45	-2.0294188	9.8645E-20	6.1595E-19
2772	LOC283788	-2.0287003	1.3262E-27	1.1906E-26
2773	COQ9	-2.0281789	4.1261E-19	2.4981E-18
2774	ZDHHC23	-2.0275587	1.1089E-17	6.2331E-17
2775	NLN	-2.0270696	6.0568E-32	6.3805E-31
2776	PLCXD3	-2.0267252	0.01559424	0.02502955
2777	LOC101927437	-2.024956	0.01992999	0.03142731
2778	SYCE1L	-2.0210373	8.5362E-07	2.2573E-06
2779	CMC4	-2.0204618	4.3489E-20	2.7566E-19
2780	PPAN	-2.0197901	6.5644E-24	5.0049E-23
2781	HNRNPA1P10	-2.0186474	9.67E-14	4.3296E-13
2782	HSPA4L	-2.0175054	2.2558E-18	1.3167E-17
2783	COX7B	-2.0175026	5.4036E-15	2.6165E-14
2784	CDNF	-2.0172301	1.7944E-05	4.2002E-05
2785	FOXP2	-2.0168724	2.6273E-06	6.6506E-06

2786	SH3PXD2A	-2.0159549	0.00016102	0.00033932
2787	STK32C	-2.0157388	0.01451244	0.02341381
2788	CCDC176	-2.0156931	9.4192E-32	9.8687E-31
2789	METRN	-2.0120629	3.6996E-20	2.3613E-19
2790	ADCY9	-2.0107662	8.2935E-17	4.4361E-16
2791	PPAN-P2RY11	-2.00945	1.4247E-31	1.4831E-30
2792	EEPD1	-2.0092149	5.2667E-26	4.4049E-25
2793	TXN	-2.0082426	2.3476E-24	1.8259E-23
2794	MYO1B	-2.0071176	3.1733E-15	1.5592E-14
2795	ARHGAP23	-2.0070514	0.00040245	0.00080849
2796	C2orf47	-2.0070376	2.5051E-43	3.8049E-42
2797	DUS3L	-2.0051527	1.3792E-28	1.286E-27
2798	HSPB7	-2.0051429	0.00101272	0.00193662
2799	LRRC32	-2.0047468	0.00012032	0.00025754
2800	ADI1	-2.0046556	4.8992E-23	3.5718E-22
2801	CREG1	-2.0028531	1.8148E-19	1.1213E-18
2802	KIAA1671	-2.0001014	4.6371E-26	3.8935E-25

Differentially expressed genes were genes with at least a 4-fold change in gene expression (|Log2FC| $\geq$ 2) and adjusted p-value < 0.05.
DEG, differentially expressed genes.

Supplementary Table S5. GSEA Enriched Gene Sets Between Developing Liver and Adult Liver Stage

Gene Set	Number of Mapped Genes	Normalized Enrichment Score	Nominal p-value	Adjusted FDR
HALLMARK_XENOBIOTIC_METABOLISM	198	-2.462108	0	0
HALLMARK_BILE_ACID_METABOLISM	112	-2.1457884	0	0
HALLMARK_COAGULATION	138	-2.1076825	0	0
HALLMARK_FATTY_ACID_METABOLISM	157	-2.022015	0	0
HALLMARK_INTERFERON_ALPHA_RESPONSE	95	-1.8721949	0	3.77E-04
HALLMARK_INTERFERON_GAMMA_RESPONSE	198	-1.8687034	0	3.14E-04
HALLMARK_ADIPOGENESIS	193	-1.8439053	0	2.69E-04
HALLMARK_PEROXISOME	104	-1.7791312	0	8.55E-04
HALLMARK_OXIDATIVE_PHOSPHORYLATION	184	-1.7322131	0	0.00155622
HALLMARK_ESTROGEN_RESPONSE_EARLY	195	-1.6892362	0	0.002099469
HALLMARK_IL6_JAK_STAT3_SIGNALING	86	-1.6181881	0	0.005116561
HALLMARK_MYOGENESIS	199	-1.5984846	0	0.006361592
HALLMARK_ESTROGEN_RESPONSE_LATE	197	-1.5044882	0.003476246	0.017742272
HALLMARK_KRAS_SIGNALING_DN	195	-1.4880034	0.004656578	0.019768484
HALLMARK_GLYCOLYSIS	197	-1.4808564	0.001140251	0.020105084
HALLMARK_APICAL_SURFACE	43	-1.4583807	0.034383953	0.024864819
HALLMARK_APICAL_JUNCTION	194	-1.3484024	0.013033175	0.07588724
HALLMARK_COMPLEMENT	198	-1.3178074	0.033838972	0.0978267
HALLMARK_ALLOGRAFT_REJECTION	199	-1.2975094	0.034285713	0.115136586
HALLMARK_INFLAMMATORY_RESPONSE	198	-1.2912956	0.03990878	0.115825266
HALLMARK_EPITHELIAL_MESENCHYMAL_TRANSITION	197	-1.2883506	0.030998852	0.113266304
HALLMARK_UNFOLDED_PROTEIN_RESPONSE	109	-1.245124	0.10974106	0.16340715
HALLMARK_ANDROGEN_RESPONSE	96	-1.241928	0.11308768	0.16084656

HALLMARK_REACTIVE_OXYGEN_SPECIES_PATHWAY	47	-1.2408462	0.14066853	0.15608045
HALLMARK_KRAS_SIGNALING_UP	195	-1.1579742	0.1529548	0.2956803
HALLMARK_PANCREAS_BETA_CELLS	40	-1.1194066	0.27873564	0.3754751
HALLMARK_NOTCH_SIGNALING	32	-1.099663	0.3179792	0.40971383
HALLMARK_P53_PATHWAY	197	-1.063865	0.31632653	0.48851454
HALLMARK_UV_RESPONSE_UP	156	-1.0521706	0.34610918	0.5050078
HALLMARK_CHOLESTEROL_HOMEOSTASIS	74	-1.0280637	0.4055118	0.5573402
HALLMARK_MYC_TARGETS_V2	58	-0.97485757	0.5046729	0.69084543
HALLMARK_PROTEIN_SECRETION	95	-0.96114844	0.54785895	0.7099009
HALLMARK_TGF_BETA_SIGNALING	54	-0.93494785	0.5769764	0.7620648
HALLMARK_PI3K_AKT_MTOR_SIGNALING	104	-0.89269847	0.7001304	0.85299206
HALLMARK_HYPOXIA	192	-0.88039273	0.77030164	0.85872376
HALLMARK_MTORC1_SIGNALING	198	-0.87643725	0.76834863	0.8435968
HALLMARK_WNT_BETA_CATENIN_SIGNALING	42	-0.85552573	0.70494187	0.867774
HALLMARK_HEDGEHOG_SIGNALING	35	-0.8398615	0.7251462	0.8793849
HALLMARK_IL2_STAT5_SIGNALING	196	-0.8150225	0.89411765	0.9041189
HALLMARK_APOPTOSIS	160	-0.77164274	0.9138756	0.9483105
HALLMARK_ANGIOGENESIS	36	-0.5851211	0.989781	0.99835086
HALLMARK_UV_RESPONSE_DN	137	0.8927917	0.8032787	0.77347726
HALLMARK_TNFA_SIGNALING_VIA_NFKB	197	0.9870906	0.49624062	0.5501477
HALLMARK_SPERMATOGENESIS	134	1.5814774	0	0.005138465
HALLMARK_DNA_REPAIR	147	1.7006581	0	0.00115
HALLMARK_MYC_TARGETS_V1	197	1.7175673	0	0.001155
HALLMARK_MITOTIC_SPINDLE	197	1.9948641	0	0
HALLMARK_HEME_METABOLISM	195	2.9883566	0	0
HALLMARK_G2M_CHECKPOINT	192	3.1803746	0	0
HALLMARK_E2F_TARGETS	197	3.2499802	0	0

Negative normalized enrichment scores represent enrichment in adult compared to developing liver stage. Positive normalized enrichment scores represent enrichment in developing liver compared to adult stage.
GSEA, gene set enrichment analysis; FDR, false discovery rate.

Supplementary Table S6. GSEA Enriched Gene Sets Between iHLC and Adult Liver Stage

Gene Set	Number of Mapped Genes	Normalized Enrichment Score	Nominal p-value	Adjusted FDR
HALLMARK_XENOBIOTIC_METABOLISM	199	-2.3753562	0	0
HALLMARK_INTERFERON_GAMMA_RESPONSE	199	-2.3076956	0	0
HALLMARK_INTERFERON_ALPHA_RESPONSE	95	-2.1519835	0	0
HALLMARK_ALLOGRAFT_REJECTION	199	-2.13837	0	0
HALLMARK_BILE_ACID_METABOLISM	112	-2.0998394	0	0
HALLMARK_FATTY_ACID_METABOLISM	157	-2.0892491	0	0
HALLMARK_OXIDATIVE_PHOSPHORYLATION	184	-2.0591	0	0
HALLMARK_IL6_JAK_STAT3_SIGNALING	86	-2.0268047	0	0
HALLMARK_ADIPOGENESIS	194	-1.989455	0	0
HALLMARK_COMPLEMENT	198	-1.8821075	0	8.44E-05
HALLMARK_COAGULATION	138	-1.8797015	0	7.68E-05
HALLMARK_INFLAMMATORY_RESPONSE	198	-1.80643	0	1.41E-04
HALLMARK_REACTIVE_OXYGEN_SPECIES_PATHWAY	47	-1.803641	0	1.30E-04
HALLMARK_MYC_TARGETS_V2	58	-1.748814	0.00129199	8.05E-04
HALLMARK_KRAS_SIGNALING_UP	195	-1.7238301	0	8.10E-04
HALLMARK_PEROXISOME	104	-1.6420039	0	0.00170994
HALLMARK_MYC_TARGETS_V1	197	-1.5960484	0	0.00306569
HALLMARK_IL2_STAT5_SIGNALING	196	-1.5587876	0.00110011	0.00494025
HALLMARK_PANCREAS_BETA_CELLS	40	-1.3610312	0.065876156	0.04869716
HALLMARK_APOPTOSIS	160	-1.3118336	0.03262092	0.07746864
HALLMARK_MTORC1_SIGNALING	198	-1.2414348	0.060540542	0.15160577
HALLMARK_CHOLESTEROL_HOMEOSTASIS	74	-1.2003199	0.1497462	0.21019456

HALLMARK_MYOGENESIS	199	-1.1950569	0.10432852	0.21023501
HALLMARK_ESTROGEN_RESPONSE_LATE	197	-1.1843537	0.120176405	0.21999861
HALLMARK_APICAL_SURFACE	43	-1.1661731	0.22764228	0.24526797
HALLMARK_UV_RESPONSE_UP	156	-1.1581572	0.15437788	0.25171155
HALLMARK_GLYCOLYSIS	197	-1.1574892	0.14571746	0.24361797
HALLMARK_UNFOLDED_PROTEIN_RESPONSE	109	-1.0776747	0.32223544	0.41994712
HALLMARK_KRAS_SIGNALING_DN	195	-1.0711917	0.3150838	0.42243275
HALLMARK_HEME_METABOLISM	197	-1.0701828	0.29726776	0.410725
HALLMARK_ESTROGEN_RESPONSE_EARLY	195	-1.0352914	0.39121115	0.48698404
HALLMARK_NOTCH_SIGNALING	32	-1.0313777	0.41343284	0.48119068
HALLMARK_TNFA_SIGNALING_VIA_NFKB	197	-1.023313	0.42387733	0.48750532
HALLMARK_HYPOXIA	192	-0.9874924	0.5066225	0.56303906
HALLMARK_DNA_REPAIR	147	-0.9783316	0.5286041	0.5687577
HALLMARK_PI3K_AKT_MTOR_SIGNALING	104	-0.92094177	0.6311971	0.6908956
HALLMARK_APICAL_JUNCTION	194	-0.87855685	0.7738359	0.7660281
HALLMARK_SPERMATOGENESIS	135	-0.77196366	0.9334862	0.924873
HALLMARK_WNT_BETA_CATENIN_SIGNALING	42	0.9497257	0.56666666	0.6003366
HALLMARK_P53_PATHWAY	197	0.9724971	0.60227275	0.57507306
HALLMARK_HEDGEHOG_SIGNALING	35	1.0015094	0.4065934	0.52705264
HALLMARK_ANDROGEN_RESPONSE	96	1.0101432	0.4	0.5480038
HALLMARK_ANGIOGENESIS	36	1.0496497	0.3463035	0.46576643
HALLMARK_E2F_TARGETS	197	1.277317	0	0.0800494
HALLMARK_UV_RESPONSE_DN	137	1.3021833	0.008064516	0.07719496
HALLMARK_PROTEIN_SECRETION	95	1.3185314	0.010928961	0.07986575
HALLMARK_EPITHELIAL_MESENCHYMAL_TRANSITION	197	1.3703502	0	0.06157754
HALLMARK_TGF_BETA_SIGNALING	54	1.4484878	0.02631579	0.04461502
HALLMARK_G2M_CHECKPOINT	192	1.5852575	0	0.01786948
HALLMARK_MITOTIC_SPINDLE	198	1.9302305	0	0

Negative normalized enrichment scores represent enrichment in adult stage compared to iHLC. Positive normalized enrichment scores represent enrichment in iHLC compared to adult stage.

GSEA, gene set enrichment analysis; iHLC, induced pluripotent stem cell-derived hepatocyte-like cells; FDR, false discovery rate.

Supplementary Table S7. RNA-sequencing Details for Liver and iHLC Samples

Sample Name	Sequencing Platform	Type of Sequencing	Total Number of Reads
Fetal/Infant Liver #1	Illumina HiSeq 2000	Single-end (50 bp)	34,422,257
Fetal/Infant Liver #2	Illumina HiSeq 2000	Single-end (50 bp)	34,422,681
Fetal/Infant Liver #3	Illumina HiSeq 2000	Single-end (50 bp)	34,376,029
Fetal/Infant Liver #4	Illumina HiSeq 2000	Single-end (50 bp)	59,930,170
iHLC #1	Illumina NextSeq 500	Single-end (35-38 bp)	19,017,296
iHLC #2	Illumina NextSeq 500	Single-end (35-38 bp)	41,317,108
iHLC #3	Illumina NextSeq 500	Single-end (35-38 bp)	32,567,698
iHLC #4	Illumina NextSeq 500	Single-end (35-38 bp)	44,497,087
iHLC #5	Illumina NextSeq 500	Single-end (35-38 bp)	15,677,295
iHLC #6	Illumina NextSeq 500	Single-end (35-38 bp)	13,110,794

iHLC, induced pluripotent stem cell-derived hepatocyte-like cells

Supplementary Table S8. Primer Information

Gene	Forward Sequence	Reverse Sequence
β-Actin	GCACTCTTCCAGCCTTCC	TGTCCACGTCACACTTCATG
OCT4	TCTCCCATGCATTCAAACTGAG	CCTTTGTGTTCCCAATTCCTTC
HNF4a	AAGCCGTCCAGAATGAGC	AATGTCGCCGTTGATCCC
TTR	AGCCATCACAGAAGTCCAC	AGCCTCAGACACAAATACCAG
A1AT	ACTTGGCTTCCAGAATGAGG	CGCTCTTCAGATCATAGGTTCC
AFP	CTGCAATTGAGAAACCCACTG	TTCCCTCTTCACTTTGGCTG
Albumin	CCTGATTACTCTGTCGTGCTG	ATTCTGAGGCTCTTCCACAAG
ABCG5	GCTCGCAGGAACCGAATTG	GGTAACCGCAGTCATTGAAGAAA
ABCG8	AGGCCCAGCGTGACAAAAG	TGGGTTCGTCGAGAATAAGGA

Supplementary Table S9: Antibody information

Target Antigen	Distributor	Host	Catalogue Number	Dilution	Application
OCT 3/4	Santa Cruz	Mouse	sc-5279	1:500	ICC
SOX17	R&D Systems	Goat	AF1924	1:250	ICC
HNF4a	Santa Cruz	Mouse	sc-374229	1:250	ICC
HNF4a	Santa Cruz	Goat	sc-6556	1:250	ICC
AFP	Sigma	Mouse	A8452	1:1000	ICC
Albumin	Cedarlane	Mouse	CL2513A	1:1000	ICC, Flow cytometry
Alexa fluor Anti-Goat IgG, 568nm	Molecular Probes	Donkey	A-11057	1:1000	ICC
Alexa fluor Anti-Mouse IgG, 488nm	Molecular Probes	Donkey	A-21202	1:1000	ICC, Flow cytometry
ABCG8	Novus Biologicals	Rabbit	NB400-117	1:500	Western blot
IRDye 800CW Anti-Rabbit IgG	LICOR	Donkey	926-32213	1:10000	Western blot
ß-actin	Life Technologies	Mouse	A1978	1:1000	Western blot
IRDye 800CW Anti-Mouse IgG	LICOR	Donkey	926-32212	1:10000	Western blot

ICC, Immunocytochemistry
